# Genome-wide characterization of cellulases from the hemi-biotrophic plant pathogen, *Bipolaris sorokiniana*, reveals the presence of a highly stable GH7 endoglucanase

**DOI:** 10.1186/s13068-017-0822-0

**Published:** 2017-05-25

**Authors:** Shritama Aich, Ravi K. Singh, Pritha Kundu, Shree P. Pandey, Supratim Datta

**Affiliations:** 1 Department of Biological Sciences, Indian Institute of Science Education and Research Kolkata, Mohanpur, 741246 India; 2 Protein Engineering Laboratory, Department of Biological Sciences, Indian Institute of Science Education and Research Kolkata, Mohanpur, India; 3 Centre for Advanced Functional Materials, Indian Institute of Science Education and Research Kolkata, Mohanpur, India

**Keywords:** *Bipolaris sorokiniana*, Cell wall-degrading enzymes, Glycosyl hydrolase, GH7 endoglucanases, Salt tolerant, Alkaliphilic, Thermostable, Ionic liquids

## Abstract

**Background:**

*Bipolaris sorokiniana* is a filamentous fungus that causes spot blotch disease in cereals like wheat and has severe economic consequences. However, information on the identities and role of the cell wall-degrading enzymes (CWDE) in *B. sorokiniana* is very limited. Several fungi produce CWDE like glycosyl hydrolases (GHs) that help in host cell invasion. To understand the role of these CWDE in *B. sorokiniana*, the first step is to identify and annotate all possible genes of the GH families like GH3, GH6, GH7, GH45 and AA9 and then characterize them biochemically.

**Results:**

We confirmed and annotated the homologs of GH3, GH6, GH7, GH45 and AA9 enzymes in the *B. sorokiniana* genome using the sequence and domain features of these families. Quantitative real-time PCR analyses of these homologs revealed that the transcripts of the *BsGH7*-*3* (3rd homolog of the GH 7 family in *B. sorokiniana*) were most abundant. *BsGH7*-*3,* the gene of BsGH7-3, was thus cloned into pPICZαC *Pichia pastoris* vector and expressed in X33 *P. pastoris* host to be characterized. BsGH7-3 enzyme showed a temperature optimum of 60 °C and a pH_opt_ of 8.1. BsGH7-3 was identified to be an endoglucanase based on its broad substrate specificity and structural comparisons with other such endoglucanases. BsGH7-3 has a very long half-life and retains 100% activity even in the presence of 4 M NaCl, 4 M KCl and 20% (v/v) ionic liquids. The enzyme activity is stimulated up to fivefold in the presence of Mn^+2^ and Fe^+2^ without any deleterious effects on enzyme thermostability.

**Conclusions:**

Here we reanalysed the *B. sorokiniana* genome and selected one GH7 enzyme for further characterization. The present work demonstrates that BsGH7-3 is an endoglucanase with a long half-life and no loss in activity in the presence of denaturants like salt and ionic liquids, and lays the foundation towards exploring the *Bipolaris* genome for other cell wall-degrading enzymes.

**Electronic supplementary material:**

The online version of this article (doi:10.1186/s13068-017-0822-0) contains supplementary material, which is available to authorized users.

## Background

Biofuels produced from lignocellulosic biomass has many potential benefits over first-generation biofuel, including lower CO_2_ emissions and no competition with food for human consumption. In lignocellulose, the cellulose and hemicellulose are embedded in a lignin matrix and not easily accessible to enzymes. Lignocellulolytic fungi can be an efficient source of specialized enzymes that aid in the degradation of complex plant cell wall components to produce sugars. The exact nature and relative abundances of these enzymes vary from one plant species to another or across tissues within a plant. One of the best known example is the cellulase cocktail, secreted by the soft rot fungus *Trichoderma reesei* in large quantities [1]. Recently, it was reported that *T. reesei* being a necrophyte lacks several protein families related to infection and degradation of living plant tissue [2]. One way to get around this limitation is to add the missing enzymes in the cellulase cocktail or to manipulate the hydrolytic efficiency of cellulolytic enzymes encoded in this model organism. Another strategy could be to explore the fungal biodiversity for synergistic enzyme activities in order to supplement and increase the hydrolytic yield achieved by a *T. reesei* cocktail or, if possible, a new and more active cocktail based on enzymes from other organisms [3, 4].

Phytopathogenic fungi produce cell wall-degrading enzymes (CWDE) that are thought to aid their invasion into host cells [5]. A major group of CWDE consists of cellulases, which are glycosyl hydrolases (GHs) and catalyse hydrolysis of the β-1,4-glycosidic bonds in cellulose. Some of the CWDE-coding gene families have expanded during evolution among different groups of fungi [6, 7]. Further, these enzymes also show preference for specific types of plant biomass [8, 9]. Cellulases can be classified into three major types, namely endoglucanases (EG), cellobiohydrolases (CBH) and β-glucosidases (BG), all of which work synergistically to efficiently degrade cellulose [10–12].

*Cochliobolus sativus* (anamorph *Bipolaris sorokiniana*) is a fungal pathogen that causes spot blotch of wheat and barley and poses a severe challenge to their farming worldwide [13]. This fungal pathogen displays an enormous variability in its pathogenic, morphological and physiological forms. On the basis of their colony colour and growth behaviour, *B. sorokiniana* is broadly grouped into three categories. The black strain with thick dark mycelia are the most sporulating and aggressive kind, and the puffy white cotton-like mycelial strain is least sporulating but grows aggressively, while the mixed strain with greyish white mycelial growth has an intermediate number of spores and is the least aggressive [14, 15]. In addition, *Bipolaris* also attacks many grasses, including switch grass that is currently being developed as a bioenergy crop for biofuel production [16].

In 1999, Geimba et al. reported the partial purification and characterization of a BG from *B. sorokiniana* [17]. The same group in 2002 reported the presence of β-xylosidase, cellobiohydrolase and chitobiohydrolase activities in six isolates of *B. sorokiniana* originating from different areas of Brazil [18]. While a few loci have been stated to contain domains of the cellulolytic enzymes in *B. sorokiniana* genome (genome portal: Joint Genome Institute (JGI), University of California; http://genome.jgi-psf.org), systematic analysis of such genes across the genome or characterization of cellulase activities has not yet been reported [19]. A detailed characterization of these genes would be the first step towards the biotechnological application of these enzymes in biomass hydrolysis in addition or as an alternative to *T. reesei* cellulases and also for developing novel approaches towards biological control of pathogens. Here, we describe an integrative genomics approach to study the *B. sorokiniana* GHs and report the biochemical characterization of a novel GH7 endoglucanase.

## Methods

### Identification and analysis of glycoside hydrolases (GH) homologs in *B. sorokiniana*

The draft genome of *B. sorokiniana* lists 273 loci that are predicted to contain the domains of cellulases [19, 20]. In order to confirm and further annotate all possible genes of glycoside hydrolase family, we reanalysed the *B. sorokiniana* genome using the HMM (Hidden Markov model) profile-based search and phylogeny-based clustering methods. We used protein sequences of the eukaryotic glycoside hydrolases, GH3, GH6, GH7, GH45 and GH61 [auxiliary activity family 9 (AA9)] from the CAZy database (http://www.cazy.org/), to construct the HMM profiles for each of the five GH family members [21]. The redundant sequences were removed from the dataset of each family using CDHIT [22]. Then, multiple sequence alignment (MSA) of each of the GH family members was performed using MAFFT v 7.123b with default parameters [23]. These MSAs were used to construct the HMM profiles for each of the GH family members. Using these HMM profiles, the predicted proteome of *B. sorokiniana* was searched by HMMER program with the *E value* set to ≤10^−5^. The predicted homologs were searched for the presence and distribution of domains using Pfam database [24]. The *B. sorokiniana* genes are prefixed as “Bs” followed by their family names. If a family contained more than one gene, they were sequentially numbered as per standard practice [25–28]. For example, GH7 family in *B. sorokiniana* contains six homologous genes, and therefore these are named as *BsGH7*-*1* to *BsGH7*-*6*. Following a commonly accepted nomenclature, references to gene names and transcripts are italicized, whereas those to proteins are straight. Phylogenetic clustering of *B. sorokiniana* GH family members (GH3, GH6, GH7, GH45 and AA9) was performed by maximum likelihood (ML) method using RAxMLv7.2.8 [29]. We also used one bacterial protein from each family (GenBank IDs: AJP42775.1, AIF91560.1, AIQ82274.1 and AIF91527.1 for GH3, GH6, GH7 and GH45 families, respectively) as an out-group for phylogenetic analysis [30, 31]. Clade robustness was assessed with 1000 bootstrap replications. FigTree was used to visualize the phylogenetic tree (http://beast.bio.ed.ac.uk/FigTree). The evolutionary divergence among GH family members was estimated using MEGA 6 [32]. The genomic architecture of homologs for each GH family member was generated using GSDS v2.0 server [33]. The coordinates of the intron–exon boundary were calculated using program ‘blastn’ on the genome sequence of *B. sorokiniana* (available at http://genome.jgi-psf.org/Cocsa1/Cocsa1.home.html) [19]. Percent identities between paralogs of each GH family were calculated with the help of Clustal Omega [34]. We used the HHpred server (http://toolkit.tuebingen.mpg.de/hhpred/) to model the *B. sorokiniana* GH structure [35]. HHpred, at first, detects remote protein homology and then predicts structures from pairwise comparison of HMM profiles (through various databases, such as PDB, SCOP, Pfam, SMART, COGs and CDD) to produce query-template alignments. Further, it generates 3D structural models from these alignments. Root mean square deviations (RMSD; Å) between structures were calculated using TMalign server (http://zhanglab.ccmb.med.umich.edu/TM-align/) [36]. Area and volume of the binding pocket on the structures were calculated using CASTp server (http://sts.bioe.uic.edu/castp/calculation.php) [37]. Distribution and arrangement of positive electrostatic patches on the structures were calculated using Patch Finder plus server (http://pfp.technion.ac.il/index.html) [38]. Pymol was used to visualize modelled structures and prepare figures [39].

### Culture maintenance and propagation

*Bipolaris sorokiniana* was maintained under standard conditions recommended for culturing this fungus. Potato dextrose agar (PDA) has been used as a common medium for isolating *Bipolaris* from natural populations of wheat and barley and for maintaining and manipulating it in the laboratory [13–15, 40, 41]. The HD3069 strain with black morphology was maintained on PDA under complete darkness at 25 °C in a fungal incubator (Eyela, Model SU-1201) [13, 14]. The black strains are aggressive, produce maximum spores and are often used in characterization of plant responses to spot blotch attack [13–15]. Ten-day-old PDA plates were used for the collection of mycelial mass for isolation of nucleic acids.

### Transcriptional profiling by quantitative real-time PCR

Approximately 200 mg of crushed mycelial mass was used for RNA isolation. Total RNA of *B. sorokiniana* was extracted using the Trizol method following the manufacturer’s instructions and treated with DNase enzyme (Invitrogen, Carlsbad, USA). cDNA was prepared using a Superscript III First-strand synthesis system, oligo-dT primers and 5 µg of DNase-treated RNA following the manufacturer’s protocol (Life Technologies, Carlsbad, USA).

Gene-specific primers for each GH family homolog (Additional file 1: Table S2a) were designed using Primer Express software version 3.0 (Applied Biosystems; http://www.appliedbiosystems.com). SYBR green chemistry (KAPA Biosystems, Wilmington, USA) was used to estimate the transcript abundance of the *B. sorokiniana GH*s using gene-specific primers. For determining the absolute amount of transcript, a standard curve was prepared for each of the genes using cDNA amounts corresponding to 50, 100, 150 and 200 ng of total RNA in four replicates. Based on the formulae obtained from the standard curve, cDNA corresponding to 150 ng of total RNA was used to evaluate the absolute transcript amount based on their respective CT values. Three independent experiments were conducted, each comprising four replicates, the mean values were used to plot the graph. Elongation factor alpha (EF-α) was used as an endogenous control.

### Statistical analysis

The software Assistat 7.6 beta was used for statistical analysis to determine the significance of differences in the expression among the *GH* members under study. Duncan multiple range test (DMRT) was performed at a level of 5% probability (*p* ≤ 0.05).

### Cloning of the *BsGH7*-*3* gene

All the chemicals used were of reagent grade. Medium for cell growth was purchased from HiMedia Laboratories (Mumbai, India). Restriction enzymes and polymerase enzyme used for PCR were from New England Biolabs (Beverly, USA) and Taq Polymerase from Biobharati LifeScience (Kolkata, India). *Escherichia coli* Top10F’ cloning strain, *Pichia pastoris* yeast expression strain X33 and the vectors were from Life Technologies (Carlsbad, USA). The fraction obtained post purification was buffer-exchanged using a 30 kDa cut-off size membrane of Amicon-Ultra-15 (Millipore, Darmstadt, Germany). Substrate and other reagents for enzymatic assays were purchased from Sigma-Aldrich (St Louis, USA).

*BsGH7*-*3* was PCR amplified using the gene-specific primers (Additional file 1: Table S2b). The cDNA template was PCR amplified by Phusion™ high-fidelity DNA polymerase on a Veriti^®^ thermal cycler (Life technologies, Carlsbad, USA) using 54–60 °C temperature gradient to identify the optimum conditions. PCR products were separated using 1% agarose gel electrophoresis and specific DNA fragments were extracted using the QIAquick Gel extraction kit (Qiagen, Hilden, Germany). The gel-purified DNA was digested with *Xho*I and *Not*I-HF and ligated to the linearized pPICZαC vector. The ligated product was transformed into *E. coli* Top10F’ and verified by colony PCR, unique site restriction digestion and DNA sequencing using 5′α-factor and the 3′AOX1 (universal primer) of pPICZαC as the sequencing primers.

### Expression and purification of the protein

The plasmid construct was linearized using the unique site restriction enzyme, *Sac*I within the 5′AOX1 region and integrated into the X33 *P. pastoris* host genome by transformation of the linearized construct into the X33 competent cells following the instructions provided with the Pichia EasyComp™ kit (Life technologies, Carlsbad, USA). Colony PCR (as per standard protocol) was used to screen for positively integrated *Pichia* clones and the Mut (methanol utilization) phenotype identified following the manufacturer’s protocol (EasySelect™ Pichia expression kit, Life Technologies, Carlsbad, USA). Phenotype determination is required to verify if the *AOX1* gene is intact towards identifying the best medium for conducting the expression studies. To overexpress BsGH7-3, a 100 mL primary culture was grown in buffered complex glycerol (BMGY) medium with 100 µg mL^−1^ of zeocin. At O.D. 2.0, the cells were harvested by centrifugation at 3000×*g* for 8 min and the pellet dissolved in buffered complex methanol (BMMY) medium such that the O.D. of the starter culture was 1.0. Cells were induced with 0.5% methanol every 24 h and grown for 96 h. The protein secreted in the medium was precipitated with 50–80% ammonium sulphate and the cell pellet dialysed against 20 mM phosphate buffer, pH 7.3. The protein was further purified by passing through a Macro-Prep Q column (Bio-Rad Laboratories, Hercules, USA) equilibrated with 20 mM Tris–HCl buffer, pH 7.0, and eluted by 20 mM Tris–HCl/500 mM NaCl, pH 7.0. After desalting the protein with 20 mM phosphate buffer (pH 7.3), concentration was measured by Bradford assay with BSA and A_280_ and purity assessed by SDS-PAGE [42].

### Enzyme activity assays

Activity of BsGH7-3 was measured by mixing 1 μg of enzyme and 2% carboxymethyl cellulose (CMC; low viscosity of 100 cps at 25 °C in 4% water and degree of polymerization 0.7) as a substrate in McIlvaine buffer to a total reaction volume of 150 μL and incubating the enzyme at *T*_opt_. DNS (3,5-dinitrosalicylic acid) assay was performed to measure the reducing ends of CMC after enzymatic reaction [43]. 150 μL of DNS reagent (1.3 M DNS, 1 M potassium sodium tartrate and 0.4 N NaOH) was added and the reaction mixture incubated at 95 °C for 5 min. Absorbance was measured at 540 nm after cooling the reaction mix to room temperature. One unit of endoglucanase activity is the amount of enzyme required to release 1 nmol of reducing sugar per minute from the substrate. Glucose was used as the standard for the estimation of reducing sugars. All assays were performed in triplicate and standard deviation was calculated.

### Determination of pH and temperature optima of BsGH7-3

Using CMC as the substrate, the effect of temperature on enzyme activity was measured from 50 to 68 °C after incubating the enzyme in a buffer of optimum pH for 30 min. The optimal pH (pH_opt_) was measured by quantitating the enzyme activity on CMC over a pH range of 5.2–8.6 using McIlvaine buffer (pH 5.2–8.1) and Tris–HCl buffer (pH 8.0 and 8.6). pH stability was measured by determining residual activity after incubating the enzyme in McIlvaine buffer pH 8.1 for 6 h at 4 °C.

### Effect of salt, metal ions, ionic liquids and detergents on BsGH7-3 activity

The effects of additives were determined by measuring enzyme activity in the presence of salt, metal ions, ionic liquids and commercial detergents (Ariel™, Tide™, Sunlight™ and SDS) in McIlvaine buffer, pH 8.1. The additives were co-incubated with enzyme at 4 °C for 1 h before measuring the enzyme activity by standard activity assay. The specific activity without any additives was considered as 100% and relative activity in the presence of additives was estimated.

### Thermostability and half-life assay

The thermostability of the enzyme was determined by incubating the enzyme in McIlvaine buffer, pH 8.1, at 60 °C. Residual enzyme activity was measured by removing aliquots at regular intervals to measure enzyme activity. The enzyme stability was also checked by assaying the enzyme after 30 days of incubation at 4 °C in 10 mM phosphate buffer, pH 7.1.

### Substrate specificity and kinetic parameters of BsGH7-3 with CMC as a substrate

The specificity of BsGH7-3 was determined by measuring specific activity across a range of substrates, namely lichenan (MP Biomedicals, Ohio, USA), β-d-glucan from barley, Avicel PH-101, CMC (Sigma-Aldrich, Saint Louis, USA) and phosphoric acid swollen cellulose (PASC). PASC was prepared following the protocol of Walseth et al. [44]. Activity was determined by incubating 1 µg of enzyme with 0.8% substrate (w/v) at 60 °C for 30 min in McIlvaine buffer pH 8.1 and then measuring the reducing sugar generated by DNS assay. The specific activity on CMC was considered to be 100% and the relative activity on other substrates was estimated. The Michaelis–Menten parameters of GH7-3 on CMC was measured between 0.5 and 18 mg mL^−1^ of CMC and determined by a non-linear regression fit of Michaelis–Menten equation using GraphPad PRISM version 7.0 (GraphPad Software, La Jolla, CA).

### Effect of GH7-3 on the reduction of substrate viscosity

30% (w/v) of substrate (lichenan and β-d-glucan) in McIlvaine buffer, pH 8.1, was incubated with 36 µg of GH7-3 for 60 min at 60 °C and cooled to room temperature. A capillary viscometer was used to measure the viscosity of the supernatant (6 mL) at room temperature with substrate viscosity in the absence of enzyme as the control. The viscosity reduction was calculated using the following equations [45]:

1 $$\mu = \left( {\mu_{\text{water}} \times t \times \, \rho } \right)/\left( {t_{\text{water}} \times \, \rho_{\text{water}} } \right)$$

2 $$\Delta \mu = \left( {\mu_{\text{control}} - \mu } \right) \times 100/\left( {\mu_{\text{control}} } \right),$$

where *μ* is the viscosity, *t* is the total flow time through viscometer, Δ*μ* is the reduction of viscosity and *ρ* is the density.

## Results

### Glycoside hydrolase (GH) in *B. sorokiniana*: annotation and sequence characterization

**Fig. 1:**
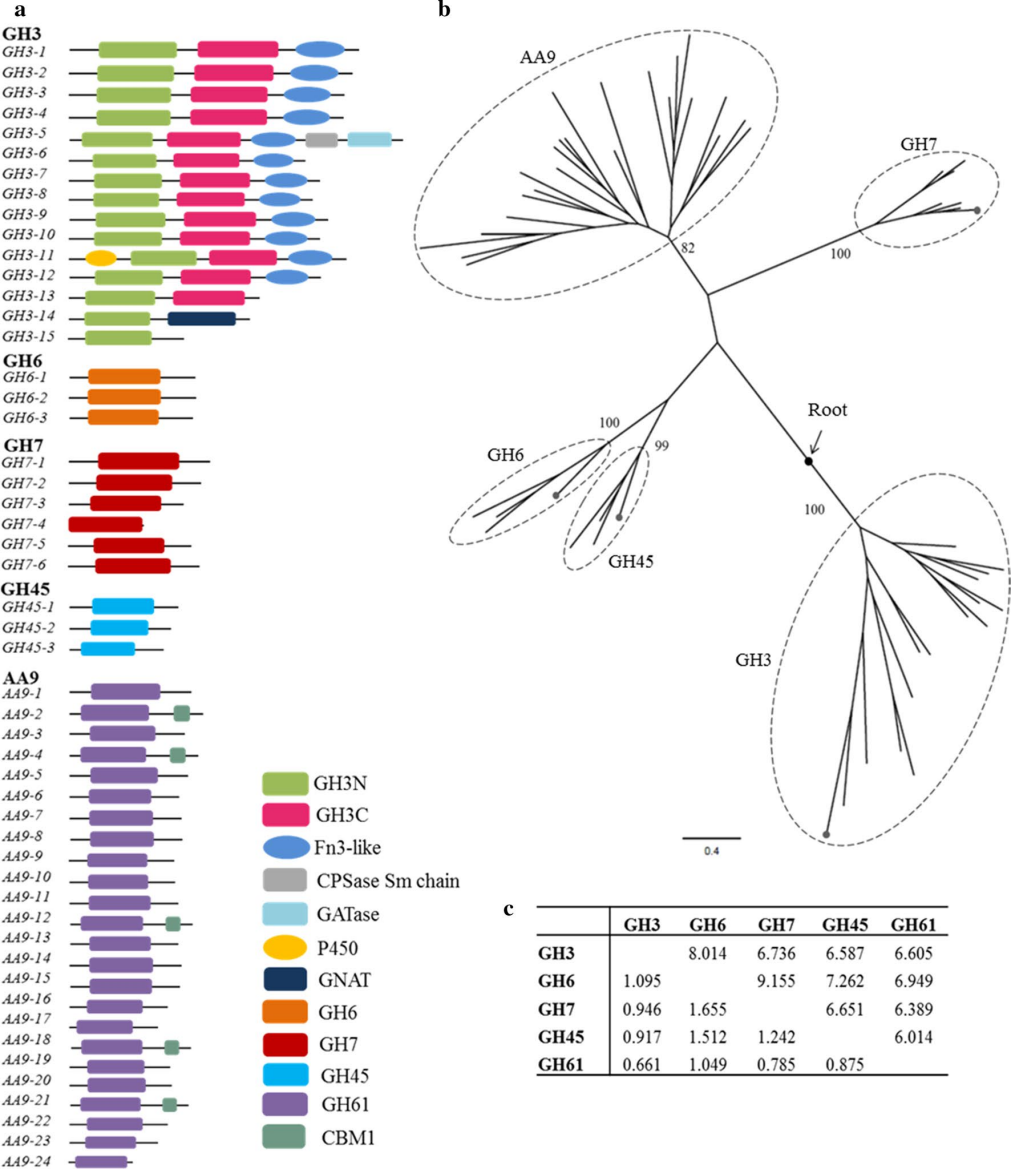
Characterization of GHs in *B. sorokiniana* genome. **a** Domain distributions in the homologs of *B. sorokiniana* GHs and redox enzymes (AA9). The *figure* shows the schematic of arrangement of domains in each of the *BsGH* homologs (comparative lengths are unscaled). **b** Phylogeny and evolution of *B. sorokiniana* GHs. Maximum likelihood rooted phylogeny of *B. sorokiniana* GHs and redox enzymes (AA9). The values on branches show the bootstrap (%). The* dotted* terminal branches in the clades of GH3, GH45, GH6 and GH7 families are the corresponding GHs from the bacterial species used as an out-group in the phylogenetic analysis. **c** Evolutionary divergence between GH family members. The *upper diagonal* represents the number of substitutions per site between the respective GH families, while the *lower diagonal* represents its standard deviation

We performed a detailed genomic characterization of GH families in *B. sorokiniana* genome and targeted five putative cellulases across GH families (GH3, GH6, GH7, GH45 and AA9) in this analysis. These five families comprise the minimum set of cellulolytic enzymes (EGs, CBHs and BGs) that are required for biomass hydrolysis and its identification is a step towards the search for all such cell wall-degrading enzymes in *B. sorokiniana* [46]. Using the HMM profile search and phylogenetic clustering methods, we confirmed the identity of 15, 3, 6, 3 and 23 *B. sorokiniana* homologs of GH3, GH6, GH7, GH45 and AA9, respectively, as initially determined by Ohm et al. [19] (Fig. 1; Additional file 1: Figures S1, S2, Table S1). One additional homolog of the AA9 family was identified in this study (locus ID: jgi|Cocsa1|155289|gm1.10_g) (Fig. 1; Additional file 1: Figures S1, S2, Table S1). The homologs of each GH family varied in the lengths of the gene as well as their transcripts (Additional file 1: Figures S1, S2, Table S1). The members of AA9 are of relatively shorter length, while the GH3 members were larger (Additional file 1: Figures S1, S2, Table S1). Further, we found variable number of introns among the homologous genes in each GH family (Additional file 1: Figure S2, Table S1). Interestingly, some members of GH3, GH6 and AA9 families are intron less (Additional file 1: Figure S2). The sequences of the homologs of each GH family in *B. sorokiniana* have diverged differently (Additional file 1: Figure S3). For example, the members of GH3 family are more diverged with mean identity 29%, while GH7 family members are relatively less diverged with 52% identity. Homologs in each family have the characteristic domain of their respective family (Fig. 1a). GH6, GH7, GH45 and AA9 family members are single domain proteins, while most of the GH3 family members contain two domains (GH3 N-terminal and GH3 C-terminal) with the exception of GH3-14 and GH3-15, which only contains a GH3 N-terminal domain (Fig. 1a) (Additional file 1: Figure S1). Additionally, GH3 family members also have a ‘Fn3-like’ domain at the C-terminal [except in GH3-13 and GH3-15] (Fig. 1a). GH3-5 contains two additional domains at the C-terminus, ‘CPSase_sm_chain’ and ‘GATase’. GH3-13 contains a ‘P450’ domain at the N-terminal end and GH3-14 contains a ‘GNAT’ domain at the C-terminus. Interestingly, only five of the members in AA9 family contain an additional cellulose binding module (CBM1 domain) at the C-terminus (Fig. 1a; Additional file 1: Figure S1).

We used maximum likelihood (ML) methods to determine the phylogenetic clustering among the GH family members in *B. sorokiniana* and obtained five robust clusters each for GH3, GH6, GH7, GH45 and AA9 (Fig. 1b). The GHs from bacteria are clustered within the clade of the respective families to indicate that horizontal gene transfer events might have played an important role in the evolution of GHs in *B. sorokiniana* and other fungi [47]. Variation in branch lengths suggests that after divergence from their common ancestor, the five GH families evolved at varied rates before their further duplication and expansion, resulting in high sequence diversities (Fig. 1b, c). We found maximum evolutionary divergence between GH6 and GH7 families with 9.155 amino acid substitutions per site (Fig. 1c). The large number of poorly aligned regions is also evident from the MSA (Additional file 1: Figure S3).

### Transcriptional profiling of glycoside hydrolases in *B. sorokiniana*

We set out to identify the minimum set of enzymes across endoglucanases, cellobiohydrolases and β-glucosidases in *B. sorokiniana* and succeeded in annotating all of these five GH families. Of these, endoglucanases and cellobiohydrolases are found across GH6, GH7 and GH45 and catalyse the hydrolysis of the β(1,4) cellulose bond to produce cellobiose. The GH3 further catalyses the hydrolysis of cellobiose into glucose. GH61 is the AA9 copper-dependent oxidative enzyme family [20]. Considering the role in driving committed reactions in cellulose degradation, we started by studying the following three families: GH6, GH7 and GH45.

**Fig. 2:**
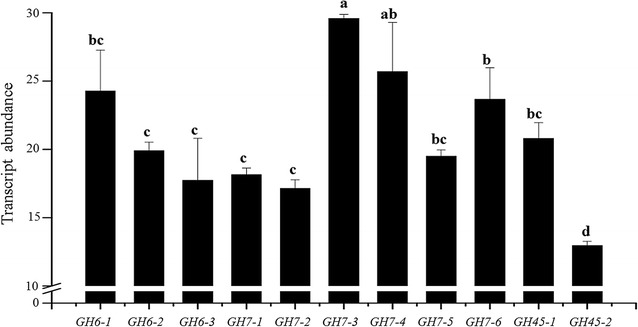
Transcript abundance of the GH family genes in *B. sorokiniana*. A standard curve using known amounts of cDNA was prepared for the three homologs of *BsGH6*, *six* belonging to *BsGH7* and *two* of *BsGH45*; (further details in “Results”) in *four* replicates. Based on the standard curve, cDNA corresponding to 150 ng of total RNA was used to evaluate the absolute transcript amount based on the respective CT values. Three independent experiments were conducted, each comprising *four* replicates, and the mean values were used to plot the graph. Data are represented as mean ± SE. Assistat 7.6 beta was used for statistical analysis (DMRT-Duncan multiple range test). The Duncan test at a level of 5% of probability was applied. *Bars* with the *same letter* do not differ statistically between themselves (*p* ≤ 0.05)

We investigated the abundances of the mRNAs of three homologs of *BsGH6*, six belonging to *BsGH7* and two of *BsGH45*, in the constitutive states. Among the three gene families, GH7 showed higher level of transcript accumulation in three of its homologs, *GH7*-*3*, *GH7*-*4* and *GH7*-*6*, followed by *GH7*-*1*, *GH7*-*2* and *GH7*-*5*. After *GH7*, *GH6*-*1* showed significantly higher accumulation compared to its other two homologs. *GH45*-*2* showed comparatively less accumulation compared to *GH45*-*1* (Fig. 2). Maximum transcript abundance was recorded for *GH7*-*3* and therefore the gene was chosen for biochemical characterization.

### Biochemical characterization of BsGH7-3

**Fig. 3:**
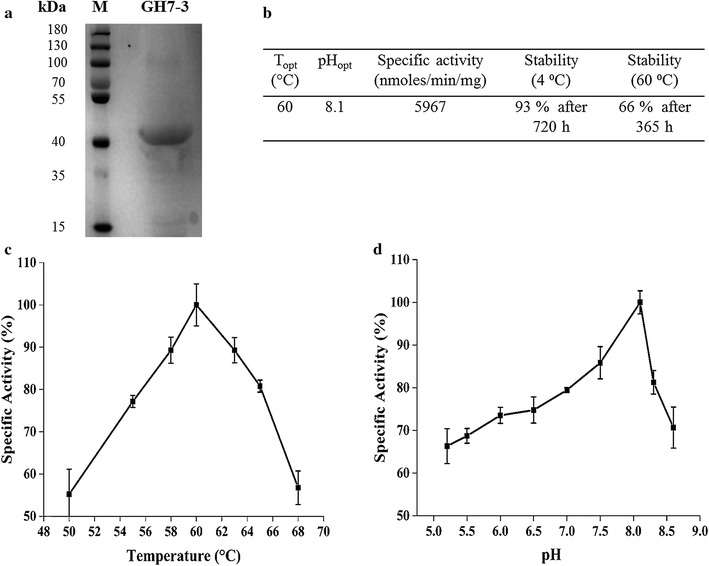
Biochemical characterization of BsGH7-3. **a** 10% SDS-PAGE of purified BsGH7-3. *Lane M* PageRuler plus pre-stained protein ladder (ThermoFisher, Waltham, USA) and BsGH7-3 stained by Coomassie Brilliant Blue R250. **b** Specific activity and stability of BsGH7-3 reported as percent residual activity at 4 and 60 °C, respectively. **c** Effect of temperature on GH7-3 measured over a range of 48 to 68 °C with 0.8% CMC as a substrate and McIlvaine buffer, pH 8.1. The residual activity was measured by the standard DNS assay and reported as % specific activity. **d** Effect of pH was determined by incubating the enzyme in buffer of pH range 5.2–8.6 for 6 h at 4 °C and then the kinetic assay was performed to determine the % specific activity

The open reading frame encoding BsGH7-3 was cloned into a *Pichia* pPICZαC expression vector and verified by sequencing. The sequenced product showed a 100% sequence match to the nucleotide sequence of the predicted GH7-3 in the *Bipolaris* genome (Additional file 1: Figure S4). Protein obtained after ammonium sulphate precipitation and anion exchange chromatography was analysed by SDS-PAGE, and the molecular weight of BsGH7-3 was in agreement with the apparent molecular mass of 46.6 kDa calculated from the sequence (Fig. 3a). The enzyme preparation had a specific activity of 5967 U mg^−1^ (1U = 1 nmol of reducing sugars formed per min per mg of BsGH7-3; Fig. 3b).

BsGH7-3 maintained a broad activity range over pH 5.0–9.0 though the pH optimum (pH_opt_) is 8.1. The enzyme retains 66% of its activity at pH 5.4 and 70% activity at pH 8.6 after overnight incubation at 4 °C (Fig. 3). Temperature optimization studies at pH 8.1 showed that at 60 °C the purified BsGH7-3 had the maximum cellulase activity (Fig. 3c).

BsGH7-3 activity was found to be stimulated by Mn^+2^ and Fe^+2^ in McIlvaine buffer, pH 8.1 (Table 1). Both metal ions together also stimulate the enzyme, resulting in a 512% increase in relative specific activity on CMC. Upon incubation of the enzyme in the presence of Mn^+2^ and Fe^+2^ for 72 h at *T*_opt_ in McIlvaine buffer pH 8.1, only 13% decrease in relative activity is observed. The metal ions could be easily removed by passage through a column packed with Chelex^®^ 100 resin (Sigma-Aldrich, St. Louis, USA), indicating the absence of specific metal binding site(s). In addition, inductively coupled plasma mass spectrometry (ICP-MS) measurements also confirmed the removal of all manganese and ferrous ions (data not shown). When enzyme activity was again measured with the metal-stripped enzyme on CMC, the specific activity decreased to the level prior to metal addition. Endoglucanases are not known to require metals as a cofactor, though there are previous reports of such proteins being stimulated by metal ions [48, 49]. In the presence of 4 M KCl and 4 M NaCl, the specific activity of BsGH7-3 increased by 25 and 10%, respectively (Table 1). Thus, BsGH7-3 is salt tolerant. To determine the enzyme’s robustness and potential use in industrial applications, BsGH7-3 activity was measured in the presence of a few readily available commercial detergents. The enzyme showed maximum stability in the presence of Tide™ (Procter & Gamble, Mumbai, India), with a residual activity of 69% after incubation for 1 h at 60 °C (Table 1). In the presence of Ariel™ (Procter & Gamble, Mumbai, India), SDS (Sigma-Aldrich, St. Louis, USA) and Sunlight™ (Hindustan Unilever, Mumbai, India), the enzyme residual activity was 62, 51 and 74%, respectively (Table 1). Thus, BsGH7-3 is stable in the presence of detergents.

**Table 1:** Effect of metal ions, salts, ionic liquids and detergents on the specific activity of BsGH7-3 and measured by standard spectrophotometric assay

Reagents	Specific activity (%)
10 mM metal ions	
Magnesium chloride	91 ± 7
Copper sulphate	100 ± 1
Manganese chloride	444 ± 3
Ferrous sulphate	304 ± 2
Manganese chloride + ferrous sulphate	512 ± 5
Ferric chloride	179 ± 1
Calcium chloride	113 ± 5
Nickel sulphate	114 ± 4
Zinc acetate	97 ± 2
4000 mM salt	
Potassium chloride	124 ± 3
Sodium chloride	109 ± 2
20% ionic liquid	
[C_2_C_1_im][C_2_C_2_PO_4_]	112 ± 3
[C_2_C_1_im][MeCO_2_]	122 ± 2
[C_2_C_1_im][Cl]	107 ± 3
7 mg mL^−1^ detergents	
Ariel™	62 ± 5
Tide™	69 ± 4
Sunlight™	74 ± 4
SDS	51 ± 3

100% specific activity = GH7-3 CMC activity in the absence of any additives

After incubation at 60 °C for 365 h (15.2 days), BsGH7-3 retained 66% of its specific activity (Fig. 3b). Further, upon incubating the enzyme for 30 days at 4 °C, a residual specific activity of 93% was retained. Ionic liquids (ILs) hold great promise for biomass pretreatment and thus have been the subject of many studies towards understanding its compatibility with enzymes. Since ILs have been generally known to denature cellulase, we desired to test BsGH7-3 stability against three ILs as a further probe of enzyme thermostability [50–52]. In the presence of 20% (v/v) 1-ethyl-3-methyl imidazolium chloride ([C_2_C_1_im][Cl]), 1-ethyl-3-methyl imidazolium phosphate ([C_2_C_1_im][C_2_C_2_PO_4_]) and 1-ethyl-3-methyl imidazolium acetate ([C_2_C_1_im][MeCO_2_]), the enzyme activity is unaffected (Table 1). BsGH7-3 is thus a very stable enzyme with a very long half-life.

**Fig. 4:**
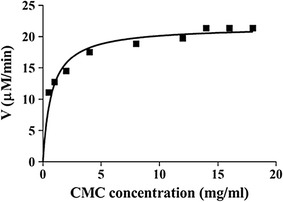
BsGH7-3 catalysed hydrolysis of carboxymethyl cellulose (CMC) as determined by visible spectrophotometry. The *solid line* indicates the best fit of the Michaelis–Menten equation. The values determined were *K*_m_ = 0.7461 mg mL^−1^, *V*_max_ = 21.64 µM min^−1^ and *k*_cat_ = 288 min^−1^

BsGH7-3 showed the highest activity towards lichenan, with the relative activity being 367% as compared to CMC. The relative specific activity towards β-d-glucan is 174% and decreases to 68 and 57% towards PASC and Avicel, respectively (Table 2). The steady-state kinetic parameters of BsGH7-3 were measured under optimal assay conditions (30 min, pH 8.1, 60 °C) by varying the CMC concentration and the data fit using a non-linear regression method (Fig. 4). The enzyme had the *K*_m_, *V*_max_ and *k*_cat_ values of 0.75 mg mL^−1^, 21.64 µM min^−1^ and 288 min^−1^, respectively. BsGH7-3 also decreases the viscosity of lichenan by 11.42% and of β-d-glucan by 9.8%, indicating that BsGH7-3 had a positive effect on viscosity reduction of substrates.

**Table 2:** Relative substrate specificity of recombinant BsGH7-3

0.5% substrate	Main linkage	Specific activity (%)
Lichenan	1,3–1,4-β-(glucose)	367 ± 31
β-d-Glucan	1,3–1,4-β-(glucose)	175 ± 15
CMC-Na	1,4-β-(glucose)	100
PASC	1,4-β-(glucose)	69 ± 4
Avicel	1,4-β-(glucose)	57 ± 9

Specific activity with CMC as a substrate and in the absence of any additives is considered as 100%

### Structural insights into the GH7-3 function

**Fig. 5:**
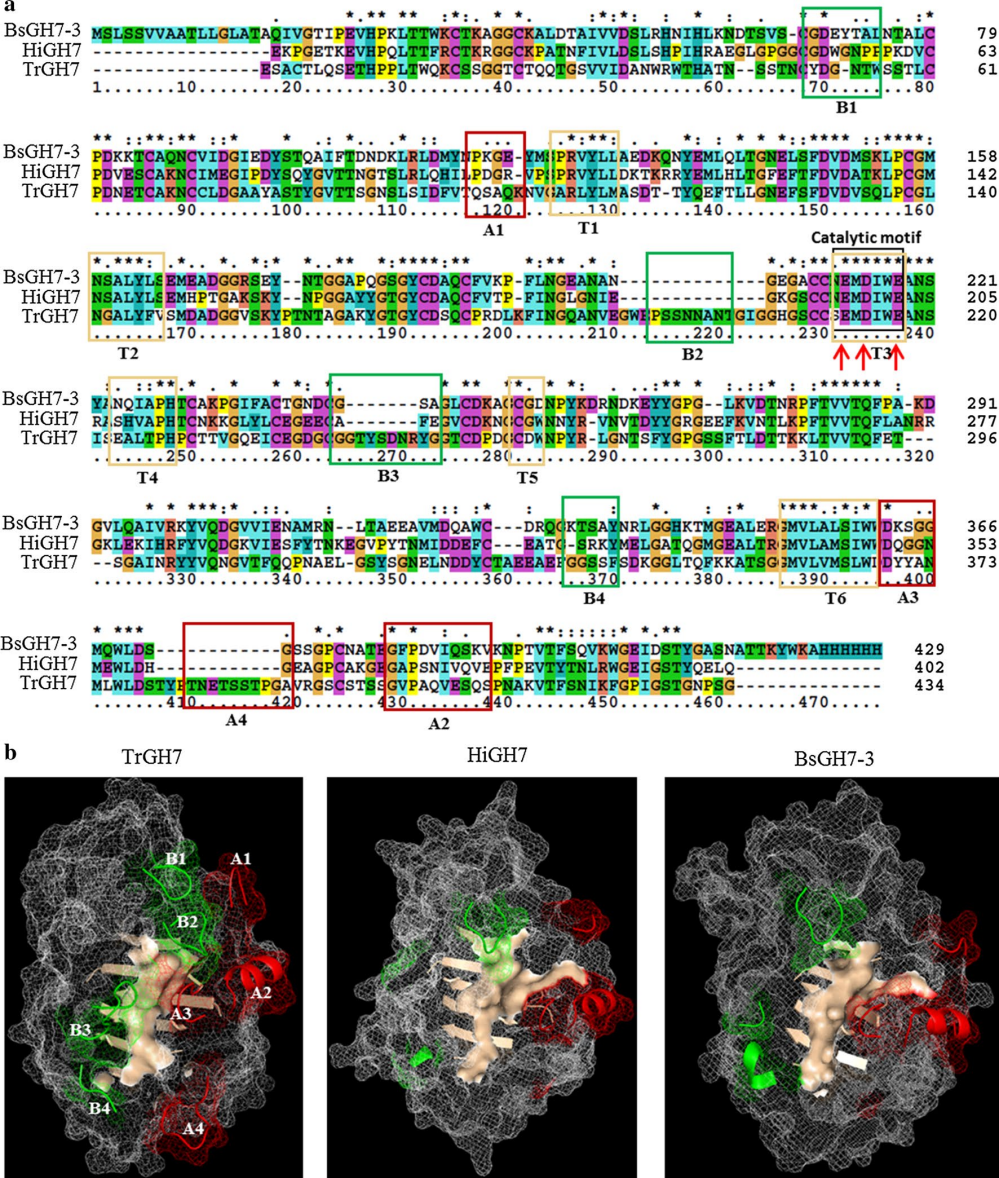
Comparative analysis of *B. sorokiniana* GH7-3 with *H. insolens* endoglucanase GH7 (PDB ID: 1OJJ-B) and *T. reesei* cellobiohydrolase GH7 (PDB ID: 7CEL). (**a**) Multiple sequence alignment of BsGH7-3, HiGH7 and TrGH7. The *black rectangular box* contains a stretch of six residues of the catalytic motif that are conserved across the three proteins. The catalytic residues are marked with *red arrows*. The *coloured rectangular boxes* denote the residues of motifs *B* (*green*), *A* (*red*) and tunnel *T* (*light yellow*). **b** Homology model-based structure of BsGH7-3 shows variation in the secondary structure of motifs *A*, *B* and *T* (*same colour* as in **a**, with HiGH7 and TrGH7). The motifs *A* (*red colour*) and *B* (*green colour*) are in cartoon view on the mesh background. The surface view of the inner lining of the tunnel is shown in *salmon colour*

While the GH7 enzyme family contains both CBH (Cel7A) and EG (Cel7B) enzymes with a similar β-sheet sandwich motif, differences exist. For example, endoglucanases have substrate tunnel-associated peptide loops of shorter lengths compared to cellobiohydrolases. To get an insight into the function of BsGH7-3, we modelled the structure of BsGH7-3. A HMM-based homology search predicted *Humicola insolens* GH7 (HiGH7, PDB ID: 1OJJ) as the best template for BsGH7-3. These two sequences are 53% identical (Fig. 5a; Additional file 1: Figure S5a). *T. reesei* GH7 (PDB ID: 7CEL; TrGH7), which is the most studied cellobiohydrolase (CBH), on the other hand possess a sequence identity of 38% with BsGH7-3 (Fig. 5a; Additional file 1: Figure S5a). Residues in the A loop (A1, A2 and A3) and B loop (B1 and B4) of BsGH7-3 are more identical to HiGH7 than TrGH7 (Fig. 5a). TrGH7 contains three additional but functionally important loops characteristic of CBHs (tunnel exit motif A4, and B2 and B3) that are absent in endoglucanases, including in BsGH7-3 and HiGH7. TrGH7 exhibits variations in all motifs, except T3 containing the catalytic residues (Fig. 5a). Although several GH7 enzymes (6 out of 27 endoglucanases and 27 out of 57 cellobiohydrolases) contain a carbohydrate binding module (CBM), BsGH7-3 does not contain any known CBM domain. Two characteristic Arg residues of CBH, Arg251 and Arg 394 in TrCel7A, are absent in BsGH7-3. Arg251 located at the base of loop B3 in TrCel7A has been implicated in coordination of the reaction product cellobiose but is absent in endoglucanases [53]. Similarly, Arg394 in TrCel7A is a key factor in processive motion of CBHs and is absent in the non-processive endoglucanases [54].

While the overall modelled structure of BsGH7-3 is not significantly different from TrGH7 (RMSD < 3Å; Fig. 5b; Additional file 1: Figure S5a), variations in the size and shape of the substrate binding tunnel were evident. The tunnel in BsGH7-3 appears to resemble a shallow crevice with inner solvent-accessible surface area of 3793.1 Å^2^ compared to the 3879.3 Å^2^ deep tunnel in TrGH7 (Fig. 5b). BsGH7-3 also shows striking differences in the area and volume of the largest binding pocket and the number of residues in the largest electrostatic patch on the protein surface compared to HiGH7 and TrGH7 (Additional file 1: Figure S5b). BsGH7-3 has a smaller binding pocket and the largest electrostatic patch is made up of only five residues (Additional file 1: Figure S5b). Further, the electrostatic potential distribution and its pattern on the surface vary between BsGH7-3, HiGH7 and TrGH7 (Additional file 1: Figure S5c). TrGH7 has relatively more negative patches than HiGH7 and BsGH7-3. Such differences in the electrostatic charge distribution may influence the interaction of protein with salt and ionic liquids.

## Discussion

Plant cell wall polysaccharides are an important source of organic compounds for use as raw material in many industrial processes and serve as a carbon source for different microorganisms including plant pathogens. Pathogens are equipped with a variety of enzymes for degrading polysaccharides. Although genes for many polysaccharide-degrading enzymes have been cloned over the past decade and commercial cocktails manufactured, the cost and efficiency of cellulases remain a challenge. Plant pathogens have evolved to break through the plant cell wall to utilize the plant’s lignocellulose to survive. The wheat pathogen *B. sorokiniana* might thus offer unique cell wall-degrading enzymes towards a more efficient saccharification of wheat straw.

We confirmed and annotated the homologs across five GH families, GH3, GH6, GH7, GH45 and AA9, in *B. sorokiniana* genome. This genome contains different numbers of paralogs ranging from 3 (in GH6 and GH45) to 24 (in GH61) (Fig. 1; Additional file 1: Figure S2, Table S1). Paralogs of the five families show different degrees of identity suggesting that each GH family may have evolved and expanded at a different rate (from 6.014 to 9.155 amino acid substitutions per site) indicating functional variability (Fig. 1c). The study on transcript abundance also suggests variations in the expression of genes within each family and among the families that show up to fourfold differences in expression (Fig. 2). To get further insight into the biochemical mechanism, we selected the BsGH7-3 homolog for further characterization.

GH7 family members are amongst the most important cellulolytic enzymes that are commonly employed in plant cell wall degradation across different eukaryotic kingdoms and play a significant role in biomass hydrolysis. GH7 enzymes typically cleave β-1,4 glycosidic bonds in cellulose/β-1,4-glucans. Endo-1,4-β-glucanase, cellobiohydrolase and endo-1,3-1,4-β-glucanase have been identified in the GH7 family. To elucidate BsGH7-3 function, we characterized the substrate specificity and modelled the structure of BsGH7-3. BsGH7-3 shows higher specific activity towards lichenan (14172.7 U mg^−1^) and β-d-glucan (6739.4 U mg^−1^) and the lowest activity towards Avicel (Avicel being a substrate specific to cellobiohydrolases) following the trends reported for other endoglucanases [45, 49, 55]. The 25% reduction in activity observed on the substrate PASC has also been reported for other GH7 endoglucanases [45]. This non-specific substrate specificity is a characteristic feature of the GH7 endoglucanase. BsGH7-3 effectively decreases substrate viscosity similar to the Cel7A endoglucanase from *Neosartorya fischeri* P1, though this decrease is lower than that of Egl7A EG from *Talaromyces emersonii* CBS394.64 [45, 55]. This reduction in substrate viscosity is also common across endoglucanases. Therefore, we have classified BsGH7-3 as an endoglucanase. The homology-based model of BsGH7-3 further suggests the enzyme to be an endoglucanase. Similar to other endoglucanase (such as HiGH7), it lacks the structural motifs A4, B2 and B3 (Fig. 5). Additionally, BsGH7-3 shows variations in the inner surface area of the binding cavity compared to HiGH7. The inner tunnel area is predicted to be 56.1 Å^2^ smaller along with a smaller binding cavity on its surface than in HiGH7 [56]. This suggests possible differences in catalytic mechanism compared to HiGH7.

BsGH7-3, with a pH_opt_ of 8.1, is an alkaliphilic GH7. The endoglucanase from *Bacillus* sp. MTCC 10048 with a half-life of around 12 h was previously reported to be an alkaliphile [57]. Most other fungal GH7 endoglucanases reported thus far have pH optima between 3.5 and 7.5 [58–60]. The EG1 from *Humicola grisea var. thermoidea* has been reported to show an optimal pH 5.0 though the enzyme was reported to be stable between pH 5.0 and 11.0 at 4 °C for 20 h [60]. The alkaliphilic nature also makes BsGH7-3 compatible to AFEX or lime pretreatment of biomass [61]. With a 65% residual activity after more than 15 days (365 h) at *T*_opt_, the half-life of BsGH7-3 is amongst the highest reported compared to other GH7 endoglucanases, particularly at high pH [58]. Chokhawala et al. reported the expression of an engineered *T. reesei* EGI variant in *T. reesei* (G230A/D113S/D115T Tr_TrEG1) with a half-life of 161 h at 60 °C and pH 4.85 in comparison to the recombinant (*T. reesei* host) wild-type TrEG1 with a half-life of 74 h at 60 °C, pH 4.85 [62]. Another EG from *Trichoderma harzianum* has also been reported to be very stable, with a little change in activity after 2 months of incubation (Additional file 1: Table S3). Here too, the enzyme has a very low turnover number at 0.45 s^−1^ on the substrate xyloglucan [63]. The alkaliphilic endoglucanase from the *Bacillus* sp. MTCC 10048 also shows little activity with a turnover number of 0.55 s^−1^ [57]. Therefore, the comparatively high kinetic efficiency with a *k*_cat_ of 4.8 s^−1^ and high stability makes BsGH7-3 a very promising alkaliphilic endoglucanase.

The stimulatory effect of BsGH7-3 observed in the presence of divalent metal ions, Mn^2+^ and Fe^2+^, is intriguing. Metal binding studies indicate that the enzyme is not a metalloenzyme since the 3- to 5-fold increase observed in the presence of metals is reversed upon metal removal. While the stimulation by metal ions has been previously reported across cellulases, and in particular stimulation of endoglucanase activity, no mechanisms have been proposed [64–68]. The activity increase is probably due to better folding of the protein and possible metal-induced multimerization effects that enhance protein stability. Further experiments are required to understand the role of metal ions in the stability of this enzyme. The enzyme was also stable in the presence of four commercial detergents tested, with residual activity in the range of 51–74%. Although ILs are also known to denature enzymes, there are some reports of endoglucanases which are stable towards ILs. The endoglucanases from *Stachybotrys microspora* are 50% active in the presence of 20% (v/v) 1-butyl-3-methylimidazolium chloride  [59]. Gladden et al. reported an endoglucanase from GH12 with the highest activity in the presence of 15% (v/v)

1-ethyl-3-methylimidazolium acetate ([C2mim][OAc]) and another from GH5 with the highest activity in 25% [C2mim][OAc] [69]. BsGH7-3 does not show any loss in activity in the presence of 20% (v/v) of the three ILs tested, indicating high stability and compatibility to IL pretreatment.

BsGH7-3 tolerates salt and also shows up to 1.25-fold increase in activity in the presence of salt. Sequence analysis shows that the acidic residues account for 12% of the total residues and the pI of the protein as determined by ProtParam is 4.96 [70]. Acidic amino acid residues help create a salt hydration shell to resist the denaturing environment created by high salt concentration and confer stability to the protein [71–73].

## Conclusions

Here we report the annotation and characterization of cellulase genes in *B. sorokiniana* and derive phylogenetic inferences. Based on expression profiling of the cellulase genes, the third homolog of GH7 was characterized to be an endoglucanase from the GH7 family. The enzyme is highly thermostable, salt tolerant and of higher kinetic competence than most similarly thermostable fungal GH7 EGs. Several other cellulase genes of the pathogen have also been shortlisted based on expression levels, and their characterization is ongoing in the laboratory. We hope that this methodology of searching and screening will further enhance the repertoire of promising enzymes, particularly in plant pathogens, and help us find novel enzymes in the degradation of specific plant biomass.

## Additional file

**Additional file 1.** Supplemental material to “Genome-wide characterization of cellulases from the hemi-biotrophic plant pathogen, *Bipolaris sorokiniana*, reveals presence of a highly stable GH7 endoglucanase”. **Figure S1.** Transcript sequences of *B. sorokiniana GHs* (*GH3, GH6, GH7* and *GH45*) and *AA9* genes. **Figure S2.** Genomic architecture of *B. sorokiniana* GHs and redox enzymes (AA9). The figure shows the schematic of arrangement of introns and exons in each of the *BsGH* homologs (comparative lengths are unscaled). **Figure S3.** Multiple sequence alignment of protein sequences of *B. sorokiniana* GHs and AA9. **Figure S4.** Complete CDS sequence of *B. sorokiniana* GH7-3 as obtained after sequencing. **Figure S5.** (a) The upper and lower diagonal in matrix represents the % of identities between the sequences and RMSD (Å) between the structures of TrGH7 (7CEL-A), HiGH7 (1OJJ-B) and BsGH7-3 respectively. (b) The structural diversities in the binding. regions among TrGH7, HiGH7 and BsGH7-3. (c) Comparative electrostatic potential distribution between TrGH7, HiGH7 and BsGH7-3. **Table S1.** The genomic features of *B. sorokiniana GHs* and *AA9* genes. Details of length of exons and ORF coordinates of *B. sorokiniana GHs* and *AA9* transcripts. **Table S2.** (a) Details of primers used for qPCR analysis of *B. sorokiniana GHs* transcripts (b) Details of primers used for cloning of *BsGH7-3* transcripts. **Table S3.** Comparison of BsGH7-3 with other fungal endoglucanases of the GH7 family with CMC as the substrate.

## Abbreviations

EG: endoglucanase
CBH: cellobiohydrolase
CMC: carboxymethyl cellulose
Bs: *Bipolaris sorokiniana*
[C_2_mim]: 1-ethyl-3-methylimidazolium
[C_2_C_1_im][Cl]: 1-ethyl-3-methylimidazolium chloride
[C_2_C_1_im][MeCO_2_]: 1-ethyl-3-methylimidazolium acetate
[C_2_C_1_im][C_2_C_2_PO_4_]: 1-ethyl-3-methylimidazolium phosphate
AA: auxiliary activity
GH: glycoside hydrolase family 7
IL: ionic liquid
PASC: phosphoric acid swollen cellulose
ICP-MS: inductively coupled plasma mass spectrometry
t_1/2_: half-life
