# Supplementary material for: Genome-wide characterization of cellulases from the hemi-biotrophic plant pathogen, Bipolaris sorokiniana, reveals the presence of a highly stable GH7 endoglucanase
Source: Biotechnol Biofuels. 2017 May 25;10:135. doi: 10.1186/s13068-017-0822-0 (PMC5445349; doi:10.1186/s13068-017-0822-0)
Supplement: Supplementary file 1 — Additional file 1. Supplemental material to “Genome-wide characterization of cellulases from the hemi-biotrophic plant pathogen, Bipolaris sorokiniana, reveals presence of a highly stable GH7 endoglucanase”. Figure S1. Transcript sequences of B. sorokiniana GHs (GH3, GH6, GH7 and GH45) and AA9 genes. Figure S2. Genomic architecture of B. sorokiniana GHs and redox enzymes (AA9). The figure shows the schematic of arrangement of introns and exons in each of the BsGH homologs (comparative lengths are unscaled). Figure S3. Multiple sequence alignment of protein sequences of B. sorokiniana GHs and AA9. Figure S4. Complete CDS sequence of B. sorokiniana GH7-3 as obtained after sequencing. Figure S5. (a) The upper and lower diagonal in matrix represents the % of identities between the sequences and RMSD (Å) between the structures of TrGH7 (7CEL-A), HiGH7 (1OJJ-B) and BsGH7-3 respectively. (b) The structural diversities in the binding. regions among TrGH7, HiGH7 and BsGH7-3. (c) Comparative electrostatic potential distribution between TrGH7, HiGH7 and BsGH7-3. Table S1. The genomic features of B. sorokiniana GHs and AA9 genes. Details of length of exons and ORF coordinates of B. sorokiniana GHs and AA9 transcripts. Table S2. (a) Details of primers used for qPCR analysis of B. sorokiniana GHs transcripts (b) Details of primers used for cloning of BsGH7-3 transcripts. Table S3. Comparison of BsGH7-3 with other fungal endoglucanases of the GH7 family with CMC as the substrate. [file 13068_2017_822_MOESM1_ESM.pdf]

Supplemental material

Genome-wide characterization of cellulases from the hemi-biotrophic plant pathogen, *Bipolaris sorokiniana*, reveals presence of a highly stable GH7 endoglucanase

Shritama Aich[&,b], Ravi K. Singh[&,a], Pritha Kundu[a], Shree P. Pandey[a,*], Supratim Datta[b, c,*]

[a]Department of Biological Sciences, Indian Institute of Science Education and Research Kolkata, Mohanpur, India; [b]Protein Engineering Laboratory, Department of Biological Sciences, Indian Institute of Science Education and Research Kolkata, Mohanpur, India; [c]Centre for Advanced Functional Materials, Indian Institute of Science Education and Research Kolkata, Mohanpur, India

[*] Corresponding authors: supratim@iiserkol.ac.in, sppandey@iiserkol.ac.in

Supplementary figure S1. Transcript sequences of *B. sorokiniana GHs* (*GH3, GH6, GH7* and *GH45*) and *AA9* genes.

GH3 genes

>*GH3-1*(jgi|Cocsa1|208057|CE3403_8724)

```
   1 CTCCACACTC ATTGTCCTAC CAGATATCGT CTCCGCTCGC TCACATCTGC ATCGGCGGCA TCACTGTACC CAACTGCTGC TGCAAACATA TACACGCTTC
 101 AATGCTAGCT TCAATATAAA CAGGGCTTCA CGCTCGCGCC GTTTGCCATA GCCTCACCTT CCCCGCCGCG TGCAACACCA CATATTCCTT TTTCCTGCTG
 201 CTCCTCTTGG ATTGACACGC TTCTATCAAC AAAATGGCTC CCTACGAAGC CCACGAGTTG CGTCGCGGCA AGTACGCGAA GCTCGATGCA CCCGTGGACG
 301 ATGACCTTGA AACCCCAACG CGATTCTCCT CCGAGTCCAA CGAGACCCTG GAAGATCTCG AAGAACACGA CCCCCTCGCA TTCGATAGCA AGTTGAAACG
 401 AAAGAAGAGT GCCGGCGACC TCAAGGTCCG GAAAGCATCG TACCGAGACA CCGAGGCCGT GACGCCATTC CCTACCCAAC AACGAAGATC CAAATGCGCA
 501 CCAGGCCGCT GTTGCTGCTG GCTGCTCGTA TTTATCGCCG CTACTGTTTT GCTACTGGCT TCGGGAGGAA TTTGGGCATA CAAGGTGTCG GTGCCTGAGG
 601 ATGGTCGCAG TCCGCCGTGG TACCCCTCGC CAAAGGGCGG AACAGACAAG GCATGGGAGG AGAGCTACAA GAAGGCTGCT GAACTGGTCA AGCAGATGAC
 701 ACTACCTGAG AAGGTTAACA TTACCACCGG CACAGGGTGG AGCATGGATC TTTGTGTGGG CCAGACTGGC CGTGTGGATA GGTTAAAGTT CCCCAGTCTA
 801 TGTCTTCAGG ACGGTCCTCT GGGAATTCGA TTTGCTGACA ACATTACCGG CTTCCCTGCA GGCATCACCA CTGGCGCGAC ATGGAGTAAG GAGCTCTTCT
 901 ACCTGCGCGG AAAAGCGCAT GGCAAGGAGG CACGACTGAA AGGTGTCAAC GTTCTACTTG GTCCTGCAAT GGGCCCTCTC GGCCGAATGC CTGCTGGCGG
1001 TCGTGTTTGG GAAGGATTCG GCTCAGACCC CGTGCTCCAA GGCATTGCAG CGGCCGCGAC TATCAAAGGC ATCCAAGACG AGGGCGTCAT GGCTACCGCA
1101 AAACACTATA TTGCCAACGA ACAGGAACAC TTCAGACAGG CGTGGGAGTG GGGCCTGCCG AATGCCATCT CCAGCAACAT CGACGACCGC ACCTTACATG
1201 AAATCTATGC ATGGCCATTC GCCGACTCGG TCAAAGCAGG TGTAACATCT ATCATGTGTT CATACAACCA GGTAAACTCC AGCTATGCCT GCCAGAATTC
1301 GAAGCTGCTC AACGGAATTT TGAAAGACGA ACTTGGTTTC CAGGGGTTCG TTCAGAGCGA CTGGTTAGCG CAACGTTCGG GTGTGGCGAG CGCCCTGGCG
1401 GGTTTGGACA TGACCATGCC TGGAGATGGC CTCAGATGGG CCAAGGGTAA CAGTCTATGG GGTTCAGAGC TCACCAAATC AGTTCTCAAC GGCAGTGTAC
1501 CCATTGATCG CGTCGATGAT ATGGTTACTC GTGTCGTAGC GTCCTGGTAC CAAGTCGGTC AGGACAAGTG GGAAAATGAT GGGCCAAACT TTTCTTCCTG
1601 GACCAACGAC CGTATCGGTA AACTACATGA AGGCAGCCCC TCGAATCACG AAATGGGTGT GGTCAACCAG TACGTCAACG CTCAAGGAGA GGGAAAAGAA
1701 GCTCATGGAC ACCTGGTGCG TCGCGTTGCA GCTGAAGGTA CAGTCTTGGT CAAGAATGAT GGCAACATGC TGCCACTAGA CCGCAAAGGT TGGAAGACCG
1801 ACGGAAAGGC CAAGGCAAAG TTCCGTGTCG GCGTTTTTGG TGAAGATGCA CGTCTCCCCC ACGACGGCAT CAACCACTGC CCTGATCAAA GCTGCAACGA
1901 GGGGACCTTG GCTTCAGGTT GGGGTTCCGG TGCCGTCAAC TTTTCGTATC TGGTCGAGCC CGTATCTGCC ATCAGACAGG CGTTTGACAA TGAGACCGTA
2001 TACGTAACAG ACTGGTTGGA GAACAAGCTC CCCAACCAAA AGGAGATTCT CGAAGATCAA GACCTTTGCA TCGTTTTCGC CAACGCTGAT TCAGGAGAAG
2101 GATATGAGCA CTACGGAAAC ATCCGAGGCG ACAGAAATGA TCTCAATCTG CAAAAAGGCG GTGCCGAGCT CATCAGAAAT GTTGCCAAAG ACTGTGGAGA
2201 AGGTATGGGC GACGTCGTTG TCGTCATCCA CACGGTTGGA TCTGTCATCC TAGAGGATTT CATCGACATC ATGAACGTCA GGGCAGTACT CATTGCCCAC
2301 CTTCCAGGTA CGGAGTCTGG AAATGCCATT GTGGATGTAC TGTTTGGCGA CGTCAACCCA TCTGGTCGTC TACCCTACAC CATTGCTAAG AAGGAAGACG
2401 ACTTTGGTCC AGGCAGCAAG GTCAAGTATC TTCCAAGTCC ATCCGACGGA CTTGCACCTC AGCAAGATTT CACCGAAGGC CTATATATCG ACTACCGCTA
2501 TTTCGACAAG CAGAACATCA CTCCCCGATT CGAGTTTGGC TACGGTCTCT CATATACACA ATTCGAGCTA TCTTCCCTCA TGATCGAAGA AAAGAGCGCA
2601 AAAACACCTC TACCCAAGCC TCGACCAGAT GGCCTCACAC CCCCAACCTA CTCCACCGTC CTTCCAGACC CAGAGTCGGT ACTCTTCCCT GAAGGCTTTA
2701 ACAAAGTAGA ACAATACATC TACCCCTGGC TCGACTCAAC CAAAGGCATC AAGAAATCCG GACCCCCACA GCCTCAACCT CCATCCTCCC CACTTAGCGA
2801 CGCAGGCGGC GGCCCAGGCG GCAACCCGGA TCTCTACACC ATCCTCCTCA CTGTCTCCGC CTCTCTCGAA AACAAAGGCC CCCTAGACGG CGCTTCCGTC
2901 GTCCAACTGT ATATTTCCCT CCCCCAAGAC TACAAGGATC ACGAGACGGG CGAGCCGGTG GATTTCCCTG TTCGTGTCTT GCGTGGGTTT GAGAAGGTGC
3001 AGCTTGAGAA GGGGAAGGGA AAGCTGGTTC AGTTTGAGGT TACGAGACGC GATTTGAGTT ATTGGGATGT TAAGAGGCAG AATTGGGTAT TGCCCATGGG
3101 AGAGATTGGG ATCCAGGTTG GGTTTTCGAG TCGGGATTTG CCATTGAAGG GGAAGTGGTG AGGTGCTCAT TGTTATTCTT GTTTTTCTGT TTAGATGGTT
3201 GGGGTGGATA GGTTAGGGAA GGAGTATGAG GTATAGGTTT TGGCGTTTTC TTTTTCTGTG CCCTTTTCTC TTTGATACTT TTTCTTTTCT TTTGTG
```

>*GH3-2*(jgi|Cocsa1|40129|fgenesh1_kg.14_#_97_#_Locus1433v1rpkm74.10)

```
   1 CACTGTGTCT GCGACTGCAT TCAAGTCCTC GTTGCAGATT GCCTCACAAG TAGCGTCTGT TGCTGTGTTG CTCGTGTCTC TGCTTCTGCG TTCCCTAGTC
 101 ACCCAGACGC CATCATGCTG TGGCTTGCAC AAGCATTGTT GGTCGGCCTT GCCCAGGCAT CGCCCAGGTT CCCTCGTGCT ACCAACGACA CTGGCAGTGG
 201 TTCTTTGAAC AATGCCCAGA GCCCGCCATT CTACCCAAGT CCTTGGGTAG ACCCTACCAC CAAGGACTGG GCAGCTGCCT ATGAAAAGGC AAAGGCTTTT
 301 GTTAGCCAAT TGACTCTGAT CGAGAAGGTC AACCTCACCA CCGGCACTGG ATGGCAGAGC GACCACTGTG TTGGTAACGT GGGCGCTATT CCTCGCCTTG
 401 ACTTTGATCC CCTCTGCCTC CAGGACAGCC CTCTCGGCAT CCGTTTTGCG GACTACGTCT CTGCTTTCCC AGCAGGTGGC ACCGTTGCTG CATCATGGGA
 501 CCGTTATGAG TTTTACACCC GCGGTAACGA GATGGGCAAG GAGCACCGTA AGAAGGGTGT CGACATTCAG CTTGGTCCTG CCATTGGACC TCTTGGTCGC
 601 CACCCCAAGG GCGGTCGTAA CTGGGAAGGC TTCAGTCCTG ATCCTGTGCT CTCGGGTATT GCCGTGGCCG AAACGGTCCG CGGTATCCAG GATGCTGGTG
 701 TCATCGCCTG CACCAAGCAC TACCTTCTGA ATGAGCAAGA GCATTTCCGT CAGCCCGGCG GTTTCAAGGA CATCCCCTTT GTCGATGCCA TCAGCTCCAA
 801 TACCGATGAC AAGACTCTAC ACGAGCTCTA CCTGTGGCCC TTTGCCGACG CCGTCCGCGC TGGTACTGGT TCCATCATGT GCTCTTACAA CAAGGCCAAC
 901 AACTCGCAAG TCTGCCAAAA TTCGTACCTC CAAAACTATA TTCTCAAGGG CGAGCTTGGC TTCCAGGGTT TCATTCTATC TGACTGGGAT GCACAGCACT
1001 CGGGTGTTGC CTCGGCTTAT GCCGGATTGG ACATGACCAT GCCTGGTGAC ACTGGATTCA ACTCTGGACT CTCTTTCTGG GGAACTAACC TGACCGTCTC
1101 AATTCTTAAC GGCACCATTC CTCAGTGGCG TCTCGACGAT GCGGCTATCC GTATCATGGC CGCATACTAC TTTGTTGGCC TTGACGAGTC TATCCCCGTC
1201 AACTTTGACA GCTGGCAAAC TAGCACATAC GGATTCGAGC ACTTTTTCGG AAAGAAGAGC TTCGGTCTGG TCAACAAGCA CGTTGATGTT CGCGAGGAGC
1301 ACTTCCGCTC TATTCGCCGC TCTGCTGCCA AGTCGACTGT TCTCCTCAAG AACTCTGGCG TCCTTCCTCT CTCTGGAAAA GAGAAGTGGA CTGCTGTATT
1401 TGGAGAAGAT GCTGGCGAAA ACCCATTGGG TCCCAACGGA TGCGCTGACC GTGGCTGCAA CTCTGGCACC TTGGCCATGG GCTGGGGTTC GGGAACTGCA
1501 GACTTCCCTT ACCTCATCAC TCCTCTCGAA GCCATCAAAC GCGAGGTGAA CGAGAACGGC GGCATGATCA CTTCAGTCAC CGACAACTAC GCTACTTCGC
1601 AGATCCAGAA CATGGCCAGC AGGGCCAGCC ACTCGATTGT TTTCGTCAAC GCCGACTCTG GTGAAGGTTA CATCACCGTT GATAACAACA TGGGTGACCG
1701 CAACAACCTG ACTGTGTGGG GCAATGGTGA TGTGCTCGTC AAGAACGTCT CTGCTCTGTG CAACAACACC ATTGTAGTTA TCCACTCTGT CGGCCCAGTC
1801 ATTGTCGATG CCTGGAAGGA GAACGCCAAC GTGACTGCCA TTCTCTGGGC TGGTCTTCCT GGCCAGGAGT CTGGTAACTC GATTGCCGAT ATTCTGTACG
1901 GACACCACAA CCCTGGTGGC AAGCTCCCCT TCACCATTGG CAGCTCTGCA GAGGAATATG GTCCTGATAT CATTTATGAG CCCACGAACG GCATCCTCAG
2001 CCCCCAGGCC AATTTTGAAG AGGGCGTCTT CATTGACTAC CGCGCGTTTG ACAAGGCGGG CATTGAGCCC ACGTACGAGT TTGGCTTTGG TCTTTCGTAT
2101 ACGACTTTTG AATACTCAGA CATCAAGGTC ACTGCGCAGT CTGCTAAGGC TTACAAACCC TTCACCGGCC AGACTTCGGC TGCCCCTACA TTCGGAAACT
2201 TCAGCAAGAA CCCCGAGGAC TACCAGTACC CTCCCGGCCT TGTTTACCCC GACACGTTCA TCTACCCCTA CCTCAACTCA ACTGACCTCA AGACGGCGTC
2301 TCAGGATCCC GAGTACGGCC TCAACGTTAC CTGGCCCAAG GGCTCTACCG ATGGTTCGCC TCAGGCCCGC ATTGCGGCTG GTGGTGCGCC CGGTGGTAAC
2401 CCCCAGCTCT GGGACGTTCT GTTCAAGGTC GAGGCCACGA TCACCAACAC TGGTGATGTT GCTGGTGACG AGGTGGCCCA GGCGTACATC TCGCTTGGTG
2501 GCCCCCACGA CCCCAAGGTG CAGCTCCGTG ACTTTGACCG CTTGACCATC CAGCCCGGCG AGAGCGTCGT TTTCACTGCC AACATCACCC GCCGTGATGT
2601 CAGCAATTGG GACACTGTCA GCCAGAACTG GGTCATTACC GAGTACCCCA AGACGATCCA CGTTGGTGCT AGTTCGAGGA ATCTTCCTCT TTCTGCCCCA
2701 CTGGACACTA GCAGCTTTAA ATAGATGGGC TTGCCATAGT GGGAAAGGAA CAAGGAATTT TTTTACAGCC AATAAGTTGC TGTTGTAATT CGTTAATCTT
2801 GATTAGCTTA GATATATACA CATCTAGCTG TTATGAACAA GTTATTTTTA TCCGAACAAA GTCAATTGCC GCATCTGGTG
```

>*GH3-3*(jgi|Cocsa1|305206|CE100552_873)

```
   1 AGCGCGATGG GCTTTCTGTT TTGGGGAATA ATGGTAGGGG GGTGGTGGTC ACACAGGTAA TGGTGGGTTT TTGCGGGGTT TGCGTCATGG GTAAGGGGAA
 101 AGAGAGAGGG AGAGAGGGAG GACTATGTAT ATGGGTGGCT TTCGCACCCT TGGTTTGAAT TGTTTTGGAG GGATTCCAGT GAACAATTTG AGTTGAAGGG
 201 ATAAGTACCT TCTTTTTGTC ACCACCAGAC GTCGGTCTTG GAATCATGGC TTCCCTTCTC TTCTTGTCTC TCTCCGGACT CGCGGCTGGC GCTGCTATCC
 301 AGCAGCGTGA CCCTGTGCCT GCAGGTTATG TTGCGCCGCC ATACTATCCT GCTCCCCATG GAGGATGGGT GTCGGACTGG GCCGAGAGCT ACCGAAAGGC
 401 TTCTCTCCTC GTTTCCAATA TGACGCTTGC TGAGAAGACT AATCTCACAG CTGGAACGGG AATCTTCATG GGCCGCTGTG TGGGAAATAC TGGTAGTGCT
 501 TTGAGAGTAG GCATTCCGCA GCTTTGCTTG CAAGATGGTC CGTTGGGTGT GAGAAACACC GACCACAACA CTGCTTTCCC GGCAGGCATC ACCGCTGGAG
 601 CAACGTGGGA CAAGGATCTC ATTTACCGAC GTGCAGTCGC TATTGGAGAG GAGTTCCGCG GAAAGGGAGT CAACATCCAT TTGGGACCAA GTGTAGGACC
 701 TCTTGGACGC AAGCCGCGAG GTGGCAGAAA TTGGGAGGGT TTTGGCTCCG ACCCGGTTCT CCAGGCATTT GGAGGTGCTC TGTCTGTAGA AGGTATTCAG
 801 AGCACTGGCG TCATTGCTAC CATCAAGCAT TTGATTGCGA ACGAGCAGGA AATGTACCGT ATGTGGAATC TTGTCAAGCC AGGCATCTCG TCAAATGTCG
 901 ATGACCGCAC GCTTCACGAG CTCTACCTTT GGCCATTCGC AGAAGGCGTA CGTTCCGGCG TCGGCTCAGT CATGATTGCT TATAATGCTG TCAACGGAAG
1001 CGCATGCGCA CAAAACAGCT ACATGACCAA CGGTCTCCTG AAGGATGAGC TCGGCTTCCA GGGTTTCGTC ATGTCTGACT GGCTATCCCA AATCTCCGGT
1101 GTCAGCTCAA CCCTCGCTGG TCTGGACATG TCCATGCCTG GCGATCGAAA TGACATCCCT CTTGTCCTCG GAAACTCCTA CTGGATGTAC GAACAGACCC
1201 GTTCGGTGCT GAACGGCTCT GTCCCAGTGG ACCGCGTCAA CGATGCCGTG ACACGCATCT TGGCCACATA CTTCCAGATG GGCCAGGACC AAAACTACCC
1301 AAGGCCCAAC TTTGACACCA ACACTCAGAA TGCCGAGGGC CCGCTCTACC CTGGTGCACT CATCTCGCCT TCAGGTGTAG TAAACGAGTT CGTGAATGTC
1401 CAGGGCAATC ACGCCGAGGT TGCTCGTGAG GTTGCTCGAG ATGCCATTAC CTTGTTGAAG AACCAAGACA ACATTCTTCC TCTGGCCGCG AGTGCCCCGC
1501 TCAAGATCTT CGGAACAGAC GCCGAGAAGA ACCCAGACGG CATCAACTCG TGCGCTGACC AGGGTTGCAA CAAAGGTACC TTGGGAATGG GCTGGGGAAG
1601 TGGCAGCGCT CGCTACCCAT ACATGGACTC GCCCATTGAT GGCTTCAAGG CTCGCGGTGC AAACTACCAA TTCTTCAACA CGGATAACTT CCCGGGTAAC
1701 TCGAACCCGT CTCCAAACGA CACTGCCGTT GTCTTTGTAA CTGCCGACTC GGGAGAAAAC TACATCACGG TTGAGGGCAA CCCAGGAGAC CGAACTTCTG
1801 CAAACCTGAA CCTGTGGCAC AATGGTGACA AACTGATCAA GGATGTTGCT GCCAAGTACT CGAATGTCGT CGTTGTCGTG CACACTGTTG GAGTTATCCT
1901 TATGAACGAA TGGCATGACC TTCCTTCTGT CAAGGCAATC GTATTCGCTC ATTTGCCCGG ACAAGAAGCA GGAAACTCTC TGATGCAGGT GCTGTACGGC
2001 GATGTCTCTC CATCTGGTCA CCTGCCTTAC ACCCTGCCCA ATGCTGAGGA CGACTTTGGC AACTCTGTGA AGCTTGTTGG ATACCAACTG GGTCAGCCTC
2101 AAGACACGTT TACAGAGGGC CTGTACATTG ACTACCGTCA CTTCCACAAG GCCAACATCA CACCCCGCTA TGCCTTTGGC CACGGACTCA GCTACACGAC
2201 CTTCTCCTTT TCCGACGCCA CAATCACCCC TGTGACGCCA CTCACCAAAA CGCCACCAGC ACGGCCAGCA AAGGGCCAAA CACCGTCGTA CTCGACCGCG
2301 ATTCCACCTG CTTCTGAAGC ATACTGGCCT GCAAACTTCA ACCGCATCTG GCGCTACTTG TATTCGTGGC TTGAAAAGAA TGATGCCGAC GCTGCTGCGC
2401 AGGTGGGAGC TTCTCCTTCG ACATACCCCT ATCCTGCGGG ATATTCCAAC GAGCAAAAGC CCGGGCCTGC AGCAGGTGGC GCACAAGGTG GCAACCCCGC
2501 GCTCTTTGAT GTGGCATACG ACGTTTCTGT AACGGTAACC AACACTGGCA GCCGTAGTGG CAAGGCTGTC GCACAGCTCT ACTTGCAGTT CCCCAACGAG
2601 AGCACGGTCG ACACGCCCAT CCTCCAGCTG CGAGACTTTG CAAAGACGTC TACGCTTGCG GCTGGAGCTA CCCAGACGTT GAAGATGCGA CTGACAAGGA
```

```
2701 AGGACCTTAG CGTGTGGGAT GTCGTTGAGC AGAATTGGGT TGTCCCTGCT GTGGGTGGTG ACTATGGGGT TTGGATTGGT GGGTCTTCGG ATGACTTGCA
2801 TCTGAGGTGC GGTACGGCTA CGGGACGTTG CCAGGGAGAT CAGACGAGCC CTGTTTGAGG GGTTCTCTTT GCTCTAACGA CATGAGCGAG GCCTTTCCTA
2901 TAACGAATGA CTTTAT
```

>*GH3-4*(jgi|Cocsa1|36266|fgenesh1_kg.6_#_189_#_Locus2312v1rpkm46.93)

```
   1 GGGCGACTTT GGTCCGGGGG CGAAGGAGCA ATGGTGCTAT ATAAGTGCCT CAGTGCAACT GCAATCATGG GTATTCAGGC TTTCGATTCA CCTTCGACTA
 101 TCTGGAATCA TGGCGCCCTC TGTCGTATCT AAGGTGCTCT CGGGCTCTGC CCTCTTGGCT GCAGTCAGTG CTCAGAACTT CGGAGGTGGT GGTCGCGGTG
 201 AAGATGCGTT CAGTTACGTC CAGCCGCTCA ACACTACCAT CCTGACCGAG TATGGCTCCT CGCCGGCTGT ATATCCTTCA CCGAAAATTT CTGGTGCTGG
 301 AGGCTGGGAG ATGGGTCTTG AGAAGGCAAA AGCTTTCGTG GCTCAACTCA CTCTAGAAGA AAAGGCTGAC ATGGTCACTG GTCAAGCCGG CCCTTGCGTT
 401 GGCAACATCG TTGCCATTCC CCGCCTGGGC TTCCCTGGTC TTTGCCTTCA CGATGGCCCT CTTGCCATTC GCGTGGCTGA TTACGCAAGC GTCTTCTCCG
 501 CCGGTGTATC CGCTGGTGCT TCATGGGACA AGGAACTCAT GTATGAGCGC GGCTACGCCA TGGGCGAAGA GTTCAGGGCA AAGGGTGCTC ACGTCTTCTT
 601 GGGTCCTGTC GCAGGACCAC TTGGACGTTC CGCCTATGCC GGCCGTAACT GGGAGGGCTT TTCCTCAGAC CCTTATCTCA GTGGTGTAGC TATGGAGAAG
 701 ACCATCCTGG CATCTCAAGA TGCAGGTGTA CAGGCTTGTC CTAAACACTG GATTGGCAAC GAGCAGGAAA TCATGCGTAA CCCTACCTAC CGCAAGGACG
 801 ACAACACCGA ACAAGCGCAA GCTGCTATCT CATCCAACAT TGATGACCGC ACCATGCACG AGCTCTACAT GTGGCCGTTC GCCAACGCTA TCAAGGCCCG
 901 CGCTGCTAGC ATCATGTGCT CGTACCAGAG AATCAATGGA TCTTACGGAT GCCAAAACTC TGCTTCACAG AACGGTCTGC TCAAGGGTGA GTTGGGCTTC
1001 CAAGGATACG TCATGTCCGA CTGGGGTGCG ACCCACTCGG GTGTTGCTTC TATCGAGGCT GGTCTTGACA TGAACATGCC TGGTGGACTT GGTGCTTACG
1101 GTTTGAACTT TGGTGTCCCA TCTTACTTTG GCGGCAACAT CACTCTTGCT GTTAACAACG GCACTGTCAA CGAGTCCCGC ATCGACGACA TGGTCCTCCG
1201 TATCATGACT CCCTACTTCC AGCTCGGCCA GGACAACGAC TTCCCCTCCG TTGACCCTTC CTCTGCTGAT CTCAACACTT TCTCTCCACG AAAAAGCTGG
1301 ACCCGCGAGT TCAACCTTAC TGGCGAGAAG AGCCGTGATG TTCGTGGCAA CCACGCCGAG CTGATCCGTC GACACGGTGC CGCTGGCACT GTCCTGTTGA
1401 AGAACGTGGA CCGTACCCTG CCTCTCAAGG CACCCCGCAA CGTTGCAGTC TTCGGTAACG GCGCCAGTGA GCTTGCTCGT AGCTCTGTCC TTAACCAGCA
1501 AAACTACGAG TACGGCGCCA TCTTTGCTGG TGGTGGCTCT GGTACCGGCC AGAACACCTA CGTGATCACG CCCCTCACCG CTATCCAGCA GCGTGTTAGG
1601 GAAGACCGTG GCATTGTTCA GTACTTCCTG AACAACACCC ACATCATCAA CAACAATGTC AGCACCCTCG TCATCCCACG CGATGTACCA GATGTCTGCA
1701 TTGTCATGCT CAAGACCTGG GCCGAGGAGG GTGATGACCG CTATCACCTT GGATCTGACT GGAACGGCGA CAAAGTTGTC GAGTCTGTTG CATCTTTCTG
1801 CAACAACACC GTCGTCGTTA CCCACTCCTC CGGCATCAAC ACCCTACCAT GGTCTGACCA CCCCAACGTC ACCGCCATCC TGGCCGCTCA CTTCCCCGGT
1901 GAAGAATCCG GCAACTCCCT CGTCGACATC CTCTACGGCG ACTACAACCC CTCTGGCCAC CTCCCCTACA CTATCGCTAA GAACGGTGAC GACTGGAATG
2001 CGCCCCCCAC CACCGAGATC CAAACCGACG GCTTCAACGA CTGGCAGAGC TGGTTCGATG AGAAGCTCGA GATTGACTAC CGCCACTTCG ACATGCACAA
2101 CATCTCCGTC CACTACGAAT TTGGCTTTGG TCTCTCCTAC ACCACCTTCT CCATCTCCGA CATCAAATCT GCTCCTGTCA AGGATGCCAT CACCTCCAAG
2201 CCCGAGGACC TTCCCATCCA GCCCGGTGGC AACCCCGCTC TCTGGGAGTG CATCTACAAC GTCACCGTCT CCGTCGCCAA CACCGGCGAC GTCGCCGGCG
2301 CTGCTGTGCC CCAGCTGTAC GTCGGCCTAC CTTCTTCCGC CCCTGCTGGC ACTCCTGTGC GTCAGCTCCG CGGCTTCGAG AAGGTGTACC TTGAGGAGGG
2401 TGAGACCCAG AGCGTCAGCT TTGAGCTTAT GCGCAGGGAT CTGAGCTACT GGGATGTTGT TAGCCAGCAG TGGGTTATTC CCGAGGGCGA GTTCAACATC
2501 TGGGTTGGCC TCAGCAGCCG TGACTTGAAG GTTCATGATA GCTTTACTGT TGTTGGACAT TAGAGGAGTA GCCTGGGAAA TGGGGTTCAT GTCATTATGA
2601 GTATATGAAT AGATTATTAA TTTCTATTAT TTGAAGAGTC A
```

>*GH3-5*(jgi|Cocsa1|172113|fgenesh1_pm.8_#_376)

```
   1 ATGCGGTCTG CGATCTTCCC GTTGCTAGCT ACGGCGGCGC TGTCTACAGC CATCCCTTTC GACCAGCCTG CTTCGAAACG TGCAGTGGGC AGTTGGGATG
 101 ATGCATACAC AAAGGCAACA GCAGCACTCG CAAAGCTATC GCAAGATGAT AAGATCGGAA TCGTTACGGG TACCGGCTGG TCGAAGAGTA ACTGTGTCGG
 201 CAATACCAAG GCAGCCAGCT CGATTGGATA CCCCTCGCTA TGTCTACAAG ATGGTCCCCT TGGTATCCGC TATGTCCGGG GTATAACCGC CTTTGCAGCT
 301 GGCATCCATG CCGCCAGCAC TTGGGATATC GATCTCATTC GTGAAAGAGG TGCTTTTCTC GGTAACGAAG CAAAGCAATT GGGTATCCAC GTGCAGCTAG
 401 GTCCGTCTGC TGGTCCCCTT GGCAAGTTTG CCAAGGGCGG CCGTAACTGG GAGGGATTTG GATCAGATCC TTATCTTCAA GGCATCATGA TGGCACAGAC
 501 CATTGAGGGT ATGCAAGAAG CCGGTGTTCA GGCCACTGCT AAGCATTGGA TTGTGAATGA ACAAGAGCTC AACCGAGACA CTATGAGCTC TGATGTGAGC
 601 GATAGGGTTC TGCGCGAGTT GTATGTTTGG CCCTTCGCGG ACGCCGCTCA CAGCAAGGTT GCGGCATTCA TGTGCAGCTA CAACAAGCTT AACTCAACAT
 701 GGGCGTGTGA AAGCGAAGGC GTTATGCAAA AACTCTTGAA AGACGAGCTT GGCCACCGTG GATACATCAT GTCGGATTGG AATGCGCAAC ACACTACTAC
 801 TGGCAGTGCA AATGGTGGCT TGGACATGAC CATGCCCGGC AGTGATTTTA GTGGCGGAAA CGTGCTCTGG GGACCGCAAC TCAAGACCGC CATTAGCAAC
 901 GGTCAAGTTC AGCAATCACG CCTCGATGAC ATGGTCAAGC GAGTCCTTGC TGCGTGGTAC CTTATGGGTC AGGACAAGGG TTATCCCGCG ACTTCCTTCA
1001 ACTCCTGGAC AATTGGGTCA AAAGAAATCA GTGGAAACCA CGGGACCAAT GTCCGAGCGA CTGCTCGAGA CGGCACTATC CTGCTGAAGA ACACTAACGG
1101 TGCGCTACCC CTGAAGAAGC CAAAGAGCAT TGCTGTCATT GGAACTGACA GTATTGTCGC ACCTCGCGGG GCCAATGCTT GTGTCGACCG TGGATGTACC
1201 GAAGGTGTTC TTACCATGGG CTGGGGTTCG GGATCAGTTG AGCTTCCACT CAATATGGTC GCGCCACTGG ATGCCATCAA GACACAGGCC CAAAAAGACG
1301 GTACGACAGT TACTTCATCG CCCACCGACA ACGCCCAGCA AGGCGCTAGC GCAGCTCAAA ATGCTGAGAT TGCCGTGGTA TGCATCAACA GCAACGCTGG
1401 TGAAGGCTAC ATCAATGTCG AAGGCAACGC TGGCGACCGA AACAACTTAG ACCCCTGGCA CAATGGCAAT GAGCTTGTCA AGGCGGTTGC TGCCGTGAAC
1501 AAGAAGACGG TGGTTGTCGT TCACAGTGTT GGGCCAATCA TCATGGAGCA GTGGATTGAG AACCCCAACG TCGTTGCAGT AGTGTGGGCA GGTCTCCCAG
1601 GCCAAGAGCC CGGCAACGGC GTTGTCGACA TCATGTATGG CGCAGCGTCG CCAAGCGGCA AGCTTCCTTA CACGATTGCG AAGAAAGAGT CTGACTACGG
1701 CACCACAATT GCCAGGGGTG ACGACAAGTC CTGGGATCTG TTCATCGACT ATCGCTACTT CGACAAGCAG AACATCACGC CCAGGTTTGA ATTTGGCTTT
1801 GGACTCTCAT ACACCAACTT CACCTACTCA GATCTCACTG TAACTGGCAA GCCTTCTGCT GGTCCTGCGA CTGGTGCTGT AGGACCTGGA GGCCCGGTGG
1901 ATCTCTTCGA AACTGTTGCT ACGGTGACTG CCAAGATTGC CAACTCTGGT GGCGTTGCTG GTGCAGAGGT ACCGCAGCTG TATCTAGGCT ACCCTGCGTC
2001 TACCAACTCT CCACCAAAGC AACTTAGAGG CTTCACCAAG CTCAAGTTGG AGGCTGGTGC TAACGGAACC GCAACATTCA AGCTGAGGAG GAGGGACTTG
2101 AGTTTCTGGG ACGAGAAGAC GAGGAAGTGG ACTGTTGCGA CGGGCGAGTA CACTGTCTTC GTCGGAGCAA GCTCCAGGGA CGTGAGGCTG ACAGGCAAGA
2201 TTATTGTCCA CCTACCCAAC AAATCACCGA ACCAAATACC TCTCTACACC ACATCTTCCA TCATCATGAT CTCCCAACTA TTCAAACCCT CCACGGTCCG
2301 GTCCGCAGGT CTCCTGGGAC GAGCGACAAA GAACCGCGTA CAGGCACGCT ACCTGGCTAC TGTGCAATCA AACACCGAAC GTGTTATGCC TACTCCTAGC
2401 GCACGCAGGG CCACTGCCGT CTCTAACGAG CCTGCCACTT TCACTATCAA GAACGGCCCC ATCTTCGAGG GCAAGTCATT TGGTGCCAAA ACCAACATTT
2501 CTGGTGAAGC CGTCTTCACA ACCTCCCTTG TCGGATACCC CGAGTCTATG ACCGACCCCT CCTACCGTGG CCAGATCCTT GTCTTCACCC AGCCACTGAT
2601 TGGTAACTAC GGTGTGCCCT CCAGCGCCCG TGATGAGCAT GGCCTGCTCC GATACTTTGA ATCACCATAC ATCCAAGCTT CTGGCATTGT TGTCCAGGAC
2701 TATGCCCTGA AGCACAGCCA CTGGACTGCC GTTGAGTCGC TTGGTGCATG GTGTGCTCGC GAGGGTGTCC CCGCCATCTC TGGTGTCGAC ACTCGTGAGG
2801 TTGTAACCTA CCTCCGTGAG CAGGGATCCT CTCTTGCTCG TATCTCCGTT GGTGAGGAGT ACGATGCTGA TGAGGACGAG GCGTACATTG ACCCTGAGGC
2901 TATCCACCTC GTGCGCCGTG TTTCCACAAA GGCACCCTTC CACGTCAGCT CCTCTCTGGG AGACATGCAC GTGGCTTTGA TTGACTGCGG TGTCAAGGAG
3001 AACATCCTCC GCAGTCTCGT CTCTCGCGGC GCCAGCGTCA CTTGCTTCCC CTACGACTAC CCTATCCACA AGGTCGCCCA CCACTTTGAT GGTGTCTTCA
3101 TCTCGAACGG CCCTGGTGAC CCAACCCACT GCACGAACAC GGTCCACAAC CTCCGCAAGC TCTTCGAGAC GTCGCAGCTC CCAGTTATGG GCATCTGCAT
3201 GGGTCACCAG CTGATTGCCC TTGCTGCCGG TGCTAAGACG ATCAAGCTCA AGTACGGCAA CCGCGCTCAC AACATCCCGG CTCTCGACCT CACCACCGGC
3301 AAGTGCCACA TTACCTCGCA GAACCACGGA TATGCCGTTG ACCCGACTAC GTTGACGAGC GAATGGAGGG AGTACTTCAC CAACCTCAAC GATCAGTCGA
3401 ACGAGGGTCT AATCCACAGC TCGCGTCCCA TTTTCAGTGC GCAGTTCCAT CCCGAAGCAA AGGGTGGACC TCTGGACTCT GCCTATCTGT TTGACAAGTA
3501 CATTGAGAAT GTGCAGCAGT ACAAGGAGCA ACAGGCCAGC TTCTCACAGA GGAGCAACAA ACCCAGCCCA CTTTTGGTGG ATTTGCTGGC CAAGGAGCGT
3601 GTTGGCGTGC ATCCTGACGC GCCCGACTTC GAGGGCCATG CTGCGGGTAT GGACAGCCAG CAGGTGAATG TTGGTGGACC TGTTGCGCCT CCTTACCAGC
3701 CCATTATGCA GAAGCCTGTT GCTTCTGCGG CTTGA
```

>*GH3-6*(jgi|Cocsa1|149234|estExt_Genewise1Plus.C_13_t10334)

```
   1 ACCGGACAAC CTGCCTGCCA AGGTAGCAAT GCGGAGAAAG AGAGAACGAC GGATTTTGAG TCCGTCCAAT GGCTTTGGGA GGAATGGGTT CTCGTCACGA
 101 GGTCCGGTAC TACGTCGTGT TTTTCCCCAG ATATGACATC AGTAATAGGG CCGGAAGAGG AGAAGAATTG CAGGTCAACG GTTTTTCTAG AGTGAGTATT
 201 TAAGGTAGCT GAGGGGACGT CGTGGACGTG ACCTCTTTTT TGATCAGCGC TCAAGACGGG TTTTCAATAA AGTCTGCAAA TATGAGGTTC GCTGACGCTT
 301 TGGCTTTGGC CATTGCTTTG TTTCCGGGTC TCTCGGCTGC CCAAGGGTAT ACCAATGAAT CTGAAGTCCC GCTATATGGG CTGTCGCCGC CTGTGTATCC
 401 TAGTCCCCAG GGCAATGGTT CGACATCCGA AGCTTGGGCC GCAGCATATG CTCGTGCAAG AGCCGTTGTC GCGCAATTGA CGCAGGAAGA ACAGTCAAAC
 501 TTGACTCACG GCGTCACGGG ACGATGTGTA GGACAGACGG GCGCAGTGCC TCGTCTTGAC ATTCCTGAAC TCTGCTTCGC TGATGCTCCG GCTGGTATTC
 601 GCGGCCAAGA ATTTGTATCG GCGTTCCCTG CCGGCATTCA TCTTGGAGCG ACGTTCGACA AAGGGCTTAT GCTGCGATAT GGCCAAGCCC TAGGTCGAGA
 701 ATTCAAAGGA AAAGGTATCC ACATTGCGCT GGGCCCTTTT ATTGGGCCTA TTGGGCGTGT TGTAAGAGGT GGAAGGAACT GGGAGGGTAT GGGTGCTGAT
 801 CCGTATCTTA ATGGTGTTGG GGGTGGTCTG CTCACCAGAG GCACCCAGGA AGCAGGTGTC ATGGCGACTC CAAAGCATTG GATTGCTCAA GAACAGGAGT
 901 ACAGACGTAG ACCTGGAAGC GAAGGCGAGG CCGTTTCGTC AAATGTTGAC GACCGCACGC TACACGAGTT GTATGCTTGG CCCTTCATGG ATGCTCTCCG
1001 TGAAGGTGCT GTTTCTGTAA TGTGCTCTTA CCAGAGGATC AATAACTCAT ATGGCTGCCA AAACTCAGCC CTGCTGAATG GAATATTGAA AACTCAATTG
1101 GGGTTCGAGG GATTCGTGGT CTCCGATTGG GATGCACAAC ATGCGGGTGT CGCATCAGCA AATGCTGGAC TTGACGTAGT GATGCCCGTA GCCAAGTTCT
1201 GGGGCAAGAA TCTCACCGAT GCCGTGAACA ACGGATCGGT TACTGCTGAA AGGTTCAACG ATATGAACAC CCGACTACTG GCAGCCTACT ACTATCTCAA
1301 CCAGGATGAG GCCGGTTTCG ACCCCCAAGT ATACCCGTAC AATGTGAAGC ACGAGATTGT AGACGTAACG GAGGACCATG GCTCTTTGAT TAGGGAAATT
1401 GGAGCCGCAG GTACCGTTCT CGTGAAGAAC GTAAACAACA CCCTTCCCCT TATTAATCCC CGCTTCTTGA ACATCTACGG CTATGACGCA GAGGTAAAGG
1501 CCTCGCCCTG GAACAACCCC TCGCGTTTCG GCGGTGGCTA TGAAGAAAAC TTTGGCTGGA ACACATTGAA CGGCACATTG ATAACAGCAG GCGGCTCGGG
1601 TTCTAGCACA CCTCAATACG TGATCAGCCC GTTCAAAGCG CTGCAAGACC GGTTAATTGC GAACCGCGGA ACACTTCGCT GGGATTTTTG GGGCGTGAAT
1701 CCCACTGTTT ACGCAAATGC CGAAGCATGC CTTGTGTTCA TAAACGCATA CGCATCCGAA AGTTTTGATC GAACATCTCT CACCGACGAT TTCAGCGACC
1801 AGCTTGTTAA CAACGTAGCT ACAAACTGCT CCAACACCAT TGTCGTTATC CACTCAGCTG GTGTCCGCAC GGTTGACGCA TGGTACGACC ATCCCAACGT
1901 CACTGCCATC CTATATGCTG GTGTCCCGGG TCAAGAAAGC GGAAACGCAA TCGCCGATGT ACTCCTCGGC GATGTAAATC CCAGTGGCCG ATTGCCATAC
2001 ACTGTCGCTC GCTCCGAATC TGACTACGGC GCCTTGCTCA ACTCGACTTT CGACCCTTCC ATCCCGGCCT TTTTGCAATC AGACTTTACA GAAGGTTTGT
2101 ATATCGACTA CCGTGACTTC AACAAGCGCA ACGTCACACC ACTGTACGAA TTCGGCTTTG GGCTGTCGTA CACTACCTTT TCTTACGGCG ATTTGTCGAT
2201 TGAGTCGGTA GCACCAAACA CTTCGCTCTC GCGCTTCCCA TCTCCCTCGG TGCCAATTGT GCAGGGCGGC CACCCAGAGC TATGGGATGT GCTGTACCGT
2301 GTCAGCGTCA ATGTCACCAA CACGGGTAAA GTAGCTGGAC ACGAGGTACC GCAATTGTAT CTTGGTGTTC CGAATGCGCC GCAGAAGCAA CTGAGGGGCT
2401 TCGAGCGTGT GGGCTTGTTG AAGCCAGGCG AGAGCGCTGT CGTGGATTTT GAGCTTCAGA GACGTGATTT GAGTATTTGG AATGTTGAGG CTCAAGCGTG
2501 GGAGCTGCAG AGGGGACGAT ATGGAGTGTG GGTTGGCGCG AGTAGTCGTG ACGTAAGACA GGAGGGGGAT ATTATTGTAC AGTAAGCCAT GGAAGGAGGC
2601 AGGTATGACC AGATAATTGT GCAGTCACTC TACGATGCT
```

>*GH3-7*(jgi|Cocsa1|94783|e_gw1.10.1255.1)
```
   1 ATGGCTTTTA TTTTCGCAAC TCGAATTTTG CTCGTACTAT TGTCCACCTT CTCTCTTTCA TCTGCGCAGA TCCCGGATGG CCTGCCTATC GACGATGCCA
 101 CGAGGCAGTA CCTCGAGTCT TTGCCTGCAG AAAAGCTAAC CAAGATCTTG AAAATCTTCC AGCAAGCTAT TGACGGTCAG GTTCCCGCAA CGCTCGAAGT
 201 GATTGAGGCT CAGGAGCTCT ATTGGAGCTA CAATAGGGGT CCTCCTTTCT ATCCAACCCC ACAGGCCAAA GGATTAGACG ATTGGGCCGA TGCTTATCAG
 301 CAGGCATCGG CAATAGTTGA CCAGATGACT ATTGAAGAGA AGGTTTCCGT TGTCTCCGGA CAAACGTCCA CCACAAATGG ATGCTCGGGC CAGATACCCG
 401 GAGTTCCTCG TCTTGGATTT CCTGGCATTT GTGTAAATGA TGGTCCGAAC GGTCTTCATG ACATCGCTGC TGTGAACGGC TATCCATCTG GCATCACTAT
 501 TGGTGCGACA TGGAACAAAA CACTTGCCCT TGAACGTGGT CAATTTATGG GTCTGGAGTC CAAGGTGAAG GGCGTCAACA ATCTGCTGGG ACCGACAGTT
 601 GGCCCCCTTG GCCGCGTAGT CATGGGCGGC AGAAATTGGG AATCTTTTTC GGTGGACCCT TACCTTTGCG GCATCATGGG TGCTCAAACT GTGTTGGGGA
 701 TGCAGGAGCA CGTTATTGCA ACTGCAAAAC ACTTCATCCT CAACGAGCAG GAGACTAATC GCAATCCAAG CTCTTTTGGG CTTGGGAACG CGTCTGTATC
 801 GGCTACCGTG GATGACAAAA CGATGCATGA GCTGTACCTA TGGCCGTTCC AGGATCTCGT CAAAGCTGGC GTAGGATCTG TCATGTGCTC TTACAATCGC
 901 ATCAACGGCT CTCATGGTTG TCAAAATAGT TACACCTTGA ATTATTTGCT AAAGACTGAG CTCGCTTTCC AGGGATACGT TCTCAGTGAC TACGGTGCTT
1001 TACACACCGG CATTGCCGCG GCCAACGCCG GTATGGATGT TGTCACGCCA TTTGAAGAAA TCTGGGGCAA AAACCTCACA ATGGCAATCT CAAATCACAC
1101 ATTGGAGGCT AGCAGGCTCG ACGACATGGT TAAACGCATT GTCGCTCCCT GGATAAAACT TGCGAAATTT GAACCGGGTT CCGGGATTCC AGCAAACGTT
1201 TCTAAGAAAC ATCAGGTCGT GAGCGCAATA AATCCTTCGT CTCGAAGAAA CATCTTCCAA GGCGCTGCTG AAGGCATCGT TTTGGTGAAG AACCACAACC
1301 ACACGCTACC CTTGAAGAAG CCAAAGATAC TTAGTCTTTA CGGATACGAT GCTCATATTT CTCTAAAGAA CATGCCCGAA GGTCTTAACA CAAAGTATAG
1401 CCTGGGATAC CAGAGTGTCA ATGTCACTGA TGAAGAAATG CAAGGCTTGT TCATCGGCGT CGGAGACCTG CCTGGGGCCG CGCGATCCGG TACGCTTCTC
1501 AGTGGAGGCG GTAGCGCCTC TACCGTGCCT GCTTACGTCT CTTCACCCTT CGCGGCTTTC TCGCAAAGGG CGCTTGAGGA TAATACCTTT TTATTTTGGG
1601 ACTTTGAATC CCAGGATCCC GTCTATGCAA ACGCCGGCTC TGACGCTTGC ATCGTTTTTA TCAATGAATT TGCCGCAGAG GGGCAGGATA GGTCCACGCT
1701 TGCTGACCGT TGGTCGGACA AGTTGGTGAT CAACGTTGCC TCTAAATGCC CTAACACTAT TGTATCTATC CATAACGCGG GCACGCGCTT GTTAGACCAG
1801 TGGATCGAAC ACCCAAATAT CACCGCAGTT CTTTTTGGTC ATCTCCCGGG TCAGGATTCA GGCAGTGCAC TCGTATCAAT TATGTACGGC GACCAAGCTC
1901 CCTCCGGCCG CCTCCCTTAT ACCGTCGCTA AGAGAGAATC TGAATACGGC GACCTCCTCT CTCCCATACG CGCCGACAAT ACCTCAAACT ATTACACATC
2001 CGCCAATCTG ACCGAAGGCG TCTACATTGA CTATCGCCAC TTCGATGCCC ACGACATTAT ACCTCGTTTT GAGTTTGGCT TCGGCCTTAC ATACACCACT
2101 TTTGAATACG CCAGCCTTAA CATCGCTCTC ACTGATCTGG GCGCAAATGC CTCTGCTCTG CCACCACCTG CGCCTGTGGG TCAAGGCGGT ATCGAGTCTC
2201 TCTGGGATGT GCTAGTCGCT GTTAAAGCCG AAATCACTAA TACAGGTCTC ACCTCTGCAC CTGAAGTTGC TCAACTGTAT CTTGGTATTC CAGGAGCGCC
2301 TGCGAAGCAG TTACGCGGAT TCGAGAAGGT GCCGTTGGAG GCAAGTGAGA GCACGACTGT CCACTTTCAC CTTACAAGAA GAGATCTTAG TATATGGGAT
2401 ACTGTGAAGC AGAGCTGGGC ACTACAAAGA GGAAAGTATA TGATTTATGT TGGGGCGAGT AGTCGGGACA TAAGACTGAG TGGAAGCTTA ATCATCTGA
```

>*GH3-8*(jgi|Cocsa1|33954|fgenesh1_kg.3_#_57_#_Locus5209v1rpkm21.01)
```
   1 GGCGGGTACT AAAAGATGAC CACGTCCCTC AGTTGACCAT CTTCGAAGTC TACCGCATAC CTCTAGTCTT GTCTCAATCT CCCATTCCTT CGCTTTCGTC
 101 GTCTTTTGAT TTCATTTGAT CGCTCGCTTC ACTCGCACTG GTTGCACGTG GCGAGAAGCG CGGCAAACCG CCCCAGATAT CCCGAAGTTA TCGAATCATT
 201 TAATTCAGCC TTTGAGGAGA TCTACAATGG CTACAATTAA CCTGGGCAAC GCTAGCCCTA CAGATGATAT CAAGCAAATC GACTTAAAGC ACCCGGCCAT
 301 CCAGATTCAA CCTCTGACCC TCGACACTCC CCTCTTGACT CCTGCTGCAA CGCCCGAGGA CGTTTCCCCA GCCGACGACG AACCAATGGC GGGCAAAAAG
 401 CTGTTTTCAA TGGAGAGGCC TGACGACAAG GCGAGGAACC TTGCTGCCAA GTTGACCTTG GAGGAACAGA TTTCGTTGCT TGCAGGAGCT GACTTTTGGC
 501 GCACTGTGGC TATTCCTGAA AAGGGCATTC CGTCAATCAA GACTTCGGAT GGACCCAATG GCGCTCGCGG AGAGTTCTTC ACTAACGGCA CACCTGCTGC
 601 TCTATTTCCA TGCGGCATAT CCATGGCATC CACGTGGAAC TTGGACATGA TTGAAGAGGT TGGCCGCCAC CTTGGCGAAG AGGCAAAGGC TCGTGGTGCA
 701 CAGGTGCTGC TTGCACCCAC TGTGTGCATG CACCGATCTC CTCTCGGAGG CCGCAATTTT GAGTCGTATT CTGAGGATCC TTTCCTGACT GGAAAGCTTG
 801 CTGCTTCCTA TGTTCGTGGC CTTCAGAGCA AGGGCGTCGC TGCTACCATC AAACACTTTC TCGGAAATGA GCAAGAGACC GAGCGACAAG CGTACGATGC
 901 CATCATTGCA GAGCGGCCGC TGCGTGAAAT CTACCTCAAG CCTTTTGAGA TAGCTGTCCG AGACGCATCA CCATGGGCGC TCATGAGTTC TTACAACATG
1001 GTCAATGGTG TTCATGCTGA TGAGTACATT CATTCTATCA AGGAAGTCCT CAGAGGCGAG TGGAAATGGA ACGGAGCCAT CATCTCTGAC TGGACGGGCA
1101 CATATGCAAC TGCGCCCTCA ATCAAAGCTG GCGTCGACAT CGAAATGCCA GGCCCGTCCA AGTGGCGCAA AGTTGAGCAA ATAAAGGAAT GTCTCGACAA
1201 TGGTGAGCTA ACTCCCAAGG ACATTGAGGA ATCCGCTGCC AGGGTACTTT ACCTTGTCGA TCGGGTCAAA GGACTCAACA ATATGACACC CGAAGAACCT
1301 GAGCGGTCAA TCGATAACAT CGAAACCCGG AACCTGATTC TCCAAGCTGG TATTGAAGGT TTGACGCTAT TGAAGAACGA GAGCAACGTT TTGCCGATTA
1401 GGGGTGCGAA GAAGATTGCA ATGATCGGAC CAAATGCCAA GCGTGCGATT GCCAGTGGTG GAGGAAGCGC TGGCTTGAAC CCTTACTACA ACGTCACACC
1501 CTGGCAAGGC CTCCAGAATC GATTCGACGG CGTGATCTCA TTTGCGCAAG GTTGCGACAG CTCAAAGTGG CTGCCACTGG CCTCTCCCTA CTGCAAATCG
1601 AATGGTGAGA CTGGTGTACG ATTGGAGTAC TACAAGGGGG ATAGGTTCAA GGGCGAACCT GTTGTTGTTC AGCACAAGGT CAGCACAGAT CTCTGGCTAT
1701 GGGATTCGGC GCCTATGGAG TTACTTCCGG ACTTTTCGTT CAAGGTCAAG ACTACACTCA CACCAAAGTC GAGTGGAAAC CACAGCTTCA GTTTCGCTTC
1801 AGTGGGACCA GGCCGCATGT TTATCGACGG CGAGTTGTTT GTTGACAACT GGGATTGGAC TGAGGAAGGC GAAGCTATGT TCTCAGCATC TGAGGATGTG
1901 CTCAAGTCGA TTTACCTAGA GGAAGGCAAG CCAGTCGAGA TCCTTATTGA GAGCACTAGC GAAGTACGGC CCGCCTCAAA GGTGTCCGTT ATCGGCAGGC
2001 GTCACGACTA TGGCGGTTGC CGCATCGGCT ATCAAGAAGA AGACAAGATC GATCGCCTAC AAGAGGCTGC AGATGTTGCA CGCGATGCCG ACGTCGCTGT
2101 CGTGGTAATT GGGCTGGACG CAGAGTGGGA GTCAGAAGGA TACGATCGCC AAACAATGGA CCTTCCAAAG AACGGCTCTC AAGATCGTCT GATCGAAGCT
2201 GTTCTAGCAG CCAACCCCCG AACTGTCATT GTCAACCAGT CTGGAACACC AGTCACTATG CCATGGGTGC ACAAGGCCCC CGCAATCTTG CAAGCATGGT
2301 ACCAAGGTCA AGAGGCTGGA AACGCGCTTG CAGATGTTCT GCTTGGAAAC AGCAGTCCTA GCGGCAAGCT GCCAACGACT TTCCCAGTCC GGATCGAAGA
2401 CAACCCAGCC TACCACAACT GGCCTGGTGA GAACCTAAAG ACTATCTACG GAGAGGGCAT CTACGTGGGT TACAGGCACT ACGAGCGATC CAAGATCGCA
2501 CCGCTTTTCC CCTTCGGCCA TGGCCTCACC TATACCAGCT TCGAATACGG AACACCAAGC ATTAGTAGCG ATGTTCTGTC CGAAAGCGAC GACATTACCA
2601 TCACTGTACC CGTTACCAAT ACAGGTTCCG TAGCCGCCCC CGAAATTGTC CAAGGGTACG TCAAGGATGT CAAGTCCACG CTCCCTCGCC CAGAAAAGGA
2701 ATTGCAAGCT TTTGGCAAGG TGTTCCTACA ACCAGGCGAG ACCAAGGACG TGGTCTTGAA ATTGAACAAG TACTCAGTGG GCTACTTTGA CACAAATCTG
2801 GGCCAGACTG GTGCTTGGAT TGCGGAAGAG GGAGTTTTTG AGGTGTTGAT TGGCGCGAGC AGCGCTGATA TCAGGGCAAA GGTCAGCTTT GAGGTCAAGG
2901 AGAGCTTCCA ATGGATCTTC TAGGCGGAGA AGACATTGTT ATGAGTACTA CGAGTTTTTT TATGTTGAAT ATTCTCAGCG GCTGCGAAGG CATTGTTGGT
3001 GCGCTGAATT GGCTTCGTGA GAGCTGTGAC GCTCCGTCTG TTGAAGATGT TGAGGTCAAG GGCATCACAC ACACAGTAGT TTGTATTGTT GAAATTTCTT
3101 CAGAAAGGGG AGAAATCATA TTGTGGATGA GAGTATAGTC GTAGGGTTGG TCGCTAGGGA GAGGACGGTT GAAAGCCCCT CAGTATTGGT AACATCTAGT
3201 AAATATTGGT AGCATCAAAG CATACTAATC ACATCTCGAA ATCTATATTT AAAC
```

>*GH3-9*(jgi|Cocsa1|147447|estExt_Genewise1Plus.C_11_t20096)
```
   1 ACCGAGCGGT GATACTCGCA ACATAAATTT TGCATCCCGG CTGCATGTAG CAAAAAGCAT TTGTACTCTC TTTTTCTCAG TCTCAGAGCC CTGGTCACAG
 101 CTCTATCTCG TTACAAAACT ACAGCCATGA GCTCAAAAGT TGAGACTATT CTCCAGTCTC TGACACTGGA AGAGAAGATC ACTCTCCTTG CTGGCAAGGA
 201 CTTTTGGGAA ACAGTTCCAA TCCCAGAAAA AGGCGTTCCT GCTATCAAGA CCAGTGATGG ACCGAACGGC GCACGCGGTG GAGTCTTTAC CGGAGGAACA
 301 CGAGCAGCCT GTTTCCCTGC TGCAGTATGC ACGGCCGCAA CATGGGATCC TGCAAACGGG AAGCGGATTG GCCATGCCTT GGCAGAGGAA ATCAAGACTA
 401 AAAGCGCGAG AGTGCTACTC GCACCCACCA TGTGCAACCA CAGGCATCCA CTCGGTGGCA GAAATTTCGA ATCATTCTCC GAGGATCCGT ATCTAGCCGG
 501 AAAGATGGCT GCTAATGTTG TGAATGGACT TCAGGAGAAA GGCATTGCAG CAACGATCAA GCACTTTGCG GCAAACGAGC AAGAGACGGA TCGATTGCGA
 601 GTCAATGAGA TTGTCTCGGA ACGTGCGCTG AGAGAAATCT ACATGAAGCC TTTTGAGATC ACTGTTAAAG AAGCCAATCC TCTTGCCGTT ATGACCTCGT
 701 ATAACAAGGT CAATGGTACA CATGCGGACT CAAATGAGTT CTTATTAAAG CAAGTTTTGC GTGGCGAATG GGGCTGGAAC GGTCTCGTTA TGAGCGATTG
 801 GGGCGGCACA AACTCTACTG CCGACTCACT GAATGCTGGA CTGGACCTCG AGATGCCTGG ACCAACAAGA TGGCGTAGCG TCGAAGCAGT GACTGAGGCG
 901 GTTAAGAAGG GCGAAGTTAC AGAAGCTACC ATCACTGAGC GAACGAGGAA CGTTTTGAAC TTGATTGAGA AGGTAGGAGC GTTCGAAAAC CCAGAGATAC
1001 CACCAGAACG GTCTGTAGTC GACCCGAAGC ATTGCAGGCT CATCCGCGAT GTTGCCGGAC AGGGAATCAC GCTGTTGAAG AATAATGGTG TTTTGCCGCT
1101 CAGAAAACAG CAAGTCAAAG GCAAGAAGAT TGGGCTTTTT GGTCTAGCCA AGGAAGCCCT CATCCATGGC GGTGGTAGTG CATCTTTGAA TGCTCACTAC
1201 CGCATCACGC CGGAAGAAGG TCTCAAATCT GCATATGGCG ACGATGTAGA ATTCAAGTTC GCTAAGGGCG CAGCAACGTA CCGTCTCATC CCCCCTCTAT
1301 CAAAAGGCTG TAGAGACATG GAGGGAAATG ATGGATGGAC GATGAAGCTT TTTGACAAAG ACAACAAAAG TAAGCCGATT AAAACGGCCC ATCGTGCCAA
1401 AGAGGCTGCA TTCTCTCCTC TGCTGGAACA TCAGGCCAAA CACAGCGAGG TCGTATTTGG CGCGACTTTT TACCCCACCG AGTCTGGCTC GCATTATCTT
1501 GGATGCTCAG GTATCGGCCC AACGACTGTA TACATCAATG AGGAACTCGT TTTCGAGCAA AAGCACAACT CACCCGACGC AATGGGCTTT CTCTTCGGTG
1601 GCAACCCAGA GAAAGAGTTC CGCGTCCCTT TTACAAAAGG CCAATGCTAC AAGATCGAGA TCATTTCAAG ACCACCGAGC GGCAGCAACG ACGACGCTGG
1701 TATCCTCGAC GGCCTCCCAG GCTTCAGACT CGGCTTCATG TACGAAGAAG AGCACGACAA AGACCTTGCC GCCGAAGCCC AAGACCTCGC AAAAGACTGC
1801 GACTACGCCA TCGTCTTCAC GGGCCACACG CCTGTCTGGG AAACCGAAGG CCAGGACCAA GCCAGCTTCC ACCTACCACG CGAAGGCACA CAAGACAAGC
1901 TCATCGCCGC CGTATCCTCG GTCAACCCCA AAACCATCGT CGTAAACTCC ACCGGCGTAC CCGTCGCCTT ACCCTGGCTC GACAACGTCG CCGCCCTCGT
2001 CCAAGCCTGG TTCCCCGGCC AAGAAGCCGG AAACGCCATC GCGGACATCC TATCCGGCGC CGTAAACCCC TCGGGCCACC TCCCCATCTC CTGGCCCAAA
2101 CGCATCGAGG ACGCGCCTGC ACATGGCAAT TTCCCCGGCG AGAGAGACGA GAGCGGCCAG CTCACCGTCA AGTACGCCGA AGGCGTTTTC GTGGGATACA
2201 GACACTACGA TCGTCTCGGT AAGGAGAAAG TACATTTTCC ATTTGGATTC GGGCTTTCGT ATACGGCGTT TGCCATCTCC AACGCGGCTG TTGCAAAGCT
2301 GTCTTCGGGC GCTTTTAATG CTTCTGCTAG CGTCACGAAT ACGGGAAGCG TGGCTGGAGC TGTGGTTGTA CAGTTGTATG TTGGGAGGAA GGAAAGGTCG
2401 GATGAGCATC CTGTCAAGAC GCTTGCGGCG TTCCACAAGG TGGATCTTCT GCCTGGGGAG GAGACAGCGG TTTTGCTGCC ATTTGCGAAT AGGGATTTTG
2501 CATACTTTGA TGAGGAGGCC AAGGAGTGGG TGGTGGAGAA GGGGTTGTAT GAGTTTTCTT TTGCGCAGAG CGCGGGACAT GTGGATGAGG TTATTGAGGT
2601 TGAGGTTGAG GGTGTGAGGG GGTTGAAGAT TTGAACGGGT GGAGAGTTGA ATGATGTAGT ATTTTGATAA TTAGCGGTTA TGAAGAGAGG GAATTGACTT
2701 GAAATTTCTC AACACT
```

>*GH3-10*(jgi|Cocsa1|176458|fgenesh1_pm.19_#_29)
```
   1 ATGATTCGAT ATACTTTCCT ACTGTTCCAT ATTGCATTCG CGAGCACACA GTCTAGCAAT GCTCACGGTG CGGCCTACAC TCAGTATGGA GGCAGTCCGC
 101 CGGTGTATCC TTCACGTATG TTGATTATTT GTCGTCTGCG TCCACACACT GACTCATCTC TCGCAGCAAA CGTCACAGGT ATCGGGGGAT GGAACGAAGC
 201 ATTGAATAAA GCGCGAGCGT TCTTAGACAA TCTCACTCTT GAAGAGAAAT CTCTAATAGT GACTGGAGCT GCAGGTCCTT GTGTTGGAAA TATCGCCCCC
 301 ATCCCGCGAC TGAACTTCAG CGGTCTTTGT CTTCAAGATG GCCTTCTCAC TATTCGTCAA GCCATCGGCG CTTCACTGTT TCCTGCCGCC CTCACGGTTG
 401 CTGCCTCTTG GGATCGTTCG CTCGCTCACC AGCGCGGTGC TTACTTGACC AAGGATTTCC GGGAAAGGGG CGCGCACGTC GCTTTAGGAC CTTCCATAGG
 501 CCCTCTTGGT CGTTCGCCCT TCGAAGGCTG CAATTGGGAA GGCTTCAGTC CAGACGCCTT TCTTTCAGGC ACTCTTGTCG AAGACACGAT CAAGGGTATG
 601 CAAAGTACAG GAGTACAAGC TTGCGTTAAG CACTTTATTG CCAACGAGCA GGAAACAAAG CGAATGCCAT CAGTGAACAA CAACGGCGTC ACTATCGACG
 701 CCGTGAGCTC TAATGTTGAT GACAAGACTA TGCACGAGAC TTACCTTTGG CCATTTTCAA ACGCCATTCG AGCTGGTTCG GCGTCTGTAA TGTGTTCCTA
```

```
 801 CAATCGTATA AATGGAAGCT ACGGATGCCA GAACTCAAAG GCGCTCAATG GACTCTTGAA GAATGAGCTT GGATTTCAGG GCTATGTCAT GTCCGATTGG
 901 GGAGCTACCC ACGCTGGCAT CGCTTCGTTC GAAGCTGGAC TCGATATGGA CATGCCCGGG GGGCAATCTC TAGTGGCGAA CAACAGTACT CTCCATGTTG
1001 AAAGGCTAGA CGACATGGTT CTTCGAATCA TGACACCATA CTACTTGCTG GGCCAGGATA GAGGCTATCC AGCTATTGAT ACGTACACGA CACAGCTAAA
1101 GTTCTTACCG GAGCCTGTCT ATACCCACAA CTACACGTAT AGTCCTGGAT CGAACTACCG TAATGATGAA GCCACTCAAC TCATCAGGAA GTTGGGAGCA
1201 TCGGGCACTG TGCTTTTGAA GAACACCGAC AACACGTTAC CGCTTCAGCA ACCGAAACAA ATTGACGTGT TTGGAAATGA TGCCGCAGAC TTGACCACCG
1301 GTCAGTACCA AGCTATTGAC TACGATATCG GGACTCTCCC TATTGGTAGC GGAAGTGGTA CAGGCTTTTT CTACCATGTC ATCTCCACGC TGGAGGTGAT
1401 CAAATCTCGA GCAGCCTCAT ATAGTGCTCA TGTTCAATAT GTCATTGGCA ACACATTCAT CGCTCTCGGT GGACTATTTG AACTGGCGCC AAAGCCCATA
1501 AATGTATGCA TCTTGTTTCT CAAATCATGG GCTTCTGAGG GTTCTGATCG TACCACATTA ATCCCAGAAT GGAACTCGAA GGTTGTCGTG GAGCGTGTCA
1601 CTGGCGTGTG CGACAACACC GTCGTCGTCT TGCACGGTGC ATCACCGAAC ACAATGCTAT GGCGAAGCAA TCCCAAGGTC ACGGCTATTC TGACCGCCCA
1701 TATGCCTGGA GAGCGAACAG GCAACTCAAT CGTCGATATT CTTTGGGGAG ATGTGAATCC CTCTGGCAGG CTTCCGTATA CAATTGCGAA CAACGACACG
1801 GAATACCGGA AGAATATTGC GAACAGCATT GCGTTCCAAG ACACTGATGA CCCGTCTGAC TGGCAAGCCG ACTTTTTCGA AGGCAACCTA ATAGACTATA
1901 AGGACGTTGC ATTTCCTTTC GGCTTCGGAC TAAATTACAC GACTTTTAAT GTCTCAAACC TCGACGAATG CCCTAATGCC CCGATTCATC CAGGTGGCAA
2001 GATGGAACTC TGGGACATAC TCGTGACAGT CGAGGCTACT GTTACTAATA CTGGTTCAAG ACTTGGGGCC GCCGTTCCAC AACTCTATCT CTCGTACCCC
2101 AGTGAGGCGA AAATGCCTGT GAGAAGCTTA CGAGGCTTTG ATAAGATCGA CATCAGCCCA GGAACGACAG AGACGGTTCT ATTCGAATTG ACGAGGCGCG
2201 ATCTGAGTTG CTGGGATACG AAGGTTCATG CATGGAGGTT ACCTACGGGA GATATCACAG TACAAGTTAG ATTTAGTTCT CGGGATCTAC CACTGCAGAG
2301 TATTATTGGT ATTTAA
```

>*GH3-11*(jgi|Cocsa1|182254|estExt_Genemark1.C_9_t10052)

```
   1 ATGGACCTTA TGCTGAGAGA TTATCTGCAA AGTACTAGAG AGAACACATC AGCCGAGCTC GACCCTGAGT TCTTAACAAA TGCTATTACT CAGGTCAAGA
 101 CGTTGCTTAT CGCTGGTAGC GGCACAACAT CAGACACGGT CTGCTTTGGA CACATGCTAC TGAGCGTGCA TCCAAAAGTA GTTCGAAAGA TGCGAGAAGA
 201 GCATGACCGT GTCTTCGCTC CCGGCATCGA CGCAACATAC GATCTTCTAG AGGCTAACCC GTACAAAATG AACGACCTTG TTTACAAGAT TACCGTAATC
 301 AAAGAGATTC TCAGATTCTA TCCGATAGCT ACACTCGCTC GTGCAAAGTC TCTTGTAGCA CTTTACACCC TCGAGGAGAA GATCAATGCT ACTTCCAACA
 401 GCGCACCTGG CGTTGCCCGT CTCGGTATTC CACCTTACCA ATGGTGGAAC GAGGGACTCC ACGGCATTGC AGGTCCCTTT ACTAGCTTCG CCAAGCAAGG
 501 CGACTACAGT TACTCAACAT CCTTCCCCCA GCCTATCTTG ATGGGTGCCG CCTTCGACGA CAACCTGATT ACCGAGGTTG CAAATGTTAT TTCCACTGAG
 601 GCGCGAGCTT TTAACAATGT CAACAGAACG GGTCTCGACT TTTGGACGCC CAACATCAAT CCCTTCCGTG ATCCTCGATG GGGACGTGGG CAAGAGACTC
 701 CCGGAGAGGA CTCGTACCAC CTTAGTTCTT ACGTCAAGGC TTTGATCCAT GGACTCCAAG GTAACGAAAC GGACCCTTAC AGGCGTGTGG TCGCTACCTG
 801 CAAGCACTAT GCTGGGTACG ATATCGAAAA CTGGAACGGC AACCTCCGCT ACCAAAATGA TGTTCAGATC TCACAGCAGG ACCTTGTGGA ATACTACCTA
 901 GCTCCATTTG AGGCTTGTGT TCAAGCCAAT GTTGGCGCCT TTATGTGCTC TTACAACGCC GTTAACGGTG CGCCACCATG TGCCGACCCT TACATGCTTC
1001 AGACAGTTCT CCGTGAGCAC TGGGGCTGGT CCAGTGATGA GCACTGGGTC ACATCAGACT GTGACTCTAT TCAGAACGTG TACTTGCCTC ACCAATGGTC
1101 TTCGACTCGT GAGGGCGCTG CTGCCGACTC TCTGAATGCG GGCACTGATC TGGATTGTGG TACTTACCTG CAGTCGCATC TTCCTGGAGC TGTCAAGCAG
1201 GGTCTCACTA ACGAGACTAC CCTTGATAAT GCTCTCATTC GCCAGTACTC TTCTCTCATC AAACTTGGTT ACTTTGATAT ACCTGAAAAC CAGCCCTATC
1301 GCCAGCTTGG CTTTGATGCG GTAGCAACTA GTGCTTCTCA GGCACTTGCT CTCAAAGCTG CTGAGGAGGG CATCGTTCTT CTGAAGAACG ACGGCGTTCT
1401 TCCAATTAAC TTCGGTTCCA AGAATGTTGG TATCTACGGT GACTGGGCCA ACGCCACTTC GCAATTGCAG GGCAACTACT TCGGAGTCGC CAAATTCTTG
1501 ACTAGTCCAT ATATGGCCCT CGAAAAGCTT GGTGTTAACG TTAGATATGC GGGCAATCTC CCAGGCGGTC AGGGAGACCC TACTACAGGC TCCTGGCCTC
1601 GCCTCTCCGG CGTCATCACC ACGAGTGACG TGCACATCTG GGTCGGTGGG ATGGACAATG GCATTGAATC CGAAGATCGA GACCGCTCCT GGTTGACCTT
1701 GACAGGTAGC CAGCTTGATG TTATTGGTCA ACTCGCCGAC ACTGGAAAGC CGGTTATCGT AATTATCATG GGAGGTGGCC AGATAGATAC CTCCCCGCTC
1801 ATCAAGAACC CCAAGATCTC TGCGGTCCTA TGGGCTGGAT ACCCCGGCCA AGACGGCGGC ACCGCCATCG TCAACATTCT CACTGGAAAA GCTGCTCCTG
1901 CTGGACGTCT CCCACAAACA CAGTACCTAT ACAAATATGT CTCCGAGGTA CCAATGACGG ACATGGCTAT GCGCCCAAGC AACAAGAACC CTGGCCGCAC
2001 ATACAAGTGG TACACCGGCA AGCCCATCTT CGAATTCGGC TACGGTCTCC ACTACACCAA CTTCTCCGCC AGCATCACCA ACCAGCCAAA ACAGAGCTAC
2101 GCCATCTCTG ACCTCGTCAA AGGCTGCAAC AGCACCGGTG GCTTCCTCGA GCGCTGCCCC TTCACCGGCA TCAACGTCAG CGTTCAGAAC ACGGGAAAGA
2201 CATCTTCCGA CTACGTCACT CTCGGCTTCC TCACCGGTTC CTTCGGTCCC AAGCCGTACC CCAAGAAATC CCTCGTCGCC TACGACCGCC TCTTCAACAT
2301 CGCCGCTAGC TCTTCTTCGA CTGCTACGCT CAACCTTACG CTTGCGTCTC TTGCACGTGT GGATGAGTCC GGCAACAAGG TCTTGTATCC TGGAGATTAC
2401 GAGTTGCAGA TTGATAATGC GCCGCTTGCT AGCGTCAAGT TTGCACTTAC TGGCAGCGAG ACGACGCTGA GCAAGTGGCC GCAACCGCCT TCCAACCGTA
2501 CGGGTCAGGG TGTGGATTTC TTCGGAGATT ATTGGTATGG TGGGAATTAA ATAGAAAGAA TTAAACAAGG TC
```

>*GH3-12*(jgi|Cocsa1|209054|CE4400_281)

```
   1 CCCCCACCGG CCCGCACACC CTGGTGGGAA CGTACTTATA AGAACCGTGG TACCTTGTCT ATAACCTATC GTCTAGATAA GCTATACTCG TGTTTCAATT
 101 TAAGATGCGC TCGCCTCTCT ACAGCGTAGC CTCTCTTCTA GTTGGGTATA TTCTATACCT TCCCAGTAGC GCTAACGGTC TTGCCCGAAG AAATGACACG
 201 GCACAGGCAG TATACAAGGA CGCTGCTGCT CCCATCGAGG ACAGAGTTTC TGATCTTCTT TCACGGATGA CAATCGAAGA GAAGACAGCT CAACTAATCC
 301 AAGGCGATAT CTCAAACTGG ATCAACACTA CCACTAATGC TGTCAACCAG ACCGGTCTCG AGTGGAACTT TAGGGTCCGT GCAGGTCAAT TTTATGTCGG
 401 CTATCCCATG CCCGCAGAAT GGATTAGCAA TGGCATCAAG ATTGGTCAGG AATACCTCAA GCAAAACACA ACCCTAGGCA TACCAGCGCT GGTTCAGACT
 501 GAGGCTATAC ACGGCTTACT GGTGGGCAAT GCGACCATCT ACAACAGTCC TATTGGTCAA GCTTGCTCGT TTGACCCAGA ACTAATCCAC GATATGGCCG
 601 TGGCTATCGC AAAAGAGTCG CTAGCTTTGG GTATCAACCA GTTGTTCGCA CCACTCGCCG ACTTGGCACG CGAATTACGT TTTGGAAGAG TAGAAGAAAC
 701 ATATGGCGAA GACCCCTTTC TGGCAGGAGA GATGGCTTAC GAGTATGTCA AAGGCGTGCA AAGCCTCAAC GTGAGCGCTA CTGTGAAGCA CTTTGCAGGT
 801 TTCAGCGCCC CCGAACAAGG TCTGAACACC GGGCCAGTCC ACGGAGGCGA ACGAGAACTT CGAACAACAT GGCTTCCGCC TTTCCACCGC GCCATTATTG
 901 ATGCTGATGC TTGGAATATC ATGGGAGCAT ATCATTCGTA TGATGGTATA CCTTCAATTG CCGATGGCCA TCTTCAAGAG ACGATTTTGC GTGACGAATG
1001 GGGCTATAAA TATTGGCTGA CTTCAGACGC TGGGGCTACG GATCGCCTTT GCTGTGCCTT CAAACTCTGT CAATGCAAGA CTAAAGATAA GCCAATTGAC
1101 AGCGAGGCTG TGACACTCAT GGCTCTCCCG AACGGCAATG ACGTGGAGAT GGGTGGTGGA TCCTACAATT ACGCAAACAT CCCTCGCTTA GTCGAGGAAG
1201 GAAAGCTGGA TATCGAAATT GTCGACAGGG CAGTTAGTCG TCAACTGCGC GCGAAGTTCC GGCAAGGACT CTTCGAACGT CCATACCAAG GGCTCCCTCT
1301 GGATGAAATC GACTCTGCCA TTCACACCGA CGAATATGTT GCACTTGCAC GGAAGTTGGA GGCCGATTCG ATTGTCCTGC TCGAGAACAA GAACAACGTA
1401 CTGCCACTAT CGAAATCTGC CAATGTCGCC GTCATCGGAC CCATGGCCAA CATTACAAAC CTTGGCGACT ACGTAGTATA CCGTTCACAA TATAACCCCA
1501 CCAACGTCAC GCCTTTACAG GGAATTCAAA AGGCATCAAA TGGTACTGTG ACCTTTGCCC AAGGCTGCGA GCGCTGGTCA AACGACCAAT CCGGGTTTCC
1601 CGACGCAGTC GCAGCAGCCA AAGCAGCTGA TGTCGCAGTT GTCGTCGTCG GAACTTGGTC GCGTGACCAG CAAGAACTCT GGCAAGGTCT CAACGCCACA
1701 ACCGGCGAGC ACGTCGATGT CGCATCGCTC AATCTCGTCG GCGCCATGGG CCCCCTCGTC CAAGCAATCA TTCAAACCGG AAAACCCACA ATCGTCGTGT
1801 ACTCTTCGGG AAAACCCGTC ACAGAGCCTT GGATCTCAGA CAACGCAGCG GGGCTTTTGC AAATGTTTTA TCCTGGCGAG CAGGGTGGAA ATGGTCTAGC
1901 TGATGTGTTG TTTGGTGACG TGACACCTTC GGGCAAACTC TCTGTTTCAT TCCCTTATGA TGTTGGAACT TTGCCGATTT ACTACGACTA CCTGAACTCT
2001 GGTCGATCGA CTGATCCTGG TGCGATATTA CCAAACGGGA CGCTCAAATT TGGCCACCAA TACGTCCTTA ATACCCCGCA GCCGCTCTAT GAGTTCGGGT
2101 ACGGTCTCTC GTATGCCAAT TTTACTTATT CCGACGTCAA GCTTTCCAAG ACGGAAGCGA GCCCAAGCGA TACTGTGACT GTGACTGTCA GCGTCACGAA
2201 CTCTTCGCCT GTCGATGGTA AAGAGGTTGT GCAAGTTTAC GTTCAGGATG TCTTCGCAAG TGTTGTTGTG CCGAACAAGG AGCTTAAGGG GTTCAAGAAG
2301 GTGTTGGTCA AGGCAGGAGA GACTGTGGAT GTATCTATTG ATTTGGAGGT GTCGAAGTGG GGACTCTGGG ATAGGAAGAT GAAGTATGTG GTTGAGAAGG
2401 GGGATTTCAT CGTCCATGTG GGGTCTAGTT CGTTGGATTT GAGGGGGAAT GGGACGGTGA CTGTTGTCTA AGTGGAGCAT GGCCGAGAGT GAAAAGATGG
2501 AGCTATTTAC AGCACTTATG TAAAATAGAT TCGTATCTTG A
```

>*GH3-13*(jgi|Cocsa1|181585|estExt_Genemark1.C_7_t10380)

```
   1 CAGAGATCAG CCACGATGCA TTTCCTTTCT ACTGCCGTGG TCCTTGGTGC CTCGTTGATA TCAAAGTCAC ATGCAGCAAT CGGTCCTGAT TGTACCAATG
 101 GTCCCTTGAG TACCAATGCG ATTTGCGATG TCAATGCGCC TCCTCATGAG AGGGCAGCGG CTCTCGTCGC AGCTATGGAG CCGCAAGAAA AGCTGGATAA
 201 CCTCGTCAGT AAATCTAAGG GTGTGTCAAG ATTAGGTCTT CCAGCGTATA ACTGGTGGGG CGAAGCTCTA CACGGTGTAG CTGGAGCACC AGGAATCAAA
 301 TTCGTCGAAC CTTACAAAAA CGCTACTTCA TTTCCTATGC CAATCCTCAT GTCAGCGGCT TTTGATGATG ATCTTATTTT CAAAATTGCC AACATTATCG
 401 GGAACGAGGC TCGAGCGTTC GGAAACGGTG GAGTCGCTCC TGTGGACTAT TGGACTCCTG ACATCAATCC CGTTCGCGAT ATACGATGGG GCCGAGCCAG
 501 TGAATCCCCC GGAGAAGACA TTCGAAGAAT CAAAGGGTAC ACCAAAGCTC TTCTTGCTGG CCTCGAGGGT GACCAAGCTC AGAGGAAGAT TATTGCAACG
 601 TGCAAACACT ATGTGGGTTA TGACATGGAA GCTTGGGGAG GATACGATCG ACACAACTTC AGTGCAAAAA TCACCATGCA AGACCTCGCA GAGTACTATA
 701 TGCCACCATT TCAGCAATGT GCGCGTGATT CCAAGGTCGG GTCATTCATG TGCAGCTACA ATGCAGTCAA CGGCATTCCA ACATGCGCTG ACACCTACGT
 801 TCTTCAAACA ATCCTGAGGG ACCACTGGAA CTGGACGGAT AGCAATAACT ATATCACTAG TGATTGTGAA GCCGTTGCAG ATATCTCTGA GAACCACAAA
 901 TACGTCGAAA CCCTTGCGCA AGGCACCGCA CTTGCTTTTG CCAAGGGTAT GGATCTTAGT TGTGAATACA CTGGATCGTC AGATATCCCA GGAGCTTGGG
1001 CACAAGGCCT TCTAAATATT TCTGTTATCG ACAAAGCGTT GACTCGACAA TATGAAGGCC TAGTCCATGC CGGCTATTTT GATGGCGCGA AGGCAACTTA
1101 TGCAAACTTG AGTTATAAAG ACATTAACAC GCCCGAAGCA CGACAACTAT CTTTGCAAGT TACCTCTGAA GGTTTGGTCA TGCTAAAGAA TGACCACACA
1201 CTTCCATTGC CTCTCACGAA GGGATCAAAA GTGGCTATGA TAGGTTTCTG GGCCAACGAC TCTTCCAAAC TCCAGGGTAT CTACAGCGGT CCACCCCCTT
1301 ACCGGCACTC CCCAGTGTTC GCTGGTGAAC AAATGGGATT AGATATGGCC ATAGCCTGGG GCCCGATGAT CCAGAACTCA AGTGTGCCCG ACAACTGGAC
1401 TACCAACGCG CTCGATGCGG CTGAGAAATC CGACTATATC CTCTACTTTG GTGGTCAAGA CTGGACAGTG GCGCAGGAAG GCTACGATCG CACTACAATC
1501 AGTTTTCCTC AAGTGCAAAT TGACCTTCTT ACCAAACTAG CTAAACTCGG CAAGCCGCTT GTTGTCATCA CGCTTGGTGA TATGACTGAT CACTCCCCTC
1601 TTCTGTCCAT GGAAGGCGTC AACTCAATCA TCTGGGCGAA TTGGCCTGGC CAAGATGGCG GTCCAGCGAT ACTAAACGTC GTTTCCGGTG CGCATGCCCC
1701 TGCAGGTCGT TTGCCAATAA CGGAATACCC GGCAGATTAT GTCAAACTTT CTATGCTTGA CATGAACTTG CGCCCACACA CCGAAAGTCC CGGTCGTACT
1801 TATCGCTGGT TTAATGAGTC CGTTCAGCCA TTCGGTTTTG GTCTACATTA TACCACTTTT GAGGCCAGCT TTGCTAGTGA AGAAGGCCTC ACCTACGATA
1901 TCGAGGAAAT TCTGGATGGC TGCACACAAC AGTATAAGGA TTTGTGTGAG GTTGCACCAC TAGAGGTCAC CGTGGCAAAC AAGGGTAACC GAACATCGGA
2001 TTTTGTCGCT CTCGCTTTCA TCAAGGGTGA GGTTGGACCT AAGCCATACC CACTGAAGAC TCTGATTACG TATGGGAGGC TCAGAGACAT TCATGGGGGC
2101 GCGAAAAAGT CAGCGTCGCT TCCGCTTACA CTTGGAGAAT TGGCTAGAGT GGATCAATCA GGCAATACTG TTATCTATCC CGGCGAATAC ACGCTTCTCC
2201 TCGACGAGCC TACCCAGGCT GAGTTGAAGT TGACTATTAC AGGTGAAGAG ACGATTCTGG ACAAATGGCC CCAGCCGCCA AACGGAGGCA ATCGGACCGT
2301 GTTTTGAGCC CTTTGTGGTG GAATGCAGGG AGTTTGATTG TATGTAATGT GCGCGAACAA TAGACCAAGT TGTGAGACC
```

>*GH3-14*(jgi|Cocsa1|192755|estExt_fgenesh1_pg.C_130045)

```
   1 TCTTTCTGTC CACCGACCGA CCGACCGAAA CTCCATTTGC CAGCCGCGCC CAAGAGCCGG TGCGCCACGG AATCCTACGT CGCATTCACA GTCCGACGCG
```

```
 101 ACGCGCCGGA TATTATCATC ATGGCCAAGA TTGAGCAAAT GGATCCTGTA TGGGATGATC TCGACAGAAC AATGGGCCAG TTGTTCATGA TGGGCTTCGA
 201 TGGCACGTCG GTGACTCCCC ACATTCGTAC CCTTATCGAG GAGCATCACG TTGGCTGCAT CCTGCTGACT GCGAAAAACC TCAAATCCGC CGAGGACACA
 301 ACAAAGCTCA TTTTTGAATT GCAAAAGATT GCCCATGATG CCGGTCATCC TGTTCCACTG GCGATTGGTA TCGACCAGGA AAATGGCGGA GTCAACAGTC
 401 TCTTTGATGA CATCTACATT CGCCAGTACC CCAGCAACAT GGGCATGGCA GCGACCGGAT CCACTGACCT AGCATTTGAA GTCGCAAAAG CAACTGCACA
 501 AGAAATTGCC GCCTGTGGTA TCAACTGGAT ATTCGGCCCA TGTCTCGATG TACTCACTAA TGCACGTAAC CAGCCCTTGG GCGTCAGAAC AGCTGGAGAC
 601 GACCCTCAGG AAGTGTCTGC CTTTGGGTGT GCGTTCATGC AGGGCTACAA AGAGGGCGGA GTTGTAACAC TGGGAAAGCA TTTTCCATCT TACGGCAACC
 701 TAGAGTTCTT GGGGTCCACG CTTGATGTGC CCATCATCAC TGAGTCATTA GAGCAGCTAA GCTTAAGTGC CTTGATCCCA TTCAAGAAGG CGATTGAAAG
 801 AGGCCTTGAT TCTATGATGG TCGGTGGTTG CGCCATGTCT TCAGCTGGAC TGAATGCTAT GCACGCTTGT CTATCCGAGC AGGTCGTCGA TGATCTACTC
 901 CGCAAGGATC TGAAATTCGA CGGCGTAGTC GTCTCAGAAT GTCTTGAGAT GGACGCTCTC AGTCACAATA TTGGTGTTGG CGGTGGAACC GTCATGGCTG
1001 TCAACGCTGG TTGCGACGTG GTCCTTCTTT GTCGCGCCCT CTCGCTGCAA CTAGAAGGCC TAAAAGGCCT CAAGACTGGC ATTGAGAGTG AGATGGTTTC
1101 GAAAGAACGC ATCTTTAACT CGCTGAGGCG CGTGTTAACC ATGAAGAAGA AGTGCACGTC GTGGGAGAAG GCGTTGAACC CTCCGGGCAT CAACTATCTC
1201 GAAAAGCTCC AGCCTCTGCA TACTGCGCTC TCCACCAAAG CATACAATAG CTCCATCACA GTCGTACGCG ACAAGAACAG GCTTCTTCCG CTGACCAATA
1301 TTCTCGACAG CGAGGAGGAA CTCCTTCTAT TGACTCCTTT GGTGAAGCCC TTGGCTGCAT CGGCTGCATC TCGAGCCATA TCTGATGCTG CCAATGGCGC
1401 GGCTAGTCAC TCTCCAGGGC CAGCTTCCTG GGATCAGAAT TCGTCCGTTA TGAGTGGAGA ACGAGTTTTC AGGGAGCTGG GCCGCTCTCT GGCGAGACAG
1501 CGTAACGGGC GACTGTTGCA CACATCGTAC ACAGCAAACG GTGTTCGTCC AGTTCACGAG AACCTCATCA GCCGAGCAAG CGCGATTATT GTGCTCACTG
1601 CAGACGCCAA TAGAAACCTG TATCAGCATG GATTCACTAA ACACGTCTCT ATGATTTGCA ACATGCAATA CACGACTGGC GGAGAGAGAC GCGAGAAGCC
1701 TCTGATTGTG GTTGCTGTAA GTTCACCGTA CGACTTTGCC ATGGATAATA CGATTGGGAC GTATATCTGT ACCTATGATT TTACCGAGAC TGCGTTAAAT
1801 TCGCTGGTAC GAGTCTTGTA TGGAGAACTA TCACCTTCAG GAACACTGCC TGGTACGATT AGCAAGAGCC AAAAACTACA CCCGTCGAAA CAGCACTGGC
1901 TGGTTGAGAT CTTTAATGAA GAACGAGATG GCAGTGCGTT GGATACACTG ATTGCAGCCG TTGTAGAAAG CACCGCGCCA AACCAACGGT CTGAGCTTTC
2001 AAGCGCAACA TCAAGCTCGT TCATCCTACA CCACCCAGAG GTGGAGGAGT CCCATTTTGT CGTCCGTAAT AGCAGTACCC AGGCACTGTA TGGCTTCTGC
2101 TCAACATACT TTTTCAAAGC TACAGGAACT GGAGTGATTG GTGCGTTGTT TGTGGATCCA GCAAGGAGGA AGCTTTCGAT TGGACATTCG CTACATAACA
2201 GAGCGATACG AACTCTACTG CAGAAGGAAG GCATTAAGAG ATTTCAACTG GGATCTCGTC TTCCGAGTAT CTATCTCGGT ATCCCAACAG GACATGGCAA
2301 CGAGCGAAAG CGCCTACGGC CATGGTTTGC GCAACTGGGA TGGGGCGCGG CACTGTCACG GCCCGTGTGC AACATGGTCG CTCGCAACCT TCAGGCATGG
2401 AGTCCACCTG CAGGCATGGC CAAGAGCCTG GCAAGCGCAG GAGCACAGTT TGACCTTGTC TATGGGTGGG ACTACGCTGG ACCGGTGCTC GACCATATCA
2501 AAACCAGTAA CCGACAAGGG CTGGCAGAGG TGTACAAGCT CGCACTCACT GATCCGACGG CGTGTGGAAT TATTCGGGCA AAGCGGCCGG AAGATGGTGC
2601 GCTGGTGGGC ACGGTTGTGC TGTACAACAG TCACTCGCGT CTAGCCGAGT ACATTCCTTC GCTCAAGGAT CTCAACGAGT CGGCTGGTGG CATTTCATCG
2701 CCGGTGATAT CACCTGGTGT TGGAGAGTAC TCGACACTAC TGCAGGGATT GATACTTCTT GGGATGCGAC AGATCAAGCA GCTAGGATGC AACGCGTGCT
2801 TGCTCGACTA TATGGATGGA GATGGCAATT TTGACGGGTT CTCAGCAATG GGGTTCGATG TCATGCACAA TTTTGAGGAA GTGAGCTGTG ATGCAGCAAC
2901 ATGGACCATG ATACCACCAG CTTAGGTTGG GTAGGGATAT GTTTTTTCTT CTGTTTGAGG CGCAATGAAG GAACCCTTTG GCTGGCTGGG ACTTGGATGA
3001 GATGAAAGGG CATGGTAGGT GTTTATACAC ACTTAATACA TATTCTCTCT TATTGGCTAC TACAGATGTG GAGAAGAAAG AAGGATGTGT TTGGCATGGA
3101 GGATTGCATC ATGGTGGCTG AGAACTAGTC GGATGTCGTG GCATGGGTGC ATAGGATGGG CTGCGGGCTT TGTGGAGTGG GAGACCGGAT GGTTTCCCTA
3201 ATAGGTAATT TGGCGCTGAC GGGCGCTGCA TGCCGCGTAC CGCGTACCGC TTGGCTTATA AGTCGCGTCA GGGTTCAAGT GAACAGGTAA ATACAGAGGC
3301 TGGGATGGCT GGATTTCAGA GAGGATTACT CGAGAGCCAC GGGCGGGGTG TAGAGATGGT GCGGAGGGGG GCGCATTGAG GATGTCGGAT TTGGTCTTGG
3401 CTGGGAGCCG TAA
```

>*GH3-15*(jgi|Cocsa1|210264|CE5610_1756)

```
   1 CAGGTGCGAT GGAAGGCAAA ACGTTGGCGC CAAGTGCAGC CATACGATGC TTGCTGGACT ACCTCCCAAG CCAATAGTGT AGCCGAGGAT TCCCCAAGAA
 101 GAAGACACCG ACGGTGGTCT CTCGGCTCTT GTACACATGA TAGACAAGGA AGACGGAGTA TTGCATGGCG AACAATGTGA GGCTGGAATA CAAGTATTGG
 201 GTCATAGTCT GCCCAACGGA TAGTCAATTG GATCTCTCGT CACTACCCAC AAAGTGCCTC GTTCCTCACT ACCGGCAAGA TGGTCGCTCG CAATCGCAGT
 301 CAGCGCAAGC TGAACACCTT CACCACGCTG TTCTTCGCTG CAACAGCCAT GTCATCGGCA ATTCGCACAC CATCGTTTGG CAAATCAACA TATCCTCGTA
 401 TGACATCACG TTCTACTGAG ATGGACTCTG CACCATATCG GGACTCGACA CTTTCAGTTG ATGAGCGAGT CGAGGATCTT GTACAACGGA TGACCGTTGA
 501 GGAAAAAGCA GGCCAGCTTT TCCACAACAT CATCAACCAA GGCCCAAATG GAACCCTGCT CAATAACACG AGGGAATCTG TGGAGGGCAA GCTCATGACC
 601 CACTTCAACC TTCACGGGCC GATTGCAGAT GTACGCGCCA CCGTGCAATG GTACAACAAC CTGCAGCAAA TGGCGCTAGA CACAAGACTG GGCATTCCAA
 701 TCACAGTCTC CTCGGATCCG CGTCACGCAT TCACCAACTC TGAAGGCAGT CAGATTGCAG CAACAAAATT CTCACAATGG CCCCAGACGC TTGGCCTAGC
 801 TGCGATCAGA GATGCTGAGC TCATCCGCAC TTTCGCTGAT ATTGCTCGAC AAGAGTACAA TGCTGTTGGC ATTCGGTCTG CCCTACACCC TCAAATCGAT
 901 GTCGCTACTG AACCCCGCTG GGCCCGCATT GGTGGCACCA TGGGCGAGAA TGCCACTCTG ACTGCTGAAC TGACTGTTGC ATACATTGAG GGATTCACTG
1001 GTCCTAACGG TTTTGGACAC GACTCAGTCA CCACAGTCTC AAAGCATTTC CCCGGCAGTG GTCCAGTCGA GGGTGGCGAG GACAGTCACT TCACCTACGG
1101 CAAGAATGCC ACATACCCTG GCAACAACTT TGAACACCAT CTTATCCCAT TCAAGGCTGC TATTGCTGCC GGAACACGAC AGATGATGCC TTACTACTCT
1201 CGCCCAATGG GAACCAAGTA TGAGGAGGTC GCTGCTGGTA TGAACAAGGG AATCGTCACT GATCTTCTGC GCGGCGAGCT CGGCTTCGAC GGCATCGTAG
1301 TCAGCGACTG GGGTCTCGTC ACTGACGGCA TAATCTTGGG CCAAGACATG CCCGCTCGAG CCTGGGGAGC TGAGAACCTC ACCGAGCTCG AACGCACTGA
1401 GAAGATCCTC AATGCAGGAA CCGATCAGCT CGGTGGCGAG GACCGCACCG ACCTCATCCT CGAGCTCGTA GAGAAGGGCA TCATCAGCGA ATCCCGCATC
1501 GATGCCTCAG TCCGCCGTCT CCTCCGCGAA AAGTTCCTCC TCGGTCTCTT CGACAACCCC TTTCTCGACG CCGACGCTGC CGTAGCCGTC GTCGGCAAAG
1601 ACGAATGGCG CGCCATCGGT TTCGAAGCCC AAAAGCGCTC CTTCACCCTC CTCACAAACA AAGACTCCTT CCTCCCCCTC TCTGCTTCAG ATTGTGGAAA
1701 GTCAAAATTC TACATCGAGG GCTTCAACAG CACGTTCCTT ACCTCGCGCA ACTTTGAAGT CGTAGCCACG CCCGAAGAGG CAGACTATGC TTTCCTGCGC
1801 CTCGTAGCGC CGTATGAGCC GCGTCCCGGC GGTTTCGAGG CGAGCTACCA CTCTGGTTCG CTTGAATACA ACGCTACGGA ACGTGCGCGC CAGGCAGCTA
1901 TCTACGCTAC CGTACCTACC GTTGTCGACA TCTTCCTCGA CCGCCCCGGT GCGTTCCCCG AGGTCGCAGA GCAAGCAGAG GCCCTCATGG TCAACTTTGG
2001 CGCTAGCGAG GATGCGTTCC TGGATGTTGT GTTTGGTGTT GATGGAAGCG GTCCGGAGGG TCTGCTGCCG TTTGATCTTC CTAGGAGTGA CGAGGCGGCT
2101 AAAACGCAGA AGGAGGATGT GCCGTTTGAT ACTTTGGACC CGGCGTTCAA GTTCGGCTAT GGGTTGAGGT ATGAATAGTA ATCCCAGGGA CAATGTAGGA
2201 TTATGAAGGG GATTTATGGG TGATTGTATT TAAGAACATG ATAATGAATA TTTCACATGA ATAGAAGTTT GCATAATTTG ATGAAC
```

GH6 genes

>*GH6-1*(jgi|Cocsa1|152794|estExt_Genewise1Plus.C_17_t10207)

```
   1 ACGCACCTCA CCACATTCCT TCTTGGTTCT CAAAGATGCT CTCCAACGTC TTCCTTACCG CTGCGCTCGC AGCCGGCCTG GCTCAGGCTC TGCCCCAGGC
 101 CACGCCTACT CCGACCCCTG CGCCCTCTGG CAACCCTTTC GCGGGCAAGA ACTTCTACGC CAACCCATAC TACTCGTCTG AAGTTCACAC CCTGGCCATG
 201 CCCTCACTTC CAGCCTCGCT GAAGCCTGCT GCTACCGCCG TGGCCAAGGT CGGATCATTC GTGTGGATGG ACACTATGGC CAAGGTTCCT CTTATGGATA
 301 CCTACCTCGC AGACATCAAG GCCAAGAACG CTGCTGGCGC AAACCTCATG GGTACTTTTG TTGTCTACGA CCTTCCTGAC CGTGACTGCG CCGCTCTGGC
 401 CTCCAACGGT GAACTCAAGA TTGACGACGG TGGCGTCGAG AAGTACAAGA CCCAGTACAT TGACAAGATT GCCGCCATCA TCAAGAAGTA CCCCGACGTC
 501 AAGATCAACC TTGCCATTGA GCCCGATTCC CTTGCCAACA TGGTCACCAA CATGGGTGTG CAAAAGTGCT CGCGCGCCGC CCCATACTAC AAGGAGCTCA
 601 CTGCCTACGC CCTCAAGACG CTCAACTTCA ACAACGTCGA CATGTACATG GACGGTGGCC ACGCCGGTTG GCTCGGCTGG GACGCCAACA TTGGTCCCAC
 701 CGCAAAGCTC TTCGCAGAGG TTTACAAGGC TGCTGGCTCT CCTCGTGGTG TCCGTGGTAT TGTCACCAAC GTCAGCAACT ACAACGCTCT CCGCATCTCC
 801 TCCTGCCCGT CCATCACCCA AGGAAACAAG AACTGCGACG AGGAGCGCTA CATCAACGCT TTAGCTCCTC TTCTCAAGAA CGAGGGTTTC CCTGCTCACT
 901 TCATCGTCGA CCAGGGCCGC TCCGGAAAGG TGCCCACTAA CCAGCAGGAG TGGGGTGACT GGTGCAACGT CTCAGGTGCT GGCTTCGGTA CCCGTCCCAC
1001 CACCAACACT GGCAATGCGC TCATTGATGC TATCGTCTGG GTCAAGCCCG GTGGCGAGTC TGACGGTACC TCCAACACCA GCGCTGCCCG CTACGATGCC
1101 CACTGCGGCA GGAACAGCGC CTTCAAGCCC GCTCCTGAGG CCGGAACCTG GTTCCAGGCT TACTTTGAGA TGCTTCTCAA GAACGCTAAC CCTGCTCTTG
1201 CTTAAGCGTC TGGTTCTTTT GGATAAGCTT GGGTAGATTG TTAGAAGGGA AAATTAGTCT GCGCGTAGTC TTCACTGCAG ATTTGGTGGA TTGTAAATAT
1301 GGCTTTGGAC CTAGAATAGG CAACGTTTGA TGTTGTAGTT CGTGTAAATA TTATACCTTT TGGAGCTAGT GCTGGTATCT CTGTAAACAC GGTGAT
```

>*GH6-2*(jgi|Cocsa1|188313|estExt_fgenesh1_pg.C_3_t20040)

```
   1 ATGAAAGGTC TTCTCTCCGC AACCGTGGCT TCGGCCATCT CTGGCGCTCT TGCGTTTCCT TCTCCTGCTG AGCGCCGCGA CATCGCTGCC CGCCAGGCTG
 101 CAGTATGCTC TTCTCCCGTC AAACTATCTG GAAACCCGTT TTCCAACCGC AGCCTCGCTG TCAACAGTTT TTATGCTGCT GAAGTCAAAG AAGCTGTTAC
 201 CAAAATCAGT GACTCGGCAC TTGCAGCAAA AGCGGCCAAG GTAGCTGAAA TTGGAAGTTA TTTCTGGATT GATACGCGTG ACAAGATCAA GATGCTTGAC
 301 GACATCATCA AGGGTACCCC ATGCAACCAG ATTCGCGGAC TAATCATTTA CGATTTGCCT GGTCGCGACT GCGCTGCCAA AGCATCCAAC GGTGAACTTG
 401 CCGTTGGCGA AATCAACATT TACAAGACGC AGTACATTGA TCCCATCGTT GCAATCATCA AGAAGTACCC AAACATAGCC TTCGCTCTGA TCATTGAGCC
 501 CGACTCTCTA CCCAACTTGG TCACCAATAG CAACTTGGCA GCTTGTCAGG CCTCTGCTTC CGGATACCGC GAGGGAGTTG CGTATGCGCT TAAGCAGCTA
 601 AATCTCCCCA ACGTAAACAT GTACATAGAT GCTGGTCACG GAGGCTGGCT CGGCTGGGAC GCCAATATCA AGCCGGGCGC GAAGGAGCTT GCATCGGTCT
 701 ACAAAAACGC TGGCAGCCCC AAGGCAGTCC GCGGTATCGC AACCAACGTT GCCGGCTGGA ACTCGTTTGA CCAGGAGCCT GGAGAATTTG CCGGTTCCTC
 801 CGACGGCCAG TACAACAAGG CGCAAAACGA GAAGAAGTAC GTTTCGCTCA TCTCGCCCGA ACTTGTCGCC GCAGGTATGC CCGGCCAGGC CATCGTCGAC
 901 ACAGGCCGCA ATGGTGTCAC GGGTCTCCGC AAGGAATGGG GTGACTGGTG CAATGTCATC GGGGCTGGAT TCGGTCGTCG CCCTACGTCT GACACGGGAT
1001 CTTCGCTCGT CGATGCTTTT GTGTGGGCCA AGCCTGGCGG CGAGAGCGAT GGTACTAGCG ATACGTCCGC GAAGCGCTAT GATTCATTCT GTGGCAAGGA
1101 CGATGCCTTC AAGCCCAGCC CTGAGGCTGG ATCCTGGAAT CAGGCCTACT TTGAGGCACT TGTCAAGAAC GCTAACCCGC CTCTATAG
```

>*GH6-3*(jgi|Cocsa1|272836|CE68182_498)

```
   1 AAACAGGAGT CCTCCATCTG TTCTCAAGCT CTCATCAATC ATGGTTCAAG TCACACAGTT CGTTTTGGCC ACTGCCGCCC TTGCTGGCGC CGCAGCTGCA
 101 TTCCCCAAAC CAGAGCCCAG AAATACCACT GTCAACCCAT GGATTGGCAA GGACCGCTAT GTTGTCGAAA GCTACGGCAA GAAGCTTGAC CAAACCATTT
 201 CTTCCTTCCT AGCAAAGAAT GACCCCCTCA ATGCAGCACG AACCCGCACT GTGGCCAAAA AGACTTCCAC CTTCGTGTGG GTTACATCGC GCGCTGGACT
 301 TAGCCAGATC CCCGAAGCCA TCCAACAAGC CCGCCGTCAA CGAAAGGGCC GAAAGAGGAT GATTGTCGGC CTTGTCCTTT ACAATCTCCC CGACCGTGAC
 401 TGCTCTGCTG GAGAGTCTGC CGGAGAGCTC AGCTCCGCCA AAGATGGCCT GAACATCTAC AAGCATGAAT TCGTCGACAA GTACGCACAG CTCGTATCTG
 501 AGGCCAAGGA TCTCGACTTT GCCATTGTCC TCGAGCCCGA CTCGCTCGGC AACGCCGTCA CCAACCAAGG TGTTCCCTTC TGCGCCAACG CCACCCCCAT
 601 CTACGAGCAA GGTATCGCTT ACGCTATTGC CAAGCTCCAG TTCCCCAATG TATCCCTATA CATGGATGCT GCCCACGGAG GCTGGCTTGG ATGGGCCGAC
 701 AACCTGAAAC CCACAGCCCA GATTTTTGCC AGAGTTGTCG AAGCGGCTAA GAAGATCAAC CCAGCGGCCA AGATCCGTGG TTACTCTACC AACGTATCCA
```

```
 801 ACTACAACCC TTTCAACGCC AAGGTCCGCG AGAACTACAC TGAATGGTCT CCATCTTGGG ATGAGAGCCA CTACGCTACC TCTCTTGGAG CGGCAATGGC
 901 AGCTGAGGGC ATGCCAACTA ACTTCATCAT CGACCAGGGC CGTGTTGCCC TCCCCGGTGC TCGCAAGGAG TGGGGCGAGT GGTGCAACGT TTCGCCCTCT
1001 GGCTTTGGAA TGCGCCCTGG AGCTGCACCA AACAACACCA ACGTTGACTC GATTGTCTGG ATCAAGCCCG GTGGTGAGAG TGACGGAGCC TGCGGTCTGG
1101 CTGGTGCGCC TGTTGCTGGT GCTTGGTTCG ACGACTACGT TCAGATGCTC GTCAAGAACG CCGACCCTCC TCTTGAGCCT ACCTACTAGA AAGGATCCCG
1201 CGGCTGAGGT TGAGGAAACT GTCGCCCGGC CTTAACTTTC GGTGTATTCA TGTAGATTTT GAACTTTCAA CTGTACATAT TGTCATGAGA ATACATAAA
```

GH7 genes

>*GH7-1*(jgi|Cocsa1|32576|fgenesh1_kg.1_#_739_#_Locus8216v1rpkm12.25)

```
   1 CAGACTCTTT ACTCAAGCCA GTCAACATGT ACAGGACACT CGCCTTCGCC TCTCTTTCGC TCTATGGAGC CGCCCGCGCT CAGCAGGTTG GCACCAGCAC
 101 TGCTGAGAAC CACCCCAAGC TGACCTGGCA AACCTGCACG GGTACCGGTG GCACCAACTG CTCCAACAAG TCTGGTTCTG TTGTGCTCGA CTCTAACTGG
 201 CGATGGGCCC ACAACGTTGG CGGATACACC AACTGTTACA CTGGCAACAG CTGGAGCACC CAGTACTGCG CTGACGGTGA CTCTTGCACC AAGAACTGCG
 301 CCATTGACGG TGCTGACTAC TCTGGCACTT ATGGTATCAC TACCAGCAAC AACGCCCTCT CCCTCAAGTT CGTCACCAAG GGCTCCTTCT CTAGCAACAT
 401 TGGTTCGCGT ACCTACCTCA TGGAGTCGGA CACCAAGTAC CAGATGTTCA ACCTCATTAA CAAGGAATTC ACCTTTGATG TCGATGTCTC TAAGCTTCCT
 501 TGCGGTCTGA ACGGTGCTCT CTACTTCGTC GAGATGGCCG CCGACGGTGG CATCGGCAAG GGCAACAACA AGGCTGGTGC CAAGTACGGA ACTGGATACT
 601 GCGACTCCCA GTGCCCTCAC GACATCAAGT TCATCAACGG AAAGGCCAAC GTCGAGGGCT GGAACCCCTC TGCTGCCGAC CCCAACGGTG GCGCCGGTAA
 701 GATCGGTGCC TGCTGCCCTG AAATGGATAT CTGGGAGGCC AACTCCATCT CGACTGCCTA CACCCCTCAC CCTTGCAAGG GCGTAGGTCT CCAGGAGTGC
 801 TCCGACGCCG CGAGCTGCGG TGACGGCTCC AACCGCTACG ACGGCCGGTG CGACAAGGAC GGATGCGACT TCAACAGCTA CCGCATGGGC GTCAAGGACT
 901 TCTACGGCCC GGGAGCCACC CTCGACACCA CCAAGAAGAT GACCGTCATC ACCCAGTTCC TCGGCTCCGG CTCCAAGCTC TCCGAGATCA AGCGTTTCTA
1001 TGTACAGAAC GGCAAGGTCT ACAAGAACTC CCAGTCGGCC GTTGCAGGCG TCACCGGCAA CTCCATCACC GAGAGCTTCT GTACCGCACA GAAGAAGGCC
1101 TTTGGCGACA CATCGTCATT CGCCACCCTC GGCGGTCTCA ACGAGATGGG TGCGTCGCTT GCTCGCGGCC ACGTCCTCGT CATGTCGCTC TGGGACGACC
1201 ACGCGGTCAA CATGCTCTGG CTCGACTCTA CCTACCCCAC CGATGCTGAC CCATCCAAGC CCGGTGCCGC CCGTGGTTCC TGCCCTACTA CCTCTGGTAA
1301 GCCAGAGGAT GTCGAGAAGA ACTCGCCCGA TGCTACGGTT GTCTTCTCCA ACATCAAGTT TGGTCCTATT GGTTCCACTT TTGCTCAGCC CGCATAAGCG
1401 CTTTCTCTTT CGCTGAGCGC GCCTGTTGTG GATGGTAGAC TCTGTACTTT GTCTTCTTGA TCTCAATGGG AGAGGTGTAG GTAGACCTTC TTCTTCTTCT
1501 TCTTCCTGTT CGATACTTTC GCTTCGACAT CTCGGAAATT GTATATACTT TTTGTTTGTT GAGTAGTGGA AAGTGGGGTG GAGGGGGAAA CTACTAGAAA
1601 TGTGTGCATA CAAAGTAGAC AAATGAAAAT TTGCATTCAG TCCA
```

>*GH7-2*(jgi|Cocsa1|39085|fgenesh1_kg.11_#_385_#_Locus3464v1rpkm31.79)

```
   1 GCCACCCACC ACTCCTTCAA CAGCATCAGC TTCTCCTCAA TTAGCCCACA ATGGCACGTT ATGCACTTCT TGCGGCAGCC CTTTTGGGTT TCACAGTCGC
 101 CCAAACTCCA GGAACCACTC CTGAAGTGCA CCCCAAGCTC ACCACATGGA AGTGCACCAA GCGTGGTGGA TGCAAGGCGC AGAATACTGC CATTGTCACA
 201 GACTCGGCCG CCCACCCCAT TCACCAGAAG AACAACTCGA CCCTAGGCTG TGGATCGTGG GGTAACGGTC CCGACCCCAC AGCATGCCCT ACAAAGGAGG
 301 CTTGCGCCAA GAACTGTATC ATCGAGGGCA TCTCCGACTA TTCGACTGTA GGCGTCTTCA CCAAGGGCGC CGACCTCCGC ATGGACATGT ACAACCCTGC
 401 TGGCAACTTT GTCAGCCCTA GGGTCTACCT GCTATCTAAG GACGAGAAGA ACTACGAGAT GCTTCAGCTC ACTGGTAACG AGCTGACCTT TGATGTCGAC
 501 ATGTCTAAGT TGCCATGTGG CATGAACGGT GCCCTGTACC TGAGTGAGAT GAGAGCTGAT GGTGGTCGCT CTAAGCTCAA CCCCAAGGGC GCTGCCTACG
 601 GTACGGGCTA CTGTGACGCA CAGTGCTTCG TGACCCCCTG GATGAACGGT GAAGGAAATG TCCAAAAGCA AGGTGTCTGC TGCAATGAAA TGGACATCTG
 701 GGAAGCCAAC AGCCGCTCCA CCTCAATCGC ACCGCACACA TGCGGCAAGC CAAGCATCTT TGGCTGCACC GGCGACGAGT GCGGCAAGAC GGGTCTCTGC
 801 GATAAGAATG GCTGTGTTGA TAACCCGTAC AAGACCCGCG GCGACAAGGC CTACTACGGC CTTAACCTCA AGGTCGACAC GAACCGCCCC TTCACTGTCG
 901 TTACGCAATT CCCTGCTAAG AACGGCGTGC TACAAGCCAT CGTGCGCAAA TACATCCAAG ATGGCGTCGT TATGGACAAC GCCGTCAAGA ACATCACCAT
1001 GGACCAGGCG TACTGCTCTG CACAGGGCGG CGCGGAAATG TACAACCAGC TCGGAGGCCA CAAGGGCATG GGTGATGCCA TGTCCCGCGG CATGGTGCTC
1101 GCCATGAGCA TCTGGTGGGA CGAGGGCGGT GCCATGCAGT GGCTTGACAG CGGTGCCTCG GGCCCATGCA ACGCTACCGA GGGCTTTCCC GCTGAGATCG
1201 TCAAGAAGGT TAAGAACCCG ACTGTGACTT TCAGCAAGAT CAAGTGGGGC GAGATTGGCT CGACTTTCTC AACAAAGAAG GCGACGGAGA AGCGATGGGA
1301 GGCCTAAAAC GCTTTCGTTG CTTAGAGAGC TTGGAAAATC ATTCTGTATG TATATATTGA GAGTTCAATC GATTGAATGA AAGAGTACAT AATACCAA
```

>*GH7-3*(jgi|Cocsa1|201219|estExt_fgenesh1_pm.C_100198)

```
   1 CCCCGTCAGA CGAGACCGTG CTCCCACGAG GCGGTATAAG TATGGTGTTG ATTCAATCGA AACAGTTCCC CAATCGCTTC TTTTCTTGAT CCAGCGGAAC
 101 CACTCCCTCT TGAAACGCTT AAACATGTCA CTTTCTTCAG TCGTCGCGGC AACCCTGTTG GGCTTGGCCA CTGCCCAAAT AGTGGGAACT ATACCAGAAG
 201 TACACCCAAA GTTGACGACG TGGAAATGCA CAAAGGCTGG TGGATGCAAG GCGCTAGACA CAGCCATTGT CGTCGACTCA CTACGCCACA ACATTCACCT
 301 GAAGAACGAC ACGAGTGTGA GCTGCGGTGA TGAATACACT GCGCTCAACA CGGCGCTCTG CCCAGACAAG AAGACTTGCG CCCAGAATTG CGTAATTGAT
 401 GGCATCGAGG ATTACTCCAC TCAAGCAATT TTCACGGACA ACGACAAGCT TCGCCTAGAC ATGTATAATC CAAAGGGCGA ATATATGAGC CCTAGGGTGT
 501 ATTTGCTAGC CGAGGATAAA CAAAACTACG AGATGCTGCA ATTGACGGGC AATGAGCTAT CCTTTGACGT CGACATGTCG AAGCTACCCT GTGGCATGAA
 601 CAGTGCTCTG TACCTTAGCG AGATGGAAGC TGATGGTGGG CGGTCCGAAT ACAATACCGG AGGTGCGCCT CAGGGTAGCG GGTACTGCGA TGCCCAGTGT
 701 TTTGTCAAAC CATTTTTGAA CGGCGAGGCC AACGCCAATG GCGAGGGCGC CTGCTGCAAC GAAATGGATA TCTGGGAGGC CAACAGTTAT GCCAACCAGA
 801 TCGCCCCCCA CACCTGCGCC AAGCCAGGTA TCTTTGCATG CACGGGCAAT GACTGCGGCA GTGCTGGACT CTGCGACAAG GCCGGATGCG GTGACAACCC
 901 GTATAAGGAC CGTAACGACA AGGAATATTA CGGACCGGGT CTCAAGGTCG ATACCAACAG GCCATTCACC GTTGTCACCC AGTTCCCCGC CAAGGACGGC
1001 GTGCTCCAGG CCATTGTCCG CAAGTACGTC CAAGATGGTG TGGTCATTGA AAACGCCATG CGCAACCTCA CTGCTGAAGA AGCAGTCATG GACCAGGCGT
1101 GGTGCGATAG GCAGGGAAAA ACCTCTGCCT ACAACAGGCT CGGCGGCCAC AAGACCATGG GTGAGGCTCT GGAACGCGGT ATGGTTCTAG CATTGAGCAT
1201 CTGGTGGGAC AAGAGCGGAG GCATGCAGTG GCTTGACAGC GGATCCAGCG GGCCTTGCAA TGCCACCGAG GGCTTCCCAG ATGTCATCCA GTCGAAGGTC
1301 AAGAACCCCA CTGTTACCTT TAGCCAGGTC AAGTGGGGTG AGATTGACTC GACGTATGGA GCTTCGAATG CGACAACCAA GTACTGGAAG GCTTAGAGCG
1401 TGGTGGCTTT GTATATATTA GATGATAGAT GTGTATATGT ACGTTTTCAA ATAGAGACGC GTGGG
```

>*GH7-4*(jgi|Cocsa1|160447|gm1.5168_g)

```
   1 ATGGGGACCA CACCTGAAGT GCATCCAAAG CTATCAACCT GGAAATGCAC AAACGCTGGC GGATGCAAGA TGCTAGACAC TTCGATTGTT ATCGATTCTT
 101 TGAGACACAA GGTCCACCCA AAATCTGATC CGAGTGCGAC TTCGAGCTGC GGCGATCGAA ATAACCCGCT CAACCTAACG GTGTGTCCGG ACAAAGCGAC
 201 ATGCGCCCAA AATTGCGTCA TCGAAGGCAT TCAAGACTAT TCGACACAAG CAGTCTTTAC TGATGGTGGA AAGCTCCGTC TAGATATGTT CAACCCAAGC
 301 GGCGAGTATA TGAGCCCTAG AGTGTACCTA CTAGGCGAAG ACAAAGAAAA TTACGAGATG TTGCAATTGA ATGGCCAAGA GTTTTCATTC GACGTTGACA
 401 TGTCAAAGCT CCCATGTGGT ATGAACAGCG CCTTGTATCT TAATGAGATG AAGGCCGATG GTGGACGCTC GAAATTGAAC CCTACTGGTG CTAATATGGG
 501 GTGGGGATAC TGTGATGCGC AGTGTTCTGT CAAGCCATTC CTAGATGGTG AAGCTAACAT CGATAAGGAA GGCGCATGCT GCAACGAAAT GGACATTTGG
 601 GAGGCTAACG GTCGAGCAAA CCAGATTGCG CCCCATGTGT GCGCCAAAGA AGGTGTGATT AGGTGCACTG GCGACGATTG CGGTGTAGCT GGTGTTTGTG
 701 GTAAGACTGG CTGTGGAGAC AATGCTTACA ACTTCCGAAA CTCCAAGGAT TTCTATGGCC CTGGTCTCAA AGTTGATACA ACTAGGCCTT TTACTGTTGT
 801 CACTCAGTTC CCAGAGAAGT ATGGTGTTCT CCAAGCTATT ATTCGAAAAT ATGTTCAGGA TGGTGTCGTT GTCGAGAACG CTATGTCAAA CGTTACTATG
 901 GATCAAGCCT GGTGCTCGGC TCAGGCTCGT GCCAGGGAAT ACAACAAATT TGGCGGCCAC AAGACTATGG GTGACGCTCT TGCTCGTGGC ATGATCCTCA
1001 CATTCAGTTT ATGGTGGGAC AAGAACGGAG GGATGCAGTG GCTTGATGGC GGGCTAAATG GACCTTGCAG CCCTGCTGAG GATTTTCCTG ATGCTATTCA
1101 GCAGAAGGTG AAGAACCCTA CTGTCACCTT TAGCCAAATT AAATGGGGTG AAATAGGCTC GACATTTGGA ACGAACCAGA CTTTACAGTC GTGGGCGACG
1201 TAG
```

>*GH7-5*(jgi|Cocsa1|343966|MIX3290_34_78)

```
   1 CTTCAGTCTA TCTCTCGAAT ATCACACTCA ACACAAACAT CAGCATCTTC AGCATGTACC GGACTCTGGT AGTTACCGCT TCTTTTCTCC ACGCAGCTCG
 101 CGGTCAGCTG GTGGGAACGC AGAAGGAGGA AGTGCACCCT AAGTTGTCAT GGCAATCTTG CACTGCCCCA GGAAGTTGTA CCACCCAAAA CGGCGAAGTA
 201 GTCGTTGATG CCAACTGGCG TTGGCTTCAT ACCAAGGAGG GTTACACGAA CTGTTATACT GGCAACACTT GGGACACGAA ATATTGCCCC GACAACAAGT
 301 CGTGCGCGGC CAACTGCGCC ATCGATGGTG CGGATTATTC AGGCACCTAC GGTATTACTA CCAGCGGCAA TGCCTTGAAG CTTAACTTCG TCACCAAGGG
 401 CTCGTATTCC ACAAACATTG GGTCACGTAA CTACCTGATG AAGGATGAAA AAACCTACCA GATGTTCAAA TTCACGGGCA ATCAAGAGTT CACGTTCGAC
 501 GTAGATGTCT CGAAGCTACC TTGCGGTTTC AATGGCGCAC TGTACTTTGT CTCTATGGAT GCAGACGGTG GTCTCAGCAA GTATCCAGGT AACAAAGCCG
 601 GTGCTAAGTA CGGAACTGGT TATTGCGACG CCCAGGCTCC AAGGGATCTT AAGTTCATTA ATGGCGAGGC CAATGTTGAA GGCTGGAAAC CGTCGTCGAA
 701 TGACCAGAAT GCCGGTGTCG GCGGTCATGG TTCCAGCTGT GCAGAGATGG ACATTTGGGA GGCTAACTCG GTCAGCACAG CAGTCACTCC ACACTCTTGC
 801 AGCACAATTG AACAAACCCG CTGCGACGGC GATTCATGTG GAGGTACGTA CTCAGCCACA CGCTATGCTG GAGTGTGCGA CGCGGATGGT TGCGACTTTA
 901 ACAGCTACCG CATGGGTGTC AAAGACTTTT ATGGAAAAGG CAAGACAGTG GACACATCTC AAAAGTTCAC AGTCGTCACA CAATTCGTTG GAACAGGTGA
1001 CAACTTGGAG ATCAGGCGCT TCTACGTGCA AGGCGGCAAA GTTATTCCTC AACCCTCATC CAAGATTCCA GGAATCGACG GCAACGCCAT CAACTCCAAG
1101 TTTTGCGACC AGCAGAAGTC TGTCTTCGGC GACCCAACTG ACTTCCAGAA GAAGGGCGGC ATGGGAGGCA TGGCCAAGGC TCTTGCTAAT GGTATGGTTC
1201 TTGTCATGAG CGTGTGGGAT GACCACAACA GCAATATGCT GTGGCTCGAT TCTACATACC CCACCGACAA GGACCCAGAA AAGGATCTTG GTGTTGCTCG
1301 AGGAGAATGT GCGACGACCA GCGGTGTACC AAAGGATCTT GAGGCCAATA ACGCAGATGC TACAGTCACT TACAGTAACA TCAAGTTCGG TCCTTTGAAC
1401 TCGACCTTCA GTGCACAATA G
```

>*GH7-6*(jgi|Cocsa1|351972|MIX11296_12_37)

```
   1 CAGTCACTAC CGCTTCCAAG CGCTCAGTCT CGACGAACAA TCTTTCAGCT ATGTCTTTTG TTTCTGTCAC CGCGGCATTC CTGGCCCTCT CCGCTCATAG
 101 CGTGTCCGGA TGGGTCATTG GAACCCAACA AACAGAGACG CATCCCAAGA TCACGTGGCA GCGTTGCACT GGCACAGGCG GAACGTCCTG CAGTAACGTG
 201 AATGGAGAAA TCGTCATTGA TGCCAACTGG CGTTGGCTCC ATTCTACCGG GGGCTACACG AACTGCTACA CTGGCAACGA GTGGAACAAG ACCGCTTGTC
 301 CTGACAATGC AGCATGCACC AAGAACTGCG CGATTGAAGG CTCCGATTAC CGTGGCACCT ATGGTATCTC GACGTCTGGA AACTCCCTTA GTCTCAAGTT
 401 CATTACAAAG GGCCAGTACT CGACAAACAT TGGGTCCCGT ACTTATCTGA TGAAGGATAC AAACAACTAT GAGATGTTCC AACTTATCGG CAACGAATTC
 501 ACTTTCGATG TCGATCTCTC ACAATTGCCA TGCGGCTTAA ATGGTGCTCT GTACTTTGTC TCTATGCCTC AGAAGGGACA GGGAACACCA GGTGCCAAAT
 601 ACGGAACAGG ATACTGCGAT GCGCAGTGCG CACGAGACTT GAAGTACATT GACGGTCAAG CCAATGCCGA AGGCTGGACG GCTTCGTCAT CCGACCCCAA
```

```
 701 CGCAGGCATC GGCAAAAAGG GCGCGTGCTG TGCCGAGATG GACATTTGGG AGGCAAACTC CATGGCCACC GCCCTGACCC CACATTCCTG CCAGCCCGAG
 801 GGCTATTCCG TCTGCGTAGA CGACACATGC GGTGGTACCT ACTCGCTTGA TCGCTACGCC GGTACCTGCG ACGCCAACGG CTGCGACTTC AACCCCTACC
 901 GCGTTGGAGT GACGGACTTT TACGGCAGGG GCAAGACCGT CGACACCACC AAGAAGATGA CAGTCGTAAC GCAGTTCCTG GGCTCTGGTA ATAAACTCAC
1001 GGAACTCAAG CGCTTCTACG TACAAAACGG TAAAGTCTTT GCTAACCCCG AGCCTACTGT TCCCGGCATG AAAGGCAACT CAATCACCCA GCAGTGGTGT
1101 GACACACAGA AGGAAGTGTT CCAAGAAGAA ATCTATCCCT TCAACAAATT TGGCGGCATG GACTCGATGG GTAAGGGTAT GGCGTTGGGC ATGGTCCTCG
1201 TCATGTCGCT TTGGGATGAC CACTACGCCA ATATGCTTTG GCTCGATGGC AAATACCCCG TCGATGCGGA TCCAGCGAAG CCAGGTGTGG CGCGCGGCGC
1301 ATGCGAAGCC GGCAGCGGTG TGCCTGCTGA TGTGGAAGCC AACAGTCCTA ATGCTCAGGT TGTATTTTCT AACATCAAGT TTGGGCCGAT TGGAAGTACA
1401 TTTGCGCAGC CTAAGTAAGG GTCGAAAAAG AAAGGGGATT TAGGAGGTGG CGGAAAAGCA GCCACGTAGG TGCTTTCTAC GGTGGCGCTG GAAGTTGCTG
1501 GTGTACGTAT GCAAGT
```

GH45 genes

>*GH45-1*(jgi|Cocsa1|40868|fgenesh1_kg.16_#_82_#_Locus110960v1rpkm0.58)

```
   1 TGGGCGATGA GGTACTGCGC AGGGCAGGTC CCACCCCGGA AGCTGACTGC ATTGGGTCGC CGGCTGAAGT AGAGAGTAGA TTGTAGATTC TTCAGATACG
 101 GGGAAGATTT GACAGCGCAG CCAAACAGCG GAATCGATCT AGGTAGAGAG TGTGGAGATG GAAGTCTAGA AACTTTCCAT CGTAACCATG ATGTTGGGTA
 201 GTATCAAAGT ATATAAATAT GCCTTGGAAG CACATTTGAC CATCCATCAA CTTGCCAAGT ATCTTCGAAA AGACTACCAA TCTTGCCCAA CATGGTATCT
 301 CTTAAGCAAC TCAGTTTTGT CGTCCTCGCC ACTGGCCTCG CCACGGTTGC TGCTCAGTCC ACAGGAAAGA CGACACGTTA CTGGGACTGT TGCAAAGGCT
 401 CCTGCGCATG GGCAGGCAAA GCCAGTGTCA CCCAACCTCT CAGGACGTGT GACAAGAACG ACAACCCCAT TAGCGATCTG ATGGCGAAGA ACGGATGTGA
 501 GAGCGGTGGC ACTGCGTTCA TGTGCACTAA CCAGACGCCG TGGGCAGTCA ACAACAGCCT CGCATATGGC TTTGCTGCTG TAAAGCTGGC CGGAAAGAGT
 601 GAGCGTGACT CATGCTGTGC TTGTTATGAG TTGACCTTCA CGTCGGGCCC CGTCGTAGGC CAGAAGATGA TTGTGCAATC CACCAACACG GGTGGTGACC
 701 TTGGAGACAA CCACTTCGAT ATTGCTATGC CAGGTGGTGG TGTAGGCATC TTTAACGGCT GTACTGCACA GTTTGGCGCG CCATCCACCG GGTGGGGTCA
 801 ACAATACGGT GGCATCTCGA GTCGCTCTGA ATGTGAAAAC TTTCCTGAGA AGCTCAAGGC AGGGTGCCGG TGGAGATTTG ACTGGTTCAA GAACGCCGAC
 901 AACCCGACTA TTAGTTTCAA GGAGGTCACC TGCCCTGGTG AGTTGACAAG CAGGACTGGC TGTATCCGGT TGTAGAGAAG GGTGAAGAAG AATAGGAGTT
1001 CCGCCATTGA TACTTGATCA GCACAAAGGA GGCAAGATGT ACATATGCAC ACATGCAATG ACGAAGCGGT GACAAACCAC GGCCAGCTGT CCCGCATCGT
1101 GAGGCATTAT AGTCAAATAA GTTCAGATCA TCGTCATCAA CCTTTTGGAG AAAATGCTTG CGTTTGTGAC ACGGCAGAAT TGTTGTCGGG GAGAAAGTGT
1201 TCCCGGACCG AACCTACACC GCCCGGCGAG GGGAAGATTG CAGCT
```

>*GH45-2*(jgi|Cocsa1|135373|estExt_Genewise1Plus.C_2_t60114)

```
  1 CTTTATTCAC ATTGACTCAC CAGTACCATT CTCTACCATG CTTTTGACTC TAGCACGCTT GTCGCTCGCT TTCTCCCTCC TTCTCCACCA TGTCTCCGGT
101 GAACGAGGTG TGACGACTCG GTACTGGGAC TGTTGCAAAC CGTCGTGCGC TTGGCCCGGC AAAGCAAAAG TGTCGGAGCC AGTTCGTACC TGTAACAAAC
201 AAGACCTGTG GCCGACTCCT CTCGACGCAA ATGCGGCTAG TGCATGTGGA AATGGCGAGG CCTACACTTG CAGCAATAAC GGGCCGTGGG CCGTGAATGA
301 TAACCTGGCT TACGGGTTTG CGGCGGCGAA TCTGAAGGGA AAGAATGAGG GGGACTGGTG CTGTGCTTGC TACGAACTCA CTTTTACCTC GGGTCCCGTC
401 AACGGAAAGA AAATGGTGGT GCAGGTAACC AACACAGGCA GCGACTTGAA GGAGAACCAG TTTGATCTTG CCATTCCCGG CGGCGGAGTC GGCATTTTCC
501 CTCAAGGCTG TGCGAAACAA TTCAACGGCG CGTGGATGGG CAATACTTTC GGGGGTTATT GGCAGCGAGA CCACTGCTAC AATCTTCCTG CTGGAGCTTT
601 TCGCGATGGC TGCTTCTGGA GGTTTGACTG GTTCAAGAAT GCGGATAATC CCAGTGTGGA TTTTAGAGAA GTTAGCTGCC CGCAGGCGAT GATTGACAGG
701 ACCCATTGTG GAAGGTGGAG CTGAGAAGGT GTCTGTGTCG TTACATGGTG GTTTTTCGTA GTGCACTGCC AGCTCCGGTG GTTCCCTCGC CATGCATACG
801 AAGTAGTAGC AGTTGAACTC TTGTGTCCTT ACGCTTATAA CCGAGCGAGA TGAATGGAAA ATATGATGAT AGATTAATGG TTTCAGT
```

>*GH45-3*(jgi|Cocsa1|36957|fgenesh1_kg.7_#_289_#_Locus8651v1rpkm11.53)

```
   1 ACCTCGACCA CTCATCTACA TCTACCTCTT CCATATATCC ACAACCATAC GCCCACAAAC ATGGCGCGAC ATGTCCGAAA CACTCTCATC TCCCTCCACC
 101 TCTTTGCCCT TCTTTCTCAT GCCGCCAATC TCAACTACTC GGGCGAAGCC GTGACTACGC GGTACTGGGA CTGCTGTAAA CCATCGTGCG GGTGGAATGG
 201 CAAGGCCGAT TTCAGCAGCC CGGTAGAGTC GTGCACAGCA GACAACAAGC CCACCAACCC TGCCGCTGGC ACTGGCTGTA ACGGTGGTGA TGCCTTCCAG
 301 TGTGCCGACC AGCAGCCATG GGCTATCAAC GACACAATGT CCTATGGCTT TGCCGGTGTC TACATCATGC CCGCCCTCAC GCGCGGCGGC ATCGAGGACG
 401 CTTGGTGTTG CGCATGCTAC CAGCTAGATT TCACCAGTGA CCCACTGATT GGAAAGAGCA TGATTATCCA GGCCTCAAAC ACGGCGTACG ACATTACAAC
 501 AACAAACCGC TTCACCCTTG CAGTACCTGG TGGCAACACA ACATCGGTCG ACGGATGCGC CAAGCAGTAT GGCGTCGACC AGTCCGTCTT TGGCCAGAGG
 601 ATGGAGGGCG TCAGTTCAAG CAACGACTGT GCAAACCTAC CCGAGCCGCT GAGAGCGGGA TGCCAGTGGC GCTTTGATTG GCTCCAGGAT GCGTCGTTTC
 701 CTAGTGCCAA TTTCAAGCGT GTCGTGTGCC CAGCCGAGAT TACGGCCAAG AGCAACTGTG TGCGCAACGA CGACAAAAGG CTTGCTTCGG GCGAGTCGAC
 801 TTCCGCAGCC TACAGTCTCA GCCCTAGCCT GCCTCATACT ATGGCTTTCG TTGCCGCCAC GATGCTAACC CTAATATCGG TATAGCATCG GTGATGTTCC
 901 CTGCGCGTCA CGTTCCTGCG GCCCGCTCAC GATCCGTCAC TCTTCATTTC GCTTGCCCTC TTTGCACTCT CGAAGCCGAG ACTGATGGCA TCGTCAGATG
1001 ACAAAGAAAG GCAAGCCCAT TTCATGCATG GATACCCTAC TTGATAAAAT ACCCGCATTA ACACGCCTGC CCTCCGTGTC CAGACCGCAC TCGCTCAGGA
1101 CTCGGAGTCG CCGGTGATGT GCTGGTGCAT AAGTGGCTTT CCCCGGTTTT TTTTCCTCAG TCTAGTATCA TCTGGCCACC AATGGAAAGG GTGTGTGTAA
1201 AAAAGATGAC ATGTACGCTA TTACGATTGA TCTGCATATT GGTGTTTGGA AAGCTTTTTC ATTTAGTGGG TGCCGGTTTA TGGACCACAT TCAGCCTTTG
1301 GAGCGTTCTG TACTCTCGTG AGAATGATGA TCACTTTGTT CTTGACCACT CA
```

AA9 genes

>*AA9-1*(jgi|Cocsa1|201224|estExt_fgenesh1_pm.C_100203)

```
  1 CCGTCTCTCT TTATCCGCAG CATAAACAGA GACTTTTCTG CAAAGATGAA GGTTTCCGCC GTTATCCTCG CTGTCGCCTC TCTGGCCAGC GCTCACTACA
101 CTCTCCCCAG CATCAACGGC GGCGTACCGT GGGCTCACGT CCGCCAAGCC AAGAACTGGC AGGACAATGG CTTCGTCGGT GATGTCACCA GCAACGATAT
201 CCGCTGCAAC CAGCTGAAGC CTGGCACTGC CACCATGACC GTCGCCGCTG GTTCCAGTGT TAAAGTCAGC GTCAACCCCA ACGCCTACCA CCCAGGCCCA
301 TTCCAGTCAT ACCTTGCCAA GGTACCCGCA GGTGCAGACA TCAACACCTG GGACCCCACT GGCGCAGTCT GGTTCCGCAT CTACGCCGAG CAGCCCAAGT
401 TTGGCAACCA GCTGACCTGG TTGGCCGCTG GCACCTACGA CATCAAGATC CCCCAGTGCA TTGCCCCCGG CAAGTACCTT ATGCGCAACG AGCACATTGC
501 CATCCACACT GCCGGCCAAA GGGGCGGCGC ACAATTCTAT CTCTCCTGCG CACAAATCGA GGTCACCGGT GGCGGCAGCA CGTCGCCAAG GAACCTTGTC
601 GCCTTTCCAG GCGCCTACAG CGCTACTGAC CCAGGTATCT TGATCAACAT CAACTACCCC ATTCCTACTA GCTATGTTAA CCCTGGCCCA CCTACCTTCA
701 CTTGCTAGAG TGCCTTGGGT ATGTAGGAGT GGTGAAGTAG CAACACTGGG GTCCTGTATA TAGATTATTT TTTCTCCATC CCTGCTGTTG TTGAGGAAGA
801 CTTGAATGAG GTTGGGAGGG TAGAAAGCTT TTTTTTTGCC TTCCCTCGAG CTCTATATGA TATGATATTA TTATAGAGAA TGTAAATATG TTCTATGGAT
901 ATACTTTCTT GAAACGATAC ACGCAC
```

>*AA9-2*(jgi|Cocsa1|173430|fgenesh1_pm.11_#_152)

```
  1 ATGAAGAACA CACTCCTCTT GACCAGCATA TCTGCCTTTG CAACTCATGT TGCAGCCCAT GCAACTTTCC AGGACCTCTG GGTCAACGGA TCAACCTGCG
101 CAAGACTACC CAACAGCAAT AGCCCCGTCA CCTCCGTAAC AAACAACGAC ATCCGCTGCA ACGCCAACCA AGGCCCCGCC GCCTCCAAAT GCAGCGTCCC
201 AGCCGGCACC ACCGTAACCA TCGAAATGCA CCAACAAAAC GGCGACCGCT CCTGCGCCAA CGAAGCCATC GGCGGCGCCC ACTACGGCCC CGTCCTCGTC
301 TACCTCTCCA AAGTCTCCGA CTCCTCCACC GCCGACGGCT CCACTCCCTT TTTCAAAATC TTCCAGGACA CTTGGGCGCG CAATCCGTCT GGCGGCGGAG
401 GCTCAGATGA CTACTGGGGC ACTAAGGATC TGAACAAGAA TTGCGGGAAG ATGGATGTAA AGATTCCTGC GGATCTCGCC GCGGGAGATT ACCTCTTGCG
501 TGCTGAAGCT ATTGCGCTGC ATGCAGCGGG TAGTACAGGT GGAGCGCAGT TTTACATGAC GTGTTATCAG ATTACTGTTA CAGGAGGTGG CTCGTCGGTT
601 CCGTCTGGCG TTTCTTTCCC GGGAGCGTAC AAGGCGTCTG ATCCGGGCAT TCTGATTAAT ATCTACCAGA AGCTTGCGTC GTATGTTGCC CCCGGCCCCG
701 CGGTTATTGC GGGTGGCACC GAAGCGGTTG CTGGACAAGC TGGGTCGGTG GTTACGGCTA CGGGAGGACC GCCGGTTGCT ACGCCGACTC AGACGAGTAC
801 GTTGAGGACG TCGACGGTGG CTTCGTCTGC GGCGCCGGCG CCGACGGATG GAGGTGGTGC GGGGGGTTGC TCGGTTGCGA AGTATGGGCA GTGTGGGGGG
901 AGGGGGTTCA CGGGATGTAC TACTTGTGCT TCTGGATCAA GTTGCAAGGC ATCGGGTGAA TTTTACTCTC AATGTTTGTA G
```

>*AA9-3*(jgi|Cocsa1|296458|CE91804_3850)

```
   1 CCCACAATTC AGAAGCCGGG GAGACAGGTA CTTCGAAATG AATCTTTTGA TTGCAAGGCG TACGGAAAAC CTGGTCGAAG GTAGGCGGAG GCGTTGGAAG
 101 AATGACGCTA GAAGAATGGC GACGAGTATA TGAACAAGAA GGAATCCAAC CTCAAGGTCT TGCTCAACAG GGACTTCTCA GCAACAGCCT CTTCACCTCC
 201 ACTCTCTTCT CTACATCGTT TGAAACGATC GTTTTCACTC CACCTTGTCA AACACCATCA CTATGCTCTC CCTCCTCGTT CTTCTTGCGC CCCTTGTGGC
 301 AGCTCACGGT CACGTTGACA AGGTCGTCGC CGATGGCAAG GAATACCAGG GCTGGAACGC TGCTCTCAAG TACCAGAACC CCATTCCTGC CACTGTTGGC
 401 TGGCAAGCCG ACAACCTCGA CAACGGCTTC GTCTCTCCCG ATGCCTTTGG TACCTCTGCC ATTGTCTGCC ACAAGCAGGG CAAGAGCAAC GGTGCCTACG
 501 TAACCGTCAA GCCAGGCAGC AAGGTCACCT TCAAGTGGGA TACCTGGCCC GTCAGCCACG TTGGTCCTGT CCAAGAGTAC ATTGCTCCTT GCAACGGCGA
 601 CTGCGGCTCC GTCAACCCTT CCAGCCTTCA GTGGACCAAG ATTTCATCCA AGGCCTGGAA GTCGGGCAGC AACCCCGGCA AGTGGGCTAC CGATGACCTG
 701 ATCGCCAACA ACTTCTCCTG GGACATTACC TTCCCTAACG TTGCTCCCGG CAACTACGTC GTTCGCCACG AGATCATCGC GCTGCACGCA GCCGGCCAGA
 801 CCAACGGCGC CCAGGCATAC CCCCAGTGCA TCAACTTCAA GGTTGAGGGC TCTGGAACCG GAAGGCTCAG TGGTGGTACT CCCGCTACCA GCTTCTACAC
 901 TGCCAACGAC CCTGGTATCA AGTTCAACGT CTTCACTTCC TTCTCCAGCT ACCCTGTTCC CGGCCCTGCT GTCAAGGCTC TGGCTAAGAG GCACGCCAAG
1001 GATCTCGAGT ACTAGACCAC TCTCTATGCA GTGCAGGAAA AAAACTACTG CATAAACTGC GTCGCGAGTT GATGAAGGGC AAGCCGGATA AAGTCACCGT
1101 AGCGTGGACC ATCCTAATCG CTTAATTCCT CGTAGAGAAC CCCCCTGTCT TTGTCTTGTT GCCTTTTTTT TAACAGACAA AGACCACTGT AAATAACGAC
1201 TTTTTGGATG CGAAGAGGAA ACACCATTCT TGTATATACT AGT
```

>*AA9-4*(jgi|Cocsa1|193395|estExt_fgenesh1_pg.C_140322)

```
   1 ACATTCCTTC TTTGCAAGAT TCGTCATTCT CTGCTTGTTT GTTTCTTTCA GTCACTCTTT CGTCACCTAG TTGACCAGCC ACTCATTCTC AACACCATCC
 101 CTACTATATA TCCCCACCAC CATATACAAG ATGAAGACCG TCGCCACCAT CGCCGCCTTG GCCGCCATGG CCAACGCCCA CGCCACTTTC CAACAGCTCT
 201 GGAAGAATGG AAAAGACCTC GAGAGCACCT GCGCCAGGCT GCCTTCCACC AACAGCCCCA TCGAGGACTA CACCAGCAAG GCTCTTCAGT GCAACGTCAG
 301 CCCTGCTGCC GCTAAGGGAA AGTGTGCGTT TGCAGCTGGT GACACAGTTA CCATTGAAAT GCACCAGCAG TTCACCAGCC GTGACTGCAA GACCGAGGCC
```

```
 401 ATTGGTGGTG CTCACTGGGG ACCCGTCCTT GCCTATCTGT CCAAGGTTGA AGATGCCGCC ACTGCCGATG GCTCCAGCGA GTTCTTCAAG ATCTACGAGA
 501 ACACCTGGGC CAAGAACCCA GCTGCGTCTC AGGGAGACAA CGACTTCTGG GGTACCAAGG ACCTCAACTA CAACTGCGGA AAGCTCGACT TCACTATCCC
 601 CAAGGACCTC GCCGCTGGTG ACTACCTCCT CCGTGCTGAG GCTATTGCGC TTCACGCCGC TGGCCCTGCT GGTGGTGCTC AGCACTACGT CACTTGCTAC
 701 CAGCTTACTG TTACTGGTAG CGGTACTCTC GCACCCAAGG GTGTCACATT CCCTGAGGCC TACAGCAAGA CTGGCCCAGG TCTTGGCTTC TCTATCCACG
 801 CCAACCTAGA CTCTTACCCT GCCCCTGGTC CTAAGCTGAT TGCTGGCGGC ACCACAGCCA CTCCCTCGCT CCTGACCTTT GGCACGCTCT CTGGTGCTCC
 901 TGCTCCTGTT GCTGGTGCTC CTGCTGATGA GGCTCCTGTC GCTTCCGCTT CGTCTGCTGC TGCTTCCACT CCTGCCGCCT CTTCCACTCC CGCTGCCACC
1001 CCTAGCAGCT CCGTCGAGGA TGCTAAGCCC TCTGCTGCCC CGACTGAGCC CTCTGCCACT CCTGCTGAGA CTCCCGCCCC CGTTGTTTCC TCTCCCGCTG
1101 CCCCCTTCCC TGTCAGCAAC AGCACTTCTT CTGCTGCTCT CCCTATCGCT ACCTCTTCCA GCGCCATCGC CTCTACCACC TTGGCGACTG CTGTCCGCCC
1201 ATCTCCTACC GGTGATGCTA GCGGCGATGC TAGCGGCTCT GTTAAAGAAT TCTACCAGTG TGGTGGCATG AACTACAAGG GTGCCACTGG ATGTGCCGAG
1301 GGCCTTGAGT GCAAGCAGTG GAACCCCTAC TATTTCCAGT GCGTCAAGTC TGGATCCAGC AACGGTTCTC AGCCTCCGCC ATCCAACAAC GCTCCTTCCA
1401 CTGGCGCTCC CTCCAACAAT GCTCCCTCTA CCGGCGCTCC ATCCAACAAC GGCCCCTCAA ACAACACCCC AGCCCCCAAG CCCGAGCCCA CCAACGTCAC
1501 CCCTGAAGCC GCTCCTTCCG CCGCTCCCTC CGCCCCCGCT GCCGGCGCTG GCGAGAAGAA GTACACCCTC GAGACCTTCA TTGCCTTCCT TGAGGAGGAG
1601 GCTGGTGCCG AGAAGGCGGC CAAGATCCGC CGTATGATTG AGGCTCTTTT CTAA
```

>*AA9-5*(jgi|Cocsa1|209542|CE4888_13651)

```
   1 CATTCATTCA TTACCTTGAG TCCTAGTGAC CAACCCTTGC AACCTCAGTT CGAGCGTCAC TACCAAAAAC ACGTTGTTTT CTGCCCTTCA AGTCAACTGT
 101 TGAACATCCA ATACCATGAA GACCCAATCT ATCCTTTTTG CGGCCCTGGC CTCGGCCCCT GCTGCCCTTG CCCACACTGT CTTTACTGAC TTTTACGTTG
 201 ACGGCGTGCC TCAGGGTGAT GGTGTTGCTA TTCGCATGAA CCCTTCAGGA GAGCACACTA GCTCCCCTAT TGGCAGTCTT GATAGCACTG ACATGGCTTG
 301 CAATGTCCAG GGTACCAAAG GAGTTTCGCG CACCCAGCCC GTCCAGGATG GTTCCGTTCT TACCTTTGAG ATTCGCGAGT GGCCCGATGA CCCATCAAAG
 401 GAGCGCCTTG ACCCTGGCCA CAAGGGACCT TGCGCCGTCT ACCTGAAGAA GGTCGAGGAT GCCACCACTG ACACTGCTGC TGGTGATGGC TGGTTCAAGA
 501 TCTTTGACCA AGGCTACGAC GCTGACTCCA AGCAATGGTG CACTGACAAG ATCATTGCAA ACAATGGTCT TCTGTCCGTC AAATTGCCCG AGAACCTTGT
 601 GCAAGGCGAC TACCTCGCTC GTCCCGAGAT TCTTGCTCTC CATGCTGCCA AAGACGGTGA CCCACAGTTC TACACTGGCT GTGCCCAAAT CTTCCTCAAG
 701 AGCACAGGCA ACCTCGTTCC CGAGTCCACT GTATCTATCC CCGGACACGT CAAGTACAAC GAGCCTTCGA CCGACTTTGA CATCTACAAC ACTCCTGCTA
 801 CCAAGTACGT TGTGCCCGGC CCCAAAGTCG CCAAGCTCAC TGCTGGCGCG ACTAAGTCTT CCGCCACTGT CCAAACCAAG GGCCTTAAGT CTCAGGGCTG
 901 CGTCATGGAG AACGCAAACT GGTGTGCCAA GGAAGTCCAA TCCTACACCG ATGAGAAGGG ATGCTGGGCT GCCGCTGAGG ACTGCTGGGC TCAGAGCGAT
1001 GTATGCTACG ATTCCGCACC CCCGACTGGT GGCAAGAACT GCAAGCTCTG GCAAGAGAAG TGCACCGACA TTGGCGCGCA GTGCAAGGCT GGCAACTGGG
1101 TCGGTCCTCC TAACAAGGGC AAGGACCTTA CACCTGCCAA GAAGTCCATT GATGTAGGCC TCGTCATGCC CAACCAGGTC CAGCCTGCCG CGCCATCCAA
1201 GGAGACGCCA GCGCCTTCAT CTGAAAAGGA GCAGGAGGAG CCAGAGTCCA AGCCTGAGCC CAAGCCTGAG ACTAAGCCTG AAAACTCCGA GTACCAACCT
1301 GCGCCTGCAC CTGCTAAGCC CGCTCCCGTT GCCGAGGCAC CAAAGCCAAC TCCCACCCCT GATGCGCCCG CAGCCCCAGC CAACGATGAG TACGACAGCC
1401 CTCCTTTCAT GGAGCCCACC AACTACCCTG CTGCTCCCCC TCCAGCCCCC TCCAAGCAGC CCACCACCCT CAGCAAAGCC CCCATCTCCA CCATCACCGT
1501 TGCCCCTTCA TCGGCTGGTG GTCCCAAGCC TACCCAGCCC GCTTGCCCCA AGGGATACAA GTGCGTCTAC GTGACTGACC TCGTCGTCGT CACTACCACC
1601 GTCTACGTGA CTGAAGGAGC TGCGCCCACT GCTACCCCTA CGCCTAGCTC GGGGAGCTAC ATGAAGCGTG CTCATCTTCA TCCCAGGAGG CGTCTGCACC
1701 TCGCTTAAGT CTAAGCTGGT GACTGAAGAT GTATATCTAC AGCTATAACT TGGGAGTTCA GATCAAGGAA AACGGGTTAC GTTCACGCCA ACGCTATATG
1801 ATTGGCAACT TTTGGTACTT GGGGTTGGGG TCTTGGCCGC GAAAAACAAT TTTTTTTTTT ACCTTTCTGC GACACTTTTG TTGTTCGCGA GTCAATGACT
1901 GGCTCTGTTC TTATGAGGGG GAATGGAATG TAGGATAGGA AGCAGACTTT TGTCTTATAT TCTTTCTTTC TTTCTTTTTT CT
```

>*AA9-6*(jgi|Cocsa1|141568|estExt_Genewise1Plus.C_6_t30009)

```
   1 ATCTATAAGA AGCAGTGGAA GTGCCGTGAA ACCAAATCCA CAGCCCGCGA CAATCACTTC CAAGCATAGC AACAGCAACT TGACACTAAA CGCAGATATG
 101 CACGCATCCA CTCTCCTCCT CGGCGCTGCC GCCCTCCTTG TCGAGAATGT ATCCGCCCAC GGTTTCGTCA AGGCCGTCAA CATCAAGGGC GTCTCAACCA
 201 AGGGCTCCGA TCCCGTCTGG TTCTACCAAG GAAACAACCG CGTCGCAACC GCTGGCTGGG ACTCTCTGAA CCAGGATCTC GGCTTCGTCG AGCCCAACAG
 301 GGCCTCATCC GCTGACGTCA ACTGCCACAA GAGCGCTACA CCAGGCAAGC TGTACGCCAA CGCTAACCCG GGCGATACCA TCGAATTCGA GTGGAACACT
 401 TGGCCAGACA GCCACAGGGG TCCCATCATC CACTACATTG CTCCTTGCAA CGGTGAATGC TCAAGCATGT CCCCCGGCGA CCTCCGCTGG TCCAAGTTCG
 501 CCCAGGAGGC TATCGTCAGC CCCAACGTCT GGGTCACTGA CAACCTCATC AAAAACGGCT TCAAGGCTTC TGTTAAGCTG CCGTCTAAGC TTGCTGCTGG
 601 CAACTATGTT GTTCGCCACG AGATTATTGC CCTGCACGGC GGATCGAGCC CTAACGGTGC TCAGCTGTAC CCTCAGTGCA TCAACGTCAA GGTTGGAGGC
 701 AGTGGCACTG TTAGGCCCTC TAACGGTGTC CCAGGTACCA GCCTGTACAC CCCCACCACC CCTGGTATCT TGTTCAACAT CTACACTAAC CCTACTTCTT
 801 ACCCCTTCCC TGGCCCTGCT CTGTGGACTG CTGCCAACTA GATGGGTCAA TGTGGGTTTG AGATGAGATG AGGGAGGTTC TCGAGATGGC CAAGGTTCTC
 901 GCCTGTTGGA ATAGGCTGCT CTTCTACTCT GTATATACTT CACTCTTTCT TGAGGGGTTC ATGGTCACGT TGGACATGTC CCCAATGTAA ATGGATGCTA
1001 TTACGACATG ACCTTGTCTA CTTTGTAGTA GACAGGTCGC ATTATATACA TGACTTGCAA ATCATCTTTG TTGGAATTAC TGATCGGTAA TTCTGCTGTG
1101 CATCGTCAAT
```

>*AA9-7*(jgi|Cocsa1|113504|estExt_Genewise1.C_4_t40025)

```
   1 CACTGCCGTG TGTGTATCAA TTTATCTCGG CATCGAGGTT GGTTGTGTAC CGAACCAGAC TTCTATCTTT TCGAAGTAGC TTATATAGAA TCCTCTTGTC
 101 TCGGCCTTTT CTCCTTTCAC TGCACTCCAT CTCCTTCCTT CTTCAACTAG ATTCTGAAAC AAAGGAAGAA CAACAAAGAT GAAATACCTC GTCCCCACCC
 201 TCGCCTTCGC TGCCACAGCT GCAGCCCATG GCTACGTCTC CAACGCCACC ATCGGCGGCA AAGACTACAC CTTCTACCAG CCCTACCAAG ACCCCTACAC
 301 CACCGGCGTC AAACGCATCT CGCGCCCCAT CCAGGGCAAC GGTCCCGTCG AAGACGTCAC GCTTTCCGAC CTGCAATGCG GTGGCTACAC TGCTGGCGGC
 401 TTCAAGGGAT CCTCCCCCGC CGCCCTACAC GCTGAGGCTG CTGCAGGCTC CGAAGTCAAG CTTTACTGGA CGCTGTGGCC TGAGAGCCAT GTTGGGCCTT
 501 CGATTACATA CATGGCAAGG TGTCCCGATA CGGGGTGTGA TGCGTATATG CCGGAATCCA ATGCCGTCTG GTTCAAGGTC CAAGAAGAAG GACGCCAAGG
 601 CACATCTAAC AAGTGGGCTT CCGATGCTAT TCGCGTTGCG GGCGGATACC TTTCGTATAC CATTCCCAAG TGCATTCCTG CGGGGTATTA TCTTGTGAGG
 701 CATGAGGTGC TGGCGCTGCA CTCTGCGTAC AAGTATCCTG GCGCGCAGTT TTATCCGGGG TGCCATCAGC TTAAGGTTAC TGGGGGTGGC AGCACGAAGC
 801 CGTCTGGTTT GGTGAGTTTT CCCGGGGCGT ACAAGGGAAC GGATGCGGGT ATTGCGTATA ATGCTTATAC TGCGCAAACT TATCCTATTC CTGGACCGAA
 901 GCTATTTACC TGCTAGATGG TAGGGGAGAA AAGGGGGCGG TGAGGTTGTG GGGTGAGAGG AGAAGTGTAT ATAGAATGAG AGGTTGGCTG TAGATAGTTT
1001 TGCTTCTGTA TATACTTTGG GGTTGTTAGA ATGGATAGAC GTGACGCTTT TTG
```

>*AA9-8*(jgi|Cocsa1|36761|fgenesh1_kg.7_#_93_#_Locus5809v1rpkm18.51)

```
   1 ACCTTCATCG CTACAGCTTC AATTCGTTGG TTTCTATACC CGTCCCTTCT TTCCTGCACA TTCGTTCATC AAGTCCGTTC ACTGTGTGAA GCCGGCAGAC
 101 ATTCTTTCAT TCCCATCGAA CATTCATTTC CATCGAACAC CTTCAACCTT TCCCAGATCA TCTCAATCTA CCACAATGTA CAAGACTCTC GCCTCCACCA
 201 TCACTGCCAT TGCGGCTCTT GCTGCTACCG CCACTGCCCA CGGCTCCCTC ACTGGTGTCA GCCTCGATGG TGTCTACGAG CAGGGCTACC TTATTGAATA
 301 CTACTATGCC ATGCAGCAAG GCGGCAAGGT CCCTGAGCAC CTTGGCTGGT ATGCTGAGAA CCTCGACAAT GGCTTTGTAG AGCCATCAAA GTTCGGCACT
 401 GGCGACATCA TCTGCCACAA GGCCGGTAGC CCCAAGGGTA GCTCAGACAC CATGGGCAAG ATCGCCGCTG GTGGCAAGAT CGACTTCCAC TGGAACACCT
 501 GGCCCGCATC CCATGTTGGT CCTGTTCTCA CTTACGCTGC TTCCTACACC GGCGACATTC AGGCTGTCAA GAAGGAGGAC CTTAAGTGGT TCAAGATTGA
 601 GGGTGTTGGT TACGAGAACG GCGAGTGGGC TGCCGTCAAG ATGATTGCCA ACAACAACAC CTGGACCACC ACTGTCCCCG AGAACCTTGC CGCTGGCAAG
 701 TACGTCTTCC GCCATGAGAT CATTGCGCTC CACGCCGCTG GCCAGGAGAA CGGTGCCCAG AACTACCCCC AGTGTGTCAA CATTGAGGTT ACCGGCAGTG
 801 GTACCGAGAA GCCCGAGGGT GTTGTTGGCA CTAAGCTCTA CACTTCCACT GACCCCGGTA TCAAGTTTGA CGTCTACCAA AGCAAGTTGG ACAGCTACGT
 901 CGTCCCTGGT CCTCCTGCCA TGGGCGCTGG CTCCGGCTCC GGCTCCGGCT CCACCCCTGC CCCCTCCAAG CCTGCCCCTT CATCCACCCC CTCCAGCTCG
1001 CCCTCTGCTC CCTCACCGAG CGCGTCTGCT CCTGCTGCTT CTGTCACTCC CGAGGCTCAG AAGCCCTCTC CTTCCACTGC TCCTTCAAGC GGTAACCTCC
1101 CCGAGACCTT CACCCTCGAC ACCTTCATCA CTTGGCTCGA GGGCAAGGCC AGCAGCTCTG CTAGCAAGGC CCGCCGCCAC GCTCGCGCTT TCCTGTAAGC
1201 GTTTTCAGGA TGGCTTATCA TTCGTCACGG CGATGAGATA ACGTGATGGA AATTCTTTTG AGGTTCGTTC GTTCGTTGGA TACGACGAGT GCCTTCGATT
1301 CTTTGTGTAT AGTACATACC CTTTTTTGTA GCATAGCGCT CTGAACATAT ACCTTTTCGC CACA
```

>*AA9-9*(jgi|Cocsa1|116305|estExt_Genewise1.C_6_t20397)

```
  1 GTTGGTCATG TGGGAATAAC GGGGGAGTGA TATAGGGGGA GGTTTGATTT GGACGGTGTA TTGTCGAGGG TGAGTGTATA TAAAAGGGGA GGCTGCGTGC
101 AAATTGGAGA GAAGGAGTGT GAGATAGTGT TGAAGACGAA GAGCAAACCA ACTACTATCG ACTCAACATG TTGTTCTCCA ATATCTTCCT GCCTACGGCG
201 CTCCTGGCTG CTCAGGCTGC TGCCCACGGC GCAGTCACCA GCTATGTTAT TGATGGCGTG ACATACCCAG GCTACGAAGG CTTCTCCCCC GCCTCCTCCC
301 CGAAAACCAT CCAACGCCAA TGGCCCGACT ACAACCCCAC CATGAGCGTC ACAGACTCTA AAGTCATGTG CAACGGCGGC ACATCCGCCG ACCTCGTCGC
401 AAAGGTCAAA GCCGGCGGCA AGATCCGCGC AATCTGGAAG CAGTGGACCC ACGAGCAAGG CCCGGTTATG GTCTGGATGT ACAAGTGCTC CGGCGACTTC
501 AAGTCGTGTA ATGGAAGTGG AAAGAAGTGG TTCAAGATCG ACCAACAAGG CATGACCGCT CCCCCCCTCT CCGGCCGCAA CTGGGGCACC GCCTTCGTCC
601 TCAAGAACCT CTACTGGGAA TCCACCGTCC CCGCCACCCT CGCCGCAGGC AACTACCTCG TGCGCCACGA GCTGCTCGCT CTGCACCAAG CACGCAGCCC
701 GCAGTTCTAC GCTGAATGTG CGCAAATCGA GGTTACGGGT GGAGGCTCGG CGTCGCCGAG TGGCGATTAC CTAGCTACGA TTCCCGGGTA TGCGTCGCAG
801 AATGATCCGG GTATCATGGT CGATACGTAT AGTAGCTCGC AGACTACATA TACGTGTCCC GGACCGAAGG TGTGGAGTGG TTAAGAGATG TGGAGATGGA
901 TAAGGGGAAG ATGGGGG
```

>*AA9-10*(jgi|Cocsa1|140540|estExt_Genewise1Plus.C_5_t40151)

```
  1 TTCGAAAAGA CACCAGCGCA AAACATCAAT ATGAGACTCT CAACACCTCT TGTCCTCGCT GCGGTAGCCT CCTCAACGAC CGCCCACACT ATCTTTCTTA
101 GCCTCAATGG CGGTGCCGTT GGCGACGGAG TTCGCGTCCC CACATACGAT GGCCCCGTAA GCGATGTAAC CTCAAATTCA ATAGTTTGCA ACGGACCCCC
201 CAACGACGTC CCTGCCAAAA CCAACACCGT CATCACCGTC CAAGCTGGCT CCAAAGCCAC CCTGACATGG CGACACACAC TCACCTCTGG GCCCAACGAT
301 GTCATCGATG CTTCACACAA GGGACCCGTC ATGGCTTATA TGAAGAAAGT CAGCGATGCA AAGACCGACT CTGGTGTTGG CGGGGGGTGG TTCAAGATTG
401 CACAGGATGC TTTTGACGGC TCGAAATGGG GCGTCGATCG CCTCATTGCC AACCAGGGCA AGCAAACCAT CACCATTCCA GCTTGCATTG CACCAGGCCA
501 GTACTTGCTC CGCGGCGAGC TTATTGCGCT CCACTCTGCT AGCCAGCTCA TGGGAGCCCA GTTCTACATG GAATGCGCGC AAATTAATGT CATTGGTGGC
601 TCTGCTAGTA AGACTCCTGC GACTGTCTCG TTTCCTGGAG CGTACAAGCA GAATGATGCG GGTATCGTGT ACAACTTGTA CACTGGGTCA AAAACTTATA
701 CCTCGCCTGG ACCGGATGTT TTCACTTGCT AGAGATGTCG ATGAGTGGTC TCCATTAATT GTAAATATTT CTCAGGATAG ATTCTTACCA TTAGAGAGTT
```

```
801 TTACACAATA AATACTTGGG
```

>*AA9-11*(jgi|Cocsa1|217959|CE13305_3528)

```
   1 TCTGTCAGTT AGTAGCTCTA ATCCCGCAAA GCTTTTGAAT GCGCCATGTT GGTGCCCCGA GTATAAGAAG TGCCCATGGT ATCTCCCAGT TGAAGGGCAT
 101 CAAGCTTGAA GCTGACACTC ACTTTCATAC ACTCTTTCGC ATCCATCATC TTTCGATTCG TTCGTCTGCC TTTCGTTCTT TGACATTCTA TCTTTTGAAC
 201 ATCTTCTCGA TCTTTTCAAA CCAACCAACT TCCACATACC AACCATCACC ATGTTTGCCA AGTCCGCTGT CATCCTGGCT ATTGCCAACC TCGCTGCCTC
 301 GCACGCTACT TTCCAGAGCT TCGTTGTCAA CGGAAAGAAC ATGGGCCAGA ACTTTGCTGT TCAGAAGCCA TCCAACGGCA ACAACCCCAT CCTCGATGTC
 401 ACCTCCACTG CCATGATTTG CAATGGTGGC AAGGCCACCG CTGACCAGGT CGAGGTCGCT GCTGGCTCCA CTATTGGCAT GCAGTGGCAC CACAACGAGG
 501 GCCCCAACCC CAATGACAAG GAGGAGCCCA TTGCCCCCTC TCACAAGGGT CCCGTCATGG TCTACATGGC TGAGGCTTCC ACCAACGGTA TCGGCAACGT
 601 CTGGACCAAG GTCTGGGAGG ATGGTTACAA GGACGGCAAG TTCGGTGTTG ACAGGTTCTA CGACAACAAG GGTTTGATCG AGATCAAGCT TCCCAACGTG
 701 AAGGCTGGCA AGTACTTGAT CCGCCCTGAG ATCATTGGTC TCCATGAGGC TAGCGCCGTC GGCAAGGCCC AGTTCTACAA CGGATGCGGT CAGATCACCG
 801 TCACTGGCAG CGGATCTGCT TCCCTCCCTG CCTCTGGTGT CGACATGACC AAGGCTTACA AGGCCACCGA CCCCGGTGTC AAGTTCGATA TCTACAACGC
 901 CTTCTCCTCG TACACTGTCC CTGGTCCCAA GGTCTGGGAC GGTGCCTCTT CCGGCAGCGC TCCTTCCACC ACTCCTTCCA ACGGCTCCGC TCCTTCTACC
1001 CCCAAGCCAT CTCCTGTCGA GGCCGCTCAG CCCGCTACCC CCTCCAAGCC CGCTGAGAAC ACCACCCCTT CCACCCCTTC AACTGGCAAC AACGGCTCTG
1101 CCCTCCCCAA GACCTTCACC ATCAACGAGT TCATCCAGTG GCTCCAGACC ACCGCTGGCT CCGCCTCCTC CAAGGCTCGC CGCCACGCCC GTGCTTTCAA
1201 CTAAGCGGTT GTCTTTTGCA CATGTTGTAC AGCATTGCAA CAAAAAAATG GTGTTTGGAA CGTCCATTTG TTGGTTC
```

>*AA9-12*(jgi|Cocsa1|245914|CE41260_5930)

```
   1 ATCCTCATCA GCCTCTGAGT TCATCAACCA GCAGTCTTTT ACATTCTTTT CAGTCCTTCT TTTCGATTCT TTGCCTTTCG CAAATCACCT TCTTTTCAAC
 101 CAACCATCTA AAACATTCGT TACTGCTAGT TCAGAATGAA GTTCTCCGCT GCTCTTATCG CCGCCGTCGC CCAAGTGGCT TCCGCTCACT ACTTCATGGA
 201 CAAGGCCATT GTCGGTGGAG TCGAGACGCC TGCCTTCAAG TACGTTCGCC AGTCGACTCG TGCTACCAAG TACAACCCCA TCAAGTTCTC CTCCAACCCC
 301 GCGGCCGACA TCCGCGACAA CTCCCATATC GATGGTCCCG ATATCGTCTG CAACCAGGGT GCTTTCTCCT CTGCCGGCAA GACCGATGTC CTGACCGTCA
 401 AGGCTGGTGA CGAGATCCGC CTCAAGCTCG CTGTCGGCGC CAAGTTCCAG CACCCCGGTC CCGAATTTGT CTACATGGCC AAGGCCCCCA GCAGTGTCAA
 501 GACCTTTGAC GGAAAGGACG GCGCCTGGTT CAAGATCCAC GAGCAGGGTG TCTGCGATGG CGGCGATTTC ACCTCTACCG CCTGGTGCGA TTACAACAGG
 601 GATTACATTT CGGCCAGGAT CCCCAAGGAC ACTCCCAACG GAGAGTACCT CGCCCGCTTC GAGCACATTG GTGTTCACCG CTCCCACGTC AACCAGCCCG
 701 AGCACTACGT CTCTTGCATG CAGATCAAGG TTGAGGGTGG CGGCAGCGGT ACCCCCGGTC CCACTGTCAA GTTCCCCGGT GCCTACAAGG CCTCTGACCC
 801 ATACGCCAAC TTCAGCATCT ACAACGGCCG CAAGGCTTTC CCCATGCCCG GTCCTACCGT CTGGAAGGGA GGTGCTGGAA CCGCCAGCGC TGGTTCTTCC
 901 CCTGCCGCCC CTGCCAACAA CGCCCCTGCT GCTCCTGCTG CCACCACTCT CGCCACCAAG CCCGCTCCTG CTGCCACTCC TGCTCCCGCT GCTGGCTGCG
1001 CTGCTCAGTG GGCCCAGTGC GGTGGCGCTG GCTTCAATGG CGCCAAGTGC TGCGCCAAGG GCTCTTGCAA GACCATGAAC CCCTACTACT CCCAATGCCA
1101 GTAAGCGTCT CGCGCTCTGG TATGATAAAA AAGGTTCAAC GGTGATGCGT GAACGATGAT GGTGGATGGG CGACTTGCCT ACCACAACCC CCCCTTCTTT
1201 GGCTTCCGGG CTACTGTACA ATCTTTCTCA AGGGCTTTCC TGGGGACTGG GCTACTTTGA CTGCTAGTTT TCCCCTGTAT ATACTCGAGG CTCCATACTT
1301 TCTTGTCATA TAAATCATTT AGTTTTCTGA TTCTTGCTCC ACATGCTACT TTCCATGAGT GATGTCTATT GTCATGAACT GGTATTTTTT GTGAACAGTC
1401 AATGGTGTTG GAAAATAAT
```

>*AA9-13*(jgi|Cocsa1|33678|fgenesh1_kg.2_#_719_#_Locus23111v1rpkm3.09)

```
   1 CGTGAATAGA TTGCAGCCCA GCGATTGACG TAGACAACGG CAAGGCTTGG CCTCACCGTC TTGAAATGGG CAGTATCTTG TGTATCATGG CAACCGTGAA
 101 CGACTGACTA TATAAGAGAT GGGCAAGGCG ACAGAATAAG GCACCTCACC TTGCCTTACC ACTCATTCAC CACCACTACA CATTCATCCA CTCTTTCAAC
 201 ATGAAGTTCT TCGCTGCTGC CATTGCTCTG CTGCCCGCTC TTGGGTCGGC CCACACCATT GCCCAGCGTG TCCGCGTCAA CGGTCAGGAC TTTGGTCTAG
 301 GCAACGGTAT TCGTGTCGCC TCGTCCAACA ACCCCATCAC CAACGTCAAC GATGGTGCCA TGGCCTGCAA CACCGGTGTC TCTTCTTCGA GCAAGGTCAT
 401 TGATGTCAGG GGCGGTGACA GGGTTGGTGT CCAGTGGGGC CATGTTGTTG GAGGAGCCCA AGGTGCCAAC GACCCAGACC ACCCCATTGC CAAGTCGCAC
 501 AAGGGTCCCT CCATCTTCTA CATGGCCAAG GTCTCCAACG CAGCCAGTGC CTCCCCCTCT GGCCTACAAT GGTTCAAGGT CGCCGAGGAC GGTCTTCAAG
 601 GCTCTACCTG GGGAGTTGAC CGCATGATCT CCAACAACGG ATGGACCGAG TTCACCGTGC CATCCTGCAT TGCTTCAGGC CAGTATCTCG TGCGAGCTGA
 701 GCTGATTGCG CTCCACAGCG CTAGCTCCGT TGGCGGTGCA CAGTTCTACA TTGGATGTGC TCAAGTCAAC GTTTCGGCAT CAGGATCAAA GACTGGAACC
 801 ACTGTCAGCT TCCCTGGCGC TTACCGGGCC GATGATGCTG GTATCAAAGT CAGCATCTAC AACTCTCAAG GTCAGCCCAC TGGTAACGGT CAGTACAAGA
 901 TCCCCGGCCC AGCCAAGCTT CAGTGCTAGA GAGGGGTAGC TATGGTAGAA GGCTTGTAGT ATAGAAGGGA CCAAGTCAAG GCAGTAGAAC AAGGACTTTT
1001 CTCTTCTCAA CACCTCATTT ATCCATACTT TGTTAGCCAA ATGAAACATT ACTTTGTGCA
```

>*AA9-14*(jgi|Cocsa1|41199|fgenesh1_kg.17_#_102_#_Locus15900v1rpkm5.17)

```
   1 CTGGTATGAC GGCCCGGGAC AAAACCGTTG CTGTTAGATT GCTCGGTGTC AGCCAGCAAG ATGGGGCGTT CAGCAGCATG AGGAATAAAT AAGAGCAGCC
 101 TTCTGCCTTG AAGTTGAACA GCACGACACA GACGATCACT TTCAAACACT CTCACATCAA CTCTCCATCG CAATCATGAA GTACTCCACC ATCTTCCTCG
 201 CTGCTGCGAC CACCGCCTCT GCCCACTCGA CATGGCAGCA ACTGTGGGTT GGAAGCACGG ACAAGGCGAC CAGCTGCACG CGTACTGTCA AGGATAACAG
 301 CCCCATCAAC AGCTTGTCTT CATCCGACAT GTTCTGTGGT CGTGGTCCCG CTGCCTCATC CGGTGTCTGT GAAGTTGCTG CCGGTACTTC CCTGACTGTT
 401 GAGATGCACG CTCAGCCCGG CGCGCGCTCA TGCTCGCAGC CTGCCATCGG CGGCAACCAC TACGGCCCGG TTCTTATCTA CATGGCCAAG GTCAGCGATG
 501 CCAAAACCGC CAGCTCCGGC TCTTTCTTCA AGGTCGCCGA GGACGGATAC ACTGGTACCA CCGCCTCATG GGGAACTGAG ATCCTCAACG CAAACTGCGG
 601 CAAGCGCTCC TTCACTGTTC CCAAGAACAT TGCCTCTGGT GACTACCTTG TCCGCGCTGA GGCTATTGCC CTACACGCCG GCGTTGGAAG CCCTCAGCCC
 701 TACGTCAGCT GCTTCCAGGT CAAGGTTACT GGAGGAGGCA GTGCTACGCC CTCTGGTGTC AGCTTCCCCG GTGCCTACAA GCTCAGCGAC CCGCTCTTCA
 801 CAAAGGCTAT CTACGACTCG AGCTTCAAGT ACGTCAGCCC TGGCCCCGCT GTCTACTCCG GTTAGGAAAC TTGAATACAC ACGATTGTGA ACGAAAGAGA
 901 CGGGGAGATT GCGGTGGTTT GGTGAATTGA GACTTTTACA CAACGGGGAG AGATTTTTTG TACATAAAAC TTCTTCAACT CCATATTGCT TGGACCATTT
1001 TTACATACCA CATATATGCC ATTTGAAATG GGAATACCTT CTTCTTCTTC TTCTTCTTCT
```

>*AA9-15*(jgi|Cocsa1|138675|estExt_Genewise1Plus.C_4_t30477)

```
  1 AGCACATTCA GTCCATTCAT TACCTACTCC ACCAAACTCT CATTCTCCAA CTTTAATCAT GAAGTGCTTC GCCGTTGCCA CTGTAGCCCT CGCGGCTCTC
101 ACTGAGGTCG CCCAGGCTCA CTACATCTTC CAGACCCTGA CTGCCAACGG CGCCAAGGGA GGTCTGTTCC AGAACATCAG GCCTGTCCCC AACAACAGCC
201 CTGTCACCGA CCTGTCAGAC AAGAACCTCC GCTGCAACAC CAACGGAAAC AGTGGCAGTG GAACCACCAC TGTGGCGGTT GCTGCTGGCT CGACCGTCAG
301 CTTCACTGCC GACCAGGCTG TCTACCACCA AGGACCAGTT TCCTTCTACA TGAGCAAGGC CAGCTCTGCT GCCTCTTCAG ATGGTTCCGG TGACTGGTTC
401 AAGATCAAGG AAATTGGTCC AAACTTCTCC AGTGGACAGG CCGTGTGGGA CATGAGCACC ACCTACTCCG TCACCATCCC CTCCCAGGTC TCCGCTGGAG
501 AGTACCTCCT CCGTATCGAG CAGCTCGGTA TCCACAACCC CGGCAGCCCA CCACAATTCT ACATCTCTTG TGCACAAATC AAGGTCACTG GTGGCGGCAG
601 CGGCAACCCC TCTCCTACCA CCAAGATCCC CGGACACGTC AAGTCCACCG ACTCTGGCTA CACTGCCAAC ATCTACAGCA ACTTCAAGAG CTACGTTGTT
701 CCCGGCCCTA AGGTCGCCAC CTTCTAAGTG TGCTGAATGA CACGCGACAC GCCATTGAGC GAGCGCTACG ATGACCCGGG TACTTTTTTT CCCAGTTGGT
801 CTTGTTTCGA ACTAGAAGTT CGATTTCACT TCACTGTATA TACCTCTGTT GCATACCATT TCGATGTGTA TACATCTATA CCCTTACTTC AATTCAACCC
901 TTATCACGAT
```

>*AA9-16*(jgi|Cocsa1|34302|fgenesh1_kg.3_#_405_#_Locus4279v1rpkm25.64)

```
   1 AGAACGTTGC AAACATTCCG CCCGAGTGGA TCCCATGACA GCGCTGTGTG GGGGGGACGT GATGGGAATA GATGAAGTTC ATGTCTTGGG TCACCACTCC
 101 CAGCGTCGTC ATGGACAAGA CACCGAGTAT AAGTATGGCC CAGTTCGCCT TGCTGTAGAA ACCATCGCAG TCCACGACTT TCGATTCTCA CTCACTCATT
 201 CATTCATTCG CTTCTTTCAA CCTTCTCTTC TTTTGAACAT ATATTCAACT TGCTCTTTAC TCCTCCAACA TGAAGGCTGG TGCTCTCCTC GCTCTGCTTG
 301 TGGCGCCTAT GGCGGCCAAT GCCCACTACA TCTTCAACAT CCTCGTCGTC AACGGCCAGA CCATCGGTGG CGAGTATGCC TACGCTCGCA AGAACAGCAA
 401 CGCGTACAAC CCCGTCATCA CCAACGACAT TGTCAACTCC AACGACCTCC GCTGCAACAG GGGCGCTGTC GCTGGTAACA CCGGTACCTA CACCGTCAAG
 501 GCCGGTGACA AGCTTGGCTT CAAGATCTTC AACAACGAGA GGATCGAGCA TCCCGGTCCT GGATTTGTCT ACATCTCCAA GGCTCCCGGA AAGGTCAAGG
 601 ACTACGATGG CAGCGGCGCC TGGACCAAGG TCATGGAGAG CGGTCTTGCC AACCCCAGCG CCCCTGGTGT TGACAAGAGC TGGGCCAACT GGGACAAGGA
 701 TAGGCTCGAG TGGACCATCC AGAAGAGCAT TCCTGCTGGT GAATACCTTG TCCGTGTCGA GCACATCGGT CTTCACGAGG GTCACGTTGG CAAGGCCCAA
 801 TTCTACATGG AGTGCTTCCA GCTGAACATC CAGTCCTCTG GCACTGGAAC CCTCGGCCCC ACTGTTAAGA TTCCCGGTCT CTACAGCGCC AACGATGCCG
 901 GTATCAAGTT CAACAAGTGG AGCAACCCCA AGTCTTACCC CATGCCCGGC CCCAAGCTCT ACGCCGGCCA GTAAGCGATT CGCATTGCCA GCCAGCCGTC
1001 AAGCAAACAA GCAAGCATTT ATGCCAGCTG CCCAGCCAGC ACACAGTCGA GGAGGAAAGG GTGTTTTTGT TCATATCCGC CCCAGTGGCG CTTCATTTCT
1101 CTCTTAGTCT ACATTCCATA GCTATGATCA ATGTGACTAT GCGTTGCTTT CCGA
```

>*AA9-17*(jgi|Cocsa1|36406|fgenesh1_kg.6_#_329_#_Locus15854v1rpkm5.18)

```
   1 CACCAGCACT TCACTGCCAA CCTTCTTTCG CTTCTCCACA GTCTTTCGCT ACTTCATTCG TTTCTCAAGG CTAGTCCTTC CATACAATTC TTTTCACTTT
 101 CCAGTCCAAG TTCAAACAAC GACAAACAAC AACACCAACC TCAATCTTAA CCTCACCTCA ACATGAAGCT TTCACTCCTC GCCGCTGCTG CCATTGCGCA
 201 GATCGCCTCC GCCCACTACT TCTTCGAGCT TCTTGTCATC GATGGCAAGG AGACCAAGCC TTATGAGTTC GTTCGTGAGA ACACTCGTTC TATCAAGTAC
 301 AACCCCACCA AGTGGAAGAA CCCTCTCGAC AACATCACCC CCGATGACAC CGACTTCCGC TGCAACAAGG GTGCTTTCGC CTCCGCCAGC CGCACCGGCG
 401 TTGCTGAGGT CAAGCCCGGA ACCAAGCTCG CCATGAAGCT CGGTGTCGCC GCCACCATGA GGCACCCAGG CCCAATCCAT GTCCACATGT CCAAGGCTCC
 501 CGGCTCCGTC AAGGAGTACG AGGGCGACGG TGACTGGTTC CTGATCAAGC AAGCCGGTCT CTGCACCAAG GGAGACATCA AGGGGGACGC CTGGTGCAGC
 601 TGGGACAAGA ACAACGTTGA ATTCGATATT CCTATCGGCA CCCCTCCCGG TGAATACCTC GTCCGCTCCC AGCACGTCGG TGTCCACGGC GCCCACGATG
 701 GCCAAGCCGA GTTCTACTAC GGGTGCGCCC AGATCAAGGT TACCGGCGAC GGCGCCGGCG TCCCAGGCCC CATGATCAAG TTCCCCGGTG GCTACAAGAA
 801 GACGGATCCC TCCTGGAACT TCAGCGTCTG GGGCGGATAC AAGGACTTCC CCTTCCCCTC GCAGACTGTT TGGACTGGCG GTAACAGCGG CGGTGCTCAG
 901 TCTCCTGCTA CCTCGCCTTC TTCCTCTCCC TCTCCCTCTG CTGCTGCCAC CAAGCCCCAG GCTACTCCTG CTCCTCCTGC TCCCAAGCCC CAGCCTTCTC
1001 AGCCTGCTAC CCCTGTTGAG CAGCCTGCCG GTGTCCCGGT CACCCAGCCC AGCAGCGCCC CTGTTGAGCA GGCTCCTTCC GATGACTACC CTGCTGGAGG
1101 TGATTACCCT GCTGGTGATG ATTACCCCAC TGAGGAGGCT CCCAAGCCTA CTCCTGCTAC CCCATCCCCT GCTACTCCTG CCACCAGCAA GCGTCCTTGC
1201 AACGGTGGCA AGAAAGCCAG GCGCGGTAGC CGTGCTTTCC GCCAGTCTCT CCGCAAGTAA AAATGTGGAT GGGTTTCTGG AAATTCTTCG AAGCAAAGAC
```

1301 ATGACAGTTT TCTGATGTTG AAACGAAGAA GGGGGGTCGT TTGCTTCTGG AATTTCGAAA AAAACTGATG GTTGGAC

>*AA9-18*(jgi|Cocsa1|32133|fgenesh1_kg.1_#_296_#_Locus2463v1rpkm43.96)
1 CAGGCCAGTC CTTTCTTTGA ACACATTCAC TAAGTTCCTT TCTACCACAT TCATTCTTTA CCTTACATTC CATCTTTGTG ATCAGGCATC ACCATCACAC
101 ATTTGAAACC CAAACACTGT ACATAACACT TATTCAACAC ACGTCACCAT GTTCACCAAG GCCATCTTCA CGAGCGCTCT TCTCTCCGCC GTCTCGGCTC
201 ACCAGAACCT CCACCAGCTT TGGGTCAACG ATGTCACGCC CGGATACCAG GTCGGCATCC GGATGCCACC CTCCAACAGC CCCGTAGTCG ATTTGACGAG
301 CAACGACATG GCCTGCAACG TTGGCGGCTC CAAGGTTCCA TCTGGCGTCA AGACAATCGA GGCTAGCGAG GGTGACAAGA TCAAGGTCCA GTGGGATAAC
401 TCTGGCCATC CGGGACCGAT TACGCACATG CTGTTTGGCC CCGTCGATGA TGCTTCCCAA GCCACTGGTA TCGGAGCCGG ATGGTTCAAG ATTGACGAGA
501 TGGACTTTGT TGGTGGAAAG TGGGCTTCCG AGATCATGCA AGCGGCCAAC ATGTCGCATG AATTCACTCT TCCCACTGGC CTTGCCAGCG GCGAGTATCT
601 ACTCCGTTCA GAGATGCTGG CACTCCACTC TTCTCAGACA AAGGGTGGCG CGCAATTCTA TATCGGCTGC ATGCAATTGA AGATCAAGGG CACCGGTAGC
701 AAGGGCTCAT GCACACCTAC CATTTCGCTT CCTGGCGCCT ACAAGGCTGA CGATGCCAAC ATCTTCATCC CTAACTACTA CAACGGATAC GTTGCCACCA
801 ACTACAAGGC TCCTGGTGGT CCGGTTGCTA CTTGCGGTGG TGCTGGTGGC GCTGCTCCCG TTGCTCCTCC TGCCTCCAAC TCAACTGAGC CTGCTCCTTC
901 TTCAATTGCC GACGAGGCTG CTGCCCCTAC TCTTGCGCCC ACCGTCCCTT CTGGCAATGA TACTCTTCCA TCGGCTCCGG CTGTCCCCAG CACGTTCGCC
1001 ACCCTCCCCA GCCCATCTGC TGGCGTTACA GCTCCCGCCG GTGCTGTGCC CACTGGCTCT GTAGGAACTG CTAAGCTCTA CTACCAGTGC GGCGGAATCA
1101 ACTACAATGG TCCCACTGAA TGCGAGGGCA CTGCCAAGTG CGTCAAGCAG AACGACTTTT ACTCGCAGTG CATCAACTAG AGGAATTGAG ATGGCACAAT
1201 GCGCAGGAGG AGATGAGATG TGGTGGTGGT GAAGAATTGT TGGTGTTCTT GTACATATAA ACTTACGTAG ATGTTGACCT ACAATTTTAC TTTTGACATT
1301 ATTGA

>*AA9-19*(jgi|Cocsa1|164026|gm1.8747_g)
1 ATGATGGGGC TTTTGACAAC CGTGACCTCG TCACTTCTTC TGGCCGGCGT ATCGGCTCAC GCACGCATCG AGAAAATCAC AACATCAGCT GGCCTGGTTT
101 ACAATGGATG GGACCCCGTT TCCTCTGAAA CATTGACACC GCCTCGTCCA TTGGCTGCAT GGTCAGCCGT GAACATGGGC AACATCTTCG TGTCTCCCTC
201 ACACTTTAAT ACGTCAAACA TTACCTGCCA CGACAATGCC ATACCTGGTG CACTTCATGT CAACACGACT GCCGGCGATA CATTGCAGTT GAAATGGAAT
301 GAATGGCCAG TCTCCCACGT CGGCGCAGTC ATGACATACA TTGCCAAGTG CAACACGACT TGTTCAAAAG CAAACAAGAA CACCTTGTCC TGGGTCAAGA
401 TTGACGAGCT GGCATGGCTC AACAGTACCG GGTGGGACAC TCTGGAGTTG GGTGGCACAT GGGCGACGGA CGTACTCATT GCCAACAACT TCACATGGGA
501 AGTCAAGATA CCAGAAGTGC TGGCAGCAGG CTATTACACC CTGAGGCACG AGCTCCTTGC GCTGCACGTG GCAGAGCAGC TGGACGGTGC ACAGGCGTAC
601 CCGCAGTGTA TAAACTTGCG AGTAGACGGA GAGACAGGTG CAGAGGCAAA GCATTTAGAA GGAGGTGTAC TAGGGACCAA TCTATACAGG CCAACAGATG
701 CGGGTATACT GGTTGATGTC CATAAGAAGC TGACTGGATA CGATACTCCA GGTCCAAAGC TATGGGAGTA CGCAAGTCCA TTACGGCAGC CATACCAATG
801 A

>*AA9-20*(jgi|Cocsa1|140970|estExt_Genewise1Plus.C_6_t10336)
1 ATGAAAGCTT CTGTATTTCT CACAACAGCT ACGCTATCCG TGACTGCACT TGCGCATGGA GGTATTGATA AGTACATTAC TGGTGATAAG GTATATCAAG
101 GTTGGGAACC ATACAACGAC CCAACCGGAC AAAAGAGTAT ACAGAGACAG TACTCATCCT ACGATCCCCT GTTCATCAAG GATCTAACAA CTGTAAACAT
201 ACGATGCAAC AACAAGGGAC TATTAGGCAC CGGTGTGACA GGCACCATTG CCGCAGGCAC CAAACTGATA ACCCACTGGA AGCAATGGAC TCATAGGCCA
301 GCCACGTTTA TGGTCTACAT GGCCAAGTGC CCCGGCTCAT GCGATAGCTG GGACGGCTCA GGCAAAGTCT GGTTCAAGAT CTTCGAGCAA GGTCTCATAT
401 CCGGGACCGA AAACGCCGGT GTCTGGGCCG GCGACGCCAT CTTCGAAACT CTCAACGCAG CCATCACCAT CCCCAATGTT CTAGCTGGCG AGTACATGTT
501 CAGACACGAA CTCACTGCCC TGCACCAGGC AAACAATCCT CAATTCTACC CAGAATGCGC CCAATTCACC GTTACGGGCT CCGGTACCGC ATCGCCTCCT
601 GCATCATTCT TGGTGTCGTT CCCTGGCGCG TATACGGGGA CGGAACCCGG CCTTGCGTTT AATATTGACT CGGAGGATGC GAAGAAGGCG ACGAAGTATC
701 CTTTGCCCGG GCCGCAGCTG TGGGATGGTA CTAGTAGTGG TAATGCGACG ATGCCTGGAT CGCCGGTACC CTCGACGGCG GCTATGAAGC AGCGTGGTGC
801 TGGTACTTCG GGGCGGCTGC GCCGGTATGG GAGATGTTAA GATATACTAG GTTCGGTAGT TGTGTGAGTG GTTGATGTGC GTGGATGATG CTGG

>*AA9-21*(jgi|Cocsa1|33777|fgenesh1_kg.2_#_818_#_Locus56599v1rpkm1.15)
1 GCTTGACGCT CAACTTCTAT TCATTCTACA TATTAGATTC GGTCTTTGAC TTGTTCATTC TTTTCGACTT CTGCTTTGAA TAGCCACTCT TTCACAAGAC
101 AGGTTCTACA CTCGACCAAT TCATCAATCA ACCGCCCATA TCCTCACTCA AAATGAAGTC CTTCATCTCT GCTCTCGCTT TCGCCGGTGC TGCCAGCGCC
201 CACTGCACCA TCTGGGGTGT TTCCGTCAAC AACAAGGACC TAGGCTACGG CAACTCTCAG GGTGGATACA TTGACACGCC CCCCAACAAC TCTCCCGTCA
301 CCGATGTTAC ATCCAAGGCG ATGGAGTGCA ACGTAGCCAA CATCAAGGCG TCCAAGTCCA TCTCTATCAA CCCTGGTGAT GAAGTCGCTG TCCAATGGTT
401 CCACAACGGT CCCGGAGCCG GCGACCAAAT CATTGATGGA TCTCATAAGG GACCAATCAA CGTCTACATG TCGAAGGCTG GCTCTTCCAT GTCGTGGACC
501 AAGATTGCCG AGGATGGATG GGACGGCAAG TCGTGGGCTG TCACCAAGCT CCGCGATGGT GCGTACAACG GAAAGAAGGG ACAGCACACA TTCAAGATGC
601 CCAACGTTGC TGCCGGAGAC TACATCATCC GTCCCGAGAT TATTGCTCTC CACGAGGGTA ACAGGCCTTC GGGTGCCCAA TTCTACATGG GATGTATTCA
701 TGTCAAGGTT GGCGGATCCG CCAGTGGCTC TCTTCCTGCC GGTGTCTCGA TCCCAGGCTA CGTCACCGCT ACTAACCCTG GTGTCTTGTT CAACATCTAC
801 GGCTCATTCC AGAACTACCC CAACCCTGGT CCCAAGCTCT GGAGCGGTGC CGGTACTGCC ACTGCTGCTG ATGCTGCTCC CGCTCCCGCT CCTGCTGCCG
901 GTGCTCCTGC TGCCTCTCCA TCTCCCGCTA CCGGTGCTCC CTCTACTGAG GCACCTTCCA GTGAGGCTCC CGCTGCTGAG GAACCCGAGG AGTGCACTGA
1001 CGATACTGCA GAGCCTGCTG ATGAGCCTGC TGCTCCCGCT CCTGCTGCTC CCGCTGCCTC TGCTCCTGCT GCTGCTCCCG CTACCGGTGG TGCTGAGGCT
1101 CAGAAGTGGC ACCAGTGCGG TGGTCAGGGC TACACGGGCG CAACTGCCTG CGTTTCAGGC ACCTCGTGCG TCAAGCAAAA CGACTACTAC CACCAATGTA
1201 TGTAAATTGG CATGGTGGAT GACCATGTTG GGCGAGTCCA GAGGGACGGG TAGTATCGGT GGGCGGTATT TGTGGAGAAG TTTGGCTTGT AGAAGTTTGT
1301 TTCTATTTGC TGGTGGTGTC AAGTGTCAAG TGGGCTCGGC CGCGTAGGGC CGGGCTTGCG CACCGCTGGT TTTCGATTCT CATGTACATA TGTAAATTTC
1401 ACGACGAATT GGATGAAAAT GCCTCTTCGG TTCAATGCCT GTGCCCTTTT CCGACATGGA CCTCGTCATG TTAGTGTACC CACCTCTGCT GTGTAAGGAC
1501 CAAGAAGCTC GTCAGTGGCC AGGGGGCTAC GTGTCTAGAA GCCCCAGGTG CCACGCGTGA AGAGCTACGT CTCAGATCTA AGCTGACCGG CCAACCCGGC
1601 AATGACATGA GGGGCCATCG GAGAGCGAGA GAGTGAGGCC GTGTTGCA

>*AA9-22*(jgi|Cocsa1|34692|fgenesh1_kg.3_#_795_#_Locus2472v1rpkm43.79)
1 GGGGGAAGGG GGACCGAAGA AGCGTCTGGA TCTCCTTGTG CAATCATTTC AGCCCTGGCT CCGACTATAC ATAAGGTCTT TTCGTTCCCC GTCCAGTCTC
101 GTTTTTCTTC ACGCTCCAGC AAGCAAGTCA TTCTTTCCGC AGTCAGTCTT TGACCAGTCA CTCTTTCATA GCCAGGTGCT ATCAAAAACT CGCTCTTCGT
201 CATTCTTTTT ATACCCTTTT GCGTTATCTC GCCAACAATA CCCCCAACGA GCCGTCTTTT GCAAACTACC GCCATTATGT CTATGTCCAA GATCTTCCAG
301 ACCACTGCTC TTGTCGCAGC CCTCATTTCC TCCGTTGCCG GCCACACTTC CGTTGAGAAG TTTGAGGCTG GAGGCAAGAC TTATGAAGGC TACCGTCAGG
401 CGAGCAAGGT CGATCCCGGT AACCAGTCTC CTGCTTGGTG GACGGCCCAA GGCTGGGGAT ACCAGCCCGT CTACGGCGAT AAAATTGCGC ACCCCGACAT
501 CATTGCTCAC AAGGACGCTT CGCCCTCGCC TTACACTGCT CCAGTCCCCG CTGGATCTGA TGTCACTTTC CACTGGCACC ATGAAGGCAG CTGTGGCGGC
601 GGCGAAGAGG GCTGGGACTG CTCCCACCAC GGCTGGACTG CCACTTATCT CGCCCCTTGC AACGGCGACT GTGCCAAGGT CGACAAGACG ACTCTCCAGT
701 TCTTCAAGAT CCATGAGTCC GCCCTGATCG ACTACCGCAA GGGCCGCTAC TCGTCCGGCT CGCCCCAAGA ACAAACCGGT TACTGGGGCA CCGACGCCAT
801 CTTCTACGAC AACCGCAACA CTCAGACAGT CAAGATCCCA TCTGACATTC CCAGCCAAAA CTACGTCCTC CGAACCGAGG TCGTCTCCAT CCACAACAAC
901 GGTGATGTTA GCAACCGCCA GTTCTGGCCT CAAGCCTTCA ACATCAAGGT GACTGGCGGT AAGAATGGCG CCAAGGTCCC TGCTGGAAAG AAGGGTACCG
1001 AGCTGTACAA GGCCAACGAC CCGCTCCTTA AGTGGGACCT TTACTGGCAC AAGGCTGGCC AGACTTCCCC AGATGCTCCT GGTCCTCAAA TCGCATCTGT
1101 TGCGTCCAGC AACTCCAGGC GCCACGCCAG GGACTTTTCT TCTTAGACGA TCTTCTTCAT CTTTCTTCAA GACTTCTTTT ATGGACATGG TTTCGGGTGG
1201 ACTATGATCT TGGTTCTATG ATCAAGACAA AAACGGAAAA CGGCGCGAGT TTGCTTCAGC GAGGGTGTGT GACGCACTAT TCTTCTTCTT ATGTTCCTTT
1301 GTGGAACTGG TTTTGAGAAC ACGACGCTTG CGGCACGATC TCATGATTTT TCTTCTTCTC TATAGATACT TATACTGCAT CCCAGCGGAT GTGAATGCAA
1401 ATGAAAATGC ATTTTCTAGG CTTCGAGCCC AA

>*AA9-23*(jgi|Cocsa1|184710|estExt_Genemark1.C_150184)
1 GCTATAAATG TCACCATGAG GATCTCCAGA AATACCTCGA ATTGTGCTCA TCCATCTCTT CAGGTTTCCT TGTCCTCACT ATCATGATGG CTCATAACTG
101 CGCCCTCATC CTCTTGCTCC TCGCAAGCCA AGCATGCGCC CACACATTCA TCTGGGGCGT CTTCGTCAAC GGCATCGATC AAGGCACCTT CACCGGCGTC
201 CGAATACCTG GATACAACGG TCAAAACGGG AAAGGCGGCT ACAATAACTC CCCCGTCAAG GACCTCTCCT CTATCGACAT GCGCTGCAAC GTCATGGGCG
301 ACGTCCAAGC CCACGACACC ATCAAAGTAG CTCCAGGCGA TAACCTGACT TTCGACTGGC ACCACGAGCT ACGCAACAAC ACCGACGAAG TAATCGCATA
401 CTCGCACCAC GGTCCCTCAC TCATCTACAT CTCGCCAGAC CCACCAACAA ACACGTCCTT CGTCAAACTC TGGCACGCCG GTAAAATCGA AAGCAATCCC
501 TTCCCGCAAG CAGGTAAATG GAGTACCACA GCCGACATCC GCGCGAACTA CGGACGCATG AATGTGCGCA TACCCGCGGG CCTGAAAGCA GGACCGTACC
601 TAATCCGCGC TGAGATGATC GGCCTGCATG AGGGCGACGT GAGCTTTGCA CAGAATCCGC GGAGAGGAGC GCAGTTCTAT CCGGATTGTG TGCAGATTGA
701 GGTTGTGGGG GGTGGCGAGG TCGAGTTGCC CGAGGGAGTC GGGTTTCCTG GGGCGTATTC GTATGAGGAT CCGGGGGTTG TGCATAATAT CTATTGCTCG
801 ACTGAAACAA AGAAACCAAC AAGGTCCGCC GCCCCCTGTC CCACCGACTA CGTTATCCCA GGCCCGGCGG TATGGTCTGG TGCGTGGCCA GAAACAACTG
901 CCGTTGCGTT GGGCCCCATC AAGGGTGTTT CGACGGCCAC GCCGTGGAGC ACGTGGATTG GGACGAATAG CGTGGTGACG AGCGCGACGT ATGTGGATGC
1001 GAAGAGTCAG ACTATTGTGG GGAGTTCAAA GTATACGGCT ACGTGGAGTG AGACGTATGC TTCGCCTACT GCGACGGCGC CGCGGTGGTA GAAGGTGAGA
1101 TGAACGTGGG GTGCGAGGTA TTTGGTATAT GGGAGTAAAG CTTTTCGGAA T

>*AA9-24*(jgi|Cocsa1|155289|gm1.10_g)
1 ATGAAGATTC TGCTGGCCAT CAGCTTCCTC GGGCACCTGT CCCACCTCAC TCAAGCGCAC TACCACTTCC CACATTTTGT AATCAACGGT CAAGTCACTG
101 GCCCACACCA GTATGTTCGC GAGCACGACA ACGGTTTCCA GCCTTCATGG GATGAAGGCA GATTCATTAA CAGCGAACAT CTCCGCTGCA ACAAGGGTTC
201 CATGAATCAC CGCACTCAGC CAAAGACCTA CAAGGTCAAG GCAGGCCAAG ATGTCATTGG TTTCCAAGCA TCTATTAACA CAGAATTATT CCATCCAGGC
301 CCCGTACAGA TTTATCTCTC GAAAGCACCC GGCGATGTTC GTGACTACGA TGGCTCTGGT GACTGGTTCA AAGTCTACCA GCTTGGACAT CAGCCTGGCT
401 CTACGGCTGA TTCGGCATGG CTGTCGTGGC ATAAGAAGCA GTTCACCTTC AAGCTCCCCG CCGAGATCCC AGCGGGCCAG TACTTGATGC GAATCGAGCA
501 GATTACCACT CATCCTCCCT ACACGAACCG AGAGTTTTAC ATTCAATGTG CGCACATCGA AGTTGCCAGC AGCTATAACG GTTCCCCCTC GCCTACGTTC
601 AAGATTCCGG GAGTATACAA CCGTGAGCAG CCATTCTTCA AGTATGACAG CTGGGCAAAG CCACAGCCGA CTGTTTGCCC GCTCCCTGGA CCCGCTATGT
701 GGCCAAACAA TAACAATTTG AATAAGATTC TCTAA

Supplementary figure S2. Genomic architecture of of *B. sorokiniana* GHs and redox enzymes (AA9). The figure shows the schematic of arrangement of introns and exons in each of the *BsGH* homologs (comparative lengths are unscaled).

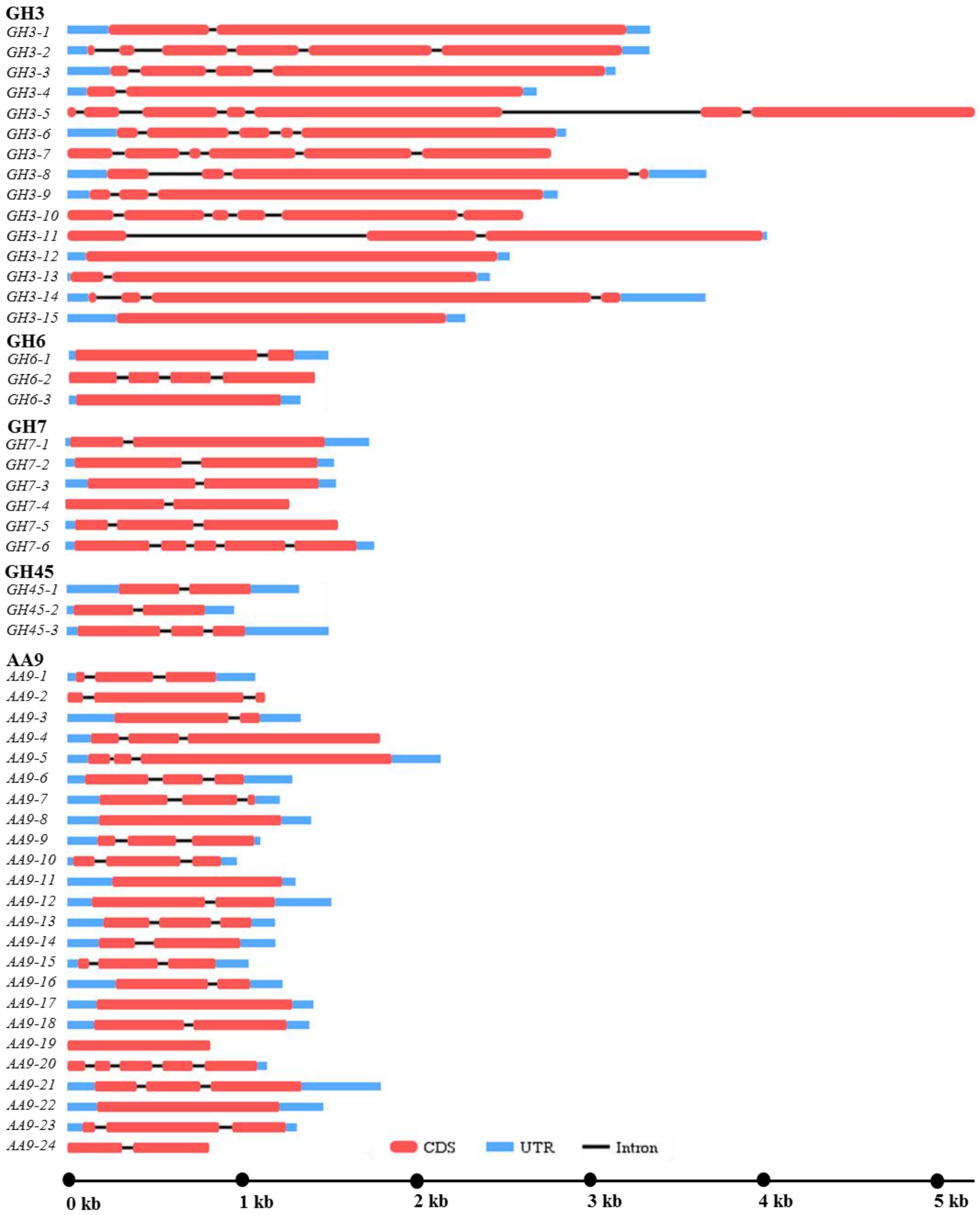

Supplementary figure S3. Multiple sequence alignment of protein sequences of *B. sorokiniana* GHs and AA9.

```
GH3-9             ---MSS------------------------------------------------------------------------------------------------------------------------------------------------      3
GH3-6             ---MRFADA--------------------------------------------------------------------------------------------LALAIA--------LFP---------GLSAAQG----------------     22
GH3-5             ---MRSA-------------------------------------------------------------------------------------------IFP-LLATA--------------------ALSTAI-----------------     18
GH3-10            ---MIRY------------------------------------------------------------------------------------------------------------------------TFLLFHI------------AFAS     15
GH3-13            ---MHFLSTA-------------------------------------------------------------------------------------------VVLGAS--------------------LISKSHA----------------     20
GH3-11            ---MDLMLRDYLQS-----------------TR-----ENTSAELDPEF------------------------------------------LTNAITQVKTLLIAGSGTTSDTV---------CFGHMLLSVHP-----------KVVRK     63
GH3-14            ---MAKIEQMDPV----------------------------WDDLDRT--------------------------------------------------MGQLFMMGFDGTSVTPHIRTLIEEHHVGCILLTAKN----------------     53
GH3-1             ---MAPYEAHELRRGKYAKLDAPVDDDLETPTRFSSESNETLEDLEEHDPLAFDSKLKRKKSAGDLKVRKASYRDTEAVTPFPTQQRRSKCAPGRCCCWLLVFIAAT--------VL----------LLASGGI----------------    113
GH3-12            ---MRSPLYS----------------------------------------------------------------------------------------VASLLVGYI--------L-----------YLPSSAN------------GLAR     28
GH3-15            ---MVARNRSQRK----------------------------------------------------------------------------------LNTFTTLFFAAT--------------------AMSSAIRT----------PSFGK     35
GH3-3             ---MASL----------------------------------------------------------------------------------------------LFLSLS--------------------GLAAGAA----------------     17
GH3-8             ---MATINLGNASP---------------------------TDDIKQID------------------------------------------LKHPAIQIQPLTLDTP--------------------LLTPAAT----------------     42
GH3-4             ---MAPS-------------------------------------------------------------------------------------------VVSKVLSGS--------------------ALLAAVSAQNFGGGGRGEDAFSY     36
GH3-2             ---M-------------------------------------------------------------------------------------------------LWLAQA--------LLV---------GLAQASP------------RFPR     21
GH3-7             ---MAFIFATR---------------------------------------------------------------------------------------ILLVLLSTF--------------------SLSSAQI----------PDGLPI     30
GH6-1             ---M---LSN-------------------------------------------------------------------------------------------VFLTAA--------------------LAAGLAQ----------------     17
GH6-2             ---MKGL------------------------------------------------------------------------------------------------------------------------LSATVAS----------------     11
GH6-3             ---MVQVTQ--------------------------------------------------------------------------------------------FVLATA--------------------ALAGAAA----------------     19
GH7-4             ---M--------------------------------------------------------------------------------------------------------------------------------------------------      1
GH7-3             ---MSLS----------------------------------------------------------------------------------------------SVVAA---------------------TLLGLAT----------------     16
GH7-1             ---MYRT----------------------------------------------------------------------------------------------LAFASL--------------------SLYGAAR----------------     17
GH7-5             ---MYRT----------------------------------------------------------------------------------------------LVVTA---------------------SFLHAAR----------------     16
GH7-6             ---MSF-----------------------------------------------------------------------------------------------VSVTA---------------------AFLALSA----------------     15
GH7-2             ---MARY----------------------------------------------------------------------------------------------ALLAA---------------------ALLGFTV----------------     16
GH45-1            ---M-------------------------------------------------------------------------------------------------VSLKQL--------------------SFVVL------------------     12
GH45-3            -----------------------------------------------------------------------------------------------MARHVRNTLISL--------------------HLFAL------------------     17
GH45-2            ---------------------------------------------------------------------------------------------------MLLTLARL--------------------SLAF-------------------     12
AA9-7             ---MKY------------------------------------------------------------------------------------------------LVPTL--------------------AFAATAA----------------     15
AA9-9             ---MLFSN---------------------------------------------------------------------------------------------IFLPTA--------------------LLAAQAA----------------     18
AA9-15            ---MKCFAV--------------------------------------------------------------------------------------------ATVALA--------------------ALTEVAQ----------------     19
AA9-10            ---MRLS----------------------------------------------------------------------------------------------TPLVLA--------------------AVASSTT----------------     17
AA9-20            ---MKAS----------------------------------------------------------------------------------------------VFLTTA--------------------TLSVTAL----------------     17
AA9-6             ---MHASTL--------------------------------------------------------------------------------------------LLGAAA--------------------LLVENVS----------------     19
AA9-24            ---MKILL---------------------------------------------------------------------------------------------AISFLG--------------------HLSHLTQ----------------     18
AA9-19            ---MMGLL---------------------------------------------------------------------------------------------TTVTSS--------------------LLLAGVS----------------     18
AA9-2             ---MKNTL---------------------------------------------------------------------------------------------LLTSIS--------------------AFATHVA----------------     18
AA9-23            ---MMAHN---------------------------------------------------------------------------------------------CALILL--------------------LLASQAC----------------     18
AA9-4             ---MKT------------------------------------------------------------------------------------------------VATIA--------------------ALAAMAN----------------     15
AA9-1             ---MKV------------------------------------------------------------------------------------------------SAVIL--------------------AVASLAS----------------     15
AA9-5             ---MKTQS---------------------------------------------------------------------------------------------ILFAAL--------------------ASAPAAL----------------     18
AA9-11            ---MFA-----------------------------------------------------------------------------------------------KSAVIL--------------------AIANLAA----------------     16
AA9-12            ---MKF-----------------------------------------------------------------------------------------------SAALIA--------------------AVAQVAS----------------     16
AA9-3             -----------------------------------------------------------------------------------------------------MLSLLV--------------------LLAPLVA----------------     13
AA9-18            ---MFT-----------------------------------------------------------------------------------------------KAIFTS--------------------ALLSAVS----------------     16
AA9-13            ---MKF-----------------------------------------------------------------------------------------------FAAAIA--------------------LLPALGS----------------     16
AA9-21            ---MKS------------------------------------------------------------------------------------------------FISAL--------------------AFAGAAS----------------     15
AA9-16            ---MKA------------------------------------------G----------------------------------------------------ALLALL--------------------VAPMAAN----------------     17
AA9-22            ---MSMSKIFQ------------------------------------------------------------------------------------------TTALVA--------------------ALISSVA----------------     21
AA9-17            ---MKL-----------------------------------------------------------------------------------------------SLLAAA--------------------AIAQIAS----------------     16
AA9-8             ---MYKTLAS-------------------------------------------------------------------------------------------TITAIA--------------------ALAATAT----------------     20
AA9-14            ---MKY------------------------------------------------------------------------------------------------STIFL--------------------AAATTAS----------------     15
GH3-Alteromonas   MDSMSHAAQ-----------------------------------------------------------------------------------------TKSHVVETTDRS--------------LGQRLILDLR----------------     31
GH6-Alteromonas   ---MNILKNRPSRS-----------------------------RMDTFN--------------------------------------------GGIGHWLKASVAGI--------------------ALTTLAA----------------     38
GH7-Bacterium     ------------------------------------------------------------------------------------------------------------------------------------------------------
GH45-Alteromonas  ---MNISQL--------------------------------------------------------------------------------------------KFIPAA--------------------ALLFAYA----------------     19
                  1.......10........20........30........40........50........60........70........80........90.......100.......110.......120.......130.......140.......150
```

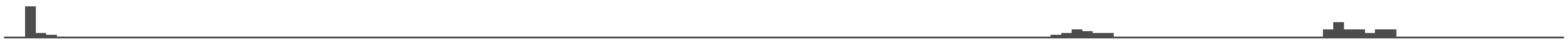

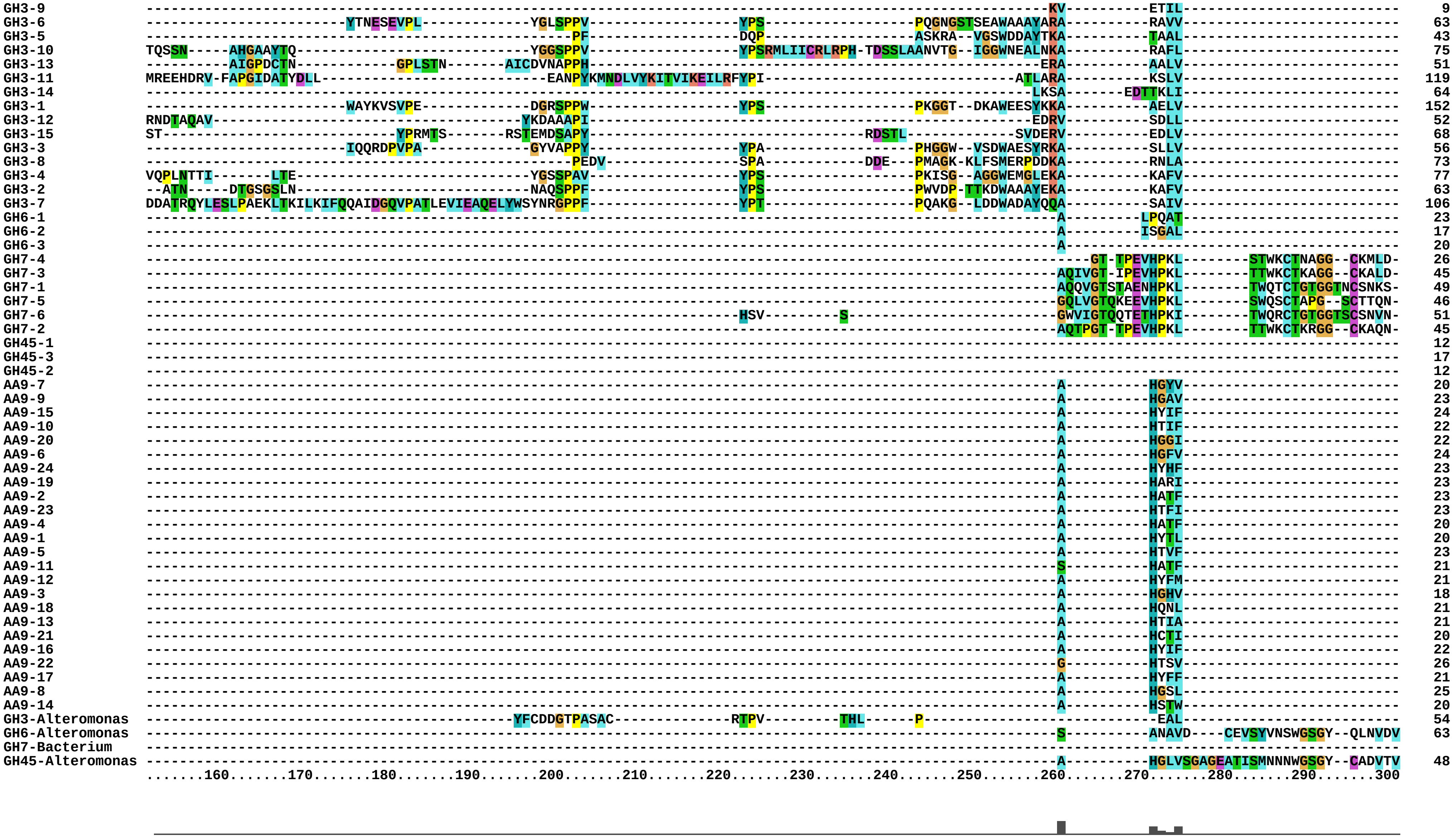

GH3-9 9
GH3-6 63
GH3-5 43
GH3-10 75
GH3-13 51
GH3-11 119
GH3-14 64
GH3-1 152
GH3-12 52
GH3-15 68
GH3-3 56
GH3-8 73
GH3-4 77
GH3-2 63
GH3-7 106
GH6-1 23
GH6-2 17
GH6-3 20
GH7-4 26
GH7-3 45
GH7-1 49
GH7-5 46
GH7-6 51
GH7-2 45
GH45-1 12
GH45-3 17
GH45-2 12
AA9-7 20
AA9-9 23
AA9-15 24
AA9-10 22
AA9-20 22
AA9-6 24
AA9-24 23
AA9-19 23
AA9-2 23
AA9-23 23
AA9-4 20
AA9-1 20
AA9-5 23
AA9-11 21
AA9-12 21
AA9-3 18
AA9-18 21
AA9-13 21
AA9-21 20
AA9-16 22
AA9-22 26
AA9-17 21
AA9-8 25
AA9-14 20
GH3-Alteromonas 54
GH6-Alteromonas 63
GH7-Bacterium
GH45-Alteromonas 48
.......160.......170.......180.......190.......200.......210.......220.......230.......240.......250.......260.......270.......280.......290.......300

```
GH3-9          --------------------QSLTLEEKITLLAGKD-------------------------------------------FWETVPIPEK------------------------------------------------------------   35
GH3-6          --------------------AQLTQEEQSNLTHGVT----------------------------------------GRCVGQTGAVPRL------------------------------------------------------------   92
GH3-5          --------------------AKLSQDDKIGIVTGTGWSK-------------------------------------SNCVGNTKAASSI------------------------------------------------------------   75
GH3-10         --------------------DNLTLEEKSLIVTGAA----------------------------------------GPCVGNIAPIPRL------------------------------------------------------------  104
GH3-13         --------------------AAMEPQEKLDNLVSKS-----------------------------------------------KGVSRL------------------------------------------------------------   73
GH3-11         --------------------ALYTLEEKINATSNSA-----------------------------------------------PGVARL------------------------------------------------------------  141
GH3-14         -----------------FELQKIAHD---------------------------------------------------------------------------------------------------------------------------   73
GH3-1          --------------------KQMTLPEKVNITTGTGWSM-------------------------------------DLCVGQTGRVDRL------------------------------------------------------------  184
GH3-12         --------------------SRMTIEEKTAQLIQGDISN----------WINTTTNAVNQ-TGLEWNFRVR-----AGQFYVGYPMPAE------------------------------------------------------------  105
GH3-15         --------------------QRMTVEEKAGQLFHNIINQ----------GPNGTLLNNTR-ESVEGKLMTH--------FNLHGPIADVRAT---------------------------------------------------------  121
GH3-3          --------------------SNMTLAEKTNLTAGTGIFM-------------------------------------GRCVGNTGSALRV------------------------------------------------------------   88
GH3-8          --------------------AKLTLEEQISLLAGAD-------------------------------------------FWRTVAIPEK------------------------------------------------------------   99
GH3-4          --------------------AQLTLEEKADMVTGQA----------------------------------------GPCVGNIVAIPRL------------------------------------------------------------  106
GH3-2          --------------------SQLTLIEKVNLTTGTGWQS-------------------------------------DHCVGNVGAIPRL------------------------------------------------------------   95
GH3-7          --------------------DQMTIEEKVSVVSGQTSTT-------------------------------------NGCSGQIPGVPRL------------------------------------------------------------  138
GH6-1          -----------------------------------------------------------------------------------------------------------------------------------------------------   23
GH6-2          -----------------------------------------------------------------------------------------------------------------------------------------------------   17
GH6-3          -----------------------------------------------------------------------------------------------------------------------------------------------------   20
GH7-4          --------------------TSIVIDSLRHK--------------------------------------------------------------------------------------------------------------------------   37
GH7-3          --------------------TAIVVDSLRHN--------------------------------------------------------------------------------------------------------------------------   56
GH7-1          --------------------GSVVLDSNWRW--------------------------------------------------------------------------------------------------------------------------   60
GH7-5          --------------------GEVVVDANWRW--------------------------------------------------------------------------------------------------------------------------   57
GH7-6          --------------------GEIVIDANWRW--------------------------------------------------------------------------------------------------------------------------   62
GH7-2          --------------------TAIVTDSAAHP--------------------------------------------------------------------------------------------------------------------------   56
GH45-1         -----------------------------------------------------------------------------------------------------------------------------------------------------   12
GH45-3         -----------------------------------------------------------------------------------------------------------------------------------------------------   17
GH45-2         -----------------------------------------------------------------------------------------------------------------------------------------------------   12
AA9-7          --------------------SNATIG-------------------------------------------------------------------------------------------------------------------------------   26
AA9-9          --------------------TSYVID-------------------------------------------------------------------------------------------------------------------------------   29
AA9-15         --------------------QTLTAN-------------------------------------------------------------------------------------------------------------------------------   30
AA9-10         ----------------------LSLN-------------------------------------------------------------------------------------------------------------------------------   26
AA9-20         --------------------DKYITG-------------------------------------------------------------------------------------------------------------------------------   28
AA9-6          --------------------KAVNIK-------------------------------------------------------------------------------------------------------------------------------   30
AA9-24         --------------------PHFVIN-------------------------------------------------------------------------------------------------------------------------------   29
AA9-19         --------------------EKITTSA------------------------------------------------------------------------------------------------------------------------------   30
AA9-2          --------------------QDLWVN-------------------------------------------------------------------------------------------------------------------------------   29
AA9-23         --------------------WGVFVN-------------------------------------------------------------------------------------------------------------------------------   29
AA9-4          --------------------QQLWKN-------------------------------------------------------------------------------------------------------------------------------   26
AA9-1          --------------------PSIN-G-------------------------------------------------------------------------------------------------------------------------------   25
AA9-5          --------------------TDFYVD-------------------------------------------------------------------------------------------------------------------------------   29
AA9-11         --------------------QSFVVN-------------------------------------------------------------------------------------------------------------------------------   27
AA9-12         --------------------DKAIVG-------------------------------------------------------------------------------------------------------------------------------   27
AA9-3          --------------------DKVVAD-------------------------------------------------------------------------------------------------------------------------------   24
AA9-18         --------------------HQLWVN-------------------------------------------------------------------------------------------------------------------------------   27
AA9-13         --------------------QRVRVN-------------------------------------------------------------------------------------------------------------------------------   27
AA9-21         --------------------WGVSVN-------------------------------------------------------------------------------------------------------------------------------   26
AA9-16         --------------------NILVVN-------------------------------------------------------------------------------------------------------------------------------   28
AA9-22         --------------------EKFEAG-------------------------------------------------------------------------------------------------------------------------------   32
AA9-17         --------------------ELLVID-------------------------------------------------------------------------------------------------------------------------------   27
AA9-8          --------------------TGVSLD-------------------------------------------------------------------------------------------------------------------------------   31
AA9-14         --------------------QQLWVG-------------------------------------------------------------------------------------------------------------------------------   26
GH3-Alteromonas --------------------KRLLVDYNIGGVILFSENI----------VSAQQLLTLN--------------------YTMQAAMAEA------------------------------------------------------------   93
GH6-Alteromonas TNNDSSSVSDWEITLEFANAPQVTNSWNVSLSTSGNTVTATDSGWNGNLNANGGSTSFGFQGSSNGSFETPTCVGSNGSQPTATPVPTAEPTPTPVPTTEPTPTPVPTTEPTPTPVPTTEPTPTPVPTTEPTPTPVPTPVPTTEPTTDPG  213
GH7-Bacterium  -----------------------------------------------------------------------------------------------------------------------------------------------------
GH45-Alteromonas ANNGGSTITDWTLELDL---NGANINNLWNGSNSGSTVTPQSH--NAS-IASGSSVTFGYCANGSGTPSIASLSVTGGGTASSSSSSSVSSSSSSSSVSSSVSSSSSSVSSSVSSSVSSSSSSSVSSSS-----------SSQSSSSTG  181
               .......310.......320.......330.......340.......350.......360.......370.......380.......390.......400.......410.......420.......430.......440.......450
```

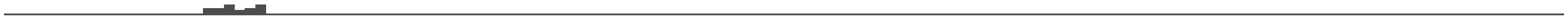

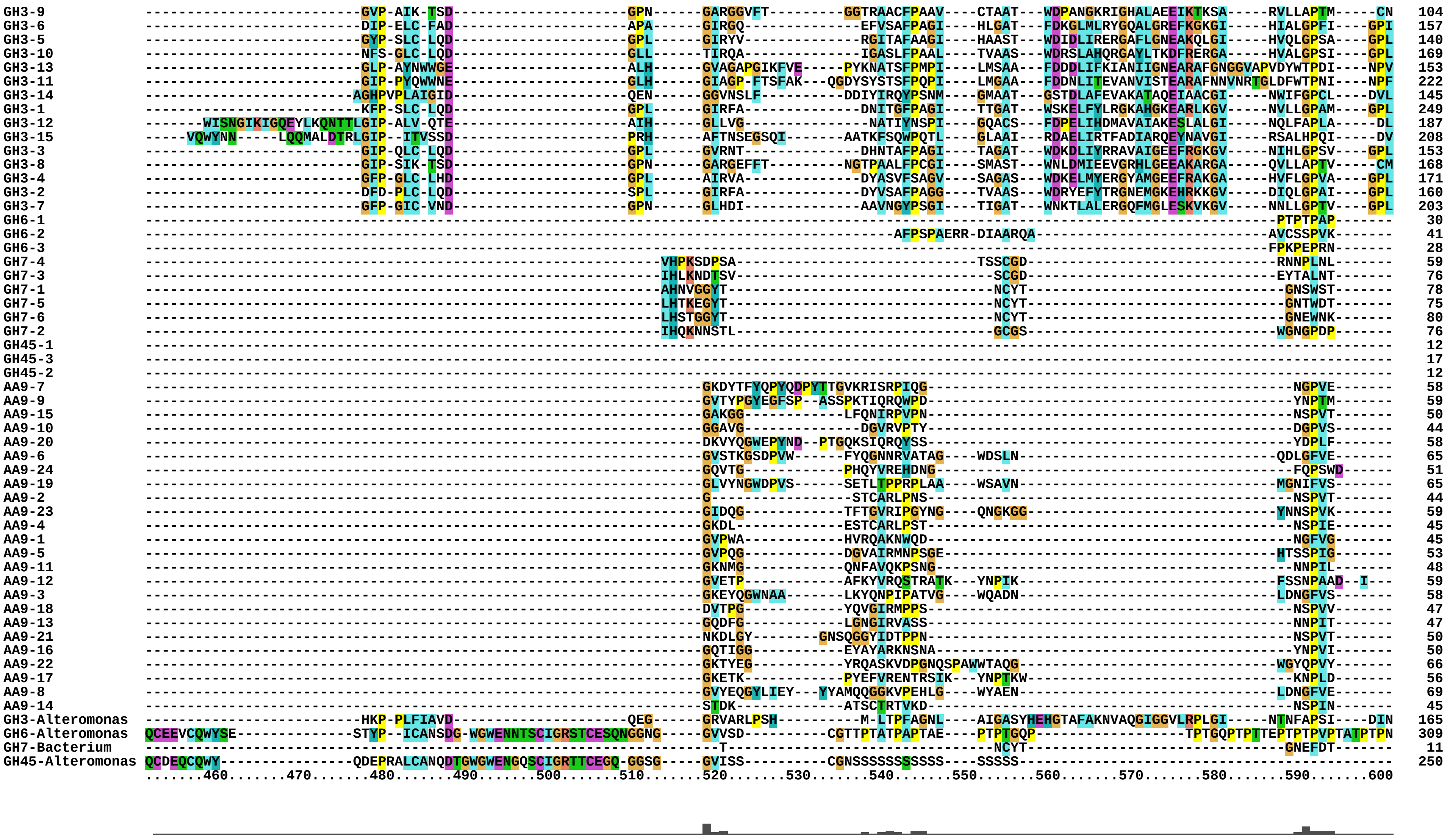

GH3-9 104
GH3-6 157
GH3-5 140
GH3-10 169
GH3-13 153
GH3-11 222
GH3-14 145
GH3-1 249
GH3-12 187
GH3-15 208
GH3-3 153
GH3-8 168
GH3-4 171
GH3-2 160
GH3-7 203
GH6-1 30
GH6-2 41
GH6-3 28
GH7-4 59
GH7-3 76
GH7-1 78
GH7-5 75
GH7-6 80
GH7-2 76
GH45-1 12
GH45-3 17
GH45-2 12
AA9-7 58
AA9-9 59
AA9-15 50
AA9-10 44
AA9-20 58
AA9-6 65
AA9-24 51
AA9-19 65
AA9-2 44
AA9-23 59
AA9-4 45
AA9-1 45
AA9-5 53
AA9-11 48
AA9-12 59
AA9-3 58
AA9-18 47
AA9-13 47
AA9-21 50
AA9-16 50
AA9-22 66
AA9-17 56
AA9-8 69
AA9-14 45
GH3-Alteromonas 165
GH6-Alteromonas 309
GH7-Bacterium 11
GH45-Alteromonas 250
.......460.......470.......480.......490.......500.......510.......520.......530.......540.......550.......560.......570.......580.......590.......600

```
GH3-9               HRHPLGGRNFESFSEDPYLAG-----------------KMAANVVNGLQE------KGIAATIKHFAANEQETDR------------------------------------------LRVNEIV-SERALREIYMKPFEITVKEANPLAV   188
GH3-6               GRVVRGGRNWEGMGADPYLNG-----------------VGGGLLTRGTQE------AGVMATPKHWIAQEQEYRRRPGSEG------------------------------------EAVSSNV-DDRTLHELYAWPFMDALR-EGAVSV   246
GH3-5               GKFAKGGRNWEGFGSDPYLQG-----------------IMMAQTIEGMQE------AGVQATAKHWIVNEQELNR------------------------------------------DTMSSDV-SDRVLRELYVWPFADAAH-SKVAAF   223
GH3-10              GRSPFEGCNWEGFSPDAFLSG-----------------TLVEDTIKGMQS------TGVQACVKHFIANEQETKRMPSVNN-------------------------------NGVTIDAVSSNV-DDKTMHETYLWPFSNAIR-AGSASV   263
GH3-13              -RDIRWGRASESPGEDIRRIK-----------------GYTKALLAGLEGDQAQ--RKIIATCKHYVGYDMEAWG--------------------------------------GYDRHNFSAKI-TMQDLAEYYMPPFQQCARDSKVGSF   244
GH3-11              -RDPRWGRGQETPGEDSYHLS-----------------SYVKALIHGLQGNETDPYRRVVATCKHYAGYDIENWN--------------------------------------GNLRYQNDVQI-SQQDLVEYYLAPFEACVQ-ANVGAF   314
GH3-14              TNARNQPLGVRTAGDDPQEVS-----------------AFGCAFMQGYKE------GGVVTLGKHFPSYGNLEFLGSTLDVPIITE---------------------------------------SLEQLSLSALIPFKKAIE-RGLDSM   232
GH3-1               GRMPAGGRVWEGFGSDPVLQG-----------------IAAAATIKGIQD------EGVMATAKHYIANEQEHFRQAWEWG----------------------------------LPNAISSNI-DDRTLHEIYAWPFADSVK-AGVTSI   340
GH3-12              ARELRFGRVEETYGEDPFLAG-----------------EMAYEYVKGVQS------LNVSATVKHFAGFSAPEQGLNTGPVHG------------------------------------------GERELRTTWLPPFHRAIIDADAWNI   272
GH3-15              ATEPRWARIGGTMGENATLTA-----------------ELTVAYIEGFTGPNGFGHDSVTTVSKHFPGSGPVEGGEDSHFT-------------------------------YGKNATYPGNNF-------EHHLIPFKAAIA-AGTRQM   302
GH3-3               GRKPRGGRNWEGFGSDPVLQA-----------------FGGALSVEGIQS------TGVIATIKHLIANEQEMYRMWNLVK------------------------------------PGISSNV-DDRTLHELYLWPFAEGVR-SGVGSV   242
GH3-8               HRSPLGGRNFESYSEDPFLTG-----------------KLAASYVRGLQS------KGVAATIKHFLGNEQETER------------------------------------------QAYDAII-AERPLREIYLKPFEIAVRDASPWAL   252
GH3-4               GRSAYAGRNWEGFSSDPYLSG-----------------VAMEKTILASQD------AGVQACPKHWIGNEQEIMRNPTYRK----------------------------DDNTEQAQAAISSNI-DDRTMHELYMWPFANAIK-ARAASI   268
GH3-2               GRHPKGGRNWEGFSPDPVLSG-----------------IAVAETVRGIQD------AGVIACTKHYLLNEQEHFRQPGGFK-------------------------------DIPFVDAISSNT-DDKTLHELYLWPFADAVR-AGTGSI   254
GH3-7               GRVVMGGRNWESFSVDPYLCG-----------------IMGAQTVLGMQE-------HVIATAKHFILNEQETNRNPSSFG---------------------------------LGNASVSATV-DDKTMHELYLWPFQDLVK-AGVGSV   294
GH6-1               --------SGNPFAGKNFYANPYYSSE----VHTLAMPSLPASLKPA-AT------AVAKVGSFVWMDTMAKVPLMDTYLA----DIKAKNAAGANLMGTFVVYDLPDRDCAALASNGELKIDDGGVEKYKTQYIDKIAAII--KKYPDV   155
GH6-2               -------LSGNPFSNRSLAVNSFYAAE----VKEAVTKISDSALAAK-AA------KVAEIGSYFWIDTRDKIKMLDDIIK----------GTPCNQIRGLIIYDLPGRDCAAKASNGELAV--GEINIYKTQYIDPIVAII--KKYPNI   159
GH6-3               -------TTVNPWIGKDRYVVESYGKKLDQTISSFLAKNDPLNAART-RT------VAKKTSTFVWVTSRAGLSQIPEAIQ----QARRQRKGRKRMIVGLVLYNLPDRDCSAGESAGELSSAKDGLNIYKHEFVDKYAQLV--SEAKDL   158
GH7-4               -----------TVCPDKATCA-----------QNCVIEGIQDYSTQAVFT------DG-GKLRLDMFNPSGEY-------------------------------------------------------------MSPRVYLL--------   111
GH7-3               -----------ALCPDKKTCA-----------QNCVIDGIEDYSTQAIFT------DN-DKLRLDMYNPKGEY-------------------------------------------------------------MSPRVYLL--------   128
GH7-1               -----------QYCADGDSCT-----------KNCAIDGADYSGTYGITT------SN-NALSLK-FVTKGSF----------------------------------------------------------SSNIGSRTYLM--------   132
GH7-5               -----------KYCPDNKSCA-----------ANCAIDGADYSGTYGITT------SG-NALKLN-FVTKGSY----------------------------------------------------------STNIGSRNYLM--------   129
GH7-6               -----------TACPDNAACT-----------KNCAIEGSDYRGTYGIST------SG-NSLSLK-FITKGQY----------------------------------------------------------STNIGSRTYLM--------   134
GH7-2               -----------TACPTKEACA-----------KNCIIEGISDYSTVGVFT------KG-ADLRMDMYNPAGNF-------------------------------------------------------------VSPRVYLL--------   128
GH45-1              ----------------------------------------ATGLATV-AA------QSTGKTTRYW-------------------------------------------DC---------------------------------------    33
GH45-3              ----------------------------------------LSHAANL-NY------SGEAVTTRYW-------------------------------------------DC---------------------------------------    38
GH45-2              -----------------------------------------SLLLHH-VS------GERGVTTRYW-------------------------------------------DC---------------------------------------    32
AA9-7               -----------DVTLSDLQCGGY--------TAGGFKGSSPAALHAE-AA------AG-SEVKLYWTLWP-------------------------------------------------------------ESHVGPSITYM--ARCP--   116
AA9-9               -----------SVTDSKVMCNG----------------GTSADLVAK-VK------AG-GKIRAIWKQW--------------------------------------------------------------THEQGPVMVWM--YKCS--   108
AA9-15              -----------DLSDKNLRCN-----------TNGN--SGSGTTTVA-VA------AG-STVSFTADQA--------------------------------------------------------------VYHQGPVSFYM--SKAS--   102
AA9-10              -----------DVTSNSIVCN-----------GPPNDVPAKTNTVIT-VQ------AG-SKATLTWRHTL--------------------------------------TSG--------------PNDVIDASHKGPVMAYM--KKVS--   108
AA9-20              ---------IKDLTTVNIRCN-----------NKG---LLGTGVTGT-IA------AG-TKLITHWKQW--------------------------------------------------------------THRPATFMVYM--AKCP--   111
AA9-6               ---------PNRASSADVNCH-----------KS----ATPGKLYAN-AN------PG-DTIEFEWNTWP-------------------------------------------------------------DSHRGPIIHYI--APCN--   118
AA9-24              --------EGRFINSEHLRCN-----------KGSMN-HRTQPKTYK-VK------AGQDVIGFQASINTE------------------------------------------------------------LFHPGPVQIYL--SKAP--   110
AA9-19              ---------PSHFNTSNITCH-----------DN----AIPGALHVN-TT------AG-DTLQLKWNEWP-------------------------------------------------------------VSHVGAVMTYI--AKCN--   118
AA9-2               -----------SVTNNDIRCN-----------AN----QGPAASKCS-VP------AG-TTVTIEMHQQNG------------------------------------DRSC--------------ANEAIGGAHYGPVLVYL--SKVS--   106
AA9-23              -----------DLSSIDMRCN-----------VMG---DVQAHDTIK-VA------PG-DNLTFDWHHE--------------------------------------LRNN--------------TDEVIAYSHHGPSLIYI--SPDP--   120
AA9-4               -----------DYTSKALQCN-----------VS----PAAAKGKCA-FA------AG-DTVTIEMHQQFT------------------------------------SRDC--------------KTEAIGGAHWGPVLAYL--SKVE--   107
AA9-1               -----------DVTSNDIRCN-----------QL----KPGTATM-T-VA------AG-SSVKVSVNPN--------------------------------------------------------------AYHPGPFQSYL--AKVPA-    95
AA9-5               -----------SLDSTDMACN-----------VQG---TKGVSRTQP-VQ------DG-SVLTFEIREWPD--------------------------------------DP--------------SKERLDPGHKGPCAVYL--KKVE--   114
AA9-11              -----------DVTSTAMICNG----------------GKATADQVE-VA------AG-STIGMQWHHNEG-----------------------------------PNPND--------------KEEPIAPSHKGPVMVYM--AEAS--   110
AA9-12              -------RDNSHIDGPDIVCN-----------QGAFS-SAGKTDVLT-VK------AG-DEIRLKLAVGAK------------------------------------------------------------FQHPGPEFVYM--AKAP--   118
AA9-3               ---------PDAFGTSAIVCH-----------KQ----GKSNGAYVT-VK------PG-SKVTFKWDTWP-------------------------------------------------------------VSHVGPVQEYI--APCN--   111
AA9-18              -----------DLTSNDMACN-----------VGGSK-VPSGVKTIE-AS------EG-DKIKVQWDN---------------------------------------------------------------SGHPGPITHMLF-GPVD--   100
AA9-13              -----------NVNDGAMACN-----------TG----VSSSSKVID-VR------GG-DRVGVQWGHVVGGA---------------------------------QGAND--------------PDHPIAKSHKGPSIFYM--AKVS--   112
AA9-21              -----------DVTSKAMECN-----------VA----NIKASKSIS-IN------PG-DEVAVQWFHN--------------------------------------GPGA--------------GDQIIDGSHKGPINVYM--SKA---   109
AA9-16              --------TNDIVNSNDLRCN-----------RGA---VAGNTGTYT-VK------AG-DKLGFKIFNNER------------------------------------------------------------IEHPGPGFVYI--SKAP--   106
AA9-22              ---------GDKIAHPDIIAH-----------KD----ASPSPYTAP-VP------AG-SDVTFHWHHEGS-------------------------------------CGG--------------GEEGWDCSHHGWTATYL--APCN--   129
AA9-17              ---------NITPDDTDFRCN-----------KGAFA-SASRTGVAE-VK------PG-TKLAMKLGVAAT------------------------------------------------------------MRHPGPIHVHM--SKAP--   113
AA9-8               ---------PSKFGTGDIICH-----------KAGSP-KGSSDTMGK-IA------AG-GKIDFHWNTWP-------------------------------------------------------------ASHVGPVLTYA--ASYT--   125
AA9-14              -----------SLSSSDMFCG-----------RG----PAASSGVCE-VA------AG-TSLTVEMHAQPG------------------------------------ARSC--------------SQPAIGGNHYGPVLIYM--AKVS--   107
GH3-Alteromonas     SEPKNPVINVRSFGEKPERVA-----------------ELGESFVAALQA------EGVMSAVKHFPGHGDTH---------------------------------------VDSHSGLPRVNH-SETKAREGDLLPFQHIIS-SATPPA   251
GH6-Alteromonas     PDAGESVGLDNPFVGAKWYVDPIW--------------SAKAAGETG-GS------KISGESTFVWMDRIGAIAGPEDGDGMGLRDHLDEALAQNANLFQFVVYDLPNRDCAALASNGELRIADGGFARYQDEYIAGITAILGDQKYAAL   438
GH7-Bacterium       -----------SICTSNEVCA-----------EQCALDGAQYSSTYGITT------SG-DSLRLN-FVTQ-SQ----------------------------------------------------------QKNIGSRLYLM--------    64
GH45-Alteromonas    --------SSSSSSSSSSSSS-----------------SSSSSSSSS-IA------GCDGYATRYW-------------------------------------------DC---------------------------------------   286
                    .......610.......620.......630.......640.......650.......660.......670.......680.......690.......700.......710.......720.......730.......740.......750
```

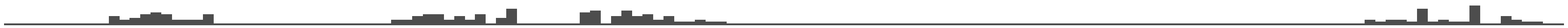

```
GH3-9          MT-----------SYNKVNGT-----H-------AD-SNEFLLKQVLRGEWGW---------------NGLVMSDWGGTN-----------------------STADSLNAGL-------DLEM-------PGPT---------RWRSVE   253
GH3-6          MC-----------SYQRINNS-----Y-------GC-QNSALLNGILKTQLGF---------------EGFVVSDWDAQH-----------------------AGVASANAGL-------DVVM-------PV-----------AKFWGK   309
GH3-5          MC-----------SYNKLNST-----W-------AC-ESEGVMQKLLKDELGH---------------RGYIMSDWNAQH-----------------------TTTGSANGGL-------DMTM-------PGS----DFSGG-NVLWGP   292
GH3-10         MC-----------SYNRINGS-----Y-------GC-QNSKALNGLLKNELGF---------------QGYVMSDWGATH-----------------------AGIASFEAGL-------DMDM-------PGGQ---------------   322
GH3-13         MC-----------SYNAVNGI-----P-------TC-ADTYVLQTILRDHWNWTDS------------NNYITSDCEAVAD-ISENHKYVE--------TLAQGTALAFAKGM-------DLSC--------------------EYTGSS   322
GH3-11         MC-----------SYNAVNGA-----P-------PC-ADPYMLQTVLREHWGWSSD------------EHWVTSDCDSIQN-VYLPHQWSS--------TREGAAADSLNAGT-------DLDC--------------------GTYLQS   392
GH3-14         MV------GGCAMSSAGLNAM-----H-------AC-LSEQVVDDLLRKDLKF---------------DGVVVSECLEMDA-LS------------HNIGVGGGTVMAVNAGC-------DVVLL----CRALSL---------QLEGLK   315
GH3-1          MC-----------SYNQVNSS-----Y-------AC-QNSKLLNGILKDELGF---------------QGFVQSDWLAQR-----------------------SGVASALAGL-------DMTM-------PGDG--LRWAKG-NSLWGS   411
GH3-12         MG-----------AYHSYDGI-----P-------SI-ADGHLQETILRDEWGY---------------KYWLTSDAGATDR-LCCAFKLCQCKTKDKPIDSEAVTLMALPNGN-------DVEM------------------GGGSYNYA   357
GH3-15         MP-----------YYSRPMGT----KYEEV----AAGMNKGIVTDLLRGELGF---------------DGIVVSDWGLVTDGIILGQDMPARAWGAENLTELERTEKILNAGT-------DQLG--------------------GEDRTD   391
GH3-3          MI-----------AYNAVNGS-------------ACAQNSYMTNGLLKDELGF---------------QGFVMSDWLSQI-----------------------SGVSSTLAGL-------DMSM-------PGDRNDIPLVLG-NSYWMY   315
GH3-8          MS-----------SYNMVNGV-----H-------AD-EYIHSIKEVLRGEWKW---------------NGAIISDWTGTY-----------------------ATAPSIKAGV-------DIEM-------PGPS---------KWRKVE   317
GH3-4          MC-----------SYQRINGS-----Y-------GC-QNSASQNGLLKGELGF---------------QGYVMSDWGATH-----------------------SGVASIEAGL-------DMNM-------PGGLGAYGLNFGVPSYFGG   342
GH3-2          MC-----------SYNKANNS-----Q-------VC-QNSYLQNYILKGELGF---------------QGFILSDWDAQH-----------------------SGVASAYAGL-------DMTM-------PGDT---GFNSG-LSFWGT   324
GH3-7          MC-----------SYNRINGS-----H-------GC-QNSYTLNYLLKTELAF---------------QGYVLSDYGALH-----------------------TGIAAANAGM-------DVVT-------PF-----------EEIWGK   357
GH6-1          KINLAIEPDSLANMVTNMGVQ-------------KC-SRAA---PYYKELTAYALKTL-NFNNVDMYMDG-GHAGWLGWD--------------------ANIGPTAKLFAEV-Y-----KAAG------SPRG----------------   238
GH6-2          AFALIIEPDSLPNLVTNSNLA-------------AC-QASA---SGYREGVAYALKQL-NLPNVNMYIDA-GHGGWLGWD--------------------ANIKPGAKELASV-Y-----KNAG------SPKA----------------   242
GH6-3          DFAIVLEPDSLGNAVTNQGVP-------------FC-ANAT---PIYEQGIAYAIAKL-QFPNVSLYMDA-AHGGWLGWA--------------------DNLKPTAQIFARV-V-----EAAKK----INPAAK---------------   244
GH7-4          -------------------GE-------------DK--ENY-------------------------------------------------------------------------------EM----------------------------   120
GH7-3          -------------------AE-------------DK--QNY-------------------------------------------------------------------------------EM----------------------------   137
GH7-1          --------------------E-------------SD--TKY-------------------------------------------------------------------------------QM----------------------------   140
GH7-5          --------------------K-------------DE--KTY-------------------------------------------------------------------------------QM----------------------------   137
GH7-6          --------------------K-------------DT--NNY-------------------------------------------------------------------------------EM----------------------------   142
GH7-2          -------------------SK-------------DE--KNY-------------------------------------------------------------------------------EM----------------------------   137
GH45-1         -----------------------------------C--KGS--------------------------------CAWAGKA--------------------SVTQPLRTCDKND---NPISDL--------------------------MA    65
GH45-3         -----------------------------------C--KPS--------------------------------CGWNGKA--------------------DFSSPVESCTADN---KPT-NP--------------------------AA    69
GH45-2         -----------------------------------C--KPS--------------------------------CAWPGKA--------------------KVSEPVRTCNKQDLWPTPL-DA--------------------------NA    66
AA9-7          ----------------------------------DTGCDAYM--------------------------PE-SNAVWFKVQ----------------------------------------EE----------------------------   137
AA9-9          --------------------G-------------DF--KSC---------------------------NG-SGKKWFKID----------------------------------------QQ----------------------------   127
AA9-15         ----------------------------------SA--ASS---------------------------DG-SG-DWFKIK----------------------------------------EI----------------------------   119
AA9-10         ----------------------------------DA--KTD---------------------------SG-VGGGWFKIA----------------------------------------QD----------------------------   126
AA9-20         --------------------G-------------SC--DSW---------------------------DG-SGKVWFKIF----------------------------------------EQ----------------------------   130
AA9-6          --------------------G-------------EC--SSM---------------------------SP-GDLRWSKFA----------------------------------------QE----------------------------   137
AA9-24         --------------------G-------------DV--RDY---------------------------DG-SG-DWFKVY----------------------------------------QL----------------------------   128
AA9-19         --------------------T-------------TC--SKA---------------------------NK-NTLSWVKID----------------------------------------EL----------------------------   137
AA9-2          ----------------------------------DS--STA---------------------------DG-ST-PFFKIF----------------------------------------QD----------------------------   123
AA9-23         --------------------P--------------------------------------------------TNTSFVKLW----------------------------------------HA----------------------------   132
AA9-4          ----------------------------------DA--ATA---------------------------DG-SS-EFFKIY----------------------------------------EN----------------------------   124
AA9-1          -------------------GA-------------DI--NTW---------------------------DP-TGAVWFRIY----------------------------------------AE----------------------------   115
AA9-5          ----------------------------------DA--TTD---------------------------TA-AGDGWFKIF----------------------------------------DQ----------------------------   132
AA9-11         --------------------T-----------------------------------------------NG-IGNVWTKVW----------------------------------------ED----------------------------   124
AA9-12         --------------------S-------------SV--KTF---------------------------DG-KDGAWFKIH----------------------------------------EQ----------------------------   137
AA9-3          --------------------G-------------DC--GSV---------------------------NP-SSLQWTKIS----------------------------------------SK----------------------------   130
AA9-18         ----------------------------------DA--SQA---------------------------TG-IGAGWFKID----------------------------------------EM----------------------------   118
AA9-13         ----------------------------------NA--ASA---------------------------SP-SGLQWFKVA----------------------------------------ED----------------------------   130
AA9-21         ---------------------------------------------------------------------G-SSMSWTKIA----------------------------------------ED----------------------------   121
AA9-16         --------------------G-------------KV--KDY---------------------------DG-SG-AWTKVM----------------------------------------ES----------------------------   124
AA9-22         --------------------G-------------DC--AKV---------------------------DK-TTLQFFKIH----------------------------------------ES----------------------------   148
AA9-17         --------------------G-------------SV--KEY---------------------------EG-DG-DWFLIK----------------------------------------QA----------------------------   131
AA9-8          --------------------G-------------DI--QAV---------------------------KK-EDLKWFKIE----------------------------------------GV----------------------------   144
AA9-14         ----------------------------------DA--KTA------------------------------SSGSFFKVA----------------------------------------ED----------------------------   123
GH3-Alteromonas FVMSAH------IQYPALDSTTLPNKHGQSQIVPAT-LSRKILHDILRNQMGY---------------KGLVITDALDMAG-IA------------SFFTKEDAVVRAFQAGA-------DIALMPYTIRTPSDI--QAFS-----DFFD   352
GH6-Alteromonas RIVAVIEVDSLPNLVTNLDEA-------------DC-QEAN-GEGGYVDGIQHALNELGKIPNVYSYVDI-AHSGWLGWS--------------------DNFSEAVALIGDAIL-----ETDK------GADS----------------   525
GH7-Bacterium  --------------------D-------------DE--DTY-------------------------------------------------------------------------------TM----------------------------    72
GH45-Alteromonas -----------------------------------C--KAH--------------------------------CSWSGNVP-------------------DGAEPLGACGVND---QPIGDI--------------------------NA   319
               .......760.......770.......780.......790.......800.......810.......820.......830.......840.......850.......860.......870.......880.......890.......900
```

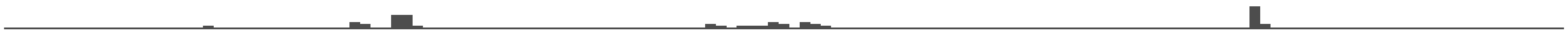

```
GH3-9             AVTEAV-KKGEVTEATITERTRNVLNLIEKVGAFE-----NPEIPPERSVV--------------------------D---PKHCRLIRDVAGQGITLLKNN-GVLPL-----------------RKQQVKGKKIGLFGLA---------   341
GH3-6             NLTDAV-NNGSVTAERFNDMNTRLLAAYYYLNQDEAGF-----DPQVYPYN----------------VKHEIVDVTED-----HGSLIREIGAAGTVLVKNVNNTLPL-----------------I----NPRFLNIYGYDA----EVKA   407
GH3-5             QLKTAI-SNGQVQQSRLDDMVKRVLAAWYLMGQDK-GY--PATSFNSWTIG--------------------SKEISGN-----HGTNVRATARDGTILLKNTNGALPL-----------------K----KPKSIAVIGTDS----IVAP   388
GH3-10            --SLVA-NNSTLHVERLDDMVLRIMTPYYLLGQDR-GY--PAIDTYTTQLK-FLP--EPVYTH--NYTYSPGSNYRND----EATQLIRKLGASGTVLLKNTDNTLPL-----------------Q----QPKQIDVFGNDA----ADLT   432
GH3-13            DIPGAW-AQGLLNISVIDKALTRQYEGLVHAGYFD-GAKATYANLSYKDIN-----------------------------TPEARQLSLQVTSEGLVMLKND-HTLPLPLT-------------------KGSKVAMIGFWAN-------   414
GH3-11            HLPGAV-KQGLTNETTLDNALIRQYSSLIKLGYFDIPENQPYRQLGFDAVA-----------------------------TSASQALALKAAEEGIVLLKND-GVLPINF--------------------GSKNVGIYGDWAN-------   484
GH3-14            GLKTGI-ESEMVSKERIFNSLRRVLTMKKKCTSWE-------KALNPPGINY------------------L------EKLQPLHTALSTKAYNSSITVVRDKNRLLPLTNILDSEEELLLLTPLVKPLAASAASRAI---------SDAA   424
GH3-1             ELTKSV-LNGSVPIDRVDDMVTRVVASWYQVGQDKWEN--DGPNFSSWTNDRIGKLHEGSPSNHEMGVVNQYVNAQGEG-KEAHGHLVRRVAAEGTVLVKNDGNMLPLDRKGWKTDGK------AK----AKFRVGVFGEDA----RLPH   543
GH3-12            NIPRLV-EEGKLDIEIVDRAVSRQLRAKFRQGLFE----RPYQGLPLDEID---------------------SAIHTD----EYVALARKLEADSIVLLENKNNVLPL-----------------S----KSANVAVIGPMA----NITN   452
GH3-15            LILELV-EKGIISESRIDASVRRLLREKFLLGLFD----NPFLDADAAVAV-----------------------VGKD----EWRAIGFEAQKRSFTLLTNKDSFLPL-----------------SASDCGKSKFYIEGFN---------   483
GH3-3             EQTRSV-LNGSVPVDRVNDAVTRILATYFQMGQDQ-NY--PRPNFDTNTQNAEGPLYPGALISP-SGVVNEFVNVQGN-----HAEVAREVARDAITLLKNQDNILPL-----------------A----ASAPLKIFGTDA----EKNP   430
GH3-8             QIKECL-DNGELTPKDIEESAARVLYLVDRVKGLN----NMTPEEPERSID--------------------------N---IETRNLILQAGIEGLTLLKNESNVLPI-----------------R----GAKKIAMIGPNA--------   404
GH3-4             NITLAV-NNGTVNESRIDDMVLRIMTPYFQLGQDN-DF--PSVDPSSADLNTFSP--RKSWTREFNLTGEKSRDVRGN-----HAELIRRHGAAGTVLLKNVDRTLPL-----------------K----APRNVAVFGNGA----SELA   456
GH3-2             NLTVSI-LNGTIPQWRLDDAAIRIMAAYYFVGLDE----SIPVNFDSWQTSTYGF--EHFFGKKSFGLVNKHVDVREE-----HFRSIRRSAAKSTVLLKNS-GVLPL-----------------SG---KEKWTAVFGEDA----GENP   437
GH3-7             NLTMAI-SNHTLEASRLDDMVKRIVAPWIKLAKFEPGSGIPANVSKKHQVV---------------------SAINPS-----SRRNIFQGAAEGIVLVKNHNHTLPL-----------------K----KPKILSLYGYDAHISLKNMP   459
GH6-1             -------VRGIVT---------------------------NVSNYNALRIS---------------------------------------------------------------------------------------------------   255
GH6-2             -------VRGIAT---------------------------NVAGWNSFDQEP------------------G------E------------------------------------------------------------------------   262
GH6-3             -------IRGYST---------------------------NVSNYNPFNAK---------------------------------------------------------------------------------------------------   261
GH7-4             -----------------------------------------------------------------------------L-------QL-----------N-GQE---------------------------FSFDVDM-------------   134
GH7-3             -----------------------------------------------------------------------------L-------QL-----------T-GNE---------------------------LSFDVDM-------------   151
GH7-1             -----------------------------------------------------------------------------F-------NL-----------I-NKE---------------------------FTFDVDV-------------   154
GH7-5             -----------------------------------------------------------------------------F-------KF-----------TGNQE---------------------------FTFDVDV-------------   152
GH7-6             -----------------------------------------------------------------------------F-------QL-----------I-GNE---------------------------FTFDVDL-------------   156
GH7-2             -----------------------------------------------------------------------------L-------QL-----------T-GNE---------------------------LTFDVDM-------------   151
GH45-1            --KNGCESGGTAF---------------------------MCTNQTPWAVN--------------------------N-------SLAY-----------------------------------------GFAAVKL-------------    99
GH45-3            --GTGC-NGGDAF---------------------------QCADQQPWAIN--------------------------D-------TMSY                                         GFAGVYIMPALT--------   107
GH45-2            --ASAC-GNGEAY---------------------------TCSNNGPWAVN--------------------------D-------NLAY                                         GFAAANL-------------    99
AA9-7             ---------GRQG------------------------------TSNKWAS---------------------------D-------AI-----------RVAG----------------------------GYLSYTI-------------   162
AA9-9             ---------GMTA---------------------------PPLSGRNWGT-----------------------------------AF-----------VLKN----------------------------LYWESTV-------------   154
AA9-15            ---------GPNF----------------------------SSGQAVWDM---------------------------S----------------------------------------------------TTYSVTI-------------   140
AA9-10            ---------AFDG--------------------------------SKWGV---------------------------D-------RL-----------IANQ----------------------------GKQTITI-------------   149
AA9-20            ---------GLIS---------------------------GTENAGVWAG---------------------------D-------AI------------FET----------------------------LNAAITI-------------   157
AA9-6             ---------AIVS-------------------------------PNVWVT---------------------------D-------NL-----------IKNG----------------------------FKASVKL-------------   161
AA9-24            ---------GHQP---------------------------GSTADSAWLS-------------------------------------------------WHK----------------------------KQFTFKL-------------   152
AA9-19            ---------AWLNSTG----------------------WDTLELGGTWAT---------------------------D-------VL-----------IANN----------------------------FTWEVKI-------------   170
AA9-2             ---------TWAR-----------------------NPSGGGGSDDYWGT---------------------------K-------DL-----------NKNC----------------------------GKMDVKI-------------   155
AA9-23            ---------GKIE-------------------------SNPFPQAGKWST---------------------------TA------DI-----------RANY----------------------------GRMNVRI-------------   163
AA9-4             ---------TWAK-----------------------NPAASQGDNDFWGT---------------------------K-------DL-----------NYNC----------------------------GKLDFTI-------------   156
AA9-1             --------------------------------------------QPKFGN---------------------------Q-------LT-----------WLAA----------------------------GTYDIKI-------------   135
AA9-5             ---------GYDA------------------------------DSKQWCT---------------------------D-------KI-----------IANN----------------------------GLLSVKL-------------   157
AA9-11            ---------GYKD--------------------------------GKFGV---------------------------D-------RF-----------YDNK----------------------------GLIEIKL-------------   147
AA9-12            ---------GVCD--------------------------GGDFTSTAWCD-------------------------------------------------YNR----------------------------DYISARI-------------   162
AA9-3             ---------AWKS----------------------------GSNPGKWAT---------------------------D-------DL-----------IANN----------------------------FSWDITF-------------   157
AA9-18            ---------DFVG--------------------------------GKWAS---------------------------E-------IM-----------QAAN----------------------------MSHEFTL-------------   141
AA9-13            ---------GLQG--------------------------------STWGV---------------------------D-------RM-----------ISNN----------------------------GWTEFTV-------------   153
AA9-21            ---------GWDG--------------------------------KSWAV---------------------------T-------KLRDGA------YNGKK----------------------------GQHTFKM-------------   149
AA9-16            ---------GLAN-------------------------PSAPGVDKSWAN-------------------------------------------------WDK----------------------------DRLEWTI-------------   150
AA9-22            ---------ALID---------------YRKGRYS--SGSPQEQTGYWGT---------------------------D-------AI----------FYDNR----------------------------NTQTVKI-------------   187
AA9-17            ---------GLCT--------------------------KGDIKGDAWCS-------------------------------------------------WDK----------------------------NNVEFDI-------------   156
AA9-8             ---------GYEN--------------------------------GEWAA---------------------------V-------KM-----------IANN----------------------------NTWTTTV-------------   167
AA9-14            ---------GYTG------------------------------TTASWGT---------------------------E-------IL-----------NANC----------------------------GKRSFTV-------------   148
GH3-Alteromonas   KLQTQL-KKGTIKRQEHLMSLQRVSNAKSSFSL---------RNFHEKGLS------------------------------WWREEVAENPWNTKNKAVEKALADAAITLLYGAEALP------LR----TTTWLAMMPDTA--------   444
GH6-Alteromonas   -------IAGFAS---------------------------NSANYTPVTEPY------------------M-----------------------------------------------------------PDPDLQVGGNPV--------   556
GH7-Bacterium     -----------------------------------------------------------------------------F-------HL-----------L-NRE---------------------------FTFDVDV-------------    86
GH45-Alteromonas  --GSAC-DGGNAH---------------------------MCQGLSPWAVS--------------------------N-------TLSY                                         GYAATSS-------------   352
                  .......910.......920.......930.......940.......950.......960.......970.......980.......990......1000......1010......1020......1030......1040......1050
```

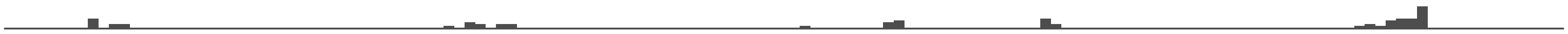

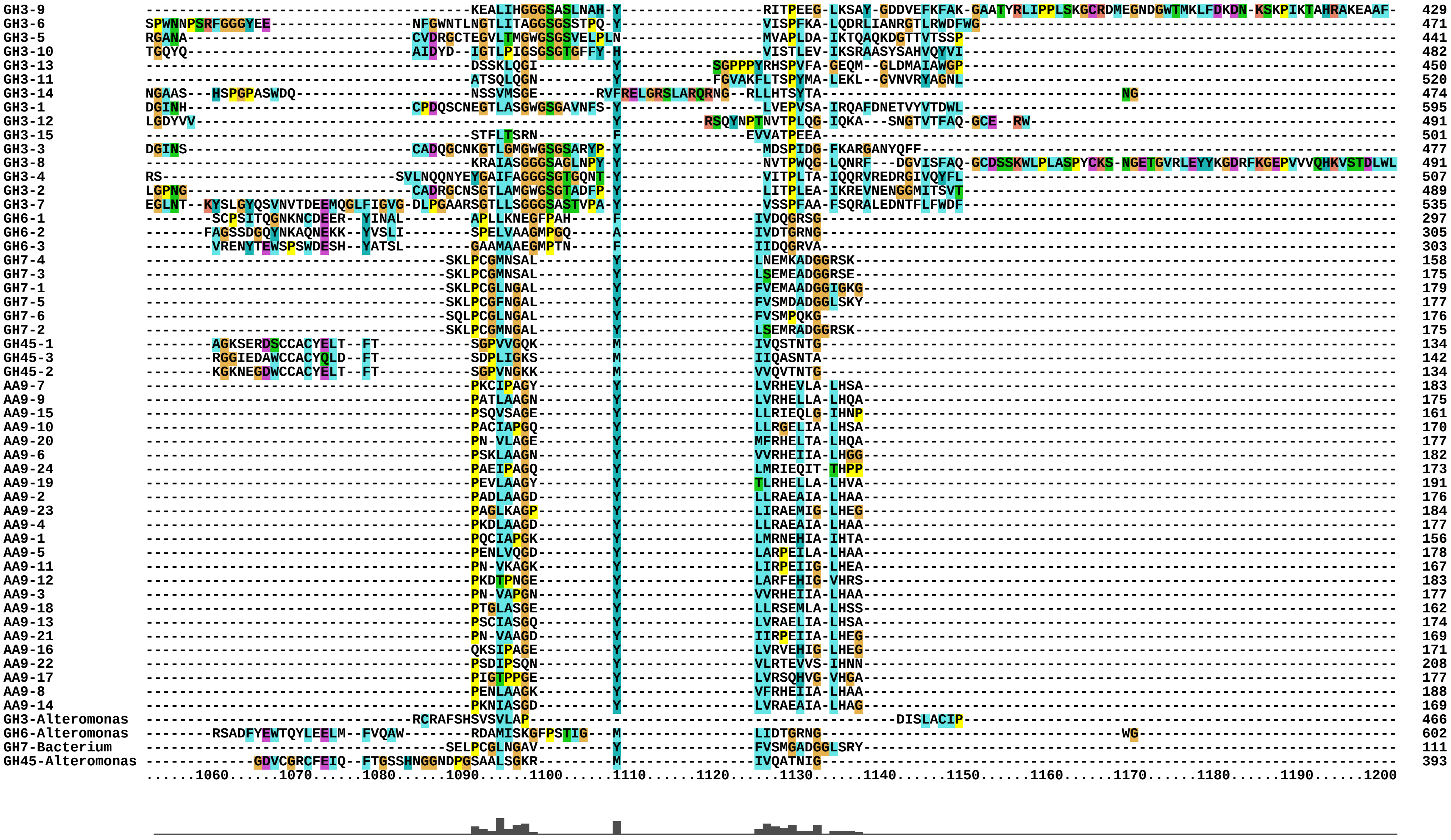
GH3-9 429
GH3-6 471
GH3-5 441
GH3-10 482
GH3-13 450
GH3-11 520
GH3-14 474
GH3-1 595
GH3-12 491
GH3-15 501
GH3-3 477
GH3-8 491
GH3-4 507
GH3-2 489
GH3-7 535
GH6-1 297
GH6-2 305
GH6-3 303
GH7-4 158
GH7-3 175
GH7-1 179
GH7-5 177
GH7-6 176
GH7-2 175
GH45-1 134
GH45-3 142
GH45-2 134
AA9-7 183
AA9-9 175
AA9-15 161
AA9-10 170
AA9-20 177
AA9-6 182
AA9-24 173
AA9-19 191
AA9-2 176
AA9-23 184
AA9-4 177
AA9-1 156
AA9-5 178
AA9-11 167
AA9-12 183
AA9-3 177
AA9-18 162
AA9-13 174
AA9-21 169
AA9-16 171
AA9-22 208
AA9-17 177
AA9-8 188
AA9-14 169
GH3-Alteromonas 466
GH6-Alteromonas 602
GH7-Bacterium 111
GH45-Alteromonas 393
1060 1070 1080 1090 1100 1110 1120 1130 1140 1150 1160 1170 1180 1190 1200

```
GH3-9              --SPLLEHQAKHSEVVFGATFYPTESGSHYLGCSGIGPTTVYINEELVFEQKHNSPDAMGFLFGGNPEKEFRVPFTKGQCYKIEIIS----RPPS-----GSNDDAGILDGLPGFRLGFMYEEEHDKDLAA-EAQDLAKDC-------DY   560
GH3-6              ------------------------------------------------------------------------------------------------------------------------------------VNPTVYANA-------EA   482
GH3-5              ------------------------------------------------------------------------------------------------------------------------------TDNAQ-QGASAAQNA-------EI   457
GH3-10             ------------------------------------------------------------------------------------------------------------------------------GNTFI-ALGGLFELA-PKPI--NV   502
GH3-13             -----------------------------------------------------------------------------------------------------------------------MIQNSSVPDNWTTNALDAAEKS-------DY   474
GH3-11             -------------------------------------P-------------------------------------------------------------------------------GGQGDPTTGSWP-RLSGVITTS-------DV   544
GH3-14             ---------------------------------------V---------------------------------------------------RPVH-------------------------------------------ENLISR----AS   487
GH3-1              ------------------------------------------------------------------------------------------------------------------------------ENKLP-NQKEILEDQ-------DL   611
GH3-12             ---------------------------------------------------------------------------------------------------------------------------SNDQSGFP-DAVAAAKAA-------DV   510
GH3-15             ----------------------------------------------------------------------------------------------------------------------------------------------------DY   503
GH3-3              -----------------------------------------------------------------------------------------------------------------------------NTDNFP-GNSNPSPN--------DT   493
GH3-8              WDSAPMELLPDFSFKV-KTTLTPKSSGNHSFSFASVGPGRMFIDGEL-FVDNWDWTEEGEAMFSASEDVLKSIYLEEGKPVEILIESTSEVRPASKVSVIGRRHDYG------GCRIG--YQEEDKIDRLQ-EAADVARDA-------DV   623
GH3-4              ------------------------------------------------------------------------------------------------------------------------------NNTHI-INNNVSTLVIPRDVP-DV   529
GH3-2              ------------------------------------------------------------------------------------------------------------------------------DNYATSQIQNMASRA-------SH   506
GH3-7              -----------------------------------------------------------------------------------------------------------------------------E------SQDPVYANAGS-----DA   549
GH6-1              ------------------------------------------------------------------------------------------------------------------------------------------------------   297
GH6-2              ------------------------------------------------------------------------------------------------------------------------------------------------------   305
GH6-3              ------------------------------------------------------------------------------------------------------------------------------------------------------   303
GH7-4              ------------------------------------------------------------------------------------------------------------------------------------------LNP---------   161
GH7-3              ------------------------------------------------------------------------------------------------------------------------------------------YNT---------   178
GH7-1              ------------------------------------------------------------------------------------------------------------------------------------------NNK---------   182
GH7-5              -------------------------------------P----------------------------------------------------------------------------------------------------GNK---------   181
GH7-6              ------------------------------------------------------------------------------------------------------------------------------------------QGT---------   179
GH7-2              ------------------------------------------------------------------------------------------------------------------------------------------LNP---------   178
GH45-1             ------------------------------------------------------------------------------------------------------------------------------------------GDL---------   137
GH45-3             ------------------------------------------------------------------------------------------------------------------------------------------YDI---------   145
GH45-2             ------------------------------------------------------------------------------------------------------------------------------------------SDL---------   137
AA9-7              ------------------------------------------------------------------------------------------------------------------------------------------YKY---------   186
AA9-9              ------------------------------------------------------------------------------------------------------------------------------------------------------   175
AA9-15             ------------------------------------------------------------------------------------------------------------------------------------------------------   161
AA9-10             ------------------------------------------------------------------------------------------------------------------------------------------SQL---------   173
AA9-20             ------------------------------------------------------------------------------------------------------------------------------------------------------   177
AA9-6              ------------------------------------------------------------------------------------------------------------------------------------------SSP---------   185
AA9-24             ------------------------------------------------------------------------------------------------------------------------------------------------------   173
AA9-19             ------------------------------------------------------------------------------------------------------------------------------------------EQL---------   194
AA9-2              ------------------------------------------------------------------------------------------------------------------------------------------GST---------   179
AA9-23             ------------------------------------------------------------------------------------------------------------------------------------DVSFAQNPR---------   193
AA9-4              ------------------------------------------------------------------------------------------------------------------------------------------GPA---------   180
AA9-1              ------------------------------------------------------------------------------------------------------------------------------------------GQR---------   159
AA9-5              ------------------------------------------------------------------------------------------------------------------------------------------KD----------   180
AA9-11             ------------------------------------------------------------------------------------------------------------------------------------------SAV---------   170
AA9-12             ------------------------------------------------------------------------------------------------------------------------------------------HV----------   185
AA9-3              ------------------------------------------------------------------------------------------------------------------------------------------GQT---------   180
AA9-18             ------------------------------------------------------------------------------------------------------------------------------------------QTK---------   165
AA9-13             ------------------------------------------------------------------------------------------------------------------------------------------SSV---------   177
AA9-21             ------------------------------------------------------------------------------------------------------------------------------------------NRP---------   172
AA9-16             ------------------------------------------------------------------------------------------------------------------------------------------HV----------   173
AA9-22             ------------------------------------------------------------------------------------------------------------------------------------------GDV---------   211
AA9-17             ------------------------------------------------------------------------------------------------------------------------------------------HD----------   179
AA9-8              ------------------------------------------------------------------------------------------------------------------------------------------GQE---------   191
AA9-14             ------------------------------------------------------------------------------------------------------------------------------------------------------   169
GH3-Alteromonas    ------------------------------------------------------------------------------------------------------------------------------------LTAKPSKQVLTRLLPHAE   484
GH6-Alteromonas    ------------------------------------GPN----------------------------------------------------RPTG-------------------------------------------ESS---------   612
GH7-Bacterium      -------------------------------------P----------------------------------------------------------------------------------------------------TNE---------   115
GH45-Alteromonas   ------------------------------------------------------------------------------------------------------------------------------------------SDV---------   396
                   ......1210......1220......1230......1240......1250......1260......1270......1280......1290......1300......1310......1320......1330......1340......1350
```

```
GH3-9             AIVFTGHTP---------------------VWETEGQ-----------DQASF----HLPREGTQDKLIAAVSSVN----PKTIVV-NSTGVPVALPWLD--NVAALVQAWFPG----------QEAG----------NAIADILSG--A   645
GH3-6             CLVFIN------------------------AYASESF-----------DRT------SLTDDF-SDQLVNNVATNC----SNTIVVIHSAGVRTVDAWYDHPNVTAILYAGVPG----------QESG----------NAIADVLLG--D   564
GH3-5             AVVCIN------------------------SNAGEGYINVEGN---AGDRN------NLDPWHNGNELVKAVAAVN----KKTVVVVHSVGPIIMEQWIENPNVVAVVWAGLPG----------QEPG----------NGVVDIMYG--A   548
GH3-10            CILFLK------------------------SWASEGS-----------DRT------TLIPEWNSKVVVERVTGVC----DNTVVVLHGASPNTM-LWRSNPKVTAILTAHMPG----------ERTG----------NSIVDILWG--D   584
GH3-13            ILYFGGQDW---------------------TVAQEGY-----------DRT------TISFPQVQIDLLTKLAKLG----KPLVVI-TLGDMTDHSPLLSMEGVNSIIWANWPG----------QDGG----------PAILNVVSG--A   559
GH3-11            HIWVGGMDN---------------------GIESEDR-----------DRS------WLTLTGSQLDVIGQLADTG----KPVIVIIMGGGQIDTSPLIKNPKISAVLWAGYPG----------QDGG----------TAIVNILTG--K   630
GH3-14            AIIVLTADAN------RNLYQHGFTKHVSMICNMQYTTG--------GERR-----------------------------EKPLIVVAVSSPY---DFAMDNTIGTYICTYDFT----------ETAL----------NSLVRVLYG--E   569
GH3-1             CIVFAN------------------------ADSGEGYEHYGNI---RGDRN------DLNLQKGGAELIRNVAKDCGEGMGDVVVVIHTVGSVILEDFIDIMNVRAVLIAHLPG----------TESG----------NAIVDVLFG--D   706
GH3-12            AVVVVGTWS---------------------RDQQELWQGLNATTGEHVDVA------SLNLVGAMGPLVQAIIQTG----KPTIVV-YSSGKPVTEPWISD-NAAGLLQMFYPG----------EQGG----------NGLADVLFG--D   605
GH3-15            AFLRLVAPYEPRPGGFEASYH---------SGSLEYNAT---------ERARQ-------------------------------AAIYATVPTVVDIFLDR-----------PGAFPE----VAEQAEALMVNFGASEDAFLDVVFGVDG   589
GH3-3             AVVFVT------------------------ADSGENYITVEGN---PGDRTSA----NLNLWHNGDKLIKDVAAKY----SNVVVVVHTVGVILMNEWHDLPSVKAIVFAHLPG----------QEAG----------NSLMQVLYG--D   586
GH3-8             AVVVIGLDA---------------------EWESEGY-----------DRQTM----DLPKNGSQDRLIEAVLAAN----PRTVIV-NQSGTPVTMPWVH--KAPAILQAWYQG----------QEAG----------NALADVLLG--N   708
GH3-4             CIVMLK------------------------TWAEEGD-----------DRY------HLGSDWNGDKVVESVASFC----NNTVVVTHSSGINTL-PWSDHPNVTAILAAHFPG----------EESG----------NSLVDILYG--D   611
GH3-2             SIVFVN------------------------ADSGEGYITVDNN---MGDRN------NLTVWGNGDVLVKNVSALC----NNTIVVIHSVGPVIVDAWKENANVTAILWAGLPG----------QESG----------NSIADILYG--H   597
GH3-7             CIVFIN------------------------EFAAEGQ-----------DRS------TLADRW-SDKLVINVASKC----PNTIVSIHNAGTRLLDQWIEHPNITAVLFGHLPG----------QDSG----------SALVSIMYG--D   631
GH6-1             --------------------------------------------------------------------------------------------------------------KVPTNQ--------QEWGD--------WC---NV-SG---   314
GH6-2             ---------------------------------------------------------------------------------------------------------------VTGLR--------KEWGD--------WC---NV-IG---   321
GH6-3             ---------------------------------------------------------------------------------------------------------------LPGAR--------KEWGE--------WC---NV-----S   318
GH7-4             -------------------------------TGANMGWGYC-------DAQCSVK-PFLDGEANID-----------------------------------------------------------KEGA--------CCNEMDIWEA---   202
GH7-3             -------------------------------GGAPQGSGYC-------DAQCFVK-PFLNGEANAN-----------------------------------------------------------GEGA--------CCNEMDIWEA---   219
GH7-1             -------------------------------AGAKYGTGYC-------DSQCPHDIKFINGKANVEGWNPSAADPN------------------------------------GGA---------GKIGA--------CCPEMDIWEA---   238
GH7-5             -------------------------------AGAKYGTGYC-------DAQAPRDLKFINGEANVEGWKPSSNDQN------------------------------------AGV---------GGHGS--------SCAEMDIWEA---   237
GH7-6             -------------------------------PGAKYGTGYC-------DAQCARDLKYIDGQANAEGWTASSSDPN------------------------------------AGI---------GKKGA--------CCAEMDIWEA---   235
GH7-2             -------------------------------KGAAYGTGYC-------DAQCFVT-PWMNGEGNVQ-----------------------------------------------------------KQGV--------CCNEMDIWEA---   219
GH45-1            -------------------------------GDNHFDIA--------------------------------------------------------------------------------------------------------M-PG---   148
GH45-3            ------------------------------TTTNRFTLA--------------------------------------------------------------------------------------------------------V-PG---   157
GH45-2            -------------------------------KENQFDLA--------------------------------------------------------------------------------------------------------I-PG---   148
AA9-7             -------------------------------PGAQFYPG---------------------------------------------------------------------------------------------------CHQLKV-TG---   202
AA9-9             -------------------------------RSPQFYAE---------------------------------------------------------------------------------------------------CAQIEV-TG---   191
AA9-15            ------------------------------GSPPQFYIS---------------------------------------------------------------------------------------------------CAQIKV-TG---   178
AA9-10            -------------------------------MGAQFYME---------------------------------------------------------------------------------------------------CAQINV-IG---   189
AA9-20            -------------------------------NNPQFYPE---------------------------------------------------------------------------------------------------CAQFTV-TG---   193
AA9-6             -------------------------------NGAQLYPQ---------------------------------------------------------------------------------------------------CINVKV-GG---   201
AA9-24            ------------------------------YTNREFYIQ---------------------------------------------------------------------------------------------------CAHIEV-AS---   190
AA9-19            -------------------------------DGAQAYPQ---------------------------------------------------------------------------------------------------CINLRV-DG---   210
AA9-2             -------------------------------GGAQFYMT---------------------------------------------------------------------------------------------------CYQITV-TG---   195
AA9-23            -------------------------------RGAQFYPD---------------------------------------------------------------------------------------------------CVQIEV-VG---   209
AA9-4             -------------------------------GGAQHYVT---------------------------------------------------------------------------------------------------CYQLTV-TG---   196
AA9-1             -------------------------------GGAQFYLS---------------------------------------------------------------------------------------------------CAQIEV-TG---   175
AA9-5             -------------------------------GDPQFYTG---------------------------------------------------------------------------------------------------CAQIFL-KS---   196
AA9-11            -------------------------------GKAQFYNG---------------------------------------------------------------------------------------------------CGQITV-TG---   186
AA9-12            -------------------------------NQPEHYVS---------------------------------------------------------------------------------------------------CMQIKV-EG---   201
AA9-3             -------------------------------NGAQAYPQ---------------------------------------------------------------------------------------------------CINFKV-EG---   196
AA9-18            -------------------------------GGAQFYIG---------------------------------------------------------------------------------------------------CMQLKI-KG---   181
AA9-13            -------------------------------GGAQFYIG---------------------------------------------------------------------------------------------------CAQVNV-SA---   193
AA9-21            -------------------------------SGAQFYMG---------------------------------------------------------------------------------------------------CIHVKV-GG---   188
AA9-16            -------------------------------GKAQFYME---------------------------------------------------------------------------------------------------CFQLNI-QS---   189
AA9-22            -------------------------------SNRQFWPQ---------------------------------------------------------------------------------------------------AFNIKV-TG--G   228
AA9-17            -------------------------------GQAEFYYG---------------------------------------------------------------------------------------------------CAQIKV-TG---   195
AA9-8             -------------------------------NGAQNYPQ---------------------------------------------------------------------------------------------------CVNIEV-TG---   207
AA9-14            ------------------------------VGSPQPYVS---------------------------------------------------------------------------------------------------CFQVKV-TG---   186
GH3-Alteromonas   TIIVGDISPQ------HAVY---------ELAGLESPENIQNRM-DEGTRH--------------DFLIQLMQSSKQ--QNKQIVFVPMRMPYVANTFMPYSDIGIATFSYSVTLNQEEESPVSSAIT----------DALVEVLLG--K   590
GH6-Alteromonas   ------------------------------SSDLETYVN-----ESRIDRR-------------------------------------------------------------------------EHRGN--------WC---------NQ   637
GH7-Bacterium     -------------------------------AGAKYGTGYC-------DSQCPRDLKFINGQANVEGWEPSDTNPN------------------------------------AGV---------GGHGS--------CCAEMDIWE----   170
GH45-Alteromonas  -------------------------------AGGQFDIL--------------------------------------------------------------------------------------------------------V-PG---   407
                  ......1360......1370......1380......1390......1400......1410......1420......1430......1440......1450......1460......1470......1480......1490......1500
```

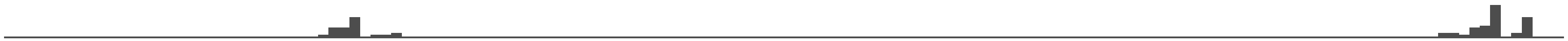

```
GH3-9              VNPSGHLPI---------------------------S---------------WPKRIEDAPAHGNFPGE-----RD--ESGQLTVKYAEGVFVGYRHYDRLGKEKVHFPFGF------------------GLSY----------------   712
GH3-6              VNPSGRLPY---------------------------T---------------VARSESDYGALLNSTF-------DPSIPAFLQSDFTEGLYIDYRDFN-KRNVTPLYEFGF------------------GLSY----------------   630
GH3-5              ASPSGKLPY---------------------------T---------------IAKKESDYGTTIARG-----------------DDKSWDLFIDYRYFD-KQNITPRFEFGF------------------GLSY----------------   604
GH3-10             VNPSGRLPY---------------------------T---------------IANNDTEYRKNIANSIAF--QDTD--DPSDWQADFFEGNLIDYK--------DVAFPFGF------------------GLNY----------------   646
GH3-13             HAPAGRLPI---------------------------TEYPADYV-KLSMLDMNLRPHTESPGRT------------------------------YRWFN--ESVQP---FGF------------------GLHY----------------   612
GH3-11             AAPAGRLPQ---------------------------TQYLYKYVSEVPMTDMAMRPSNKNPGRT------------------------------YKWYT----GKPIFEFGY------------------GLHY----------------   685
GH3-14             LSPSGTLPG---------------------------T---------------ISKSQKLHPSKQHWLVEIFNEERDGSALDTLIAAVVESTAPNQRSEL-SSATSSSFILHHPEVEESHFVVRN---SSTQALY----------------   657
GH3-1              VNPSGRLPY---------------------------T---------------IAKKEDDFGPGSKVKYLP--SPSDG---LAPQQDFTEGLYIDYRYFD-KQNITPRFEFGY------------------GLSY----------------   774
GH3-12             VTPSGKLSV---------------------------SFPYDVGTL-PIYYDYLNSGRSTDPGAI----------------------LPNGTLKFGHQYV-LNTPQPLYEFGY------------------GLSY----------------   670
GH3-15             SGPEGLLPF--------------D----------------------------LPRSDEAAKTQKEDVP--------------------------------FDTLDPAFKFGY------------------GLRY----------------   631
GH3-3              VSPSGHLPY---------------------------T---------------LPNAEDDFGNSVKLVGY---------QLGQPQDTFTEGLYIDYRHFH-KANITPRYAFGH------------------GLSY----------------   650
GH3-8              SSPSGKLPT---------------------------T---------------FPVRIEDNPAYHNWPGE------------NLKTIYGEGIYVGYRHYE-RSKIAPLFPFGH------------------GLTY----------------   769
GH3-4              YNPSGHLPY---------------------------T---------------IAKNGDDWNAPPTTEI-----QTD--GFNDWQSWFDEKLEIDYRHFD-MHNISVHYEFGF------------------GLSY----------------   677
GH3-2              HNPGGKLPF---------------------------T---------------IGSSAEEYGPDI----IY--EPTN--GILSPQANFEEGVFIDYRAFD-KAGIEPTYEFGF------------------GLSY----------------   662
GH3-7              QAPSGRLPY---------------------------T---------------VAKRESEYGDLLSPIR------ADNTSNYYTSANLTEGVYIDYRHFD-AHDIIPRFEFGF------------------GLTY----------------   698
GH6-1              -AGFGTRPT---------------------------TNTGNALI---DAIVWVK-PGGESDGTS---------------------------------------------------------------NTSAARY----------------   353
GH6-2              -AGFGRRPT---------------------------SDTGSSLV---DAFVWAK-PGGESDGTS---------------------------------------------------------------DTSAKRY----------------   360
GH6-3              PSGFGMRPG---------------------------AAPNNTNV---DSIVWIK-PGGESDGAC--------------------------------------------------------------------------------------   351
GH7-4              NGRANQIAPHVCAKEGVIRCTG-DDCGV-------AGVCGKTGC---GDNAYNFRNSKDFYGPG-------------------------------------LKVDTTRPFTVVT----QFPEKYGVLQAIIRKYVQDGVVVENAMSNVT-   299
GH7-3              NSYANQIAPHTCAKPGIFACTG-NDCGS-------AGLCDKAGC---GDNPYKDRNDKEYYGPG-------------------------------------LKVDTNRPFTVVT----QFPAKDGVLQAIVRKYVQDGVVIENAMRNLTA   317
GH7-1              NSISTAYTPHPCKGVGLQECSDAASCGD--GSNRYDGRCDKDGC---DFNSYRM-GVKDFYGPG-------------------------------------ATLDTTKKMTVIT----QFLGSGSKLSEIKRFYVQNGKVYKNSQSAVAG   341
GH7-5              NSVSTAVTPHSCSTIEQTRCDG-DSCGGTYSATRYAGVCDADGC---DFNSYRM-GVKDFYGKG-------------------------------------KTVDTSQKFTVVT----QFVGTGDNL-EIRRFYVQGGKVIPQPSSKIPG   340
GH7-6              NSMATALTPHSCQPEGYSVCVD-DTCGGTYSLDRYAGTCDANGC---DFNPYRV-GVTDFYGRG-------------------------------------KTVDTTKKMTVVT----QFLGSGNKLTELKRFYVQNGKVFANPEPTVPG   339
GH7-2              NSRSTSIAPHTCGKPSIFGCTG-DECGK-------TGLCDKNGC---VDNPYKTRGDKAYYGLN-------------------------------------LKVDTNRPFTVVT----QFPAKNGVLQAIVRKYIQDGVVMDNAVKNIT-   316
GH45-1             -GGVGIF----------------------------NGCTAQFGA---PSTGW----GQQYGGIS-------------------------------------S------------------------------------------------   177
GH45-3             -GNTTSV-D---------------------------GCAKQYGV---DQSVF----GQRMEGVS-------------------------------------S------------------------------------------------   186
GH45-2             -GGVGIFPQ---------------------------GCAKQFNG------AWM---GNTFGGYW-------------------------------------Q------------------------------------------------   176
AA9-7              -GGSTK-PS---------------------------G-------------------LVSFPGAY-------------------------------------KGTDAGIAYNA---------------------Y----------------   230
AA9-9              -GGSAS-PS---------------------------GDY-----------------LATIPGYA-------------------------------------SQNDPGIMVDT---------------------Y----------------   221
AA9-15             -GGSGN-PS---------------------------P-------------------TTKIPGHV-------------------------------------KSTDSGYTANI---------------------Y----------------   206
AA9-10             -GSASK-TP---------------------------A-------------------TVSFPGAY-------------------------------------KQNDAGIVYNL---------------------Y----------------   217
AA9-20             -SGTAS-PP---------------------------ASF-----------------LVSFPGAY-------------------------------------TGTEPGLAFNIDS---------------------EDA------------   227
AA9-6              -SGTVR-PS---------------------------N-------------------GVPGTSLY-------------------------------------TPTTPGILFNI---------------------Y----------------   229
AA9-24             -SYNGS-PS---------------------------P-------------------TFKIPGVY-------------------------------------NREQPFFKYDS---------------------W----------------   218
AA9-19             -ETGAE-AKH------------------------LEG-------------------GVLGTNLY-------------------------------------RPTDAGILVDV---------------------H----------------   241
AA9-2              -GGSSV-PS-----------------------------------------------GVSFPGAY-------------------------------------KASDPGILINI---------------------Y----------------   222
AA9-23             -GGEVELPE-----------------------------------------------GVGFPGAY-------------------------------------SYEDPGVVHNI---------------------Y----------------   237
AA9-4              -SGTLA-PK-----------------------------------------------GVTFPEAY-------------------------------------SKTGPGLGFSI---------------------H----------------   223
AA9-1              -GGSTS-PR---------------------------N-------------------LVAFPGAY-------------------------------------SATDPGILINI--------------------NY----------------   204
AA9-5              -TGNLV-PE---------------------------S-------------------TVSIPGHV-------------------------------------KYNEPSTDFDI---------------------Y----------------   224
AA9-11             -SGSASLPA---------------------------S-------------------GVDMTKAY-------------------------------------KATDPGVKFDI---------------------Y----------------   215
AA9-12             -GGSGT-PG---------------------------P-------------------TVKFPGAY-------------------------------------KASDPYANFSI---------------------Y----------------   229
AA9-3              -SGTGR-LS---------------------------G-------------------GTPATSFY-------------------------------------TANDPGIKFNV---------------------F----------------   224
AA9-18             -TGSKGSCT---------------------------P-------------------TISLPGAY-------------------------------------KADDANIFIPN----Y----------------Y----------------   211
AA9-13             -SGSKT--G---------------------------T-------------------TVSFPGAY-------------------------------------RADDAGIKVSI---------------------YNSQG------------   224
AA9-21             -SASGS-LP---------------------------A-------------------GVSIPGYV-------------------------------------TATNPGVLFNI---------------------Y----------------   216
AA9-16             -SGTGT-LG---------------------------P-------------------TVKIPGLY-------------------------------------SANDAGIKFNK---------------------W----------------   217
AA9-22             KNGAKV-PA-----------------------------------------------GKKGTELY-------------------------------------KANDPLLKWDL---------------------Y----------------   256
AA9-17             -DGAGV-PG---------------------------P-------------------MIKFPGGY-------------------------------------KKTDPSWNFSV---------------------W----------------   223
AA9-8              -SGTEK-PE-----------------------------------------------GVVGTKLY-------------------------------------TSTDPGIKFDV---------------------Y----------------   234
AA9-14             -GGSAT-PS-----------------------------------------------GVSFPGAY-------------------------------------KLSDPLFTKAI---------------------Y----------------   213
GH3-Alteromonas    SQAHSYAP----------------------------------------------------------------------------------------------------------------------------------------------   598
GH6-Alteromonas    PGGVGFFPQ---------------------------AAPA-AGV---DAYVWVK-PQGESDGVS----------------------------------------DPNFEIDP---------------NDPNKQH----------------   684
GH7-Bacterium      ------------------------------------------------------------------------------------------------------------------------------------------------------   170
GH45-Alteromonas   -GGVGQF----------------------------NACSSQWGV---SNDEL----GAQYGGFL-------------------------------------AACKDELGYNA-----------------SLSQY----------------   451
                   ......1510......1520......1530......1540......1550......1560......1570......1580......1590......1600......1610......1620......1630......1640......1650
```

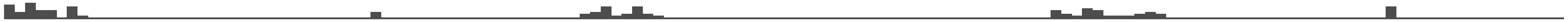

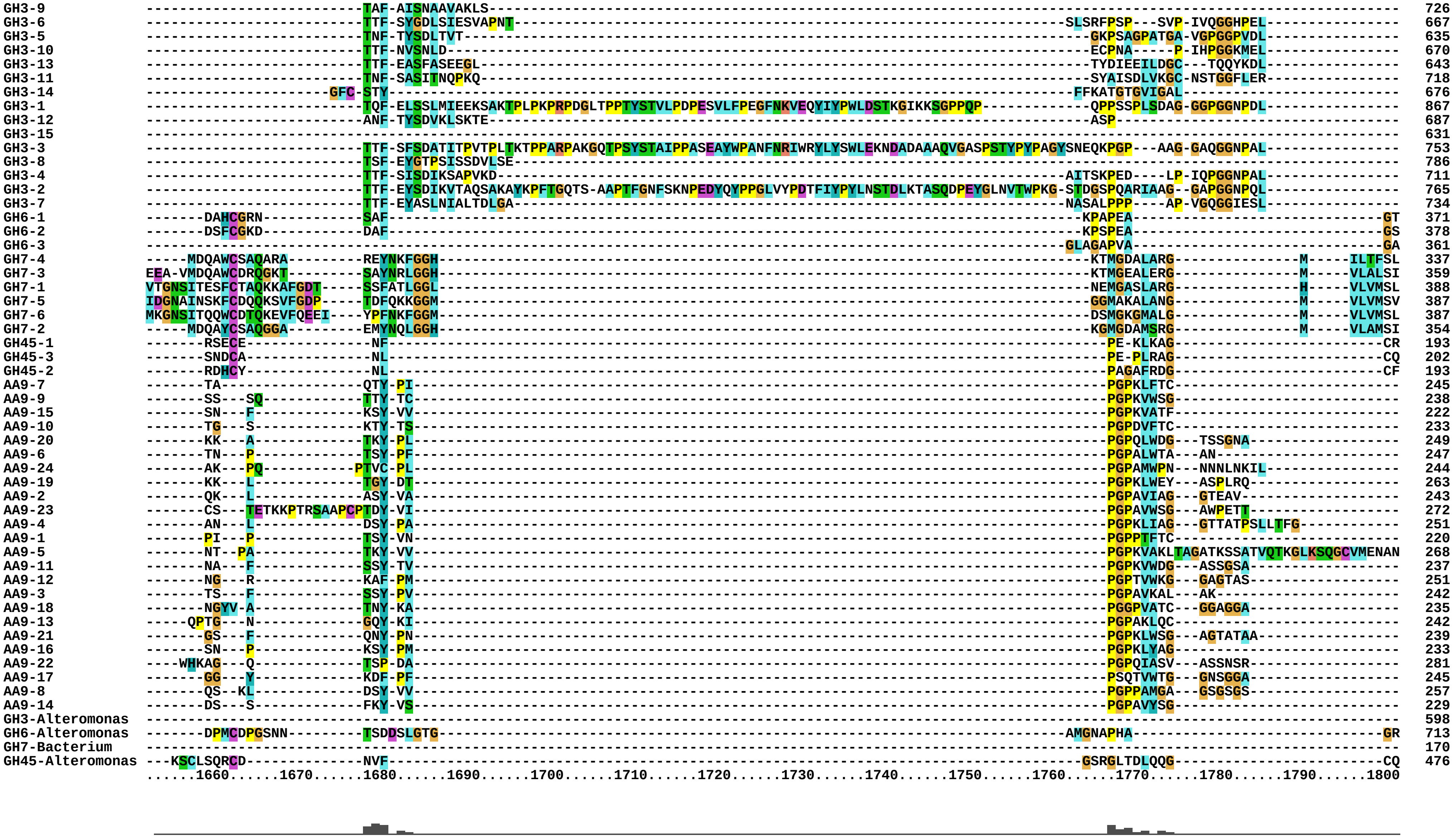
GH3-9 726
GH3-6 667
GH3-5 635
GH3-10 670
GH3-13 643
GH3-11 718
GH3-14 676
GH3-1 867
GH3-12 687
GH3-15 631
GH3-3 753
GH3-8 786
GH3-4 711
GH3-2 765
GH3-7 734
GH6-1 371
GH6-2 378
GH6-3 361
GH7-4 337
GH7-3 359
GH7-1 388
GH7-5 387
GH7-6 387
GH7-2 354
GH45-1 193
GH45-3 202
GH45-2 193
AA9-7 245
AA9-9 238
AA9-15 222
AA9-10 233
AA9-20 249
AA9-6 247
AA9-24 244
AA9-19 263
AA9-2 243
AA9-23 272
AA9-4 251
AA9-1 220
AA9-5 268
AA9-11 237
AA9-12 251
AA9-3 242
AA9-18 235
AA9-13 242
AA9-21 239
AA9-16 233
AA9-22 281
AA9-17 245
AA9-8 257
AA9-14 229
GH3-Alteromonas 598
GH6-Alteromonas 713
GH7-Bacterium 170
GH45-Alteromonas 476
1660 1670 1680 1690 1700 1710 1720 1730 1740 1750 1760 1770 1780 1790 1800

```
GH3-9            --------------------------------------------------------------------------------SGAFNASASVTNTGSVA------------GAVVVQLYVGRKER--------SDEHPVKTLAA--FHKVD-  773
GH3-6            -----------------------------------------------------------W--------------------DVLYRVSVNVTNTGKVA------------GHEVPQLYLGVPNA------------PQKQLRG--FERVGL  712
GH3-5            -----------------------------------------------------------F--------------------ETVATVTAKIANSGGVA------------GAEVPQLYLGYPAS---------TNSPPKQLRG--FTKLK-  682
GH3-10           -----------------------------------------------------------W--------------------DILVTVEATVTNTGSRL------------GAAVPQLYLSYPSE---------AKMPVRSLRG--FDKID-  717
GH3-13           --------------------------------------------------------------------------------CEVAPLEVTVANKGNRT------------SDFVALAFIKGEVG--------PKPYPLKTLIT--YGRLRD  691
GH3-11           --------------------------------------------------------------------------------CPFTGINVSVQNTGKTS------------SDYVTLGFLTGSFG--------PKPYPKKSLVA--YDRLFN  766
GH3-14           -----------------------------------------------------------FV--------D-PA-------RRKLSIGHSLHNRAIRTLLQKEGIKRFQLGSRLPSIYLGIPTGH---------GNERKRLRP-WFAQLG-  740
GH3-1            -----------------------------------------------------------Y--------------------TILLTVSASLENKGPLD------------GASVVQLYISLPQDYKDHETGEPVDFPVRVLRG--FEKVQ-  923
GH3-12           --------------------------------------------------------------------------------SDTVTVTVSVTNSSPVD------------GKEVVQVYVQDVFA--------SVVVPNKELKG--FKKVL-  734
GH3-15           ---------------------------------------------------------------------E--------------------------------------------------------------------------------  632
GH3-3            -----------------------------------------------------------F--------------------DVAYDVSVTVTNTGSRS------------GKAVAQLYLQFPNE-------STVDTPILQLRD--FAKTST  803
GH3-8            --------------------------------------------------------------------------------SDDITITVPVTNTGSVA------------APEIVQGYVKDVKS--------TLPRPEKELQA--FGKVF-  833
GH3-4            -----------------------------------------------------------W--------------------ECIYNVTVSVANTGDVA------------GAAVPQLYVGLPSS-------APAGTPVRQLRG--FEKVY-  760
GH3-2            -----------------------------------------------------------W--------------------DVLFKVEATITNTGDVA------------GDEVAQAYISLGGP----------HDPKVQLRD--FDRLT-  811
GH3-7            -----------------------------------------------------------W--------------------DVLVAVKAEITNTGLTS------------APEVAQLYLGIPGA------------PAKQLRG--FEKVP-  778
GH6-1            WFQAYFEML-------------------------------------------------------------------------------------------------------------------------LKNANPALA-----------  389
GH6-2            WNQAYFEAL-------------------------------------------------------------------------------------------------------------------------VKNANPPL------------  395
GH6-3            WFDDYVQML-------------------------------------------------------------------------------------------------------------------------VKNADPPLEPTY--------  382
GH7-4            WWDKNGGMQ--------------------------------------------------WL--------D----------GG-------------LN------------GPCSPAEDFPDAIQQ-------KVKNPTVTFSQIKWGEIG-  386
GH7-3            WWDKSGGMQ--------------------------------------------------WL--------D----------SG-------------SS------------GPCNATEGFPDVIQS-------KVKNPTVTFSQVKWGEID-  408
GH7-1            WDDHAVNML--------------------------------------------------WL--------D----------STYPTDADPSKP-GAAR------------GSCPTTSGKPEDVEK-------NSPDATVVFSNIKFGPIG-  449
GH7-5            WDDHNSNML--------------------------------------------------WL--------D----------STYPTDKDPEKDLGVAR------------GECATTSGVPKDLEA-------NNADATVTYSNIKFGPLN-  449
GH7-6            WDDHYANML--------------------------------------------------WL--------D----------GKYPVDADPAKP-GVAR------------GACEAGSGVPADVEA-------NSPNAQVVFSNIKFGPIG-  448
GH7-2            WWDEGGAMQ--------------------------------------------------WL--------D----------SG-------------AS------------GPCNATEGFPAEIVK-------KVKNPTVTFSKIKWGEIG-  403
GH45-1           WRFDWFK----------------------------------------------------------------------------------------------------------------------------NADNPTISFKE------V-  212
GH45-3           WRFDWLQ----------------------------------------------------------------------------------------------------------------------------DASFPSANFKR------V-  221
GH45-2           WRFDWFK----------------------------------------------------------------------------------------------------------------------------NADNPSVDFRE------V-  212
AA9-7            ------------------------------------------------------------------------------------------------------------------------------------------------------  245
AA9-9            ------------------------------------------------------------------------------------------------------------------------------------------------------  238
AA9-15           ------------------------------------------------------------------------------------------------------------------------------------------------------  222
AA9-10           ------------------------------------------------------------------------------------------------------------------------------------------------------  233
AA9-20           ------------------------------------------------------------------------------------------------------------------------------------------------------  249
AA9-6            ------------------------------------------------------------------------------------------------------------------------------------------------------  247
AA9-24           ------------------------------------------------------------------------------------------------------------------------------------------------------  244
AA9-19           ------------------------------------------------------------------------------------------------------------------------------------------------------  263
AA9-2            --------------------------------------------------------------------------------AGQAGSVVTATGGPPVA------------TP---------------------------------------  262
AA9-23           --------------------------------------------------------------------------------AVALGPIKGVSTATPWSTWIG--------TNSVVTSATYVDAKS---QTIVGSSKYTATWSE-TY-----  325
AA9-4            --------------------------TLSGAPAPVAGA---------------------------------PADEAPVASASSAAASTPAASSTPAA------------TPSSSVEDAKPSAAP---------TEPSATPAE-TPAPVV-  319
AA9-1            ------------------------------------------------------------------------------------------------------------------------------------------------------  220
AA9-5            WCAKEVQSYTDEKGCWAAAEDCWAQSDVCYDSAPPTGGKNCKLWQEKCTDIGAQCKAGNWVGPPNKGKDLTPAKKSIDVGLVMPNQVQPAAPSKETP------------APSSEKEQEEPESKP----EPKPETKPENSEYQ--------  394
AA9-11           ------------------------------------------------------------------------------------PSTTPSNGSAPST------------PKPSPVEAAQPATP----------SKPAEN-----------  270
AA9-12           --------------------------------------------------------------------------------AGSSPAAPANNAPAAPA------------ATTLA-------------------TKPAPA-----------  279
AA9-3            ------------------------------------------------------------------------------------------------------------------------------------------------------  242
AA9-18           -----------------------------------------------------------------------------------APVAPPASNSTEPA------------PSSIADEAAAPTLAP---------TVPSGND----------  271
AA9-13           ------------------------------------------------------------------------------------------------------------------------------------------------------  242
AA9-21           --------------------------DAAPAPAPAAGA---------------------------------P-----------AASPSPATGAPSTE------------APSSEAPAAEEPEEC---------TDDTAEPA---------  289
AA9-16           ------------------------------------------------------------------------------------------------------------------------------------------------------  233
AA9-22           ------------------------------------------------------------------------------------------------------------------------------------------------------  281
AA9-17           ----------------------------------------------------------------------------------QSPATSPSSSPSPSAAATKPQATP---APPAPKPQPSQPATPVEQPAGVPVTQPSSAPVE--------  302
AA9-8            --------------------------------------------------------------------------------GSTPAPSKPAPSSTPSS------------SPSAPSPSASAPAAS----VTPEAQKPSPS-----------  300
AA9-14           ------------------------------------------------------------------------------------------------------------------------------------------------------  229
GH3-Alteromonas  ---------------------------------------------------------------------------------------------------------------------------------------------------LS-  600
GH6-Alteromonas  WFPEAFETF-------------------------------------------------------------------------------------------------------------------------IQNAYPEL------------  730
GH7-Bacterium    ------------------------------------------------------------------------------------------------------------------------------------------------------  170
GH45-Alteromonas WYVDWFE----------------------------------------------------------------------------------------------------------------------------AADNPSLKYRE------V-  495
                 ......1810......1820......1830......1840......1850......1860......1870......1880......1890......1900......1910......1920......1930......1940......1950
```

```
GH3-9             LLPGEETAVLLPFANRDFAYFDE---------------------EAKEWVVEK------------------------GLYEFSFAQSAGH-VDEVIEVEVEGVRGLKI------------------------------------------   835
GH3-6             LKPGESAVVDFELQRRDLSIWNV---------------------EAQAWELQR------------------------GRYGVWVGASSRD-VRQEGDIIVQ-------------------------------------------------   767
GH3-5             LEAGANGTATFKLRRRDLSFWDE---------------------KTRKWTVAT------------------------GEYTVFVGASSRD-VRLTGKIIVHLPNKSPNQIPLYTTSSIIMISQLFKPSTVRSAGLLGRATKNRVQARYLA   786
GH3-10            ISPGTTETVLFELTRRDLSCWDT---------------------KVHAWRLPT------------------------GDITVQVRFSSRD-LPLQSIIGI--------------------------------------------------   771
GH3-13            IHGGAKKSASLPLTLGELARVD----------------------QSGNTVIYP------------------------GEYTLLLDEPTQAELKLT----ITGEETILDKWP---------------------------------------   752
GH3-11            IAASSSSTATLNLTLASLARVD----------------------ESGNKVLYP------------------------GDYELQIDNAPLASVKFA----LTGSETTLSKWP---------------------------------------   827
GH3-14            WGAALSRPVC-NMVARNLQAWSPPA------------------GMAKSLASAG----AQFDLVYGWDY--------AGPVLDHIKTSNRQGLAEVYKLALTDPTAC-------------GIIRAKRPEDGALVGTVVLYNSHSRLAEYIP   846
GH3-1             LEKGKGKLVQFEVTRRDLSYWDV---------------------KRQNWVLPM------------------------GEIGIQVGFSSRD-LPLKGK-----------------------------------------------------   974
GH3-12            VKAGETVDVSIDLEVSKWGLWD----------------------RKMKYVVEK------------------------GDFIVHVGSSSLD-LRGNGTVTVV-------------------------------------------------   788
GH3-15            ------------------------------------------------------------------------------------------------------------------------------------------------------   632
GH3-3             LAAGATQTLKMRLTRKDLSVWDV---------------------VEQNWVVPAVG----------------------GDYGVWIGGSSDD-LHLRCGTATGRCQG---------------------------------------------   864
GH3-8             LQPGETKDVVLKLNKYSVGYFDT------------------NLGQTGAWIAEE------------------------GVFEVLIGASSAD-IRAKVSFEVKE------------------------------------------------   892
GH3-4             LEEGETQSVSFELMRRDLSYWDV---------------------VSQQWVIPE------------------------GEFNIWVGLSSRD-LKVHDSFTVVG------------------------------------------------   816
GH3-2             IQPGESVVFTANITRRDVSNWDT---------------------VSQNWVITE------------------------YPKTIHVGASSRN-LPLSAPLDTSSFK----------------------------------------------   869
GH3-7             LEASESTTVHFHLTRRDLSIWDT---------------------VKQSWALQR------------------------GKYMIYVGASSRD-IRLSGSLII--------------------------------------------------   832
GH6-1             ------------------------------------------------------------------------------------------------------------------------------------------------------   389
GH6-2             ------------------------------------------------------------------------------------------------------------------------------------------------------   395
GH6-3             ------------------------------------------------------------------------------------------------------------------------------------------------------   382
GH7-4             STFGT------------------NQ-------------------TLQSWAT---------------------------------------------------------------------------------------------------   400
GH7-3             STYGA------------------SN------------------ATTKYWKA---------------------------------------------------------------------------------------------------   423
GH7-1             STFAQ------------------PA-----------------------------------------------------------------------------------------------------------------------------   456
GH7-5             STFSA-------------------------------------------------------------------------------------------------------------------------------------------------   454
GH7-6             STFAQ------------------PK-----------------------------------------------------------------------------------------------------------------------------   455
GH7-2             STFST------------------KK------------------ATEKRWEA---------------------------------------------------------------------------------------------------   418
GH45-1            TCPGE----------------------------------------------------------------------------------------------LTSRTGCIR------------------------------------------   226
GH45-3            VCPAE----------------------------------------------------------------------------------------------ITAKSNCVRNDD------------------------KRLASGESTSAAYS-   252
GH45-2            SCPQA----------------------------------------------------------------------------------------------MIDRTHCGRWS----------------------------------------   228
AA9-7             ------------------------------------------------------------------------------------------------------------------------------------------------------   245
AA9-9             ------------------------------------------------------------------------------------------------------------------------------------------------------   238
AA9-15            ------------------------------------------------------------------------------------------------------------------------------------------------------   222
AA9-10            ------------------------------------------------------------------------------------------------------------------------------------------------------   233
AA9-20            TMPGS------------------PVP----------------------------------------------STAAMKQRGAGTSGRLRR-YGRC-------------------------------------------------------   279
AA9-6             ------------------------------------------------------------------------------------------------------------------------------------------------------   247
AA9-24            ------------------------------------------------------------------------------------------------------------------------------------------------------   244
AA9-19            -----------------------PYQ----------------------------------------------------------------------------------------------------------------------------   266
AA9-2             ---------------------------------------------------------TQTSTLRTSTVASSAAPAPT-DGGGAGGCSVAK-YGQCGGRGFTGCTTCASGSS------------------------CKASGEFYSQCL---   326
AA9-23            ASPTATA----------------PR-----------------------------------------------------------------------------------------------------------------------------   334
AA9-4             SSPAA------------------PF------------------PVSNSTSSAALPIATSSSAIASTTLATAVRPSPTGDASGDASGSVKE-FYQCGGMNYKGATGCAEGLE------------------------CKQWNPYYFQCVKSG   408
AA9-1             ------------------------------------------------------------------------------------------------------------------------------------------------------   220
AA9-5             PAPAPAKPAPVAEAPKPTPTPDAPAAPANDEYDSPPFMEPTNYPAAPPPAPSKQPTTLSKAPI---------STITVAPSSAGGPKPTQP--------------ACPKGYK------------------------CVYVTDLVVVTTTV-   496
AA9-11            TTPST------------------PSTGNNG---------------------SALPKTFTINEFIQW------LQTTAGSASSKARRHARA---------FN-------------------------------------------------   317
AA9-12            ATPAP------------------------------------------------------------------------------AAGCAAQ-WAQCGGAGFNGAKCCAKGS-------------------------CKTMNPYYSQCQ---   322
AA9-3             -------------------------------------------------------------------------------------RHAKD------------------------------------------------------------   247
AA9-18            TLPSA------------------PAVP---------------------------------STFATLPSPSAGVTAPAGAVPTGSVGTAKL-YYQCGGINYNGPTECEGTAK------------------------CVKQNDFYSQCIN--   343
AA9-13            ------------------------------------------------------------------------------------------------------------------------------------------------------   242
AA9-21            DEPAA------------------PA------------------PAAPAASAPA-------------------------AAPATGGAEAQK-WHQCGGQGYTGATACVSGTS------------------------CVKQNDYYHQCM---   350
AA9-16            ------------------------------------------------------------------------------------------------------------------------------------------------------   233
AA9-22            -------------------------------------------------------------------------------------RHARD---------FSS------------------------------------------------   289
AA9-17            QAPSD----------------DYPAGG--------------DYPAGDDYPTEEAP-----------------KPTPATPSPATPATSKRP---------------CNGGKK------------------------ARRGSRAFRQSLRK-   365
AA9-8             TAPSSG---------------NLPET-------------------------------FTLDTFITW------LEGKASSSASKARRHARA------------------------------------------------------------   338
AA9-14            ------------------------------------------------------------------------------------------------------------------------------------------------------   229
GH3-Alteromonas   W----------------------------------------------------------------------------------------------------------------------------------------RFNQH--------   606
GH6-Alteromonas   ------------------------------------------------------------------------------------------------------------------------------------------------------   730
GH7-Bacterium     ------------------------------------------------------------------------------------------------------------------------------------------------------   170
GH45-Alteromonas  ACPSA----------------------------------------------------------------------------------------------ITSRSGVDRGF----------------------------------------   511
                  ......1960......1970......1980......1990......2000......2010......2020......2030......2040......2050......2060......2070......2080......2090......2100
```

```
GH3-9             ------------------------------------------------------------------------------------------------------------------------------------------------------   835
GH3-6             ------------------------------------------------------------------------------------------------------------------------------------------------------   767
GH3-5             TVQSNTERVMPTPSARRATAVSNEPATFTIKNGPIFEGKSFGAKTNISGEAVFTTSLVGYPESMTDPSYRGQILVFTQPLIGNYGVPSSARDEHGLLRYFESPYIQASGIVVQDYALKHSHWTAVESLGAWCAREGVPAISGVDTREVVT   936
GH3-10            ------------------------------------------------------------------------------------------------------------------------------------------------------   771
GH3-13            -----------------------------------------------------------------------------QP-----------------------------------------------------------------------   754
GH3-11            -----------------------------------------------------------------------------QP-----------------------------------------------------------------------   829
GH3-14            SLKDLNE--------------------------------SAGG--------------------------------ISSP--------------------------------------------------------------------VIS   864
GH3-1             ------------------------------------------------------------------------------------------------------------------------------------------------------   974
GH3-12            ------------------------------------------------------------------------------------------------------------------------------------------------------   788
GH3-15            ------------------------------------------------------------------------------------------------------------------------------------------------------   632
GH3-3             -----------------------------------------------------------------DQ---------TSPV----------------------------------------------------------------------   870
GH3-8             ------------------------------------------------------------------------------------------------------------------------------------------------------   892
GH3-4             ------------------------------------------------------------------------------------------------------------------------------------------------------   816
GH3-2             ------------------------------------------------------------------------------------------------------------------------------------------------------   869
GH3-7             ------------------------------------------------------------------------------------------------------------------------------------------------------   832
GH6-1             ------------------------------------------------------------------------------------------------------------------------------------------------------   389
GH6-2             ------------------------------------------------------------------------------------------------------------------------------------------------------   395
GH6-3             ------------------------------------------------------------------------------------------------------------------------------------------------------   382
GH7-4             ------------------------------------------------------------------------------------------------------------------------------------------------------   400
GH7-3             ------------------------------------------------------------------------------------------------------------------------------------------------------   423
GH7-1             ------------------------------------------------------------------------------------------------------------------------------------------------------   456
GH7-5             ------------------------------------------------------------------------------------------------------------------------------------------------------   454
GH7-6             ------------------------------------------------------------------------------------------------------------------------------------------------------   455
GH7-2             ------------------------------------------------------------------------------------------------------------------------------------------------------   418
GH45-1            --------------------------------------------------------------------------------------------------------L---------------------------------------------   227
GH45-3            --------------------------------------------------------------------------------------------------------LSPSL-----------------------------------------   257
GH45-2            ------------------------------------------------------------------------------------------------------------------------------------------------------   228
AA9-7             ------------------------------------------------------------------------------------------------------------------------------------------------------   245
AA9-9             ------------------------------------------------------------------------------------------------------------------------------------------------------   238
AA9-15            ------------------------------------------------------------------------------------------------------------------------------------------------------   222
AA9-10            ------------------------------------------------------------------------------------------------------------------------------------------------------   233
AA9-20            ------------------------------------------------------------------------------------------------------------------------------------------------------   279
AA9-6             ------------------------------------------------------------------------------------------------------------------------------------------------------   247
AA9-24            ------------------------------------------------------------------------------------------------------------------------------------------------------   244
AA9-19            ------------------------------------------------------------------------------------------------------------------------------------------------------   266
AA9-2             ------------------------------------------------------------------------------------------------------------------------------------------------------   326
AA9-23            ------------------------------------------------------------------------------------------------------------------------------------------------------   334
AA9-4             SSNGSQP----PPS-------NNAPST----GAPSNNAPSTGAPSN------------------NGPS-------NNTPA---------PKPEPTNVTPEAAPSAAPSA-----------------------------------------   468
AA9-1             ------------------------------------------------------------------------------------------------------------------------------------------------------   220
AA9-5             -----------------------------------------------------------Y------------------------------------VTEGAAPTATPT------------------------------------------   509
AA9-11            ------------------------------------------------------------------------------------------------------------------------------------------------------   317
AA9-12            ------------------------------------------------------------------------------------------------------------------------------------------------------   322
AA9-3             ------------------------------------------------------------------------------------------------------------------------------------------------------   247
AA9-18            ------------------------------------------------------------------------------------------------------------------------------------------------------   343
AA9-13            ------------------------------------------------------------------------------------------------------------------------------------------------------   242
AA9-21            ------------------------------------------------------------------------------------------------------------------------------------------------------   350
AA9-16            ------------------------------------------------------------------------------------------------------------------------------------------------------   233
AA9-22            ------------------------------------------------------------------------------------------------------------------------------------------------------   289
AA9-17            ------------------------------------------------------------------------------------------------------------------------------------------------------   365
AA9-8             -----------------------------------------------------------F------------------------------------------------------------------------------------------   339
AA9-14            ------------------------------------------------------------------------------------------------------------------------------------------------------   229
GH3-Alteromonas   ------------------------------------------------------------------------------------------------------------------------------------------------------   606
GH6-Alteromonas   ------------------------------------------------------------------------------------------------------------------------------------------------------   730
GH7-Bacterium     ------------------------------------------------------------------------------------------------------------------------------------------------------   170
GH45-Alteromonas  ------------------------------------------------------------------------------------------------------------------------------------------------------   511
                  ......2110......2120......2130......2140......2150......2160......2170......2180......2190......2200......2210......2220......2230......2240......2250
```

```
GH3-9             ------------------------------------------------------------------------------------------------------------------------------------------------------  835
GH3-6             ------------------------------------------------------------------------------------------------------------------------------------------------------  767
GH3-5             YLREQGSSLARISVGEEYDADEDEAYIDPEAIHLVRRVSTKAPFHVSSSLGDMHVALIDCGVKENILRSLVSRGASVTCFPYDYPIHKVAHHFDGVFISNGPGDPTHCTNTVHNLRKLFETSQLPVMGICMGHQLIALAAGAKTIKLKYG 1086
GH3-10            ------------------------------------------------------------------------------------------------------------------------------------------------------  771
GH3-13            ----------------------------P----------------------------------------------------------------NG------------------------------------------------------G  758
GH3-11            ----------------------------P------------------------------------------------------------------------------------------------------------------------S  831
GH3-14            ----------------------------PGVGEYSTLLQ----------------GLILLG-----MRQIKQLGCNACLLDY----------MDG--------D---------------------------------------------G  902
GH3-1             ------------------------------------------------------------------------------------------------------------------------------------------------------  974
GH3-12            ------------------------------------------------------------------------------------------------------------------------------------------------------  788
GH3-15            ------------------------------------------------------------------------------------------------------------------------------------------------------  632
GH3-3             ------------------------------------------------------------------------------------------------------------------------------------------------------  870
GH3-8             ------------------------------------------------------------------------------------------------------------------------------------------------------  892
GH3-4             ------------------------------------------------------------------------------------------------------------------------------------------------------  816
GH3-2             ------------------------------------------------------------------------------------------------------------------------------------------------------  869
GH3-7             ------------------------------------------------------------------------------------------------------------------------------------------------------  832
GH6-1             ------------------------------------------------------------------------------------------------------------------------------------------------------  389
GH6-2             ------------------------------------------------------------------------------------------------------------------------------------------------------  395
GH6-3             ------------------------------------------------------------------------------------------------------------------------------------------------------  382
GH7-4             ------------------------------------------------------------------------------------------------------------------------------------------------------  400
GH7-3             ------------------------------------------------------------------------------------------------------------------------------------------------------  423
GH7-1             ------------------------------------------------------------------------------------------------------------------------------------------------------  456
GH7-5             ------------------------------------------------------------------------------------------------------------------------------------------------------  454
GH7-6             ------------------------------------------------------------------------------------------------------------------------------------------------------  455
GH7-2             ------------------------------------------------------------------------------------------------------------------------------------------------------  418
GH45-1            ------------------------------------------------------------------------------------------------------------------------------------------------------  227
GH45-3            ----------------------------PHTMAFVAATM---------------------------LTLIS-------------------------------------------------------------------------------  273
GH45-2            ------------------------------------------------------------------------------------------------------------------------------------------------------  228
AA9-7             ------------------------------------------------------------------------------------------------------------------------------------------------------  245
AA9-9             ------------------------------------------------------------------------------------------------------------------------------------------------------  238
AA9-15            ------------------------------------------------------------------------------------------------------------------------------------------------------  222
AA9-10            ------------------------------------------------------------------------------------------------------------------------------------------------------  233
AA9-20            ------------------------------------------------------------------------------------------------------------------------------------------------------  279
AA9-6             ------------------------------------------------------------------------------------------------------------------------------------------------------  247
AA9-24            ------------------------------------------------------------------------------------------------------------------------------------------------------  244
AA9-19            ------------------------------------------------------------------------------------------------------------------------------------------------------  266
AA9-2             ------------------------------------------------------------------------------------------------------------------------------------------------------  326
AA9-23            ------------------------------------------------------------------------------------------------------------------------------------------------------  334
AA9-4             ----------------------------PAAGAGEKKYT---------------------------LETFIAF----------------LEEEAG------------------------------------------------------A  493
AA9-1             ------------------------------------------------------------------------------------------------------------------------------------------------------  220
AA9-5             ----------------------------PSSGSYMKRAH---------------------------LHPRRRL-----------------------------------------------------------------------------  527
AA9-11            ------------------------------------------------------------------------------------------------------------------------------------------------------  317
AA9-12            ------------------------------------------------------------------------------------------------------------------------------------------------------  322
AA9-3             ------------------------------------------------------------------LEY---------------------------------------------------------------------------------  250
AA9-18            ------------------------------------------------------------------------------------------------------------------------------------------------------  343
AA9-13            ------------------------------------------------------------------------------------------------------------------------------------------------------  242
AA9-21            ------------------------------------------------------------------------------------------------------------------------------------------------------  350
AA9-16            ------------------------------------------------------------------------------------------------------------------------------------------------------  233
AA9-22            ------------------------------------------------------------------------------------------------------------------------------------------------------  289
AA9-17            ------------------------------------------------------------------------------------------------------------------------------------------------------  365
AA9-8             ------------------------------------------------------------------L-----------------------------------------------------------------------------------  340
AA9-14            ------------------------------------------------------------------------------------------------------------------------------------------------------  229
GH3-Alteromonas   ------------------------------------------------------------------------------------------------------------------------------------------------------  606
GH6-Alteromonas   ------------------------------------------------------------------------------------------------------------------------------------------------------  730
GH7-Bacterium     ------------------------------------------------------------------------------------------------------------------------------------------------------  170
GH45-Alteromonas  ------------------------------------------------------------------LNDVSN------------------------------------------------------------------------------  517
                  ......2260......2270......2280......2290......2300......2310......2320......2330......2340......2350......2360......2370......2380......2390......2400
```

```
GH3-9             ------------------------------------------------------------------------------------------------------------------------------------------------------  835
GH3-6             ------------------------------------------------------------------------------------------------------------------------------------------------------  767
GH3-5             NRAHN-IPALDLTTGKCHITSQNHGYAVDPTTLTSEWREYFTNLNDQSNEGLIHSSRPIFSAQFHPEAKGGPLDSAYLFDKYIENVQQYKEQQASFSQRSNKPSPLLVDLLAKERVGVHPDAPDFEGHAAGMDSQQVNVGGPVAPPYQPI 1235
GH3-10            ------------------------------------------------------------------------------------------------------------------------------------------------------  771
GH3-13            NRT-------------------------------------------------------VF------------------------------------------------------------------------------------------  763
GH3-11            NRTGQ---GVDF--------------------FGDYWY-----------------------------------------------------------------------------------------------------GGN--------  849
GH3-14            NFDGFSAMGFDVMHNFEEVSCD-----------AATWT--------------------MI------------------------------------------------------------------------------------PP----  933
GH3-1             ------------------------------------W-----------------------------------------------------------------------------------------------------------------  975
GH3-12            ------------------------------------------------------------------------------------------------------------------------------------------------------  788
GH3-15            ------------------------------------------------------------------------------------------------------------------------------------------------------  632
GH3-3             ------------------------------------------------------------------------------------------------------------------------------------------------------  870
GH3-8             ---------------------------------SFQW---------------------IF------------------------------------------------------------------------------------------  898
GH3-4             -------------------------------------------------------------------------------------------------------------------------------H----------------------  817
GH3-2             ------------------------------------------------------------------------------------------------------------------------------------------------------  869
GH3-7             ------------------------------------------------------------------------------------------------------------------------------------------------------  832
GH6-1             ------------------------------------------------------------------------------------------------------------------------------------------------------  389
GH6-2             ------------------------------------------------------------------------------------------------------------------------------------------------------  395
GH6-3             ------------------------------------------------------------------------------------------------------------------------------------------------------  382
GH7-4             ------------------------------------------------------------------------------------------------------------------------------------------------------  400
GH7-3             ------------------------------------------------------------------------------------------------------------------------------------------------------  423
GH7-1             ------------------------------------------------------------------------------------------------------------------------------------------------------  456
GH7-5             ------------------------------------------------------------------------------------------------------------------------------------------------------  454
GH7-6             ------------------------------------------------------------------------------------------------------------------------------------------------------  455
GH7-2             ------------------------------------------------------------------------------------------------------------------------------------------------------  418
GH45-1            ------------------------------------------------------------------------------------------------------------------------------------------------------  227
GH45-3            ----------------------------------------------------------V-------------------------------------------------------------------------------------------  274
GH45-2            ------------------------------------------------------------------------------------------------------------------------------------------------------  228
AA9-7             ------------------------------------------------------------------------------------------------------------------------------------------------------  245
AA9-9             ------------------------------------------------------------------------------------------------------------------------------------------------------  238
AA9-15            ------------------------------------------------------------------------------------------------------------------------------------------------------  222
AA9-10            ------------------------------------------------------------------------------------------------------------------------------------------------------  233
AA9-20            ------------------------------------------------------------------------------------------------------------------------------------------------------  279
AA9-6             ------------------------------------------------------------------------------------------------------------------------------------------------------  247
AA9-24            ------------------------------------------------------------------------------------------------------------------------------------------------------  244
AA9-19            ------------------------------------------------------------------------------------------------------------------------------------------------------  266
AA9-2             ------------------------------------------------------------------------------------------------------------------------------------------------------  326
AA9-23            ------------------------------------W-----------------------------------------------------------------------------------------------------------------  335
AA9-4             EKAAK--------------------------------------------------IRRMIEALF--------------------------------------------------------------------------------------  507
AA9-1             ------------------------------------------------------------------------------------------------------------------------------------------------------  220
AA9-5             -------------------------------------------------------------------------------------------------------------------------------HLA--------------------  530
AA9-11            ------------------------------------------------------------------------------------------------------------------------------------------------------  317
AA9-12            ------------------------------------------------------------------------------------------------------------------------------------------------------  322
AA9-3             ------------------------------------------------------------------------------------------------------------------------------------------------------  250
AA9-18            ------------------------------------------------------------------------------------------------------------------------------------------------------  343
AA9-13            ------------------------------------------------------------------------------------------------------------------------------------------------------  242
AA9-21            ------------------------------------------------------------------------------------------------------------------------------------------------------  350
AA9-16            ------------------------------------------------------------------------------------------------------------------------------------------------------  233
AA9-22            ------------------------------------------------------------------------------------------------------------------------------------------------------  289
AA9-17            ------------------------------------------------------------------------------------------------------------------------------------------------------  365
AA9-8             ------------------------------------------------------------------------------------------------------------------------------------------------------  340
AA9-14            ------------------------------------------------------------------------------------------------------------------------------------------------------  229
GH3-Alteromonas   ------------------------------------------------------------------------------------------------------------------------------------------------------  606
GH6-Alteromonas   ------------------------------------------------------------------------------------------------------------------------------------------------------  730
GH7-Bacterium     ------------------------------------------------------------------------------------------------------------------------------------------------------  170
GH45-Alteromonas  -------------------SCN--------------------------------------------------------------------------------------------------------------------------------  520
                  ......2410......2420......2430......2440......2450......2460......2470......2480......2490......2500......2510......2520......2530......2540......2550
```

```
GH3-9             ---------   835
GH3-6             ---------   767
GH3-5             MQKPVASAA  1244
GH3-10            ---------   771
GH3-13            ---------   763
GH3-11            ---------   849
GH3-14            --------A   934
GH3-1             ---------   975
GH3-12            ---------   788
GH3-15            ---------   632
GH3-3             ---------   870
GH3-8             ---------   898
GH3-4             ---------   817
GH3-2             ---------   869
GH3-7             ---------   832
GH6-1             ---------   389
GH6-2             ---------   395
GH6-3             ---------   382
GH7-4             ---------   400
GH7-3             ---------   423
GH7-1             ---------   456
GH7-5             -Q-------   455
GH7-6             ---------   455
GH7-2             ---------   418
GH45-1            ---------   227
GH45-3            ---------   274
GH45-2            ---------   228
AA9-7             ---------   245
AA9-9             ---------   238
AA9-15            ---------   222
AA9-10            ---------   233
AA9-20            ---------   279
AA9-6             ---------   247
AA9-24            ---------   244
AA9-19            ---------   266
AA9-2             ---------   326
AA9-23            ---------   335
AA9-4             ---------   507
AA9-1             ---------   220
AA9-5             ---------   530
AA9-11            ---------   317
AA9-12            ---------   322
AA9-3             ---------   250
AA9-18            ---------   343
AA9-13            ---------   242
AA9-21            ---------   350
AA9-16            -Q-------   234
AA9-22            ---------   289
AA9-17            ---------   365
AA9-8             ---------   340
AA9-14            ---------   229
GH3-Alteromonas   ---------   606
GH6-Alteromonas   ---------   730
GH7-Bacterium     ---------   170
GH45-Alteromonas  ---------   520
                  .........
```

Supplementary figure S4. Complete CDS sequence of *B. sorokiniana* GH7-3 as obtained after sequencing.

ATGTCACTTTCTTCAGTCGTCGCGGCAACCCTGTTGGGCTTGGCCACTGCCCAAATAGTGGGAACTATACCAGAAGTACACCCAA
AGTTGACGACGTGGAAATGCACAAAGGCTGGTGGATGCAAGGCGCTAGACACAGCCATTGTCGTCGACTCACTACGCCACAAC
ATTCACCTGAAGAACGACACGAGTGTGAGCTGCGGTGATGAATACACTGCGCTCAACACGGCGCTCTGCCCAGACAAGAAGACT
TGCGCCCAGAATTGCGTAATTGATGGCATCGAGGATTACTCCACTCAAGCAATTTTCACGGACAACGACAAGCTTCGCCTAGACA
TGTATAATCCAAAGGGCGAATATATGAGCCCTAGGGTGTATTTGCTAGCCGAGGATAAACAAAACTACGAGATGCTGCAATTGA
CGGGCAATGAGCTATCCTTTGACGTCGACATGTCGAAGCTACCCTGTGGCATGAACAGTGCTCTGTACCTTAGCGAGATGGAAG
CTGATGGTGGGCGGTCCGAATACAATACCGGAGGTGCGCCTCAGGGTAGCGGGTACTGCGATGCCCAGTGTTTTGTCAAACCAT
TTTTGAACGGCGAGGCCAACGCCAATGGCGAGGGCGCCTGCTGCAACGAAATGGATATCTGGGAGGCCAACAGTTATGCCAAC
CAGATCGCCCCCCACACCTGCGCCAAGCCAGGTATCTTTGCATGCACGGGCAATGACTGCGGCAGTGCTGGACTCTGCGACAAG
GCCGGATGCGGTGACAACCCGTATAAGGACCGTAACGACAAGGAATATTACGGACCGGGTCTCAAGGTCGATACCAACAGGCC
ATTCACCGTTGTCACCCAGTTCCCCGCCAAGGACGGCGTGCTCCAGGCCATTGTCCGCAAGTACGTCCAAGATGGTGTGGTCATT
GAAAACGCCATGCGCAACCTCACTGCTGAAGAAGCAGTCATGGACCAGGCGTGGTGCGATAGGCAGGGAAAAACCTCTGCCTA
CAACAGGCTCGGCGGCCACAAGACCATGGGTGAGGCTCTGGAACGCGGTATGGTTCTAGCATTGAGCATCTGGTGGGACAAGA
GCGGAGGCATGCAGTGGCTTGACAGCGGATCCAGCGGGCCTTGCAATGCCACCGAGGGCTTCCCAGATGTCATCCAGTCGAAG
GTCAAGAACCCCACTGTTACCTTTAGCCAGGTCAAGTGGGGTGAGATTGACTCGACGTATGGAGCTTCGAATGCGACAACCAAG
TACTGGAAGGCTCATCATCACCATCATCATTAA

Supplementary figure S5. (a) The upper and lower diagonal in matrix represents the % of identities between the sequences and RMSD (Å) between the structures, respectively, of TrGH7 (7CEL-A), HiGH7 (1OJJ-B) and BsGH7-3. (b) The structural diversities in the binding regions among TrGH7, HiGH7 and BsGH7-3. (c) Comparative electrostatic potential distribution between TrGH7, HiGH7 and BsGH7-3.

(a)	TrGH7	HiGH7	BsGH7-3
TrGH7		38.00	38.00
HiGH7	2.14		53.00
BsGH7-3	2.21	0.39	

(b)	Largest binding pocket (Å)		No of residues in largest positive patch
	Area	Volume	
TrGH7	1777.5	2813.5	12
HiGH7	1652.8	2327.8	25
BsGH7-3	1251.5	2129.2	5

(c)

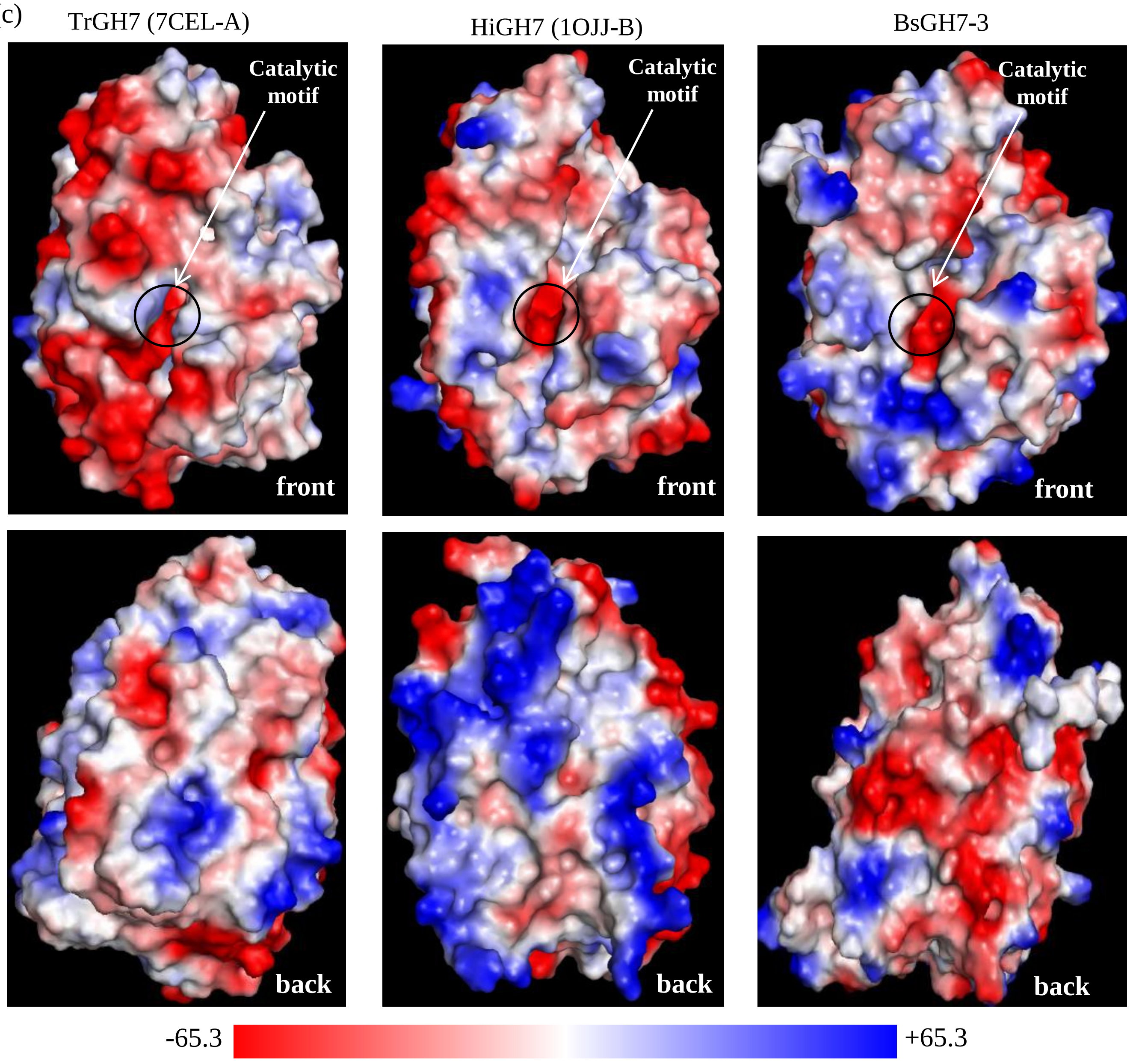

Supplementary table S1: The genomic features of *B. sorokiniana GHs* and *AA9* genes. Details of length of exons and ORF coordinates of *B.sorokiniana GHs* and *AA9* transcripts.

Gene name	Gene ID (JGI)	Transcript length	Exon coordinate (on transcript)	ORF
GH3				
GH3-1	jgi|Cocsa1|208057|CE3403_8724	3298	1-807, 808-3298	236 to 3163
GH3-2	jgi|Cocsa1|40129|fgenesh1_kg.14_#_97_#_Locus1433v1rpkm74.10	2880	1-154, 155-242, 243-615, 616-977, 978-1684, 1685-2880	115 to 2724
GH3-3	jgi|Cocsa1|305206|CE100552_873	2916	1-350, 351-729, 730-943, 944-2916	246 to 2858
GH3-4	jgi|Cocsa1|36266|fgenesh1_kg.6_#_189_#_Locus2312v1rpkm46.93	2641	1-281, 282-2641	110 to 2563
GH3-5	jgi|Cocsa1|172113|fgenesh1_pm.8_#_376	3735	1-46, 47-247, 248-674, 675-783, 784-2208, 2211-2450, 2451-3735	1 to 3735
GH3-6	jgi|Cocsa1|149234|estExt_Genewise1Plus.C_13_t10334	2639	1-405, 406-874, 875-1048, 1049-1117, 1118-2639	283 to 2585
GH3-7	jgi|Cocsa1|94783|e_gw1.10.1255.1	2499	1-259, 260-573, 574-638, 639-1135, 1136-1755, 1756-2499	1 to 2499
GH3-8	jgi|Cocsa1|33954|fgenesh1_kg.3_#_57_#_Locus5209v1rpkm21.01	3254	1-467, 468-595, 596-2868, 2869-3254	227 to 2923
GH3-9	jgi|Cocsa1|147447|estExt_Genewise1Plus.C_11_t20096	2716	1-246, 247-416, 417-2716	127 to 2734
GH3-10	jgi|Cocsa1|176458|fgenesh1_pm.19_#_29	2316	1-264, 265-724, 725-816, 817-977, 978-1969, 1970-2316	1 to 2316
GH3-11	jgi|Cocsa1|182254|estExt_Genemark1.C_9_t10052	2572	1-325, 326-955, 956-2572	1 to 2550
GH3-12	jgi|Cocsa1|209054|CE4400_281	2541	1 to 2541	105 to 2471
GH3-13	jgi|Cocsa1|181585|estExt_Genemark1.C_7_t10380	2379	1-207, 208-2379	16 to 2307
GH3-14	jgi|Cocsa1|192755|estExt_fgenesh1_pg.C_130045	3413	1-167, 174-286, 287-2811, 2812-3413	121 to 2925
GH3-15	jgi|Cocsa1|210264|CE5610_1756	2286	1 to 2286	280 to 2178
GH6				
GH6-1	jgi|Cocsa1|152794|estExt_Genewise1Plus.C_17_t10207	1396	1-1056, 1057-1396	36 to 1205
GH6-2	jgi|Cocsa1|188318|estExt_fgenesh1_pg.C_3_t20040	1188	1-269, 270-442, 443-670, 671-1188	1 to 1188
GH6-3	jgi|Cocsa1|272836|CE68182_498	1299	1-1299	41 to 1189
GH7				
GH7-1	jgi|Cocsa1|32576|fgenesh1_kg.1_#_739_#_Locus8216v1rpkm12.25	1644	1-323, 324-1644	27 to 1397
GH7-2	jgi|Cocsa1|39085|fgenesh1_kg.11_#_385_#_Locus3464v1rpkm31.79	1398	1-653, 654-1398	51 to 1307

GH7-3	jgi|Cocsa1|201219|estExt_fgenesh1_pm.C_100198	1465	1-725, 726-1465	125 to 1369
GH7-4	jgi|Cocsa1|160447|gm1.5168_g	1203	1-552, 553-1203	1 to 1203
GH7-5	jgi|Cocsa1|343966|MIX3290_34_78	1421	1-240, 241-666, 667-1421	54 to 1420
GH7-6	jgi|Cocsa1|351972|MIX11296_12_37	1516	1-471, 472-605, 606-729, 730-1069, 1070-1516	51 to 1418
GH45				
GH45-1	jgi|Cocsa1|40868|fgenesh1_kg.16_#_82_#_Locus110960v1rpkm0.58	1245	1-630, 631-1245	292 to 975
GH45-2	jgi|Cocsa1|135373|estExt_Genewise1Plus.C_2_t60114	887	1-375, 376-887	38 to 724
GH45-3	jgi|Cocsa1|36957|fgenesh1_kg.7_#_289_#_Locus8651v1rpkm11.53	1352	1-522, 523-702, 703-1352	61 to 885
AA9				
AA9-1	jgi|Cocsa1|201224|estExt_fgenesh1_pm.C_100203	926	1-97, 98-424, 425-926	46 to 708
AA9-2	jgi|Cocsa1|173430|fgenesh1_pm.11_#_152	981	1-90, 91-926, 927-981	1 to 981
AA9-3	jgi|Cocsa1|296458|CE91804_3850	1243	1-902, 903-1243	263 to 1015
AA9-4	jgi|Cocsa1|193395|estExt_fgenesh1_pg.C_140322	1654	1-291, 292-576, 577-1654	131 to 1653
AA9-5	jgi|Cocsa1|209542|CE4888_13651	1982	1-210, 211-302, 303-1982	116 to 1708
AA9-6	jgi|Cocsa1|141568|estExt_Genewise1Plus.C_6_t30009	1110	1-451, 452-672, 673-1110	98 to 841
AA9-7	jgi|Cocsa1|113504|estExt_Genewise1.C_4_t40025	1053	1-561, 562-871, 872-1053	179 to 916
AA9-8	jgi|Cocsa1|36761|fgenesh1_kg.7_#_93_#_Locus5809v1rpkm18.51	1364	1-1364	176 to 1198
AA9-9	jgi|Cocsa1|116305|estExt_Genewise1.C_6_t20397	917	1-271, 272-536, 237-917	168 to 884
AA9-10	jgi|Cocsa1|140540|estExt_Genewise1Plus.C_5_t40151	820	1-153, 154-570, 571-820	31 to 732
AA9-11	jgi|Cocsa1|217959|CE13305_3528	1277	1-1277	251 to 1204
AA9-12	jgi|Cocsa1|245914|CE41260_5930	1419	1-770, 771-1419	136 to 1104
AA9-13	jgi|Cocsa1|33678|fgenesh1_kg.2_#_719_#_Locus23111v1rpkm3.09	1060	1-460. 461-752, 753-1060	201 to 929
AA9-14	jgi|Cocsa1|41199|fgenesh1_kg.17_#_102_#_Locus15900v1rpkm5.17	1060	1-380, 381-1060	176 to 865
AA9-15	jgi|Cocsa1|138675|estExt_Genewise1Plus.C_4_t30477	910	1-122, 123-458, 459-910	59 to 727
AA9-16	jgi|Cocsa1|34302|fgenesh1_kg.3_#_405_#_Locus4279v1rpkm25.64	1154	1-787, 788-1154	270 to 974
AA9-17	jgi|Cocsa1|36406|fgenesh1_kg.6_#_329_#_Locus15854v1rpkm5.18	1377	1-1377	163 to 1260
AA9-18	jgi|Cocsa1|32133|fgenesh1_kg.1_#_296_#_Locus2463v1rpkm43.96	1305	1-654, 655-1305	149 to 1180
AA9-19	jgi|Cocsa1|164026|gm1.8747_g	801	1-801	1 to 801
AA9-20	jgi|Cocsa1|140970|estExt_Genewise1Plus.C_6_t10336	894	1-100, 101-189, 190-373, 374-544, 545-894	1 to 840

AA9-21	jgi|Cocsa1|33777|fgenesh1_kg.2_#_818_#_Locus56599v1rpkm1.15	1648	1-390, 391-695, 696-1648	153 to 1205
AA9-22	jgi|Cocsa1|34692|fgenesh1_kg.3_#_795_#_Locus2472v1rpkm43.79	1432	1-1432	166 to 1188
AA9-23	jgi|Cocsa1|184710|estExt_Genemark1.C_150184	1151	1-155, 156-788, 789-1151	84 to 1091
AA9-24	jgi|Cocsa1|155289|gm1.10_g	735	1-308, 309-735	1 to 735

Supplementary table S2.

(a) Details of primers used for qPCR analysis of *B. sorokiniana GHs* transcripts.

S. No.	Gene name	Forward primer (5’→3’)	Length	Reverse primer (5’→3’)	Length
1	*Elongation factor 1*	GCTGGTGACTCCAAGAACGA	20	GAACTTGCAGGCAATGTGGG	20
2	*GH6-1*	ATCAACGCTTTAGCTCCTCTTCTC	24	CCTGGTCGACGATGAAGTGA	21
3	*GH6-2*	GAAAGGTCTTCTCTCCGCAAC	22	GGAGAAGAGCATACTGCAGC	21
4	*GH6-3*	GTCACACAGTTCGTTTTGGC	20	CGACAACATAGCGGTCCTTG	20
5	*GH7-1*	GTACAGGACACTCGCCTTCG	20	AGTGCTGGTGCCAACCTG	18
6	*GH7-2*	GCACGTTATGCACTTCTTGCG	21	GCATCCACCACGCTTGG	17
7	*GH7-3*	CACTTTCTTCAGTCGTCGCG	19	GTGTCTAGCGCCTTGCATC	18
8	*GH7-4*	GACCACACCTGAAGTGCATC	20	CCAGCGTTTGTGCATTTCCAG	21
9	*GH7-5*	GAACGCAGAAGGAGGAAGTG	20	GGGTGGTACAACTTCCTGG	19
10	*GH7-6*	GGAACCCAACAAACAGAGACG	21	CGATTTCTCCATTCACGTTACTGC	24
11	*GH45-1*	TGTCGTCCTCGCCACTG	17	CAGGAGCCTTTGCAACAGTC	20
12	*GH45-2*	CTAGCACGCTTGTCGCTC	18	AGTCGTCACACCTCGTTCAC	20

(b) Details of primers used for cloning of *BsGH7-3* transcripts.

S. No.	Gene name	Forward primer (5’→3’)	Length	Reverse primer (5’→3’)	Length
1	*GH7-3*	CCGCTCGAGAAAAGAATGTCACTTTCT	27	TTGCGGCCGCTTAATGATGATGGTGATGATGAGCCT	36

Supplementary table S3.

Comparison of BsGH7-3 with other fungal endoglucanases of the GH7 family with CMC as the substrate.

Species	T_{opt} (ºC)	pH_{opt}	Specific activity (µmoles/min/mg)	Half life (h)	Reference
Trichoderma harzianum (ThCel7B)	55	3	26	60% after 100 days at 55 ºC	[1]
Bipolaris sorokiniana (BsGH7-3)	60	8.1	5.967	66 % after 15 days (365 h)	In this study
Trichoderma reesei (TrEG1)	60	4.5	10.28	> 5 days at 50 ºC	[2]
Myceliophthora thermophila (MtEG7a)	60	5	177	9.96 h at 70 ºC	[3]
*Cryptococcus sp.*S2	40-50	3.5	4.93	1 h at 90 ºC	[4]
Neosartorya fischeri P1 (Cel7A)	60	5	375 ± 3	1 h at 70 ºC	[5]
Talaromyces emersonii (EGL7A)	70	4.5	791	20 % after 1 h at 75 ºC	[6]
*Bispora sp.*MEY-1 (Bgl7A)	60	5	395	< 5 mins at 70 ºC	[7]

References:

1. Pellegrini VO, Serpa VI, Godoy AS, Camilo CM, Bernardes A, Rezende CA, Junior NP, Franco Cairo JP, Squina FM, Polikarpov I: **Recombinant *Trichoderma harzianum* endoglucanase I (Cel7B) is a highly acidic and promiscuous carbohydrate-active enzyme**. *Appl Microbiol Biotechnol* 2015, **99**(22):9591-9604.
2. Chokhawala HA, Roche CM, Kim T-W, Atreya ME, Vegesna N, Dana CM, Blanch HW, Clark DS: **Mutagenesis of *Trichoderma reesei* endoglucanase I: impact of expression host on activity and stability at elevated temperatures**. *BMC Biotechnology* 2015, **15**(1):1-12.
3. Karnaouri AC, Topakas E, Christakopoulos P: **Cloning, expression, and characterization of a thermostable GH7 endoglucanase from *Myceliophthora thermophila* capable of high-consistency enzymatic liquefaction**. *Appl Microbiol Biotechnol* 2014, **98**(1):231-242.
4. Thongekkaew J, Ikeda H, Masaki K, Iefuji H: **An acidic and thermostable carboxymethyl cellulase from the yeast *Cryptococcus sp.* S-2: purification, characterization and improvement of its recombinant enzyme production by high cell-density fermentation of *Pichia pastoris***. *Protein Expr Purif* 2008, **60**(2):140-146.
5. Liu Y, Dun B, Shi P, Ma R, Luo H, Bai Y, Xie X, Yao B: **A Novel GH7 Endo-β-1, 4-Glucanase from *Neosartorya fischeri* P1 with Good Thermostability, Broad Substrate Specificity and Potential Application in the Brewing Industry**. *PloS ONE* 2015, **10**(9):e0137485.
6. Wang K, Luo H, Shi P, Huang H, Bai Y, Yao B: **A highly-active endo-1, 3-1, 4-β-glucanase from thermophilic *Talaromyces emersonii* CBS394. 64 with application potential in the brewing and feed industries**. *Process Biochem* 2014, **49**(9):1448-1456.
7. Luo H, Yang J, Yang P, Li J, Huang H, Shi P, Bai Y, Wang Y, Fan Y, Yao B: **Gene cloning and expression of a new acidic family 7 endo-β-1, 3-1, 4-glucanase from the acidophilic fungus *Bispora sp.* MEY-1**. *Appl Microbiol Biotechnol* 2010, **85**(4):1015-1023.
